# Direct solar energy charging of metal||air batteries enabled by photo-coupled electrodes

**DOI:** 10.1038/s41467-026-73926-z

**Published:** 2026-06-02

**Authors:** Xinlong Fu, Yi Wang, Changshui Huang, Feng He, Ruiqiao Wu, Qian Chang, Jingxiang Yang, Yuliang Li

**Affiliations:** 1 Beijing National Laboratory for Molecular Sciences (BNLMS), Institute of Chemistry, Chinese Academy of Sciences, Beijing, China; 2 School of Chemical Sciences, University of Chinese Academy of Sciences, Beijing, China

**Keywords:** Batteries, Energy, Solar energy, Batteries, Two-dimensional materials

## Abstract

The realization of solar-charging within rechargeable batteries has been a dream of several generations of scientists, marking a transformation in sustainable energy storage. The key challenge is that the photo-rechargeable electrodes need to simultaneously possess high photovoltaic efficiency and cycling stability. Herein, through dynamic reconfiguration of *sp*-hybridized carbon networks via direct photoexcitation, we present nitrogen-substituted graphdiyne as a metal-free photoelectrode for integrated solar-charging in rechargeable batteries. Nitrogen-substituted graphdiyne accelerates oxygen evolution reaction kinetics by the synergistic effect of improved intermediate adsorption and hole-mediated oxidation under light excitation. Nitrogen-substituted graphdiyne-based photo-coupled positive electrodes are applicable to multiple metal||air batteries (Zn||air, Li||O_2_, Mg||air, Fe||air, and Al||air), including a low charging voltage of 1.33 V and 96.9% energy efficiency in Zn||air batteries, along with stability over 230 cycles at 100 mA cm^−2^. The Li||O_2_ battery achieved an efficiency of 96.3%, while Mg||air, Fe||air, and Al||air systems exhibited reduced charging voltages. This research has pioneered a class of photoelectrodes whose active sites are directly and dynamically defined by light, opening avenues for high-efficiency solar-driven energy conversion and storage.

## Abstract (web summary)

Rechargeable metal||air batteries require high overpotentials. Here, authors reveal that light can dynamically reconfigure the sp-C network in nitrogen-substituted graphdiyne, creating transient catalytic sites that lower the charging voltage and enable stable photo-coupled metal||air batteries.

## Introduction

The direct conversion of solar energy into storable electrical energy within rechargeable batteries presents a route and transformative approach for achieving the efficient utilization of abundant and clean solar energy to meet growing energy demands in an environmentally friendly way^1–3^. This concept is particularly compelling when paired with rechargeable metal||air batteries (RMABs), including Zn||air, Li||O_2_, Mg||air, and related systems^4^^,^^5^^,^^6^^,^^7^. Such systems are attractive and have already become promising candidates in the field of sustainable energy storage. Due to their high theoretical specific energies (exceeding 1000 Wh kg^−1^, rivaling fossil fuels), inherent safety, and environmental compatibility^8–14^, they help address major scientific issues in sustainable energy storage and contribute to achieving the dual-carbon strategy. Although the combination of solar-powered charging and RMAB brings great advantages, practical implementation faces a critical bottleneck, which is the absence of highly efficient and stable photoelectrodes^15,16^. The photoelectrodes, which scientists have been eagerly awaiting, are a key component for the integration of solar energy with a battery. The current development is unsatisfactory, limiting the full-scale realization of this transformative energy storage system. Another key issue is that during the charging process of RMABs, the sluggish kinetics of the oxygen evolution reaction (OER) at the air positive electrode result in high overpotentials^17,18^, severely compromising energy efficiency and cycle life^19–22^. This challenge is compounded by the reliance of current positive electrodes on scarce and expensive noble metal catalysts (e.g., Pt, IrO_2_), which impose high cost and scalability constraints^23,24^. Furthermore, high charging voltages induce side reactions that accelerate air electrode degradation, further shortening the cycle life of RMABs^25–28^.

In the face of numerous complex issues, a large number of studies have focused on the combination of solar energy and photocatalytic technology, providing innovative strategies to address the current situation. By integrating photocatalysis directly into the air electrode to form a photo-coupled electrode, photon energy can generate electron–hole pairs that actively participate in or accelerate electrode reactions^29–31^. This photo-electrochemical synergy reduces charging voltages and diminishes dependence on precious-metal catalysts^32–34^. Recent studies on semiconductor-based photoelectrodes have experimentally demonstrated the fact that the overpotential decreases under light irradiation^35,36^. However, the existing designs of photo-coupled RMABs predominantly rely on traditional semiconductor heterojunctions or bandgap engineering, and there are intrinsic and interrelated obstacles in their applications ^37–39^.

In these systems, light primarily serves as an external energy source for the generation of charge carriers, while catalytically active sites remain statically unaltered, and show negligible photo-induced changes in electronic structure or intrinsic activity^40,41^. Consequently, these systems often suffer from insufficient catalytic site activation, suboptimal charge separation under operational conditions, and material degradation in harsh electrochemical environments. To overcome these issues, through in-depth analysis and reflection, we propose a concept for the direct photoexcitation of the catalytic units themselves. Specifically, by targeting *sp*-hybridized carbon (*sp*-C) domains, photon absorption can dynamically reconfigure local electronic density, creating transient, highly active sites that are fundamentally distinct from their ground-state counterparts^42–48^. This approach represents a fundamental shift from utilizing light solely to generate external charge carriers to actively engineering the intrinsic properties of catalytic centers in real-time through direct photoexcitation.

In this study, to address the critical issues in photo-coupled RMABs, we introduce nitrogen-substituted graphdiyne (NGDY) as a metal-free photoelectrode, capitalizing on the distinctive photoexcitation dynamics inherent to the *sp*-C structures of NGDY. Light-irradiated X-ray photoelectron spectroscopy (XPS) provides direct evidence of photo-induced charge redistribution within NGDY, revealing an intrinsic transformation of catalytic centers under optical excitation. Operando Raman spectroscopy further elucidates accelerated OER kinetics at the NGDY positive electrode under operational light conditions. Unlike conventional approaches that rely on photo-generated holes at static sites, our findings demonstrate that light-induced electron reorganization generates transient, high-activity sites, fundamentally enhancing OER dynamics. This mechanistic insight enables high-performance photo-coupled RMABs (Zn||air, Li||O_2_, Fe||air, Mg||air). Notably, the photo-coupled Zn||air battery (PZAB) achieves low charging voltages (compared with commercial Pt/C and IrO_2_) at 100 mA cm^−2^, while the NGDY-based photo-coupled Li||O_2_ battery (PLOB) attains 96.3% energy efficiency under light irradiation. This work establishes photoexcited *sp*-C materials as a transformative platform for direct solar-energy conversion in next-generation RMABs, advancing high-power, sustainable energy storage.

## Results

### Synthesis and characterization of photo-coupled air electrode

**Fig. 1:**
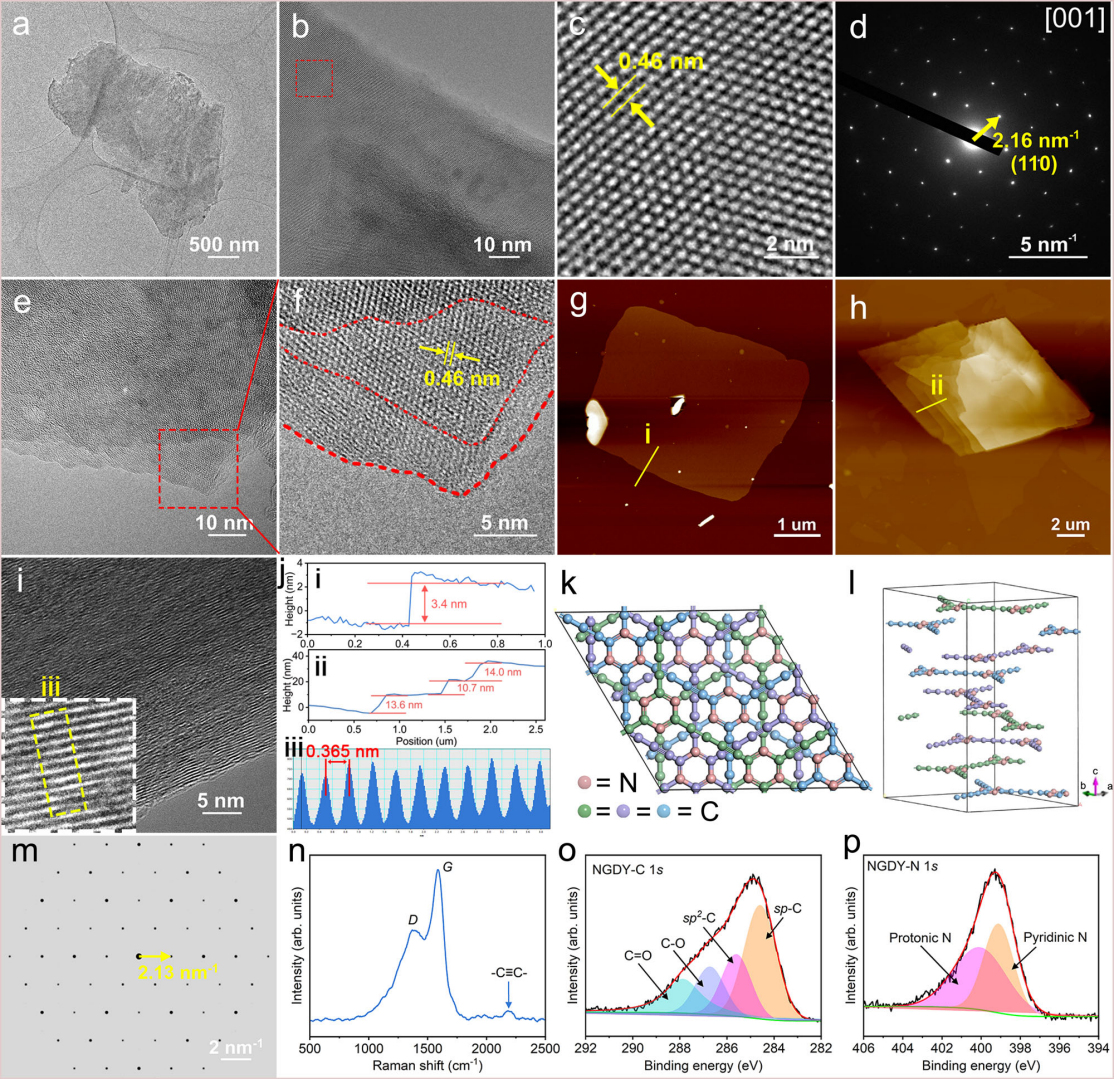
Morphology and structural characterization. **a** TEM image of NGDY. **b**, **c** HR-TEM images of NGDY. **d** SAED pattern of NGDY. **e** HR-TEM image of stacked crystalline NGDY nanosheets. **f** The magnified HR-TEM image. **g** AFM image of NGDY nanosheet. **h** AFM image of stacked NGDY nanosheets. **i** HR-TEM image of NGDY showing interlayer spacing. **j** The cross-sectional analysis along the line in (**g**)–(**i**). **k** Top view and **l** side view of the crystal structure of NGDY with 9-layers stacking structure. **m** Simulated SAED pattern using the 9-layers stacking structure mode. **n** Raman spectrum of NGDY. **o** XPS C 1 *s* spectrum of NGDY. **p** XPS N 1 *s* spectrum of NGDY. Source data are provided as a Source Data file.

NGDY nanosheets were synthesized in-situ on carbon cloth (CC) coated with copper foil using a modified Glaser-Hay coupling reaction. After the reaction was completed, the color of CC changed from gray to black (Supplementary Fig. 3), indicating the successful synthesis of NGDY on CC. NGDY powder was obtained by ultrasonic exfoliation from the copper foil. Scanning electron microscopy (SEM) images (Supplementary Figs. 4 and 5) reveal that, upon reaction completion, the originally smooth CC surface became uniformly coated with a porous NGDY film, which enhances mass transport during electrocatalysis. The energy-dispersive spectroscopy elemental mapping shows a uniform distribution of C and N elements in NGDY (Supplementary Fig. 6). Transmission electron microscopy (TEM) characterization exhibited crystalline structures of NGDY nanosheets (Fig. 1a, b and Supplementary Fig. 7). High-resolution TEM (HRTEM) revealed NGDY with a measured lattice spacing of 0.46 nm (Fig. 1c), corresponding to the (110) plane of its in-plane hexagonal lattice^49,50^. The corresponding selected area electron diffraction pattern (SAED) shows hexagonal symmetry in the NGDY, indicating the existence of C6 rotational symmetry and long-range order (Fig. 1d) ^51^.

The morphology of NGDY was further characterized, alongside a comprehensive structural analysis. TEM image also revealed layers of stacked crystalline NGDY nanosheets (Fig. 1e), which preserved high crystallinity at the edges of the stacks (Fig. 1f). Atomic force microscopy (AFM) images demonstrated that the NGDY nanosheets are flat (Fig. 1g), with a thickness of 3.4 nm (Fig. 1j). AFM further identified layers of stacked trapezoidal NGDY nanosheets (Fig. 1h). The spacing of 0.365 nm (Fig. 1i, j) corresponds to the interlayer distance within the stacked nanosheet structure, suggesting that an individual NGDY nanosheet consists of roughly nine stacked layers. To further validate, a comprehensive study was conducted to compare the experimental data with computational models of the NGDY nine-layer stacking configuration (Supplementary Fig. 8), derived from its optimized crystal structure (Fig. 1k, l). The SAED pattern simulated from the nine-layer stacking model displayed hexagonal symmetry and showed perfect match with the experimental pattern (Fig. 1m), thereby validating the proposed stacking model^52^. We further characterized the chemical structure of NGDY. The Raman spectrum showed *D* and *G* peaks of the triazine ring at 1368 cm^−1^ and 1583 cm^−1^, respectively (Fig. 1n). Two characteristic peaks of the conjugated diyne chain within the *sp*-C framework were observed at 1966cm^−1^ and 2180 cm^−1^, respectively. XPS quantification established an N:C atomic ratio of 1:3, consistent with stoichiometric NGDY (Fig. 1o-p) ^53,54^.

**Fig. 2:**
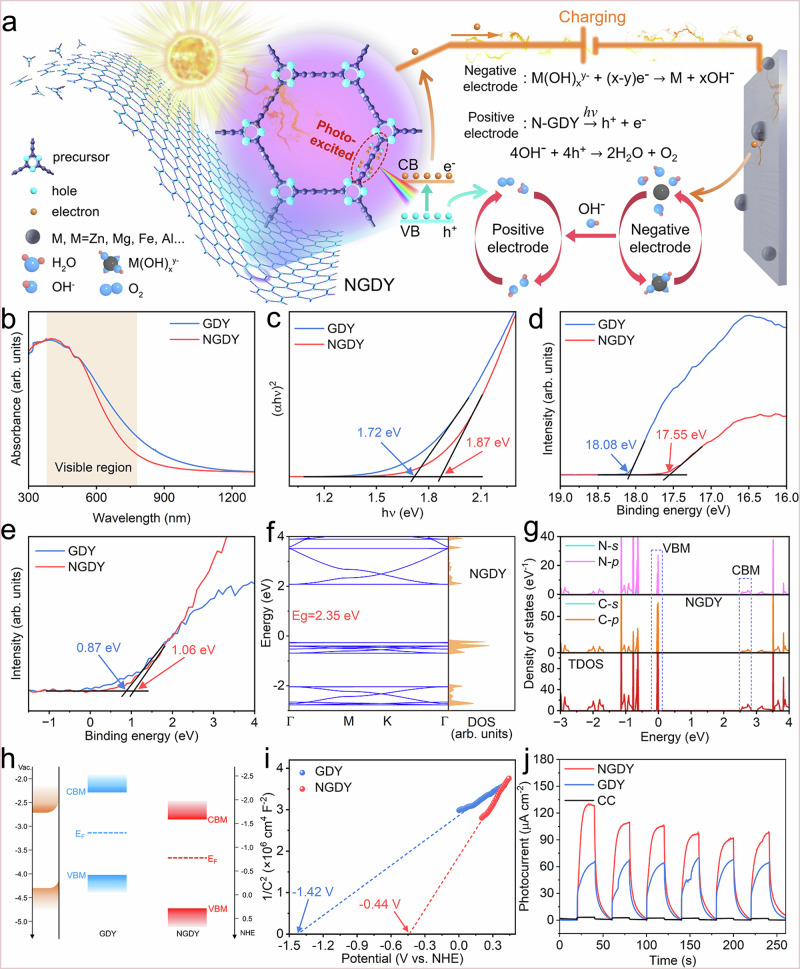
Band structure and photoelectrochemical performance. **a** Schematic illustration of the photoexcited *sp*-C for photo-coupled air electrodes. **b** Ultraviolet-visible spectra of GDY and NGDY. **c** Tauc plots of GDY and NGDY. **d** UPS spectra and (e) XPS valence band spectra of GDY and NGDY. **f** The calculated band structure and density of states of NGDY. **g** PDOS of NGDY. **h** Band structure diagram of GDY and NGDY. E_F_: Fermi level. **i** Mott–Schottky plots of GDY and NGDY. **j** Photocurrent densities under intermittent light irradiation without applied bias voltages (1 M KOH, light source: 320–800 nm). Source data are provided as a Source Data file.

As intrinsic semiconductors, GDY and its derivatives have demonstrated photocatalytic and conversion properties^2,31^, rendering NGDY a promising photo-coupled electrode to achieve high-performance photo-coupled batteries (Fig. 2a). The intrinsic bandgap of the NGDY has been studied to show the impact of the N element on its electronic structure^53,55,56^. Ultraviolet-visible spectrum displayed that both GDY and NGDY have good absorption in the visible light range (Fig. 2b). The corresponding Tauc curves showed that the band gap of GDY was 1.72 eV, while the band gap of NGDY was 1.87 eV (Fig. 2c), indicating that the introduction of N element effectively widens the band gap. The ultraviolet photoelectron spectroscopy (UPS) exhibited that the cutoff edges of GDY and NGDY are 18.08 eV and 17.55 eV (Fig. 2d), respectively, corresponding to work functions of 3.14 eV and 3.67 eV, respectively. XPS valence band spectra indicated that the valence band maximum values of GDY and NGDY are 0.87 eV and 1.06 eV relative to the Fermi level, respectively (Fig. 2e). Furthermore, the band structure of NGDY was studied through density functional theory (DFT) calculations. As shown in Fig. 2f, NGDY exhibited a natural bandgap of 2.35 eV. The bandgap of GDY was calculated to be 0.45 eV (Supplementary Fig. 11), which is consistent with the reported results^57^. In order to get closer to the real situation, a 9-layer stacked model was used to calculate the band gap of NGDY, and the result was 1.94 eV (Supplementary Fig. 12). The projected density of states (PDOS) results showed that the energy band of NGDY is mainly composed of C-*p* and N-*p* orbitals (Fig. 2g), and the introduction of N has an impact on the valence band maximum (VBM) and conduction band minimum (CBM) of GDY, highlighting nitrogen’s pivotal role in modifying the band structure (Fig. 2h), thereby tailoring the electronic properties for photocatalysis.

The photoelectrochemical performance of NGDY was carefully observed. The Mott-Schottky curve shows that the flat band potentials of GDY and NGDY are -1.42 V and -0.44 V, respectively (Fig. 2i). The positive shift for NGDY aligns with band structure measurements and suggests favorable band bending for photocatalysis. The positive slope in the Mott-Schottky curves indicated the n-type semiconductor characteristics of NGDY^58^. NGDY exhibited increased photocurrent density under light irradiation, reaching 130.5 μA cm^−^^2^ (Fig. 2j), indicating that the structure of NGDY is conducive to the separation and migration of photo-generated electrons and holes. The linear sweep voltammetry (LSV) curves demonstrated that the intrinsic OER activity of NGDY could be further enhanced under light irradiation (Supplementary Fig. 16 and 17), conclusively establishing the critical role of photoexcitation in boosting its catalytic performance.

### Charge transfer behavior of photo-excited *sp*-C

**Fig. 3:**
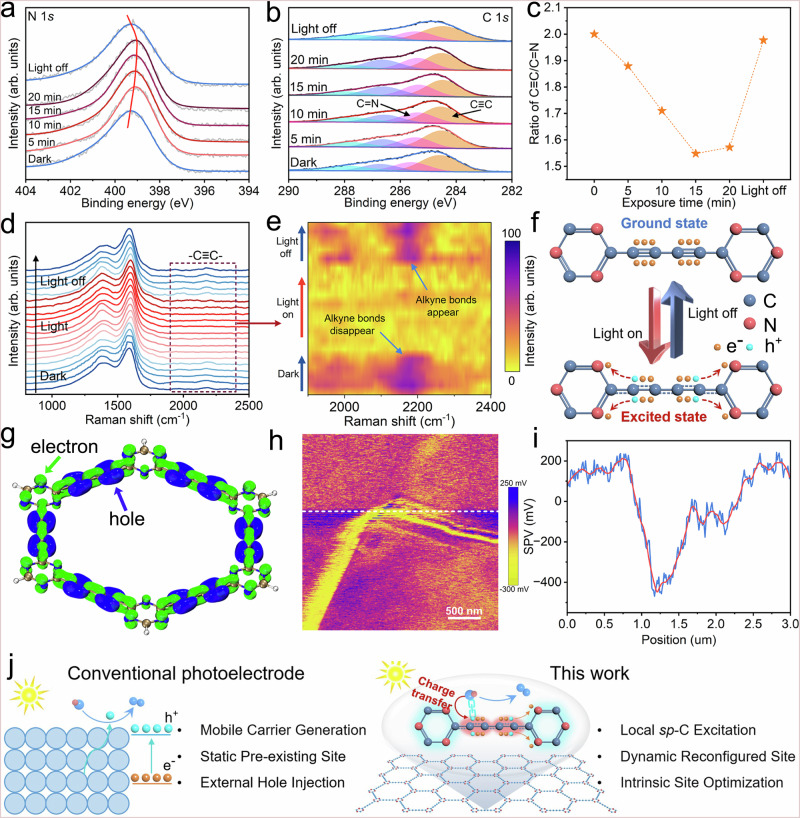
Charge transfer behavior of *sp*-C in NGDY under light excited. **a** XPS N 1 *s* spectra of NGDY in the dark and under light irradiation. **b** XPS C 1 *s* spectra of NGDY in the dark and under light irradiation. **c** The ratio changes of *sp*-C/*sp*^*2*^-C under light irradiation. **d** Raman spectra of NGDY in the dark and under light irradiation. **e** The 2D color-filled contour plot of the Raman spectra magnified in the dashed box in (**d**). **f** Schematic illustration of the photo-generated electron-hole pairs separation process in NGDY. **g** The transition map of electrons and holes in NGDY under the light excitation. **h** SPV image of NGDY. **i** Line profiles of the SPV of NGDY. **j** Schematic illustration of a conventional photoelectrode and our proposed light-induced dynamic reconfiguration of *sp*-C. Source data are provided as a Source Data file.

To elucidate the charge transfer behavior of NGDY under photoexcitation, we conducted a series of characterizations (Fig. 3a, b). Firstly, we studied the changes in the XPS spectrum of NGDY under light irradiation. The N 1 *s* peak of NGDY appeared at 399.3 eV in the dark condition (Fig. 3a). After applying light irradiation, the N 1 *s* peak rapidly moved towards the low-energy region, with a peak position of 399.1 eV, and remained at this lower binding energy for 5 to 20 min under light irradiation (Fig. 3a). When the light was removed, the N 1 *s* peak fully reverted to 399.3 eV. This reversible, light-triggered negative shift provided direct spectroscopic evidence of electron accumulation on nitrogen atoms during photoexcitation, indicating intramolecular electron transfer. Concurrently, the C 1 *s* spectrum revealed that the initial ratio of *sp*-C to *sp*^*2*^-C was 2:1, and this ratio began to decrease after applying light irradiation (Fig. 3b, c). After 15 minutes of light exposure, the ratio of *sp*-C to *sp*^*2*^-C remained around 1.55. After removing the light, the ratio of *sp*-C to *sp*^*2*^-C quickly returned to its initial 2:1 (Fig. 3c). The reversible reduction in the *sp*-C component signifies that photoexcitation depletes electrons specifically from the alkyne bonds, confirming their role as electron donors. Furthermore, by examining the Raman spectra of NGDY under light irradiation, we validated this charge transfer behavior. NGDY exhibits the characteristic peak related to the vibration of alkyne bonds at 2180 cm^-1^ in the dark condition (Fig. 3d, e)^59^. After applying light irradiation, the characteristic peaks of alkyne bonds disappear, while after removing light, the two characteristic peaks of alkyne bonds gradually appear again. These results indicate that under irradiation, the alkyne bond in NGDY is excited, and the charge on the alkyne bond is partially transferred to the N atom (Fig. 3f). This collective evidence clearly establishes the direct experimental observation of intramolecular charge transfer from photoexcited *sp*-C to N acceptors within the NGDY framework. DFT simulations of electron-hole transition distributions provided theoretical validation, showing hole localization around the alkyne bonds and electron accumulation on adjacent single bonds and nitrogen atoms upon excitation, further corroborating the light-induced electron redistribution from *sp*-C to N sites (Fig. 3g).

The separation behavior of photo-generated carriers in NGDY under light excitation was directly observed using a Kelvin probe atomic microscope. The surface photovoltage (SPV) image demonstrated that NGDY has an ordered stacked nanosheet morphology and revealed the potential distribution of NGDY nanosheets under light irradiation (Fig. 3h). The SPV profile recorded a remarkably high surface potential difference of ~450 mV upon light exposure (Fig. 3i), demonstrating NGDY’s high efficiency in separating photo-generated charge carriers. This SPV magnitude directly correlates with the efficient intramolecular charge transfer observed spectroscopically, highlighting its contribution to macroscopic carrier separation. In a word, the fundamental distinction of our system lies in the object of photoexcitation. In conventional photoelectrodes, light generates charge carriers that migrate to static, pre-existing active sites without altering their intrinsic electronic structure. In contrast, in NGDY, the *sp*-C moieties themselves are the active sites; photoexcitation directly and reversibly reconfigures their electronic structure, thereby dynamically optimizing their intrinsic catalytic properties in real time (Fig. 3j).

### The performance of NGDY-based photo-coupled MABs

**Fig. 4:**
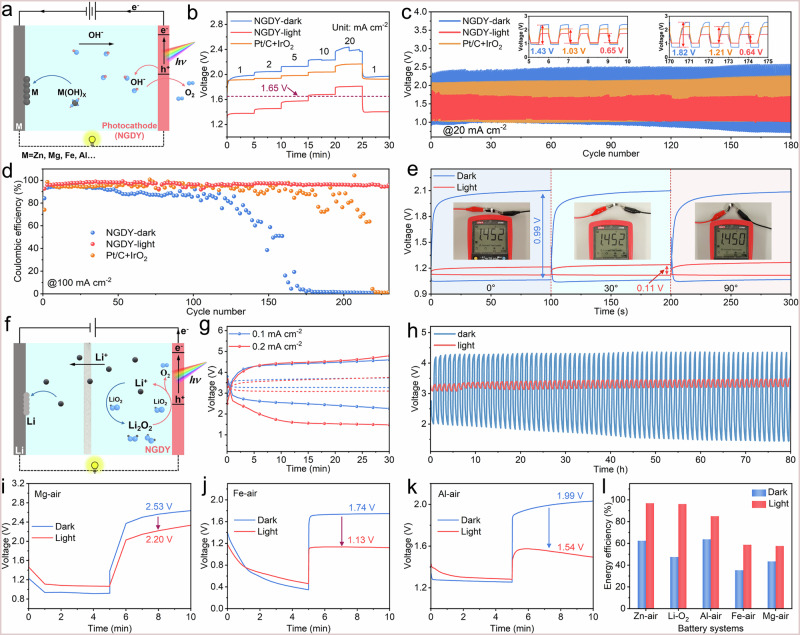
Performance of photo-coupled air electrode based on NGDY. **a** Structure of the PZAB. **b** Charge curves at current densities from 1 mA cm^−2^ to 20 mA cm^-2^ based on NGDY and commercial Pt/C + IrO_2_. **c** Cycling performance of PZAB based on NGDY and commercial Pt/C + IrO_2_ at 20 mA cm^−2^. **d** Coulombic efficiency of the battery over long-term cycling at 100 mA cm^−2^. **e** Discharge and charge profiles of the solid PZAB with and without light irradiation under different bending conditions (0°, 30° and 90°) at 1 mA cm^−2^. **f** Structure and the proposed operating mechanism of PLOB. **g** Discharge and charge profiles of the PLOB with (dash line) and without (solid line) light irradiation. **h** Cycling performance of PLOB based on NGDY at 0.01 mA cm^−2^. **i** Discharge and charge profiles of the photo-coupled Mg||air battery with and without light irradiation. **j** Discharge and charge profiles of the photo-coupled Fe||air battery with and without light irradiation. **k** Discharge and charge profiles of the photo-coupled Al||air battery with and without light irradiation (Details of the test can be found in the Methods section.). **l** Energy efficiency of NGDY-based MABs in the dark and light irradiation. All electrochemical measurements were performed at 25 ± 0.5 °C. Source data are provided as a Source Data file.

To assess the photocatalytic activity and electrochemical stability of NGDY as a highly efficient photo-coupled air electrode in practical application scenarios, the as-prepared NGDY was fabricated into a PZAB for comprehensive electrochemical evaluation. The battery configuration is illustrated in Fig. 4a, featuring a zinc negative electrode, electrolyte (6 M KOH and 0.2 M zinc acetate), and the NGDY-based photo-coupled air positive electrode (Supplementary Fig. 24). The operational mechanism is illustrated in Fig. 4a and Supplementary Fig. 25. Under photoexcitation, the electronic structure of NGDY is dynamically reconfigured, generating highly active *sp*-C sites with intrinsically enhanced catalytic activity. Photo-generated electrons are injected into the conduction band and transferred to the Zn electrode through an external circuit, facilitating the deposition of zinc. Simultaneously, the photo-excited catalytic surface of NGDY, enriched with photo-generated holes and optimized electronic states, dramatically facilitates the oxidation of OH^-^ to O_2_. This process effectively compensates for the elevated charging voltage typically required during battery charging. Moreover, the photothermal effects at the electrode/electrolyte interface would lower oxygen reaction barriers and accelerate charge/mass transfer (Supplementary Fig. 66)^60,61^, yet this effect has been minimized through infrared filtering and active cooling. The predominant performance enhancement arises from the intrinsic photoelectrochemical response of NGDY. As shown in the LSV curves (Supplementary Fig. 26), the PZAB based on NGDY exhibits reduced polarization potential under light irradiation, even lower than the commercial Pt/C and IrO_2_ electrode. These findings demonstrate that NGDY actively participates in the battery reactions, enabling the conversion of light energy into chemical energy that is stored during charging, thereby enhancing the reaction kinetics.

The NGDY-based PZAB delivered a specific capacity of 792.84 mA h g_Zn_^−1^ and a specific energy of 880.05 mW h g_Zn_^−1^ based on the consumption of Zn (Supplementary Fig. 27). Galvanostatic charging curves revealed excellent rate capability at various current densities across 1 to 20 mA cm^−2^ (Fig. 4b). At a current density of 1 mA cm^−2^, the charging voltage decreased from 1.96 V in the dark to 1.33 V under light irradiation, lower than the theoretical voltage of zinc||air batteries (1.65 V) and also lower than 1.91 V based on commercial Pt/C + IrO_2_ (Fig. 4b), while the energy efficiency increased from 61.7% to 96.9% (Supplementary Fig. 28 and Supplementary Table 3). Even at a high current density of 20 mA cm^−2^, the charging voltage of the battery was only 1.80 V, highlighting the potential of facilitating reaction kinetics under light to achieve high current density charging of the battery for fast-charging technology. Remarkably, at a high current density of 20 mA cm^−2^, the PZAB employing the NGDY photo-coupled electrode exhibited no voltage decay over 180 cycles, with the voltage gap changing only from an initial 0.65 V to 0.64 V (Fig. 4c). In contrast, under dark conditions, the voltage gap increased from 1.43 V to 1.82 V during charge-discharge cycling. Similarly, the voltage gap of the zinc||air battery utilizing a commercial Pt/C + IrO_2_ electrode increased from 1.03 V to 1.21 V after cycling. All those demonstrated the long-term stability of NGDY as a photoelectrode (Fig. 4c).

To evaluate its application prospects in fast-charging zinc||air batteries, we further investigated the cycling performance of NGDY-based PZAB at a high current density of 100 mA cm^−2^. Polished copper foil was employed as the current collector instead of zinc electrodes to enable a more accurate evaluation of energy conversion efficiency and discharge products. As shown in Fig. 4d, under light irradiation, the PZAB exhibited continuous and stable cycling over 230 cycles while maintaining a coulombic efficiency above 96%. In contrast, the coulombic efficiency of the battery cycled in the dark began to decline rapidly after 100 cycles. Similarly, the battery using commercial Pt/C+IrO₂ also showed a rapid decrease in coulombic efficiency after 170 cycles, accompanied by a sharp increase in charging voltage (Supplementary Fig. 31). The chemical structure and morphological stability of NGDY after the cycle were confirmed by SEM, TEM, and XPS (Supplementary Fig. 32 to 35). Post-cycling SEM characterization was performed after removing the current collector to examine the zinc deposition morphology. Extensive formation of zinc dendrites was observed after high-current charging in the absence of light (Supplementary Fig. 36). These dendritic structures are highly prone to detach from the electrode, which can result in irreversible capacity loss and increase the risk of internal short circuits and potential battery failure. In contrast, under light irradiation, the deposited zinc exhibited a uniform and flat morphology without large prominent particles (Supplementary Fig. 37). This dense and homogeneous deposition effectively helps prolong the battery cycle life and mitigates safety hazards associated with dendrite formation during fast-charging conditions (Supplementary Fig. 38 to 39). The enhanced stability originates from the milder operating conditions brought about by photo-excitation. Specifically, the lower charging overpotential reduces the degradation of the high-voltage driving electrode/electrolyte in the positive electrode, while promoting a uniform, dendrite-free zinc coating on the negative electrode.

In addition to progress in aqueous PZABs, we have further expanded our strategy to construct solid-state PZABs and other photo-coupled RMABs using the NGDY photoelectrode. These results demonstrate not only the broad applicability of our design strategy but also the remarkable versatility and adaptability of NGDY electrodes across diverse energy storage systems. The solid-state PZAB employing NGDY as the photoelectrode demonstrated an open-circuit voltage of 1.46 V (Supplementary Fig. 41). It exhibited stable cycling performance across a range of current densities from 0.1 to 1 mA cm^−2^ (Supplementary Fig. 42), along with strong mechanical robustness, maintaining consistent power output under repeated bending and folding conditions (Fig. 4e and Supplementary Fig. 43), underscoring its applicability in wearable and flexible electronics. Under light irradiation, the polarization curve showed reduced overpotential (Supplementary Fig. 46). Furthermore, during intermittent light exposure, the charging voltage progressively decreased (Supplementary Fig. 47), demonstrating enhanced operational stability under photo-assisted conditions. Notably, at a current density of 1 mA cm^−2^, the charging voltage dropped substantially from 2.05 V in the dark to 1.20 V under light, while the energy efficiency increased from 51.6% to 93.6% (Fig. 4e). These results clearly indicate that the photoexcitation of NGDY within the battery effectively facilitates electrode reaction kinetics and promotes efficient solar energy conversion and utilization.

Lithium||oxygen batteries are similarly hindered by pronounced voltage hysteresis and low energy efficiency, primarily due to sluggish reaction kinetics^62^. Additionally, the inevitable accumulation of incompletely decomposed Li_2_O_2_ and parasitic products at the positive electrode leads to pore clogging and rapid capacity fade^63^. The integration of a photo-coupled electrode offers a direct solution to these fundamental challenges. As illustrated in Fig. 4f and Supplementary Fig. 52, upon light exposure, the NGDY electrode undergoes electronic restructuring that creates transient, highly oxidative centers alongside photo-generated holes, which directly contribute to the efficient decomposition of Li_2_O_2_, while the photoelectrons are transported through the external circuit to the lithium electrode, facilitating the reduction of Li ions to lithium metal. This synergy between light-driven catalytic activation and carrier-mediated redox processes enables highly efficient operation of the PLOB. The CV plot of the NGDY electrode under light irradiation displays a more positive ORR onset potential and a more negative OER potential, with a larger integral area compared to the plot in the dark (Supplementary Fig. 53). The two OER peaks of the plot under light irradiation are attributed to the deintercalation process of Li_2_O_2_ and the oxidation process of Li_2_O_2_, respectively^64^. At a current density of 0.1 mA cm^-2^, the voltage gap decreased from 1.81 V in the dark to 0.39 V under light irradiation (Fig. 4g). Similarly, at 0.2 mA cm^−2^, the voltage gap decreased from 2.62 V to 0.52 V (Fig. 4g). The PLOB equipped with an NGDY photoelectrode demonstrated cycling stability over 80 h, maintaining a consistently narrow voltage gap throughout both discharge and charge processes, and achieving a high energy efficiency of 96.3%. This performance outperformed the device operated in the dark, which exhibited a progressively widening voltage gap over time (Fig. 4h). Those results highlight the critical role of photoexcited charge carriers in facilitating electrochemical reactions and improving the long-term cycling durability of PLOB. Electrochemical impedance spectroscopy (EIS) analysis indicated a reduction in charge transfer resistance (Rct) under light irradiation, as confirmed by the diminished semicircular arc in the high-frequency region. The Rct value continued to decrease with increasing light intensity (Supplementary Fig. 55), providing strong evidence for photo-accelerated interfacial charge transfer kinetics. This enhancement promotes efficient electron transport to redox-active sites, thereby substantiating the observed improvements in battery performance. Furthermore, control experiments confirmed that the photoresponse originates from the intrinsic photoelectric conversion of the material, rather than being dominated by the thermal effect induced by illumination (Supplementary Figs. 64–67).

Similarly, the polarization curve of the Mg||air battery based on NGDY was improved under light irradiation (Supplementary Figs. 68 and 69), with a notable reduction in the charging voltage across current densities of 0.1 to 1 mA cm^-2^ (Supplementary Figs. 70 and 71). The charging voltage decreased from 2.53 V to 2.20 V and the energy efficiency increased from 43.4% to 57.6% (Fig. 4i). Given the traditionally high voltage polarization and limited cycling stability of Mg||air batteries^65,66^, the integration of photo-coupled air electrodes offers a promising strategy to mitigate excessive charging voltages, thereby advancing the development of rechargeable Mg||air batteries with enhanced cycling performance for practical applications. Figure 4j illustrates the photo-coupled charging capability of the Fe||air battery (Supplementary Fig. 72 to 74), where the battery exhibited a decreased charging potential of 1.13 V and an increased energy efficiency of 58.7% under light irradiation. The Al||air battery based on NGDY exhibited a low charging voltage of 1.54 V and a high energy efficiency of 85.0% under light irradiation (Fig. 4k and Supplementary Figs. 76, 77). Those results further validate that the utilization of solar energy to accelerate the oxygen evolution reaction kinetics can be generalized to other MAB systems to address the challenge of charging overpotential (Fig. 4l).

### In-situ characterization and theoretical study of photo-coupled MABs

**Fig. 5:**
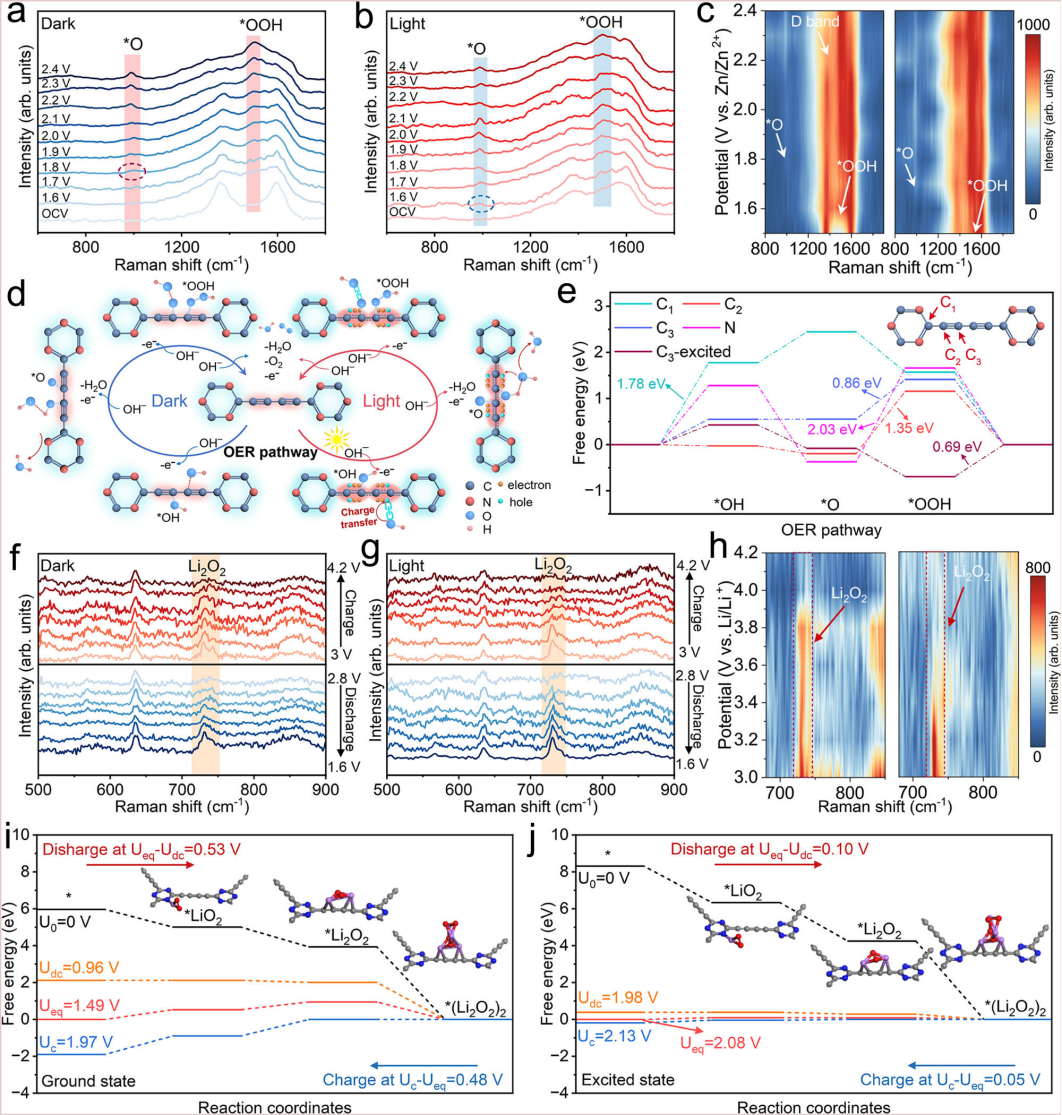
In-situ characterization of NGDY-based photo-coupled air electrode. **a** In-situ Raman spectra of NGDY-based PZAB during the charging process in the dark. **b** In-situ Raman spectra of NGDY-based PZAB during the charging process under light irradiation, and **c** the corresponding 2D color-filled contour plot of the in-situ Raman spectra (Left: in the dark. Right: under light irradiation. Recorded during a LSV scan of the battery charging voltage from 1.6 V to 2.4 V at a scan rate of 0.1 mV s^−1^, with spectra acquired at every 0.1 V interval). **d** Schematic illustration of OER pathways on the NGDY in the dark and under light irradiation. **e** Free energy diagrams of different sites for OER. **f** In-situ Raman spectra of NGDY-based PLOB in the dark. **g** In-situ Raman spectra of NGDY-based PLOB under light irradiation. The spectra were collected over a full cycle: discharging from 2.8 V to 1.6 V and then charging from 3.0 V to 4.2 V at a scan rate of 0.1 mV s^−1^, with spectra recorded at 0.2 V intervals. **h** The corresponding 2D color-filled contour plot of the in-situ Raman spectra of NGDY-based PLOB during the charging process (Left: in the dark. Right: under light irradiation). Free energy diagrams at zero, equilibrium, discharge and charge voltages of NGDY for PLOB at **i** ground state and **j** excited state. All in-situ measurements were performed at 25 ± 0.5 °C. Source data are provided as a Source Data file.

In-situ Raman spectroscopy was employed to examine the intermediate species formed during the reaction process in NGDY-based photo-coupled MABs. In the dark, the emergence of the *OOH intermediate peak at 1504 cm^−1^ was observed when the charging voltage reached 1.7 V (Fig. 5a). Subsequently, at 1.8 V, a peak corresponding to the *O intermediate appeared at 990 cm^−1^. With further increases in charging voltage, the intensities of both *OOH and *O peaks progressively intensified. In contrast, under light irradiation, the *OOH peak was detected at a lower charging voltage of 1.6 V, and the *O peak emerged at 1.7 V (Fig. 5b, c). The earlier appearance of these key intermediates under light exposure indicates a reduction in the reaction barrier for the battery charging process. Interestingly, during charging in the dark, the intensities of the *O and *OOH peaks continued to increase with voltage, becoming particularly pronounced at 2.4 V, where the *OOH peak even obscured the NGDY G-band peak. In contrast, under light irradiation, the intensities of these intermediate peaks exhibited little change beyond 2.1 V. This phenomenon can be attributed to the photoexcitation of NGDY, which enhances its catalytic activity, thereby facilitating the rapid reaction of the *O and *OOH intermediates and preventing their accumulation. Under dark conditions, however, the intermediates are unable to react promptly, leading to their progressive accumulation and increased peak intensities with rising voltage.

Based on this, we can conclude that the generation of photo-generated holes imparts a substantial driving force for the oxidation of the adsorbed intermediates, thereby synergistically accelerating the overall reaction kinetics (Fig. 5d). The adsorption energies of key OER intermediates (*OH, *O and *OOH) on NGDY were calculated for both the ground and excited states, with the results shown in Supplementary Fig. 78. In the ground state, the adsorption energies for *OH, *O, and *OOH were determined to be −2.93 eV, −5.15 eV, and −1.01 eV, respectively. Notably, upon photoexcitation, the adsorption energies for all three intermediates decreased to −3.77 eV, −5.50 eV, and −1.45 eV. This consistent reduction in adsorption energies across all intermediates suggests that the photoexcited NGDY surface exhibits an enhanced affinity for capturing these species. The Gibbs free energy of OER at different sites in NGDY indicates that the C_3_ site is the active site for catalyzing OER (Fig. 5e). In the excited state, due to the generation of photo-generated holes at the C_3_ site under photoexcitation, the free energy for catalyzing OER is further reduced. Consequently, the synergistic effect between improved intermediate adsorption and hole-mediated oxidation facilitates the OER process under light irradiation.

Furthermore, we conducted operando Raman spectroscopy on a PLOB to gain deeper insight into the operational mechanism of NGDY-based MABs. Throughout charge-discharge cycling, the characteristic peak at 730 cm^−^^1^ is assigned to the O-O stretch of Li_2_O_2_, exhibited regular intensity variations, while peaks at 635 cm^−1^ and 850 cm^−^^1^ were attributed to the electrolyte LiTFSI in TEGDME (Fig. 5f, g)^67^. During discharge, the Li_2_O_2_ peak gradually intensified as the voltage decreased (Fig. 5f and Supplementary Fig. 79). Upon charging, the Li_2_O_2_ peak continuously decreased. Strikingly, when charging under light irradiation, the Li_2_O_2_ peak almost completely vanished by 4.0 V (Fig. 5g, h). In contrast, during charging in the dark, the peak for Li_2_O_2_ still persisted at 4.2 V (Figs. 5f, 5h). This spectral difference offers definitive mechanistic insight and direct spectroscopic evidence that photoexcitation of NGDY drastically promotes the reaction kinetics in Li||O_2_ chemistry, enabling the rapid and complete consumption of intermediates that would otherwise accumulate and degrade performance in the dark due to sluggish kinetics.

The reaction free energy diagrams for the nucleation and decomposition pathways of (Li_2_O_2_)_2_ clusters on the NGDY surface under both ground and photoexcited states are shown in Fig. 5i and 5j, respectively. The discharge overpotential is defined as U_eq_ – U_dc_, and the charge overpotential as U_c_ – U_eq_, where U_eq_ represents the equilibrium voltage at which the entire Gibbs free energy change is zero. Here, U_dc_ refers to the highest potential allowing a continuous decrease in Gibbs free energy during discharge, and U_c_ denotes the lowest potential driving the free energy increase during charging. The discharge overpotential is reduced from 0.53 V in the ground state to 0.10 V under photoexcitation, while the charge overpotential drops from 0.48 V to 0.05 V. The reduced energy barriers under light irradiation directly contribute to the remarkably low charge overpotential, indicating that light excitation effectively minimizes the thermodynamic limitations of the oxygen electrochemistry. The adsorption energies and optimized configurations of reactants, intermediates, and products on NGDY in both electronic states are provided in Supplementary Figs. 81 and 82. The decreased adsorption energies under light excitation suggest stronger binding of Li–O_2_ intermediates, confirming enhanced interfacial stabilization. The strengthened adsorption of key intermediates further promotes efficient surface-mediated reaction kinetics, preventing undesirable solution-mediated pathways that often lead to capacity fading.

## Discussion

In summary, this work provides a research approach to address the critical kinetic limitations in MABs by pioneering a novel material strategy centered on the photoexcitation of *sp*-hybridized carbon in the NGDY electrode. We have demonstrated, through in-situ characterizations, that light irradiation triggers a targeted intramolecular charge redistribution within NGDY, which is the cornerstone of its superior functionality. This process not only generates a high density of active sites but also optimally modulates the adsorption of OER intermediates, thereby dramatically accelerating the reaction kinetics. The universality of this approach is validated by constructing high-performance photo-coupled Zn||air, Li||O_2_, Mg||air, and Fe||air batteries. The PZAB based on NGDY demonstrated low charging voltages below the thermodynamic equilibrium potential and the ability to enable safe, dendrite-free operation at a high current density of 100 mA cm^-2^, which underscores a performance that surpasses noble-metal and most reported systems and highlights the transformative potential of this technology for fast-charging applications. Moreover, the PLOB achieved high stability and an energy efficiency of 96.3%. Beyond presenting a superior catalyst, this study introduces a conceptual framework for photoelectrode design, moving beyond traditional semiconductor paradigms to exploit the photophysical properties of precisely engineered carbon networks. The principle of photoexcited molecular carbon structures will open broad avenues for the future development of sustainable, high-power, and cost-effective energy storage and conversion systems.

## Methods

### Materials and reagents

All the chemicals were purchased commercially and directly utilized. Trimethylsilylacetylene (≥ 98%), n-butyllithium (2.5 mol L^−1^ in hexane), cyanuric chloride (≥99.5%) and Pd(PPh_3_)_4_ (AR. Pd ≥ 9%) were purchased from Energy Chemical Co., Ltd. Tetrahydrofuran (THF, ≥ 99.5%), pyridine (≥99.5%), ethyl acetate (≥99.8%), acetone (≥ 99.8%) and Dimethyl sulfoxide (DMSO, ≥99.5%) were bought from Concord Technology Co. Ltd. Zinc acetate dihydrate [Zn(CH_3_COO)_2_·2H_2_O, ≥99.0%] was purchased from Macklin Biochemical Technology Co. Ltd. KOH, copper foil (0.1 mm in thickness, ≥99.9%) and Magnesium Trifluoromethanesulfonate [Mg(OTf)_2_, 99.3%] were bought from Sinopharm Chemical Reagent Co. Ltd. Zn foil (0.25 mm in thickness, ≥99.98%) was purchased from Alfa Aesar. Polyvinyl Alcohol (PVA, average MW 145,000) and Polyethylene oxide (PEO, average MW 100,000) were purchased from Aladdin Co. Carbon cloth (HCP331P) was provided by Shanghai Hesen Electrical Co., Ltd. The electrolyte for PLOB [1 M Lithium bis((trifluoromethyl)sulfonyl)azanide (LiTFSI) in Tetraethylene glycol dimethyl ether (TEGDME)] was obtained from Guangdong Canrd New Energy Technology Co., Ltd.

### Synthesis of precursor (2,4,6-(trimethylsilyl) ethynyl-1,3,5-triazine)

The precursor was synthesized according to the literature^2^. To prepare 2,4,6-(trimethylsilyl) ethynyl-1,3,5-triazine, under an argon atmosphere, 10 mL of trimethylsilylacetylene was introduced into a purified THF solution in a three-necked flask, which was then placed in an acetone bath cooled with liquid nitrogen. Subsequently, 30 mL of n-butyllithium was added dropwise over 30 min, followed by an additional 30 min of reaction. Then, 15 g of anhydrous zinc chloride was added, and the acetone bath was removed. After stirring at a temperature of 25 ± 0.5 °C for 2 h, 1 g of cyanuric chloride, 1 g of Pd(PPh_3_)_4_, and 40 mL of purified toluene were added sequentially. The reaction mixture was stirred overnight at 50 °C under argon. Upon cooling to 25 ± 0.5 °C, dilute hydrochloric acid was added, and the mixture was diluted with ethyl acetate and washed three times with saturated NaCl solution. The organic layer was dried over anhydrous MgSO_4_ and concentrated under reduced pressure. Finally, the residue was purified by column chromatography to obtain the pure 2,4,6-(trimethylsilyl) ethynyl-1,3,5-triazine. The precursor was subjected to NMR measurement to verify its purity.

### Synthesis of NGDY

The NGDY was synthesized through an optimized room-temperature method. Under Ar protection, 10 mg 2,4,6-(trimethylsilyl) ethynyl-1,3,5-triazine was dissolved in 20 mL dichloromethane in an ice bath. The whole system was kept away from light and stirred for 20 minutes. Then 1 mL TBAF was added and reacted for 10 min. The solution was then diluted with 20 mL ethyl acetate, washed with saturated NaCl three times, and the organic phase was dried twice with anhydrous MgSO_4_ and then filtered. The deprotected precursor in dichloromethane was combined with carbon cloth (2.5 × 4 cm^2^) pre-assembled between copper foils (3 × 5 cm^2^, washed with 1 M HCl, cleaned with acetone, and dried) within a sealed glass reactor, followed by dropwise addition of 1 mL pyridine. The reactor was maintained at 25 ± 0.5 °C for 24 h. The resulting material underwent sequential washing with DMF, acetone, 1 M HCl, and deionized water, followed by drying at 60 °C in a vacuum.

### Characterization

Scanning electron microscope (SEM) images and Energy dispersive X-ray spectroscopy (EDS) elemental mapping were obtained from Hitachi SU8020, 10.0 kV acceleration voltage. Transmission electron microscopy (TEM) and high-resolution TEM (HRTEM) images were recorded on a JEM-2100F field emission transmission electron microscope at an accelerated voltage of 200 kV. Raman spectra were measured on a Renishaw-2000 Raman spectrometer and a HORIBA microscopic confocal laser Raman spectrometer. The excitation light source of the Renishaw-2000 Raman spectrometer was 473 nm, and the excitation light source of the HORIBA microscopic confocal laser Raman spectrometer was 532 nm.

The XPS and UPS measurements were tested on a multifunctional photoelectron spectrometer (ESCALAB250XI). For the XPS spectra under light irradiation, the optical fiber was used to irradiate the sample surface and test the sample every five minutes. The light source for UPS was He I light source (excitation energy: 21.22 eV). The UV-vis-NIR diffuse reflection spectra were tested on a spectrophotometer (Lambda 1050 UV/VIS/NIR spectrometer, PerkinElmer), and the band gaps were calculated from the corresponding Tauc plot based on the following equation,

1 $${{{\rm{Eg}}}}={{{\rm{h}}}}{{{\rm{v}}}}={{{\rm{hc}}}}/\lambda$$

where Eg is the band gap, h is Planck’s constant (h = 4.1356676969 × 10^−15 ^eV·s), c is the speed of light (c = 299792458 m s^−1^), and *λ* is the wavelength of the light.

KPFM images and surface photovoltage (SPV) was measured using the KPFM module on a large range high speed atomic force microscopy (Dimension FastscanBio). Steady-state PL spectra and PL decay spectra were measured on the FLS980 fluorescence spectrometer. For steady-state PL spectra, the excitation wavelength was 390 nm. A 390 nm monochromatic filter was placed between the light source and the sample, and a 410 nm cutoff filter was placed between the sample and the detector. The wavelength range for detection was 450 nm–850 nm. PL decay spectra were measured by time-correlated single-photon counting with an excitation wavelength of 405 nm, and the decay curves were fitted to obtain the average fluorescence lifetimes (*τ*_*ave*_):

2 $${\tau }_{{ave}}=\,\frac{\sum {B}_{i}{\tau }_{i}^{2}}{\sum {B}_{i}{\tau }_{i}}$$

### Theoretical calculations

DFT calculations were conducted using the plane-wave-based method with spin-polarization implemented in the Vienna Ab initio Simulation Package (VASP). The Blöchl’s all-electron-like projector augmented wave (PAW) pseudo-potential was applied to consider the core-valence electron interactions. The Perdew-Burke-Ernzerhof functional of generalized gradient approximation (GGA-PBE) was adopted to describe the exchange-correlation interactions. For the geometry optimization calculations, the energy and force convergence criteria were set to 1.0 × 10^−5^ eV/atom and 0.01 eV/Å, respectively. A plane-wave cutoff energy of 400 eV and the Γ-centered k-point meshes sampled in the Monkhost-Pack grid were employed. Long-range van der Waals (vdW) interaction was calculated using the Grimmer’s DFT-D3 method. To avoid the periodic interactions between slabs, a vacuum layer as large as 20 Å was used along the c direction. The free energy in the excited state was performed approximatively using the constrained DFT calculations, by promoting one electron from the highest occupied molecular orbitals to the lowest unoccupied molecular orbitals at each k point (Supplementary Data 1, 2).

The adsorption energy $\left({E}_{{\mbox{ads}}}\right)$ of Li_m_O_n_ and ORR/OER intermediates on the electrode surface (Supplementary Data 3–8) was calculated using the following equation:

3 $${E}_{{ads}}={E}_{{X}^{*}}-{E}_{*}-{E}_{X}$$

where ${E}_{{X}^{*}},{E}_{*},$ and ${E}_{X}$ are the DFT-calculated total energies of the electrode surface with adsorbed Li_m_O_n_ or ORR/OER intermediates, the bare electrode surface, and isolated Li_m_O_n_ or ORR/OER intermediates, respectively.

To calculate the reaction free energies, a harmonic approximation was applied to adsorbates and gaseous species to make corrections for the DFT-calculated total energy, which was described as follows:

4 $$G\left(X\right)={E}_{X}+{{ZPE}}_{X}-T{S}_{X}^{{vib}}$$

where the vibrational zero-point energy corrections (ZPE) and vibrational entropy corrections (TΔS,298 K adopted in this work) were added.

The hole-electron analysis was used to further characterize the excitation between the ground state and excited state of NGDY, which was carried out using the Multiwfn program and was displayed using the VMD program, respectively. The ground state and excited state structures of the NGDY fragment were optimized using DFT and TDDFT methods, respectively, which were performed using the Gaussian 16 program (Revision B01). The popular hybrid density functional B3LYP, adding the D3 version of Grimme’s dispersion with Becke-Johnson damping function, was used. The Pople series triple-z basis set, 6-311 G(d) was used for all atoms.

### Photo/electrochemical performance

The photoelectrochemical performance was measured using a quartz electrolytic cell with a typical three-electrode setup on an electrochemical workstation (CHI 660E, Shanghai CH. Instruments, China) in a photoelectrochemical chamber (25 ± 0.5 °C). A saturated calomel electrode (SCE) and a graphite rod were adopted as the reference electrode and counter electrode, respectively. The carbon cloth grown with NGDYs was used directly as the working electrode. Mott–Schottky (M-S) plots were tested in 1 M Na_2_SO_4_ at 1000 Hz. The photocurrent was tested in 1 M KOH without applied potential and with intermittent light irradiation. A 300 W xenon lamp (PLS-SXE 300, Perfect Light, Beijing) was used as the light source. An 800 nm infrared filter was employed to tailor the incident spectrum to the ~320–800 nm range. The light intensity at the sample position was calibrated to 500 mW cm^−2^ using a photo radiometer. LSV curves were measured in a 1 M KOH solution at a scan rate of 10 mV s^−1^ and with 100% iR compensation. The potentials applied for electrochemical measurements were converted to RHE by using the following equation,

5 $${E}_{{RHE}}={E}_{{SCE}}+0.059{{{\rm{pH}}}}+{{{{{\rm{E}}}}}^{0}}_{{{{\rm{SCE}}}}}$$

where *E*_*SCE*_ is the measured potential, E^0^_SCE_ is 0.242 V.

### Fabrication and measurements of photo-coupled MABs

#### Aqueous PZAB

The device for fabricating PZAB (Supplementary Fig. 24) was obtained from Changsha Spring New Energy Technology Co., Ltd. A polished Zinc foil (3 × 5 cm^2^, polished with sandpaper before use, cleaned with ethanol, and dried) was used as the negative electrode, and NGDY/CC (1 × 1 cm^2^) was directly used as the positive electrode. The electrolyte was a mixed solution of 6 M KOH and 0.2 M Zn(CH_3_COO)_2_ (250 mL). The time of each charge-discharge cycle was 10 min (5 min discharge followed by 5 min charge). All the measurements were carried out using a CHI 660E electrochemical workstation and a LAND CT3002AU battery test system in a photoelectrochemical chamber (25 ± 0.5 °C). The performance of PZAB under light irradiation was measured under the same conditions except for the addition of a light source on the positive electrode. A 300 W xenon lamp (PLS-SXE 300, Perfectlight, China) served as the full-spectrum light source. For all photo-coupled battery tests, an 800 nm infrared filter (to attenuate infrared heating) was employed to tailor the incident spectrum to the ~320–800 nm range. The output spectrum of the xenon light source was provided in Supplementary Fig. 64. The light intensity at the sample position was calibrated to 500 mW cm^−2^ (Supplementary Fig. 24) using a photo radiometer (CEL-NP2000, Ceaulight). For comparison, the commercial Pt/C and IrO_2_ catalysts (5 mg) were dispersed in 950 uL of ethanol, and then 50 uL of Nafion solution (5 wt%) was added. After the dispersion solution is ultrasonically dispersed, it is evenly dripped onto the carbon cloth electrode (loading: 1 mg cm^−2^). After the test was completed, the positive and negative electrodes were carefully taken out, washed with deionized water for three times, and then dried under vacuum at 60 °C. The dried samples were protected in an Ar atmosphere at 25 ± 0.5 °C and were immediately subjected to ex-situ measurements.

To accurately assess the long-term operational stability, zinc deposition morphology evolution, and, crucially, the Coulombic efficiency (CE) of the Zn||air batteries at a high current density of 100 mA cm^−2^, a specific testing protocol employing a copper foil as the negative electrode current collector was adopted. This method replaces the conventional zinc foil and allows for the direct evaluation of zinc plating/stripping reversibility per cycle. The procedure is as follows: a polished copper foil was assembled as the working electrode (negative electrode). In each cycle, the battery was first charged at a constant current density of 100 mA cm^−2^ for 60 s to deposit zinc onto the copper substrate. Subsequently, it was immediately discharged at the same current density until the deposited zinc was completely stripped, indicated by the cell voltage dropping to approximately 0 V, and the discharge time was recorded. The CE for each individual cycle was then calculated using the formula:

6 $${CE}=\,\frac{{Q}_{{discharge}}}{{Q}_{{charge}}}\times 100\%=\frac{{t}_{{discharge}}}{{t}_{{charge}}}\times 100\%$$

The battery energy efficiency (*η*_*battery*_) was calculated by the following formula:

7 $${\eta }_{{battery}}=\frac{{E}_{{discharge}}}{{E}_{{charge}}}\times 100\%$$

Where *E*_*discharge*_ refers to the electric energy released when discharging, and *E*_*charge*_ stands for the energy required to charge. The calculation of power conversion efficiency (PCE) of the PZABs was as follows:

8 $${PCE}=\frac{{E}_{{dark}}-{E}_{{light}}}{{P}_{{in}}}\times 100\%$$

Where *P*_*in*_ is the light intensity.

#### Solid PZAB

Firstly, the polymer gel electrolyte was prepared: 5 g PVA and 0.5 g PEO were dissolved in 50 mL deionized water under magnetic stirring. The solution was then heated to 95 °C for 2 h under stirring. Next, 5 mL 18 M KOH solution was dropwise added, and the solution was kept stirring at 95 °C for another 40 minutes. After the solution cooled to 25 ± 0.5 °C, it was poured onto a plate to form a thin film, followed by freezing at −30 °C for 1 h and then thawed at temperature of 25 ± 0.5 °C. Finally, a piece of N-GDY/CC (0.5 × 1 cm^2^) and the polished zinc foil (0.5 × 1 cm^2^) were placed tightly on the two sides of gel electrolytes (0.5 × 1 cm^2^) without any additives. The time of each charge-discharge cycle was 200 s. All the measurements were performed by a CHI 760E electrochemical workstation in a photoelectrochemical chamber (25 ± 0.5 °C).

#### PLOB

The PLOB was assembled in an Ar-filled glovebox with both H_2_O and O_2_ contents below 0.1 ppm. The battery was assembled in a coin-type (CR2032) cell. The cap in contact with the air electrode of the cell was punched to ensure the entrance of oxygen and light. The Li foil (diameter: 1.25 cm, thickness: 0.55 mm, purity: ≥99.9%) is stored in an Ar-filled glovebox (O_2_ < 0.1 ppm, H_2_O < 0.1 ppm) and is directly taken out when used. The electrolyte is a solution of 1 M LiTFSI in TEGDME (150 μL), and the separator was a Waterman glass fiber membrane. The NGDY/CC electrodes were cut into circular pieces with a diameter of 0.6 cm. After purging with O_2_ in a homemade glass cell for 30 min, galvanostatic discharge/charge cycles were conducted on a LAND CT3002AU battery test system (Supplementary Fig. 51) in a photoelectrochemical chamber (25 ± 0.5 °C). For the photo-coupled battery tests, an 800 nm infrared filter was employed to tailor the incident spectrum to the ~320–800 nm range. The time of each charge-discharge cycle was 60 min (30 min discharge followed by 30 min charge). After the test was completed, the battery was disassembled in the glove box (O_2_ < 0.1 ppm, H_2_O < 0.1 ppm, 25 ± 0.5 °C). Carefully took out the positive and negative electrodes, ant the positive electrodes were immersed in TEGDME for 12 h and dried in a glove box for 48 h. The electrodes were protected with Ar, and ex-situ measurements were immediately performed.

#### Photo-coupled Mg||air battery

The photo-coupled Mg||air battery was assembled in a coin-type (CR2032) cell. The cap in contact with the air electrode of the cell was punched to ensure the entrance of light. A polished Mg foil (diameter: 1.5 cm, thickness: 0.15 mm, purity: ≥ 99.9%, polished with sandpaper before use, cleaned with ethanol, and dried) was utilized as the negative electrode, and NGDY/CC (cut into circular pieces with a diameter of 1.5 cm) was used as the positive electrode. The electrolyte comprised a 1 M Mg(OTf)_2_ solution in DMSO, and the separator was a Waterman glass fiber membrane. The discharge and charge profiles were measured at a current density of 0.1 mA cm^-2^ in a photoelectrochemical chamber (25 ± 0.5 °C). The time of each charge-discharge cycle was 10 min (5 min discharge followed by 5 min charge).

#### Photo-coupled Fe||air battery

The device for fabricating a photo-coupled Fe||air battery was the same as the aqueous PZAB. The negative electrode was a polished Fe foil (3 × 5 cm^2^, thickness: 0.1 mm, purity: ≥ 99.9%, polished with sandpaper before use, cleaned with ethanol, and dried) and the electrolyte was 6 M KOH (250 mL). The NGDY/CC electrodes were cut into 1 × 1 cm^2^ square shapes. The battery was discharged at a current density of 0.1 mA cm^−2^ and charged at a current density of 1 mA cm^−2^ in a photoelectrochemical chamber (25 ± 0.5 °C). The time of each charge-discharge cycle was 10 min (5 min discharge followed by 5 min charge).

#### Photo-coupled Al||air battery

The device for fabricating photo-coupled Al||air battery was the same with the aqueous PZAB. The negative electrode was a polished Al foil (3 × 5 cm^2^, thickness: 0.1 mm, purity: ≥99.9%, polished with sandpaper before use, cleaned with ethanol, and dried) and the electrolyte was 1 M KOH (250 mL). The NGDY/CC electrodes were cut into 1 × 1 cm^2^ square shapes. The discharge and charge profiles were measured at a current density of 1 mA cm^−2^ in a photoelectrochemical chamber (25 ± 0.5 °C). The time of each charge-discharge cycle was 10 min (5 min discharge followed by 5 min charge).

### In-situ Raman characterization

In-situ Raman characterization was conducted using a customized electrochemical cell equipped with a quartz optical window to allow laser access and, where applicable, simultaneous light irradiation. A Horiba confocal Raman microscope with a 532 nm laser was used for all measurements. For experiments under photoexcitation, light from a 300 W xenon lamp (CME-X305, Microenerg Beijing Technology Co., Ltd) was focused onto the working electrode surface through an optical fiber. All in-situ Raman measurements were performed at 25 ± 0.5 °C.

For the Zn||air battery measurements, the negative electrode consisted of a polished zinc foil, and the positive electrode was the NGDY grown on carbon cloth (NGDY/CC). The electrodes were separated by a glass fiber membrane (Whatman), and 150 µL of electrolyte (6 M KOH + 0.2 M zinc acetate) was added. The cell was connected to a CHI 660E electrochemical workstation. Raman spectra were collected by performing an LSV scan on the battery charging voltage from 1.6 V to 2.4 V, with a scan rate of 0.1 mV s^−1^ and spectrum acquired at every 0.1 V interval.

For the Li||O_2_ battery measurements, the cell comprised a lithium foil negative electrode, an NGDY/CC positive electrode, and a glass fiber separator soaked with 150 µL of electrolyte (1 M LiTFSI in TEGDME). The positive electrode was sealed against a quartz window, and the cell body was connected to an O_2_ gas bag. The electrochemical protocol involved a full cycle: first, discharging from 2.8 V to 1.6 V, followed by charging from 3.0 V to 4.2 V with a scan rate of 0.1 mV s^−1^. Raman spectra were collected at 0.2 V intervals throughout both the discharge and charge processes.

## Supplementary information

Supplementary information

Description of Additional Supplementary Files

Supplementary Data 1-8

Transparent Peer Review file

## Source data

Source Data

## Data Availability

The data supporting the findings of this study are available in the main text or its Supplementary Information files. Source data are provided with this paper.

## References

[1] Liu, X. et al. Utilizing solar energy to improve the oxygen evolution reaction kinetics in zinc-air battery. *Nat. Commun.* **10**, 4767 (2019). 31628345 10.1038/s41467-019-12627-2 PMC6800449

[2] Fu, X. et al. Direct solar energy conversion on a zinc–air battery. *Proc. Natl. Acad. Sci. USA* **121**, e2318777121 (2024). 38547057 10.1073/pnas.2318777121 PMC10998616

[3] Li, J., Zhang, K., Wang, B. & Peng, H. Light-assisted metal-air batteries: progress, challenges, and perspectives. *Angew. Chem. Int. Ed.* **61**, e202213026 (2022). 10.1002/anie.202213026 36196996

[4] Liang, S. et al. Efficient carrier separation via Ru@TS@C zeolite: enabling photo-cathodes for high-efficiency photo-assisted metal−air batteries. *Angew. Chem. Int. Ed.* **64**, e202512477 (2025). 10.1002/anie.202512477 40913557

[5] Qi, L. et al. Potential-driven structural evolution of single-atom rhenium sites enables high-performance oxygen electrode reaction and rechargeable Zn-air battery. *CCS Chem.* **7**, 2508–2519 (2024).

[6] Wen, B. et al. Exciton dissociation into charge carriers in porphyrinic metal-organic frameworks for light-assisted Li-O_2_ batteries. *Adv. Mater.* **36**, 2405440 (2024). 10.1002/adma.202405440 38801657

[7] Chen, K. et al. Batch preparation of fe single-atom catalysts for ultrahigh power density zinc-air batteries. *CCS Chem.* **7**, 1844–1855 (2024).

[8] Song, J. et al. Biomimetic design for zinc-based energy storage devices: principles, challenges and opportunities. *Chem. Soc. Rev.* **54**, 8071–8135 (2025). 40762116 10.1039/d5cs00093a

[9] Jiang, Y. et al. Discretizing Cobalt spin–orbitals through tuning the crystal symmetry for Zinc-air batteries. *J. Am. Chem. Soc.* **147**, 25825–25833 (2025). 40644701 10.1021/jacs.5c07623

[10] Jiang, Z. et al. Heavy atom-induced spin–orbit coupling to quench singlet oxygen in a Li-O_2_ battery. *J. Am. Chem. Soc.* **147**, 10992–10998 (2025). 40106234 10.1021/jacs.4c15230

[11] Zhu, Z. et al. Rechargeable batteries for grid scale energy storage. *Chem. Rev.* **122**, 16610–16751 (2022). 36150378 10.1021/acs.chemrev.2c00289

[12] Zhu, Y. et al. Carbon nanotube-supported mixed-valence Mn_3_O_4_ electrodes for high-performance lithium-oxygen batteries. *ChemPhysMater* **3**, 94–102 (2024).

[13] Wang, M. et al. Edge-dislocated WO_3_ photocathode toward efficient photo-assisted Li-O_2_ batteries. *Adv. Mater.* **37**, e01716 (2025). 40685876 10.1002/adma.202501716

[14] Shu, X. et al. Synergistic phosphorus modification of iron-nitrogen-carbon electrocatalysts for durable rechargeable Zinc-air batteries. *CCS Chem.* **8**, 1568–1579 (2025).

[15] Yuan, Z., Mao, H., Yu, D. & Chen, X. Photo-assisted metal-air batteries: recent progress, challenges and opportunities. *Chem. Eur. J.* **29**, e202202920 (2022). 10.1002/chem.202202920 36437508

[16] Xue, H., Gong, H., Yamauchi, Y., Sasaki, T. & Ma, R. Photo-enhanced rechargeable high-energy-density metal batteries for solar energy conversion and storage. *Nano Res. Energy* **1**, e9120007 (2022).

[17] Zhu, Y. et al. Beyond conventional structures: emerging complex metal oxides for efficient oxygen and hydrogen electrocatalysis. *Chem. Soc. Rev.* **54**, 1027–1092 (2025). 39661069 10.1039/d3cs01020a

[18] Li, M., Lv, Q., Si, W., Hou, Z. & Huang, C. Sp-hybridized nitrogen as new anchoring sites of iron single atoms to boost the oxygen reduction reaction. *Angew. Chem. Int. Ed.* **61**, e202208238 (2022). 10.1002/anie.202208238 35879858

[19] Wang, A. et al. Fast-charging Zn-air batteries with long lifetime enabled by reconstructed amorphous multi-metallic sulfide. *Adv. Mater.* **34**, e2204247 (2022). 36177691 10.1002/adma.202204247

[20] Li, Y. et al. Main-group element-boosted oxygen electrocatalysis of Cu-N-C sites for zinc-air battery with cycling over 5000 h. *Nat. Commun.* **15**, 8365 (2024). 39333097 10.1038/s41467-024-52494-0 PMC11436649

[21] Gu, T. et al. Ampere-hour-scale quasi-solid-state zinc–air batteries with a wide operating temperature range (−50 to 60 °C). *J. Am. Chem. Soc.* **147**, 15029–15040 (2025). 40123246 10.1021/jacs.4c16807

[22] Zheng, Y. et al. Selective growth of graphdiyne-based vanadium–iridium oxide interfaces for efficient alkaline oxygen evolution reaction. *ChemPhysMater* **4**, 124–130 (2025).

[23] Liu, J.-N. et al. A data-driven bifunctional oxygen electrocatalyst with a record-breaking ΔE = 0.57 V for ampere-hour-scale zinc-air batteries. *Joule* **8**, 1804–1819 (2024).

[24] Zhu, L. et al. One-step solvothermal preparation of Fe-doped Ni0.85Se/NF: An efficient catalyst for the oxygen evolution reaction. *ChemPhysMater* **4**, 78–85 (2025).

[25] Li, F. et al. Oxygen vacancy-mediated growth of amorphous discharge products toward an ultrawide band light-assisted Li-O2 Batteries. *Adv. Mater.* **34**, 2107826 (2022). 10.1002/adma.202107826 35266208

[26] Si, W., Li, M., Yan, X., Lv, Q. & Huang, C. Porous nitrogen-doped graphdiyne templated from zinc acetylacetonate for enhanced oxygen reduction reaction. *ChemPhysMater* **4**, 274–279 (2025).

[27] Zhou, Y. et al. Lewis-acidic PtIr multipods enable high-performance Li–O_2_ batteries. *Angew. Chem. Int. Ed.* **60**, 26592–26598 (2021). 10.1002/anie.202114067 34719865

[28] Wang, S. et al. Electronic structure formed by Y_2_O_3_-doping in lithium position assists improvement of charging-voltage for high-nickel cathodes. *Nat. Commun.* **16**, 1 (2025). 39746907 10.1038/s41467-024-52768-7 PMC11697207

[29] Liang, S. et al. Accelerated confined mass transfer of MoS_2_ 1D nanotube in photo-assisted metal-air batteries. *Adv. Mater.* **36**, e2307790 (2023). 38088221 10.1002/adma.202307790

[30] Ge, H. et al. Polyoxometallate cluster induced high-entropy oxide Sub-1 nm nanosheets as photoelectrocatalysts for Zn-air batteries. *J. Am. Chem. Soc.* **146**, 10735–10744 (2024). 38574239 10.1021/jacs.4c00652

[31] Fu, X. et al. Directly growing graphdiyne nanoarray cathode to integrate an intelligent solid Mg-moisture battery. *J. Am. Chem. Soc.* **145**, 2759–2764 (2023). 36579966 10.1021/jacs.2c11409

[32] Wang, X.-X. et al. Metal–organic framework-based mixed conductors achieve highly stable photo-assisted solid-state lithium–oxygen batteries. *J. Am. Chem. Soc.* **145**, 5718–5729 (2023). 36880105 10.1021/jacs.2c11839

[33] Li, F. et al. Oxygen vacancy-mediated growth of amorphous discharge products toward an ultrawide band light-assisted Li-O_2_ batteries. *Adv. Mater.* **34**, e2107826 (2022). 35266208 10.1002/adma.202107826

[34] Zhu, Z. et al. Internal electric field and interfacial bonding engineered step-scheme junction for a visible-light-involved lithium-oxygen battery. *Angew. Chem. Int. Ed.* **61**, e202116699 (2022). 10.1002/anie.202116699 35018699

[35] Qiao, G. Y. et al. Perovskite quantum dots encapsulated in a mesoporous metal-organic framework as synergistic photocathode materials. *J. Am. Chem. Soc.* **143**, 14253–14260 (2021). 34459185 10.1021/jacs.1c05907

[36] Lv, Q. et al. Semiconducting metal-organic polymer nanosheets for a photoinvolved Li-O_2_ battery under visible light. *J. Am. Chem. Soc.* **143**, 1941–1947 (2021). 33467851 10.1021/jacs.0c11400

[37] Fang, Z. et al. Capturing visible light in low-band-gap C_4_N-derived responsive bifunctional air electrodes for solar energy conversion and storage. *Angew. Chem. Int. Ed.* **60**, 17615–17621 (2021). 10.1002/anie.202104790 34014029

[38] Kisch, H. Semiconductor photocatalysis-mechanistic and synthetic aspects. *Angew. Chem. Int. Ed.* **52**, 812–847 (2013). 10.1002/anie.201201200 23212748

[39] Sun, X. et al. Solar energy catalysis. *Angew. Chem. Int. Ed.* **61**, e202204880 (2022). 10.1002/anie.202204880 PMC9400894 35471594

[40] Fang, Z. et al. In-situ monitoring of dynamic behavior of catalyst materials and reaction intermediates in semiconductor catalytic processes. *J. Semicond.* **43**, 041104 (2022).

[41] Liu, B., Wu, H. & Parkin, I. P. New Insights into the Fundamental Principle of Semiconductor Photocatalysis. *ACS Omega* **5**, 14847–14856 (2020). 32596623 10.1021/acsomega.0c02145 PMC7315598

[42] Fu, X. et al. Graphdiyne: the emerging energy conversion material. *Adv. Funct. Mater.* **35**, 2424691 (2025).

[43] He, F. & Li, Y. Advances on theory and experiments of the energy applications in graphdiyne. *CCS Chem.* **5**, 72–94 (2023).

[44] Fu, X. et al. Strain and deformation drive efficient hydrogen evolution for adjustable solid mg-moisture battery. *CCS Chem.* **7**, 3473–3483 (2025).

[45] Fu, X. et al. Bandgap-regulation for directly solar energy conversion in zinc-air battery with 4.55% PCE. *Angew. Chem. Int. Ed.* **65**, e17650 (2026). 10.1002/anie.202517650 41410181

[46] Wu, R., Huang, C. & Li, Y. Emerging electrochemical energy conversion materials: Graphdiyne. *Adv. Mater*. 10.1002/adma.202600027, e00027, (2026). 10.1002/adma.202600027 41858186

[47] Zhang, Z. et al. The preparation of porous CuO@F-GDY nano-arrays for high-performance sodium-ion battery anodes. *Small* **22**, e14822 (2026). 41553030 10.1002/smll.202514822

[48] Yan, X. et al. Sp-C coupled geminal-atom facilitate desorption of hydroxyl groups to generate hydrogen. *Carbon* **247**, 121032 (2026).

[49] Gao, J. et al. Boosting Fe cationic vacancies with graphdiyne to enhance exceptional pseudocapacitive lithium intercalation. *Angew. Chem. Int. Ed.* **62**, e202307874 (2023). 10.1002/anie.202307874 37408177

[50] Wu, R. et al. Recent progress in the preparation of graphdiyne-based materials. *ChemistryEurope* **3**, e202500141 (2025).

[51] Do, D. P., Lee, E., Bui, V. Q. & Lee, H. Recent progress in two-dimensional graphdiyne: Synthesis, characterization, and applications. *ChemPhysMater* **4**, 91–107 (2025).

[52] Kan, X. et al. Interfacial synthesis of conjugated two-dimensional N-Graphdiyne. *ACS Appl. Mater. Inter.* **10**, 53–58 (2018). 10.1021/acsami.7b17326 29260842

[53] Wang, M. et al. Modulation of electronic structure of metal-free graphdiyne via precise nitrogen modification for oxygen evolution reaction. *ChemPhysMater* **4**, 289–295 (2025).

[54] Huang, H. et al. Intrinsic nitrogen-doped graphdiyne boosts sodium storage via fast ion desolvation kinetics. *Chem. Eng. J.* **532**, 174229 (2026).

[55] Liu, B. et al. Revealing the mechanism of sp-N doping in graphdiyne for developing site-defined metal-free catalysts. *Adv. Mater.* **35**, 2206450 (2023). 10.1002/adma.202206450 36217835

[56] Yang, Q. et al. Stabilizing interface pH by N-modified graphdiyne for dendrite-free and high-rate aqueous Zn-ion batteries. *Angew. Chem. Int. Ed.* **61**, e202112304 (2022). 10.1002/anie.202112304 34799952

[57] Fang, Y., Liu, Y., Qi, L., Xue, Y. & Li, Y. 2D graphdiyne: an emerging carbon material. *Chem. Soc. Rev.* **51**, 2681–2709 (2022). 35253033 10.1039/d1cs00592h

[58] Guan, D. H. et al. Light/electricity energy conversion and storage for a hierarchical porous In_2_S_3_@CNT/SS cathode towards a flexible Li-CO_2_ battery. *Angew. Chem. Int. Ed.* **59**, 19518–19524 (2020). 10.1002/anie.202005053 32419313

[59] Gao, J. et al. The alkynyl π Bond of sp-C enhanced rapid, reversible li-c coupling to accelerate reaction kinetics of lithium ions. *J. Am. Chem. Soc.* **146**, 27030–27039 (2024). 39300785 10.1021/jacs.4c08920 PMC11669086

[60] Kumar, A. et al. Light vs heat: dissecting the de-intercalation in photo-rechargeable batteries. *Nano Lett.* **25**, 628–634 (2025). 39760220 10.1021/acs.nanolett.4c04013

[61] Pujari, A. et al. Does heat play a role in the observed behavior of aqueous photobatteries?. *ACS Energy Lett.* **8**, 4625–4633 (2023). 37969251 10.1021/acsenergylett.3c01627 PMC10644369

[62] Wang, H. et al. Porous materials applied in nonaqueous Li-O_2_ batteries: status and perspectives. *Adv. Mater.* **32**, 2002559 (2020). 10.1002/adma.202002559 32715511

[63] Lu, J. et al. Aprotic and aqueous Li–O_2_ batteries. *Chem. Rev.* **114**, 5611–5640 (2014). 24725101 10.1021/cr400573b

[64] Zheng, X. et al. Theoretical design and structural modulation of a surface-functionalized Ti_3_C_2_T_x_ MXene-based heterojunction electrocatalyst for a Li-oxygen battery. *ACS Nano* **16**, 4487–4499 (2022). 35188376 10.1021/acsnano.1c10890

[65] Li, C. S., Sun, Y., Gebert, F. & Chou, S. L. Current progress on rechargeable magnesium-air battery. *Adv. Energy Mater.* **7**, 1700869 (2017).

[66] Zhang, J. et al. Approaches to construct high-performance Mg-air batteries: from mechanism to materials design. *J. Mater. Chem. A* **11**, 7924–7948 (2023).

[67] Zhu, Z., Shi, X., Fan, G., Li, F. & Chen, J. Photo-energy conversion and storage in an Aprotic Li-O_2_ battery. *Angew. Chem. Int. Ed.* **58**, 19021–19026 (2019). 10.1002/anie.201911228 31591805

