# Supplementary material for: Direct solar energy charging of metal||air batteries enabled by photo-coupled electrodes
Source: Nat Commun. 2026 Jun 2;17:7060. doi: 10.1038/s41467-026-73926-z (PMC13392032; doi:10.1038/s41467-026-73926-z)
Supplement: Supplementary file 1 — Supplementary information [file 41467_2026_73926_MOESM1_ESM.pdf]

Supplementary information

Direct solar energy charging of metal||air batteries enabled by photo-coupled electrodes

Xinlong Fu[1, †], Yi Wang[1, 2, †], Changshui Huang[1, 2, *], Feng He[1, *], Ruiqiao Wu[1, 2], Qian Chang[1], Jingxiang Yang[1, 2], Yuliang Li[1, 2, *]

[1.] Beijing National Laboratory for Molecular Sciences (BNLMS), Institute of Chemistry, Chinese Academy of Sciences, Beijing 100190, China.

[2.] School of Chemical Sciences, University of Chinese Academy of Sciences, Beijing 100049, China.

[†] These authors contributed equally to this work: X. Fu, Y. Wang.

*Corresponding author: huangcs@iccas.ac.cn; hefeng2018@iccas.ac.cn; ylli@iccas.ac.cn

Table of contents

Supplementary Figs. 1-82 .... S1

Supplementary Tables 1-9 .... S84

References S94

Supplementary Figs.

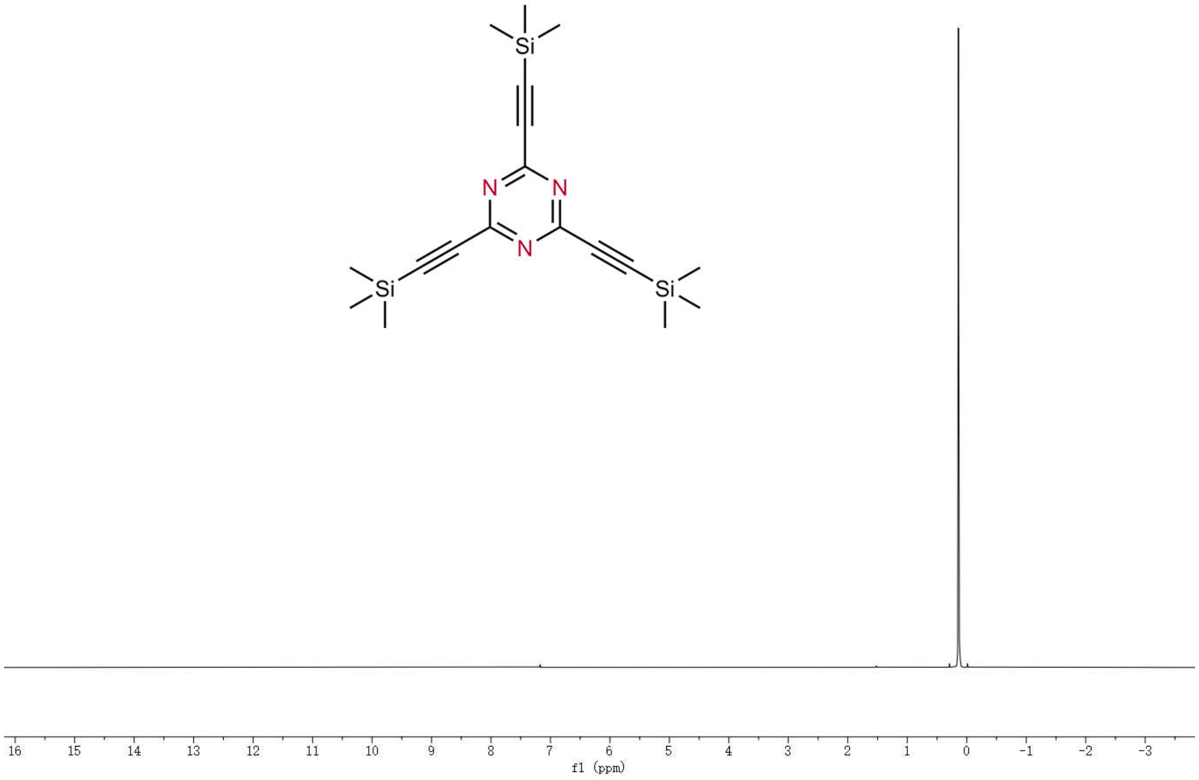

Supplementary Fig. 1. ^{1}H NMR spectrum of the precursor.

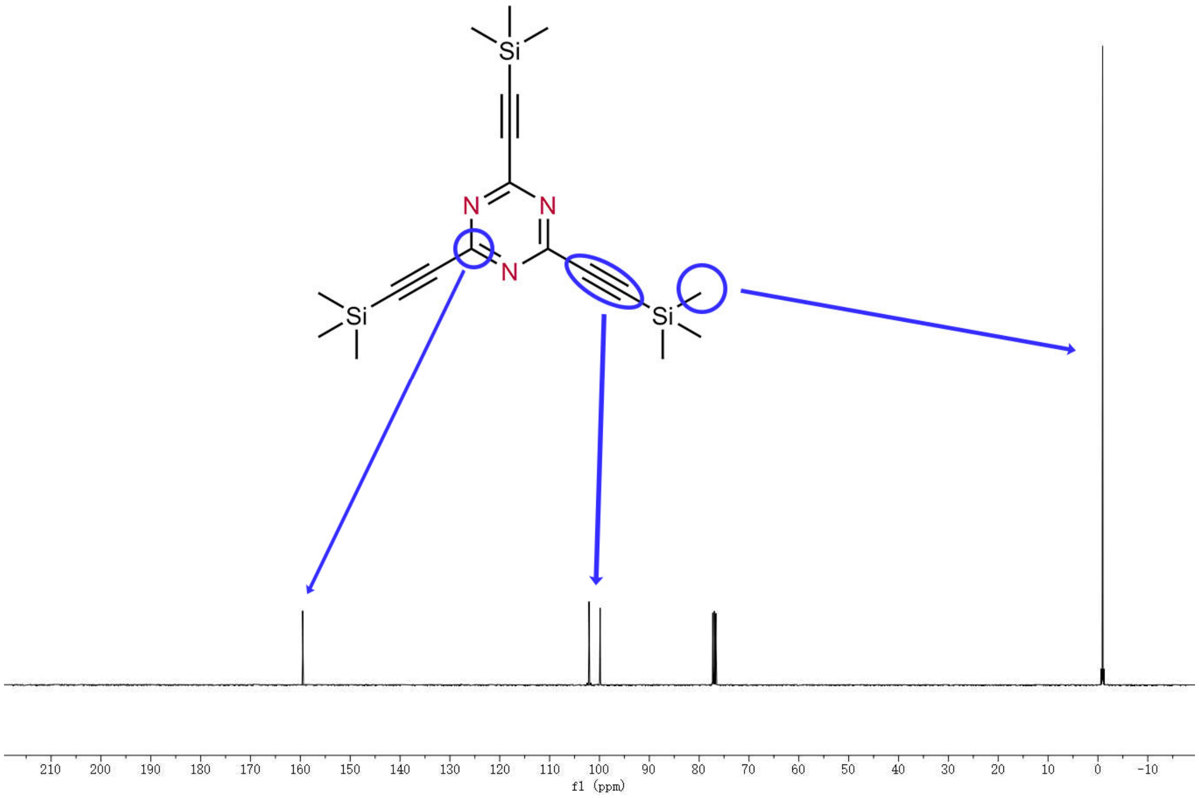

Supplementary Fig. 2. ^{13}C NMR spectrum of the precursor.

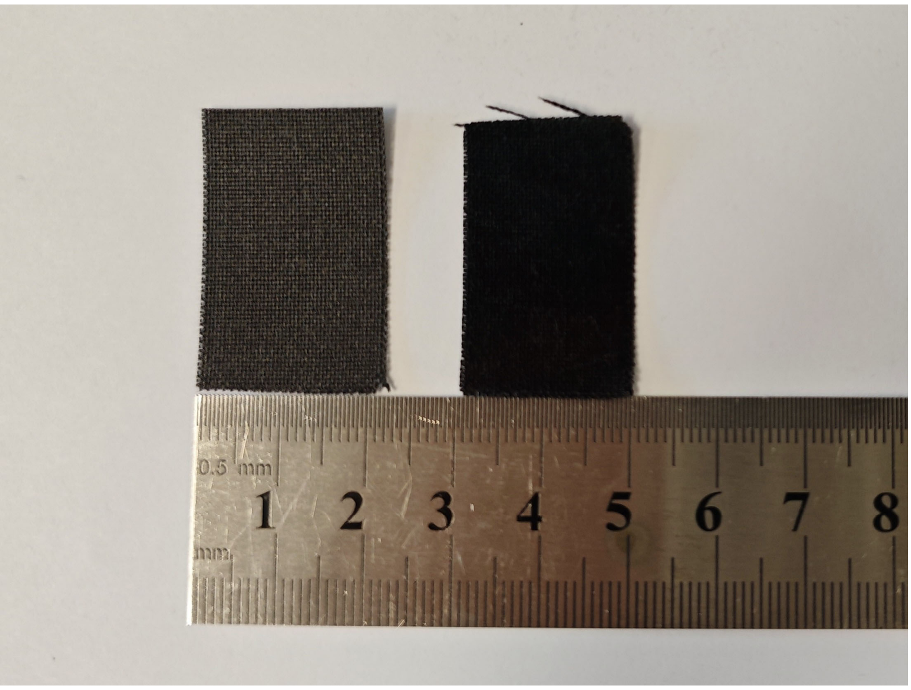

Supplementary Fig. 3. Optical image of carbon cloth (left) and NGDY grown on CC (right).

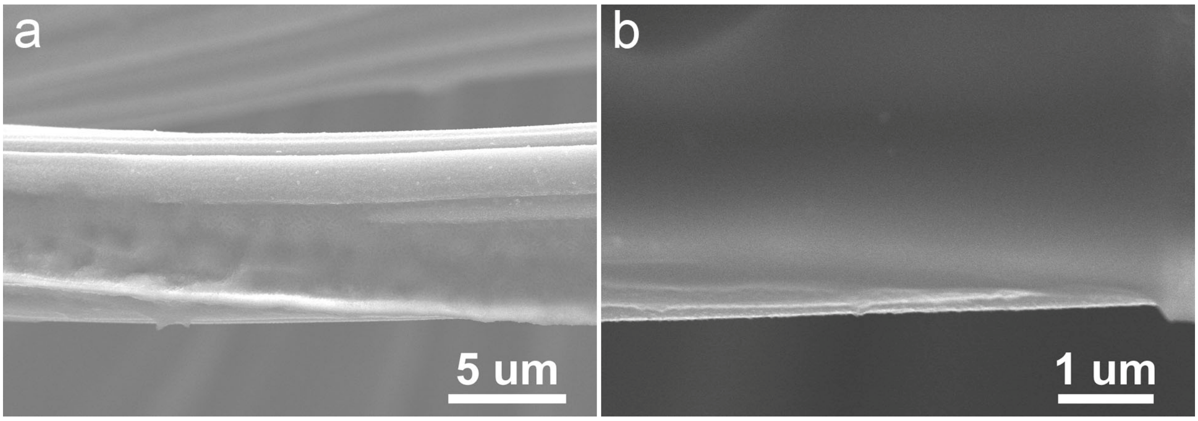

Supplementary Fig. 4. SEM images of CC.

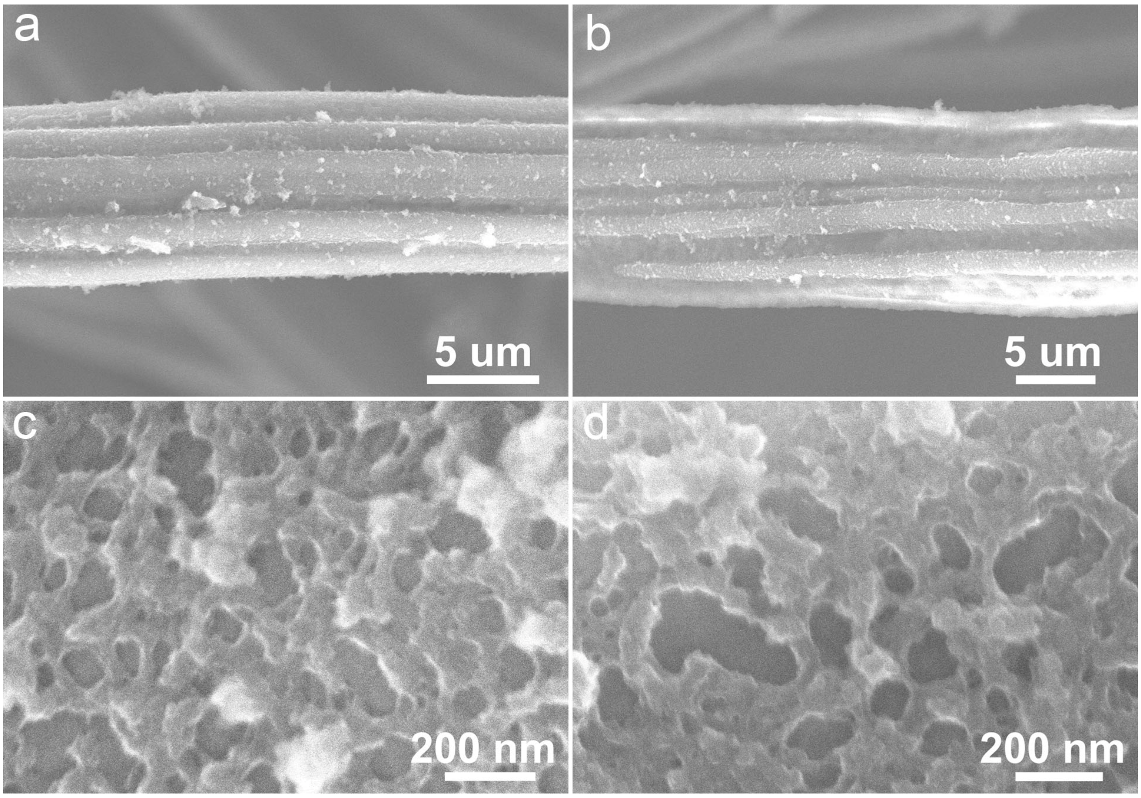

Supplementary Fig. 5. SEM images of NGDY/CC.

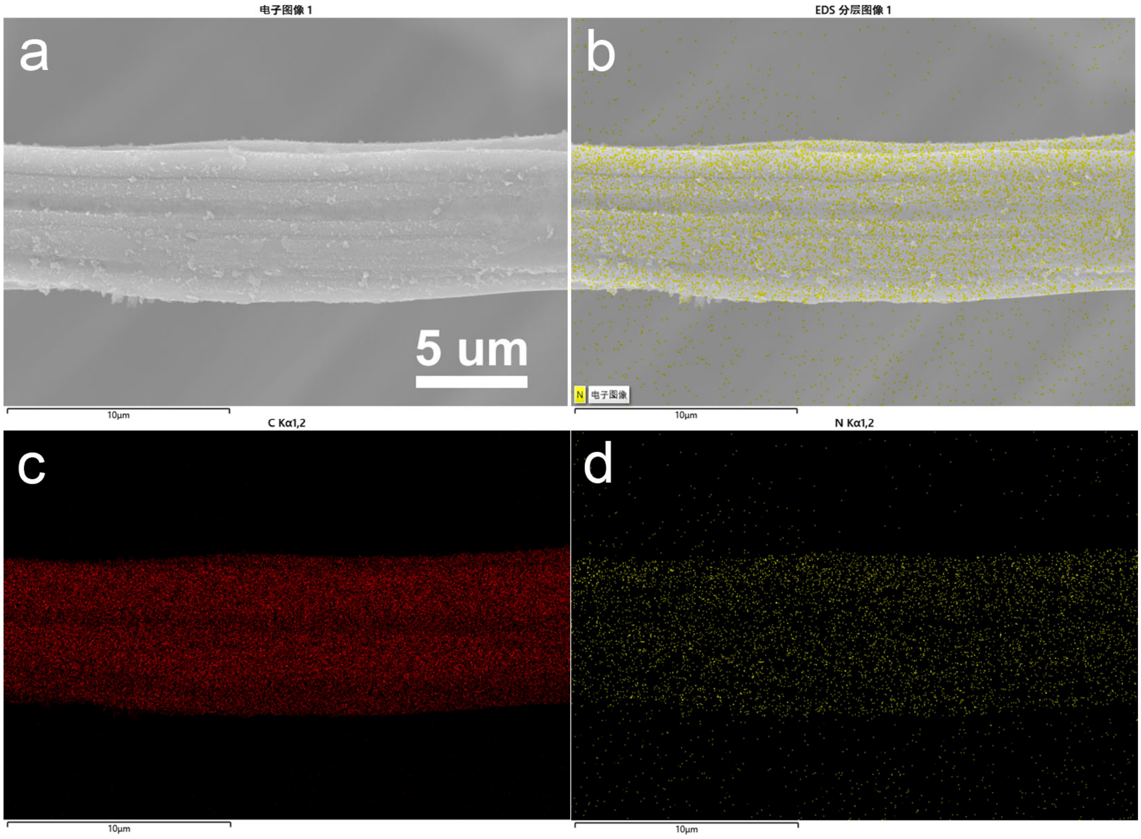

Supplementary Fig. 6. Elemental mapping of the C and N in NGDY.

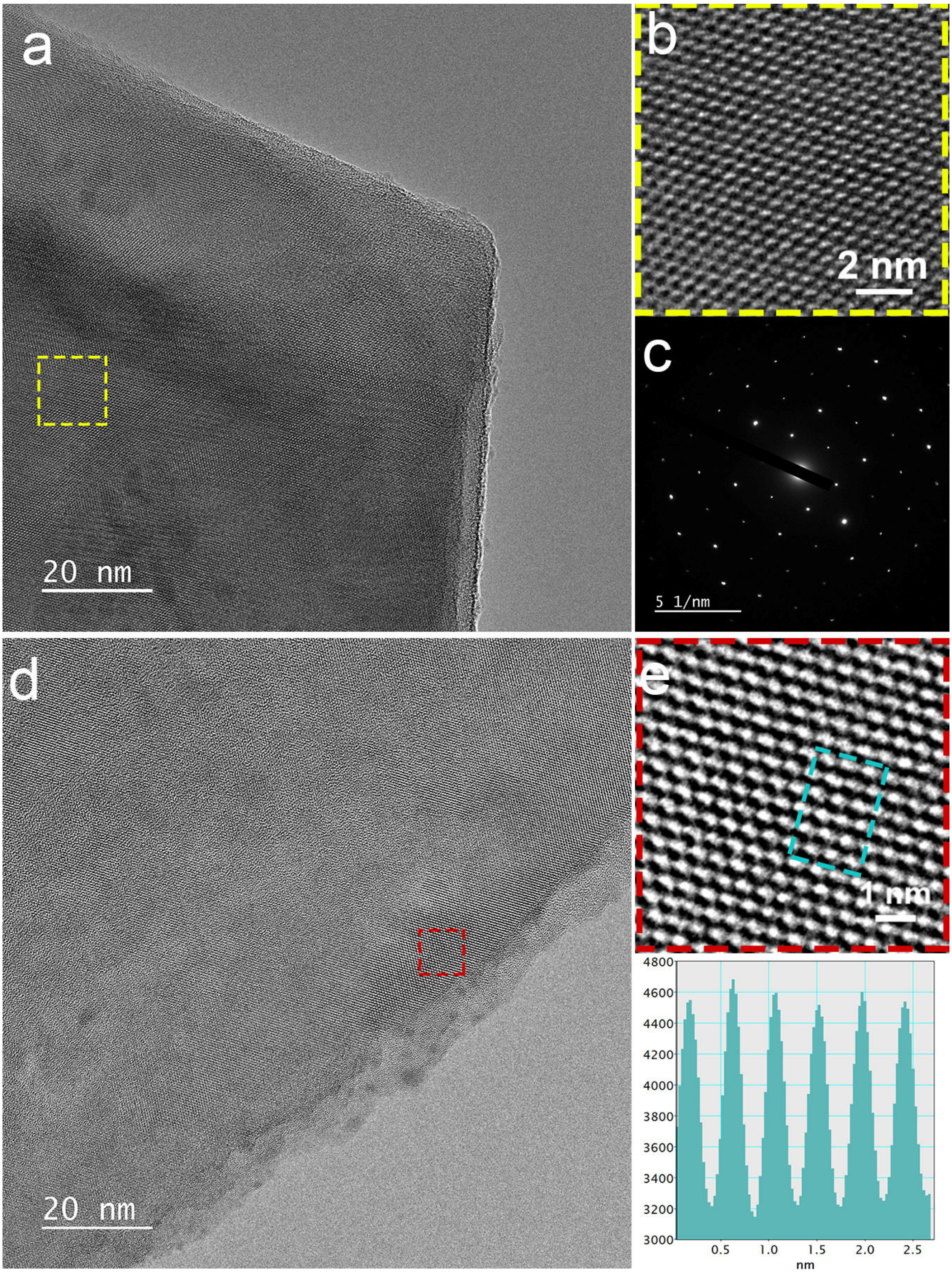

Supplementary Fig. 7. (a, d) TEM images of NGDY. (b) Magnified TEM image of NGDY. (c) SAED pattern. (e) Magnified TEM image of NGDY and corresponding sectional analysis along the dashed line.

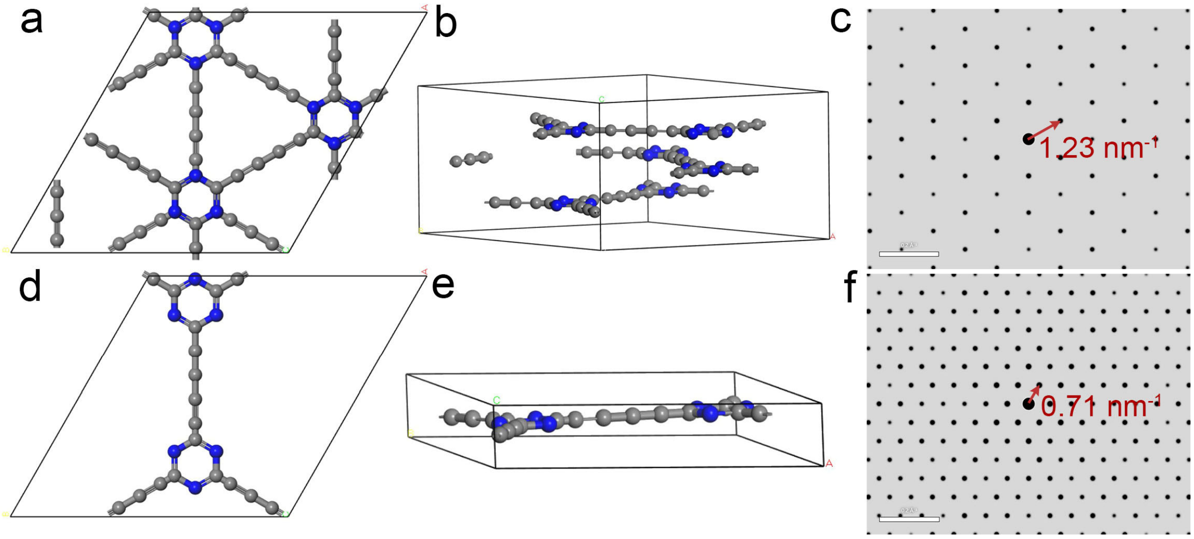

Supplementary Fig. 8. (a) Top view and (b) side view of the crystal structure of NGDY with ABC stacking structure. (c) Simulated SAED pattern using the ABC stacking structure mode. (d) Top view and (e) side view of the crystal structure of NGDY with AA stacking structure. (f) Simulated SAED pattern using the AA stacking structure mode.

To verify the stacking mode of NGDY, we proposed three possible models: AA stacking mode, ABC stacking mode, and 9-layer stacking mode ($A_1B_1C_1A_2B_2C_2A_3B_3C_3$ stacking) and simulated their SAED patterns, as shown in Figure 1k-m and Supplementary Fig. 8. Comparing the experimental SAED pattern result with the simulated SAED patterns, it is obvious that the experimental result was in perfect agreement with the simulated pattern of 9-layer stacking mode.

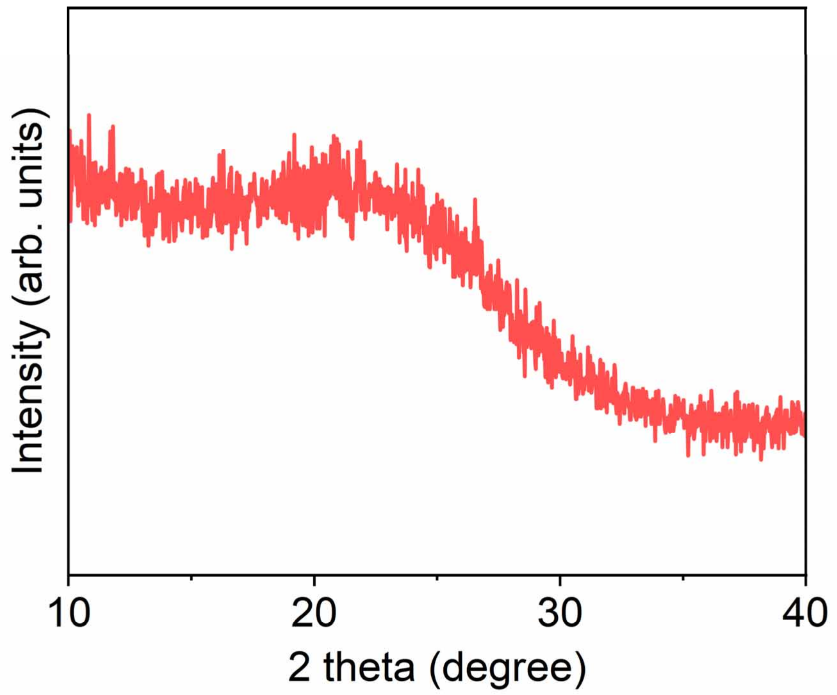

Supplementary Fig. 9. XRD pattern of NGDY.

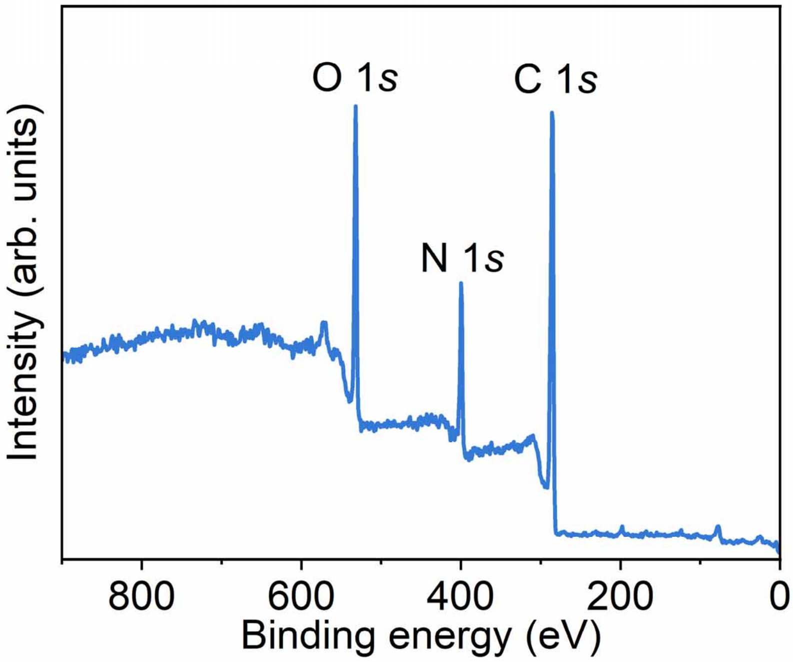

Supplementary Fig. 10. XPS survey spectrum of NGDY.

The C 1*s* peak can be divided into four sub peaks, corresponding to *sp*-C (284.6 eV), sp^2-C (285.6 eV), C-O (286.7 eV), and C=O (287.9 eV) (Figure 1o). The experimental peak area ratio of *sp*-C to sp^2-C were calculated to be 2:1, which precisely matched theoretical values and confirmed the designed hybrid carbon hybridization. The N 1*s* peak can be divided into pyridine nitrogen at 399.15 eV and proton nitrogen at 400.1 eV (Figure 1p). The proton nitrogen may originate from the complexation of pyridine N with impure molecules.[1]

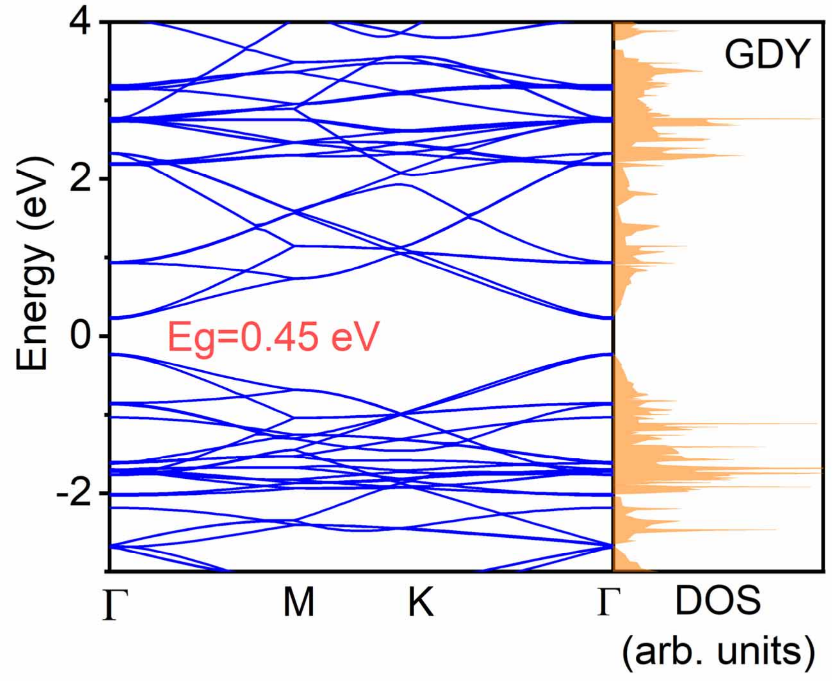

Supplementary Fig. 11. The calculated band structure and DOS of GDY.

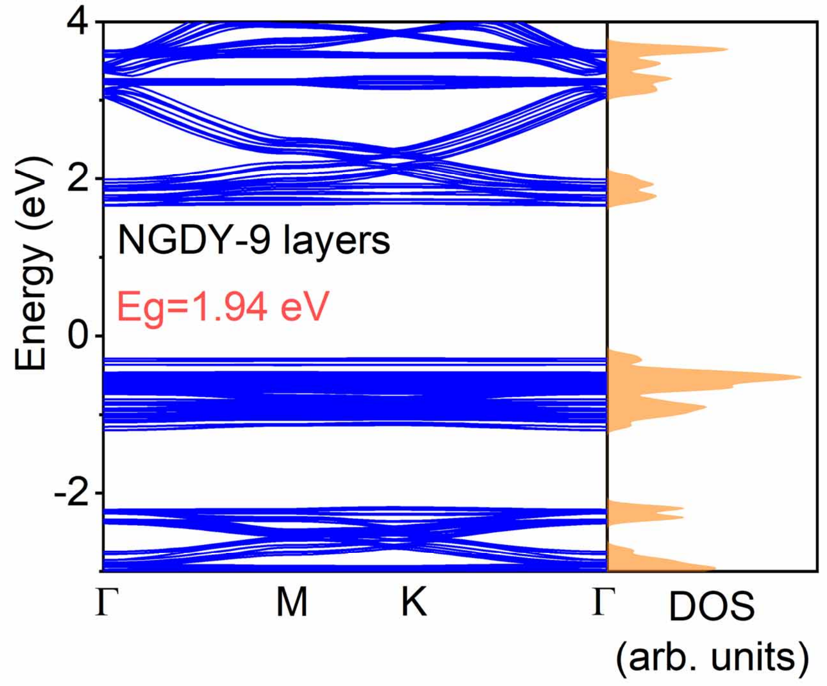

Supplementary Fig. 12. The calculated band structure and DOS of NGDY based on 9-layer stacked model.

The optimized nine-layer model was adopted to get closer to the actual situation, and the band gap was calculated to be 1.94 eV. The narrowing of the band gap was caused by the interaction between the layers. The remaining minor discrepancy between DFT-calculated bandgap and experimental bandgap arises from the differences between the fundamental electronic bandgap and the optically measured transition onset.

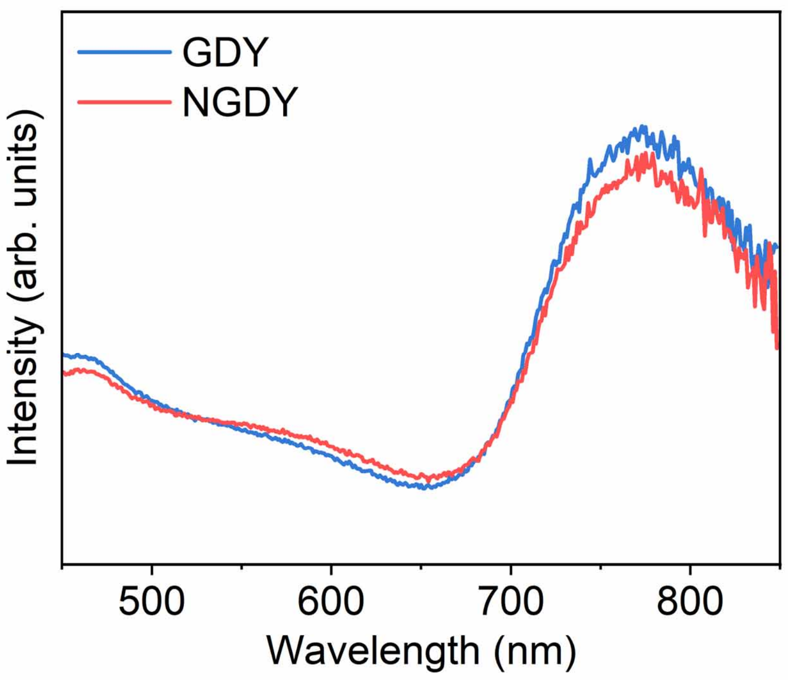

Supplementary Fig. 13. Steady-state PL spectra of GDY and NGDY samples under excitation wavelength of 390 nm.

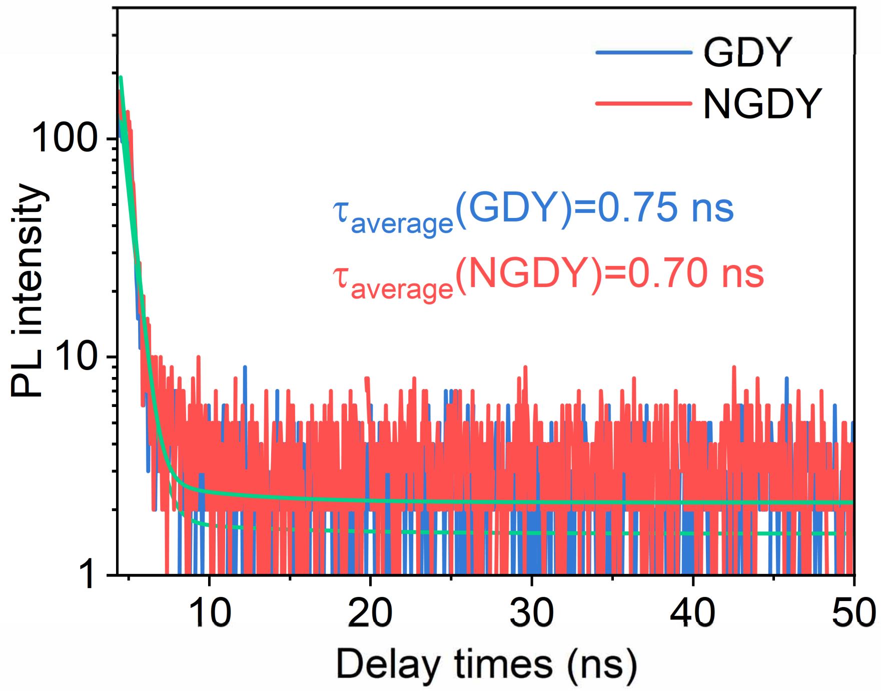

Supplementary Fig. 14. PL decay spectra measured at λ = 405 nm.

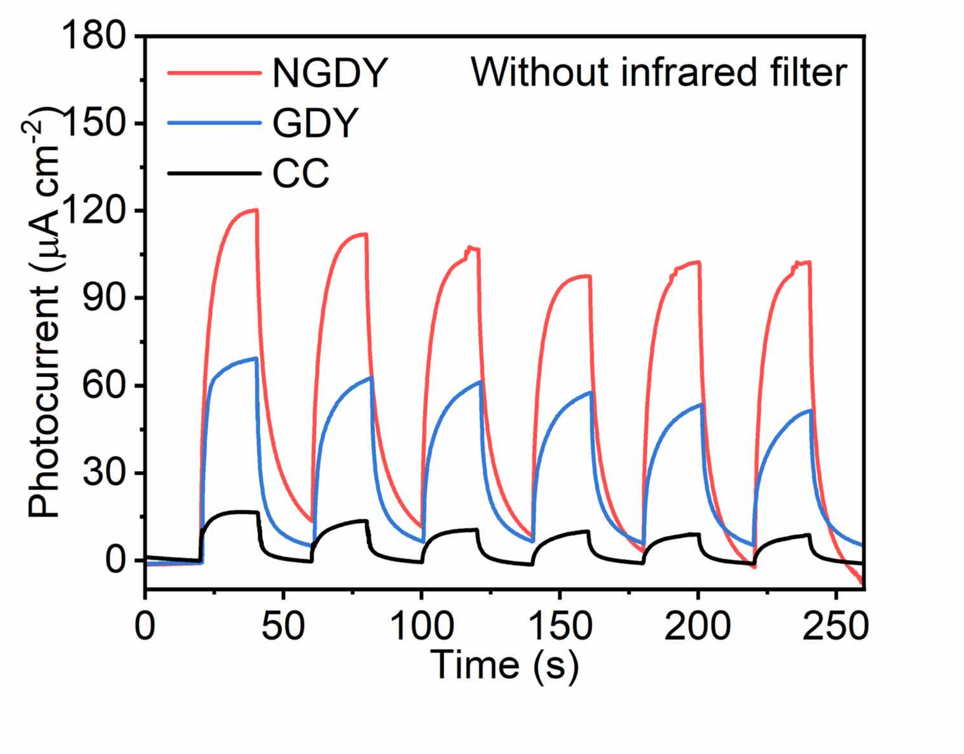

Supplementary Fig. 15. Photocurrent densities under intermittent light irradiation without 800 nm infrared filter (1 M KOH, light source: > 320 nm).

This control experiment presents the photocurrent response without the 800 nm infrared filter. Under full-spectrum illumination, the bare carbon cloth (CC) substrate exhibits a detectable transient current, which is primarily attributed to photothermal effects arising from infrared absorption. In stark contrast, when the infrared component is filtered out (Fig. 2j), this signal in CC diminishes to a negligible level (see main text).

Crucially, the photocurrents of both GDY and NGDY remain substantial and stable regardless of the presence of the IR filter, demonstrating that their response originates from intrinsic photoexcitation rather than thermal artifacts. This direct comparison underscores the necessity of spectral filtering to isolate genuine photoelectrochemical activity and confirms that the performance enhancements reported in this work are driven by the semiconductor properties of the materials, not by incidental heating.

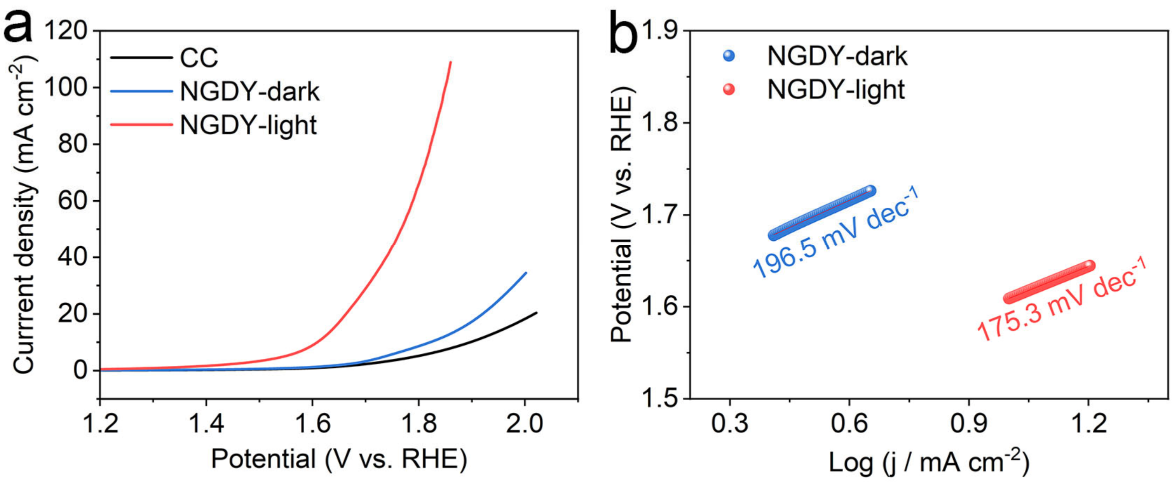

Supplementary Fig. 16. LSV curves of CC and NGDY for OER with or without light irradiation.

The linear sweep voltammetry (LSV) curves were tested to study the OER photo/electrocatalytic performance of NGDY, showing a significant improvement of catalytic current under light irradiation. The overpotential at 10 mA cm^{-2} decreased from 589.9 mV in the dark to 376.9 mV, highlighting the crucial role of light in improving the OER kinetics of NGDY.

The Tafel slopes decreased form 196.5 mV dec^{-1} in the dark to 175.3 mV dec^{-1} under light irradiation, which is a clear indicator of accelerated OER kinetics. The decrease in Tafel slope signifies a lower energetic barrier for the rate-determining step and reflects a change in the reaction pathway facilitated by photoexcitation. Importantly, such a change in Tafel slope cannot be explained by a simple thermal effect or an additive photocurrent, as a purely thermal contribution would typically shift the entire polarization curve to lower overpotentials but would not substantially alter the Tafel slope. And a mere photocurrent addition would increase the current at a given potential without modifying the kinetic parameter (b value). Therefore, the observed lowering of the Tafel slope provides direct electrochemical evidence that light reshapes the OER energetics on NGDY, consistent with our proposed mechanism of photoinduced charge redistribution and transient active-site generation.

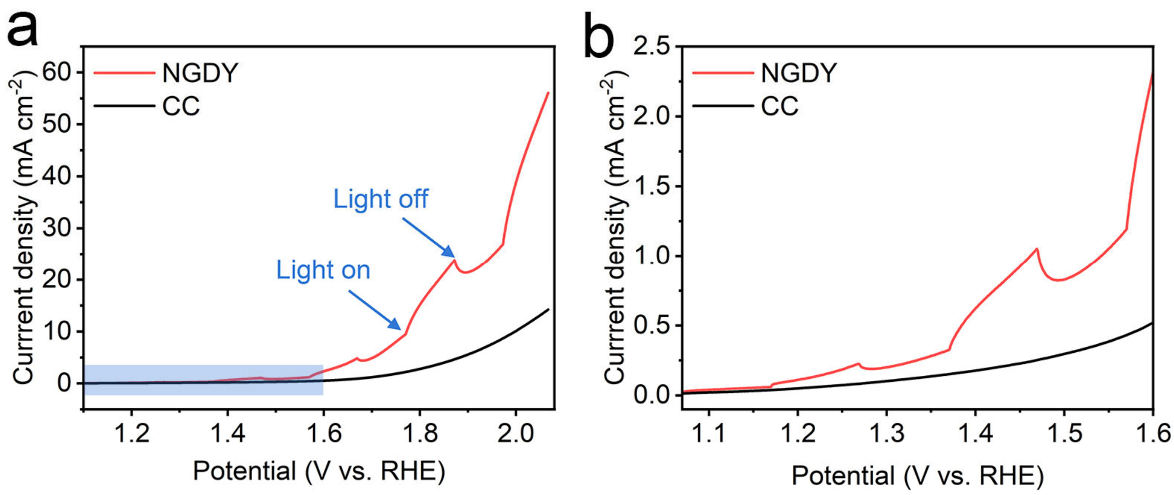

Supplementary Fig. 17. (a) LSV curves of CC and NGDY/CC for OER under intermittent illumination in 1 M KOH. (b) Enlarged LSV curves.

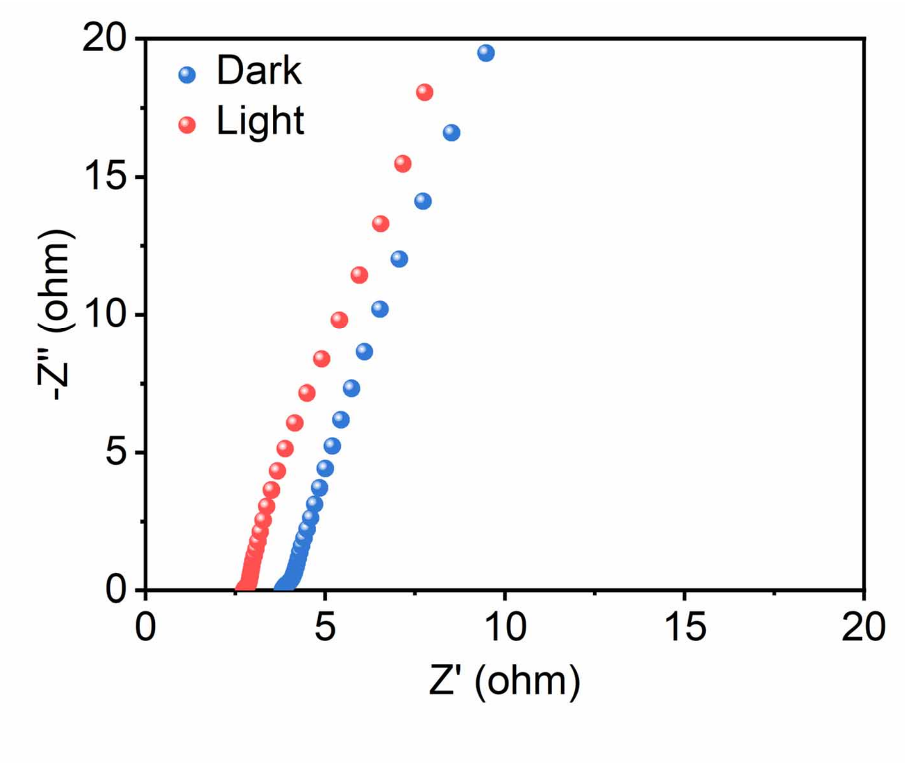

Supplementary Fig. 18. Nyquist plots of NGDY in the dark and light irradiation.

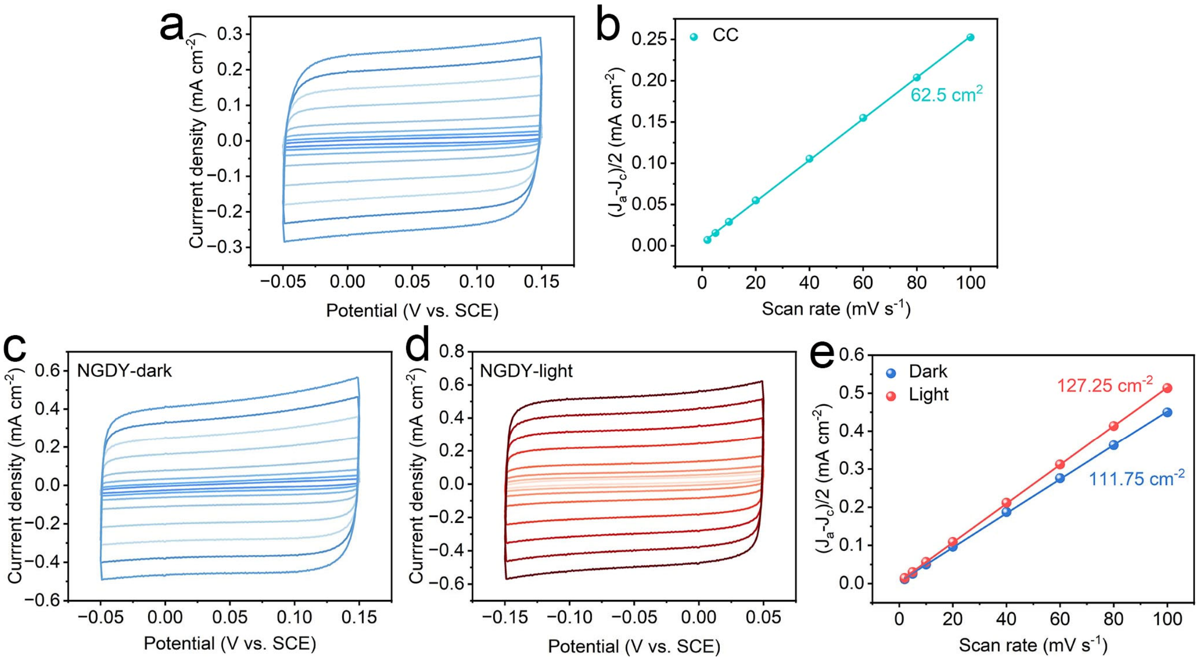

Supplementary Fig. 19. CV measurements of (a) CC a127.25nd (b) the capacitive current density for CC against scan rates. CV measurements of NGDY/CC (c) in the dark and (d) under light irradiation and (e) the capacitive current density for NGDY/CC against scan rates.

The electrochemically active surface area (ECSA) was measured by CV within a non-Faradaic potential range. The scan rates ranged from 2 mV s^{-1} to 100 mV s^{-1} in 1 M KOH solution. The double layer capacitance (C_{dl}) equals the slope of the liner fitting of the charging current density differences ($J = \frac{J_a - J_c}{2}$) at a potential of 0 V (vs. SCE) against the scan rate. The ECSA was estimated by using the following equation:

$$ECSA = C_{dl} / C_S$$

Where C_S is 0.040 mF cm^{-2} based on reported values. 62.5 cm^2, and the ECSA of CC was calculated to be 111.75 cm^2 in the dark and 127.25 cm^2 under light irradiation. The significant increase in ECSA of NGDY under light irradiation indicated that light activates the active site in NGDY and increases the effective area involved in the electrochemical reaction.

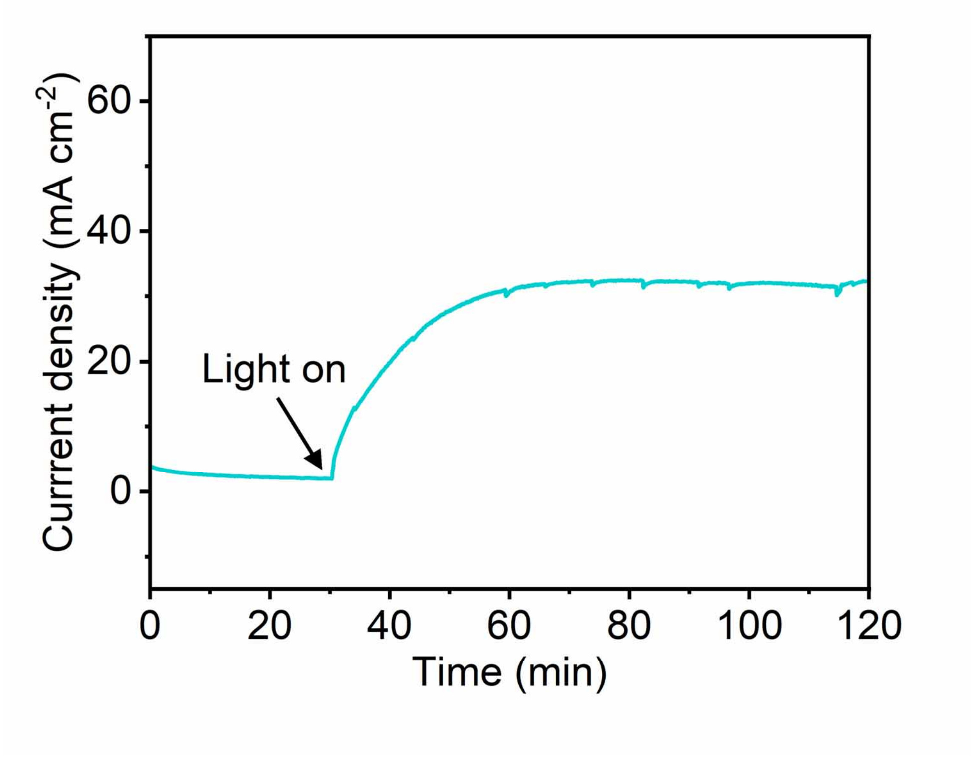

Supplementary Fig. 20. Current-time curve of NGDY/CC for OER in 1 M KOH at 1.8 V (vs. RHE) with and without light irradiation.

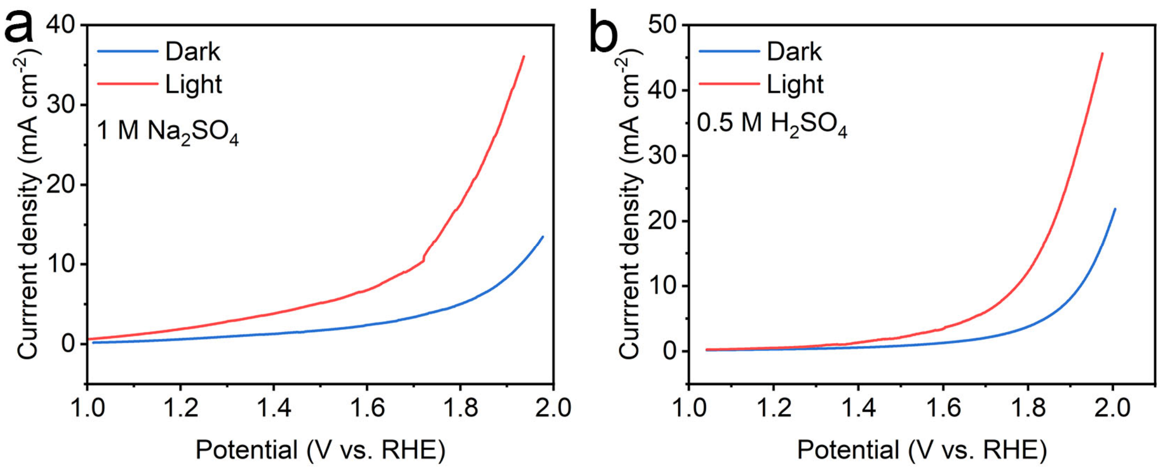

Supplementary Fig. 21. LSV curves of NGDY/CC for OER in (a) 1 M Na_2SO_4 and (b) 0.5 M H_2SO_4.

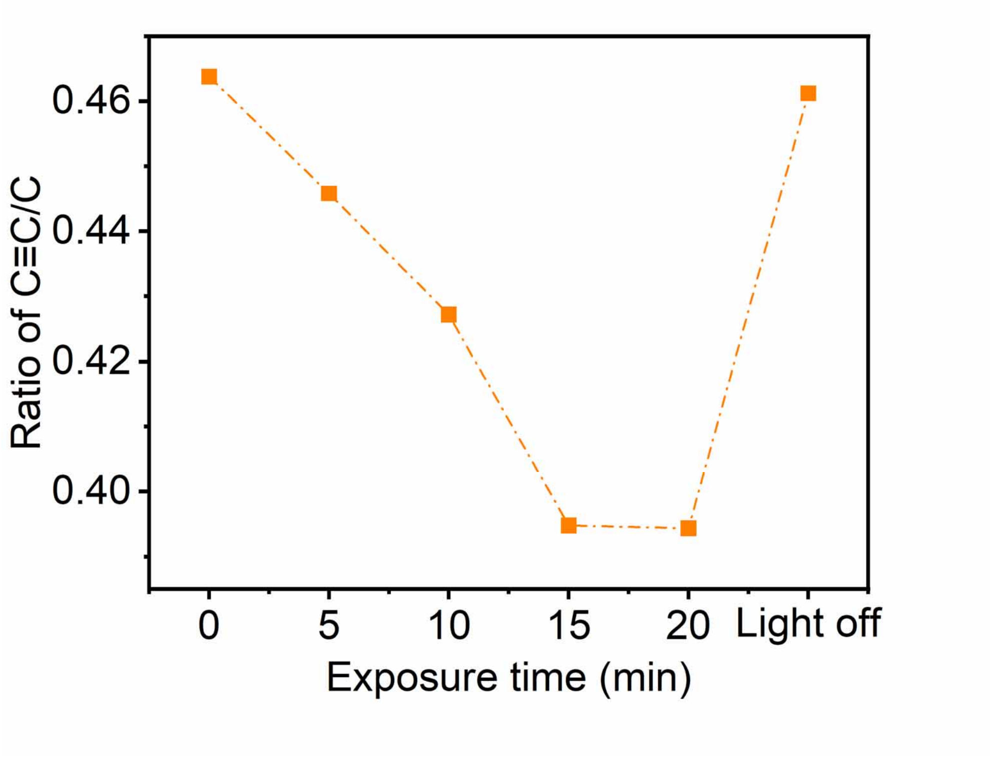

Supplementary Fig. 22. The changes in the proportion of *sp*-C throughout the C peak under light irradiation.

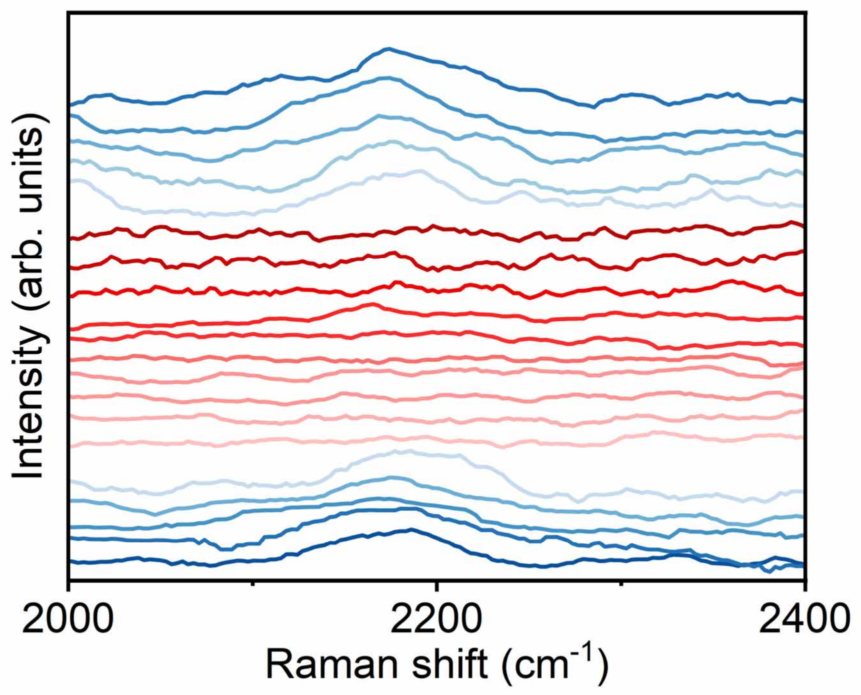

Supplementary Fig. 23. The enlarged view at 2000 - 2400 cm^{-1} in Fig. 3d.

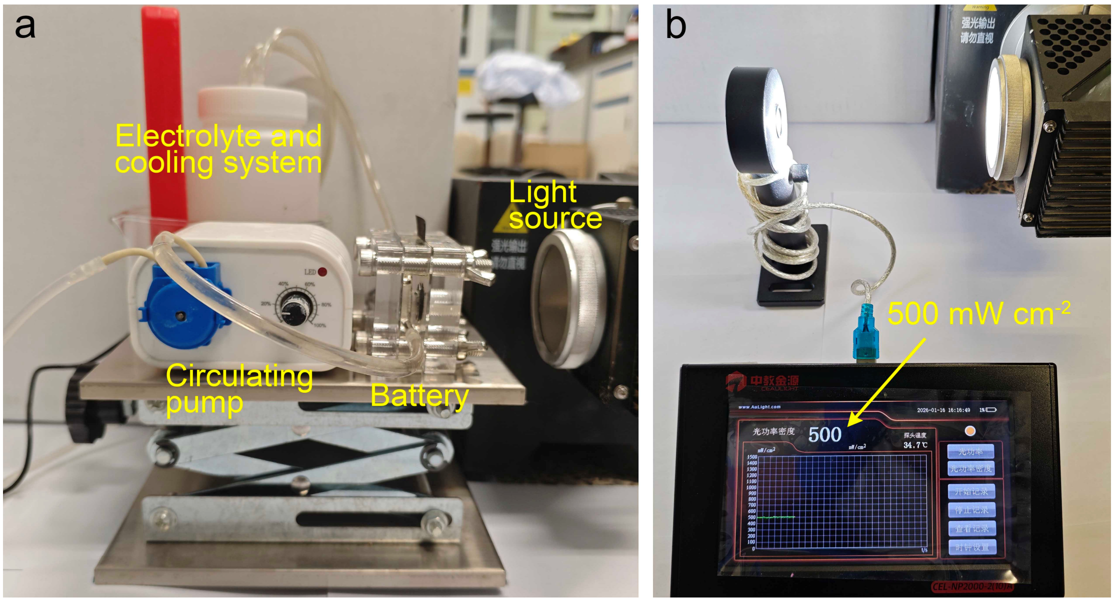

Supplementary Fig. 24. (a) The optical photograph of the working device of PZAB. (b) The light intensity was calibrated to 500 mW cm^{-2} using a photo radiometer.

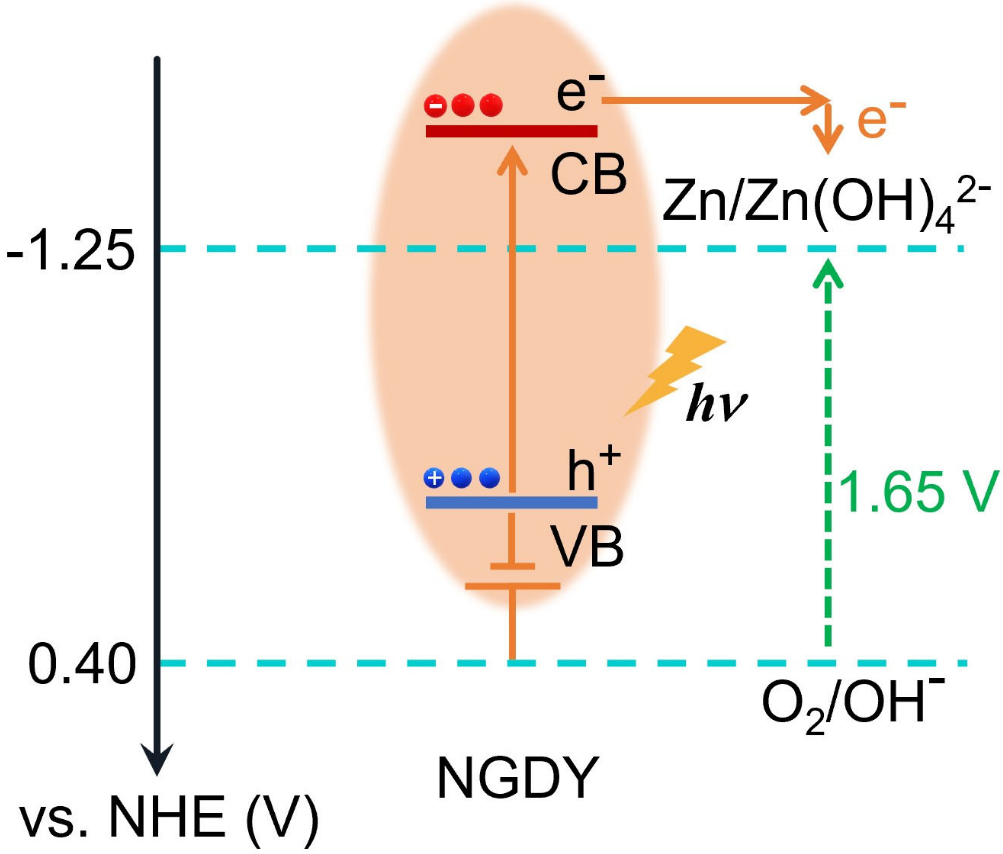

Supplementary Fig. 25. The proposed operating mechanism of PZAB.

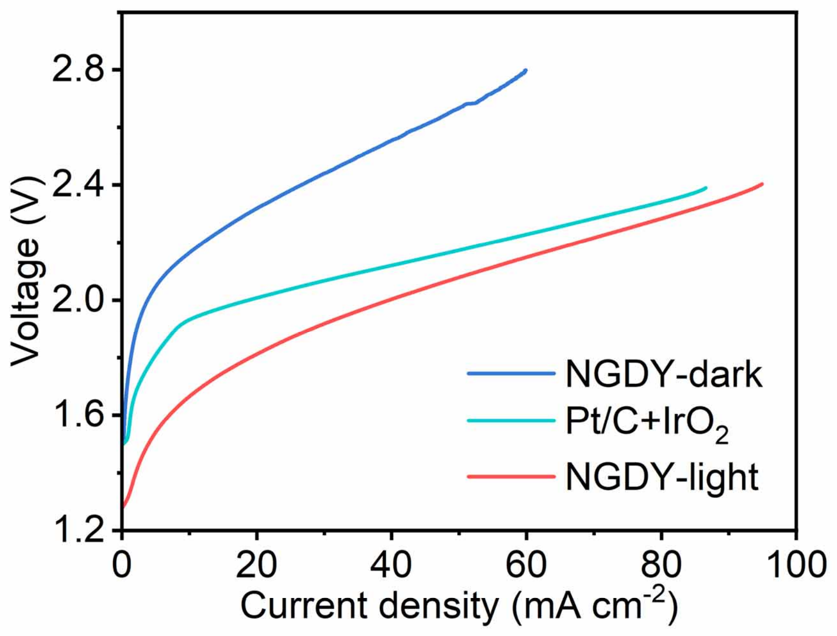

Supplementary Fig. 26. Charge (in the dark and light) polarization curves of PZAB assembled with NGDY and commercial Pt/C+IrO_2.

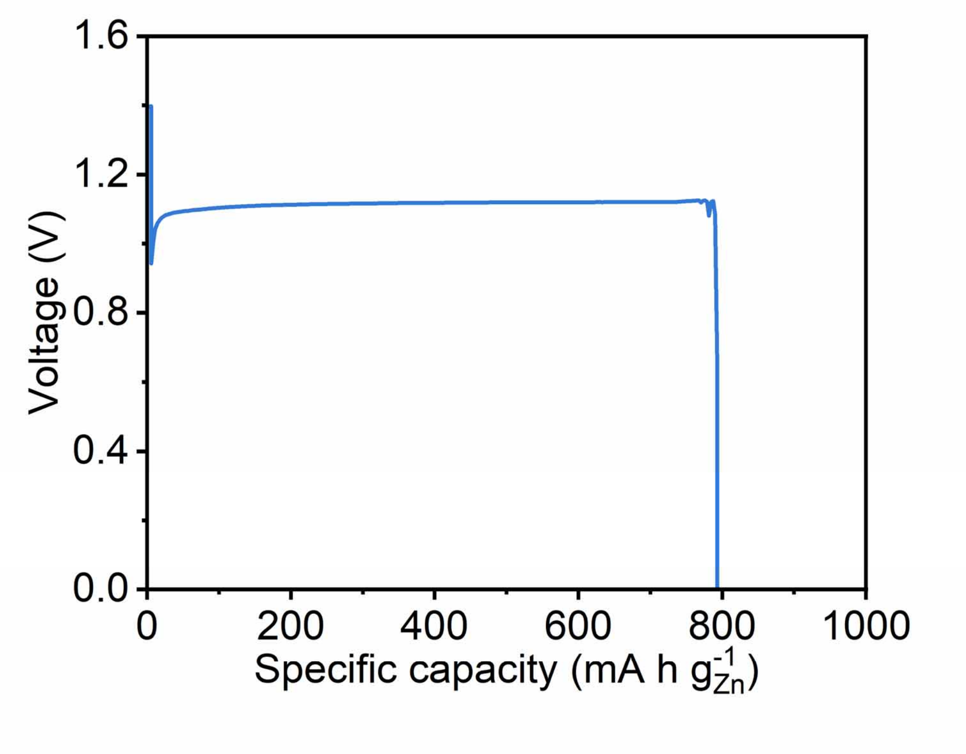

Supplementary Fig. 27. Specific capacity of PZAB based on NGDY (current density: 20 mA cm^{-2}). The specific energy was calculated based on the consumed Zn (119 mg).

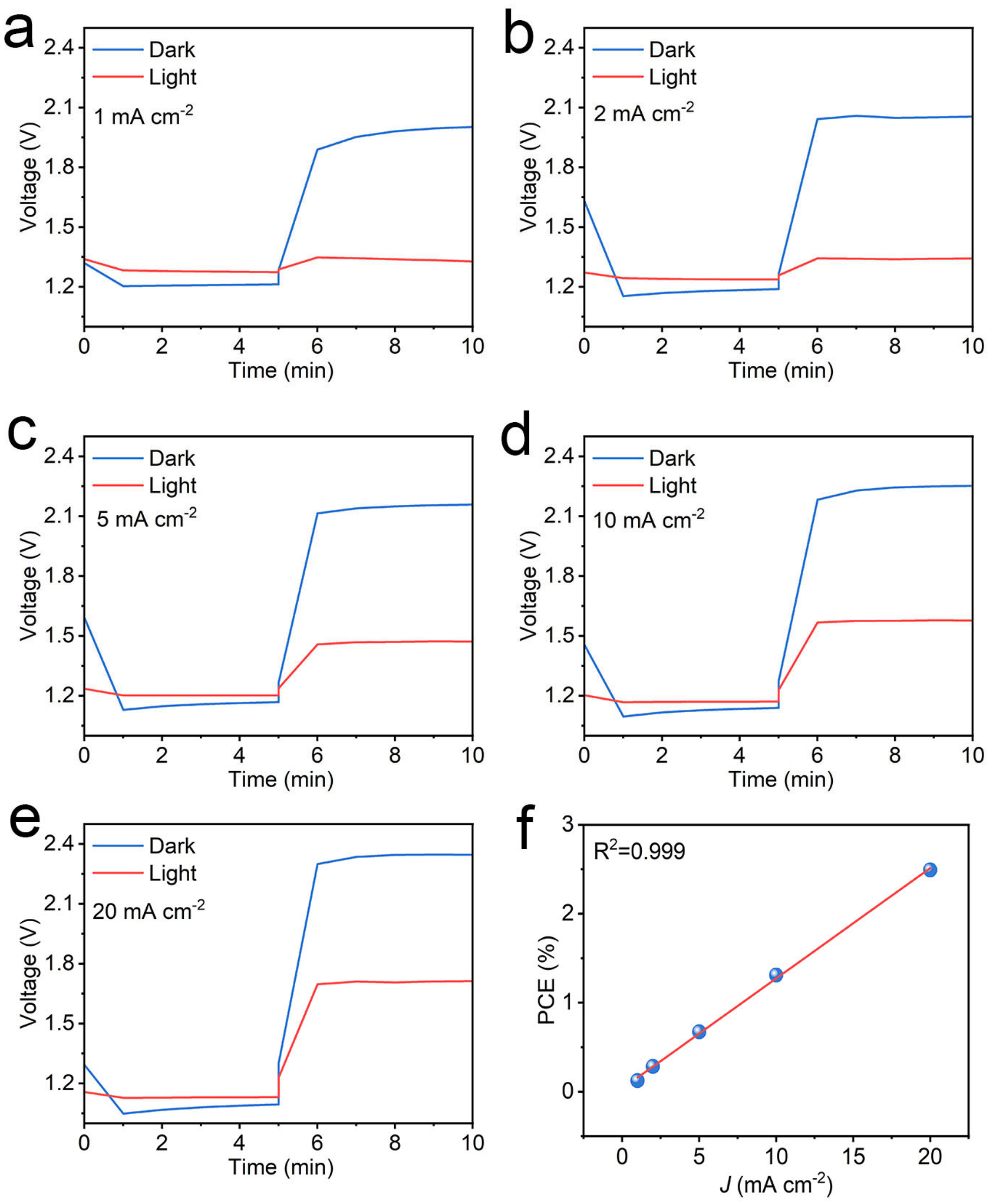

Supplementary Fig. 28. Discharge and charge curves of NGDY-based PZAB in the dark and under light irradiation at various current density from a) 1 mA cm^{-2}, b) 2 mA cm^{-2}, c) 5 mA cm^{-2}, d) 10 mA cm^{-2} and e) 20 mA cm^{-2}. f) The corresponding PCE at different current density.

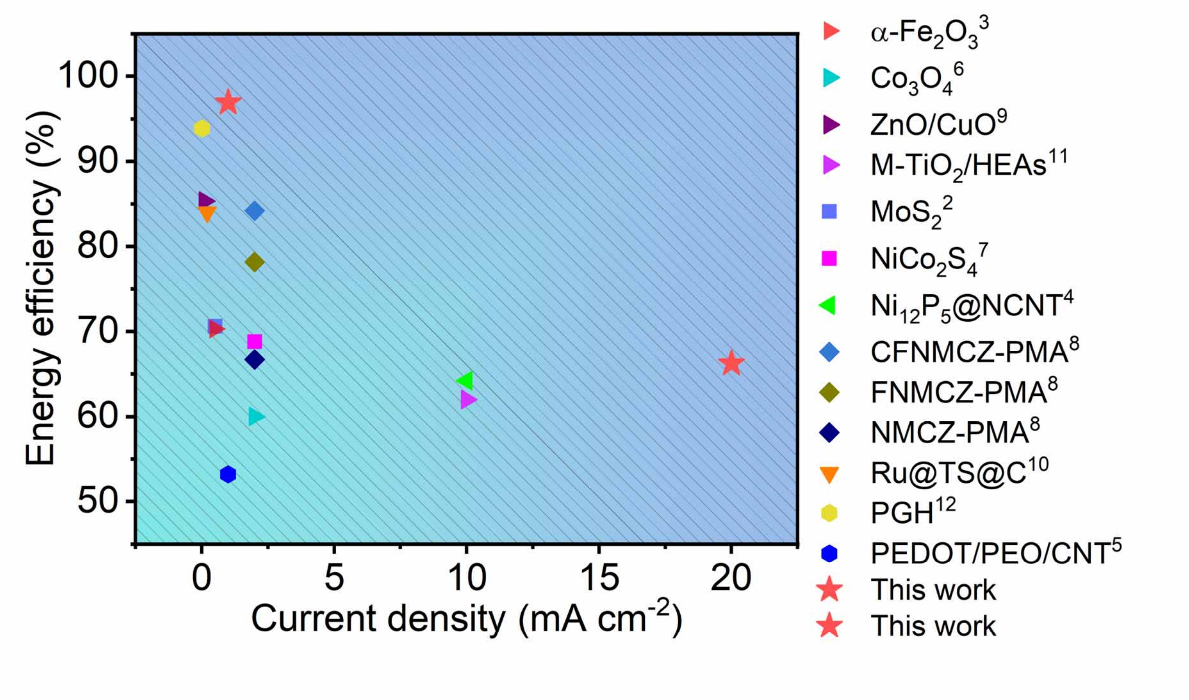

Supplementary Fig. 29. Comparation of energy efficiency and current density of NGDY based PZAB and other reported electrocatalysts based photo-coupled two-electrode Zn||air battery systems.[2,3,4,5,6,7,8,9,10,11,12] The source of the literature data shown in this figure can be found in Supplementary Table 8.

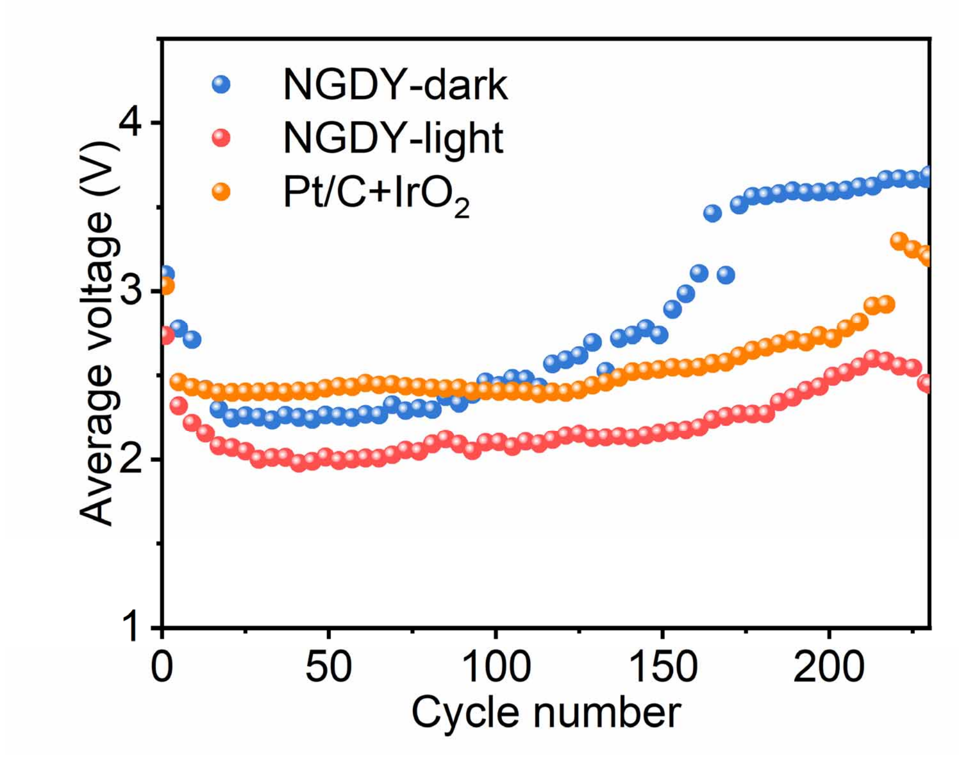

Supplementary Fig. 30. The average charging voltage of PZAB based NGDY and commercial Pt/C+IrO_2 over long-term cycling at 100 mA cm^{-2}.

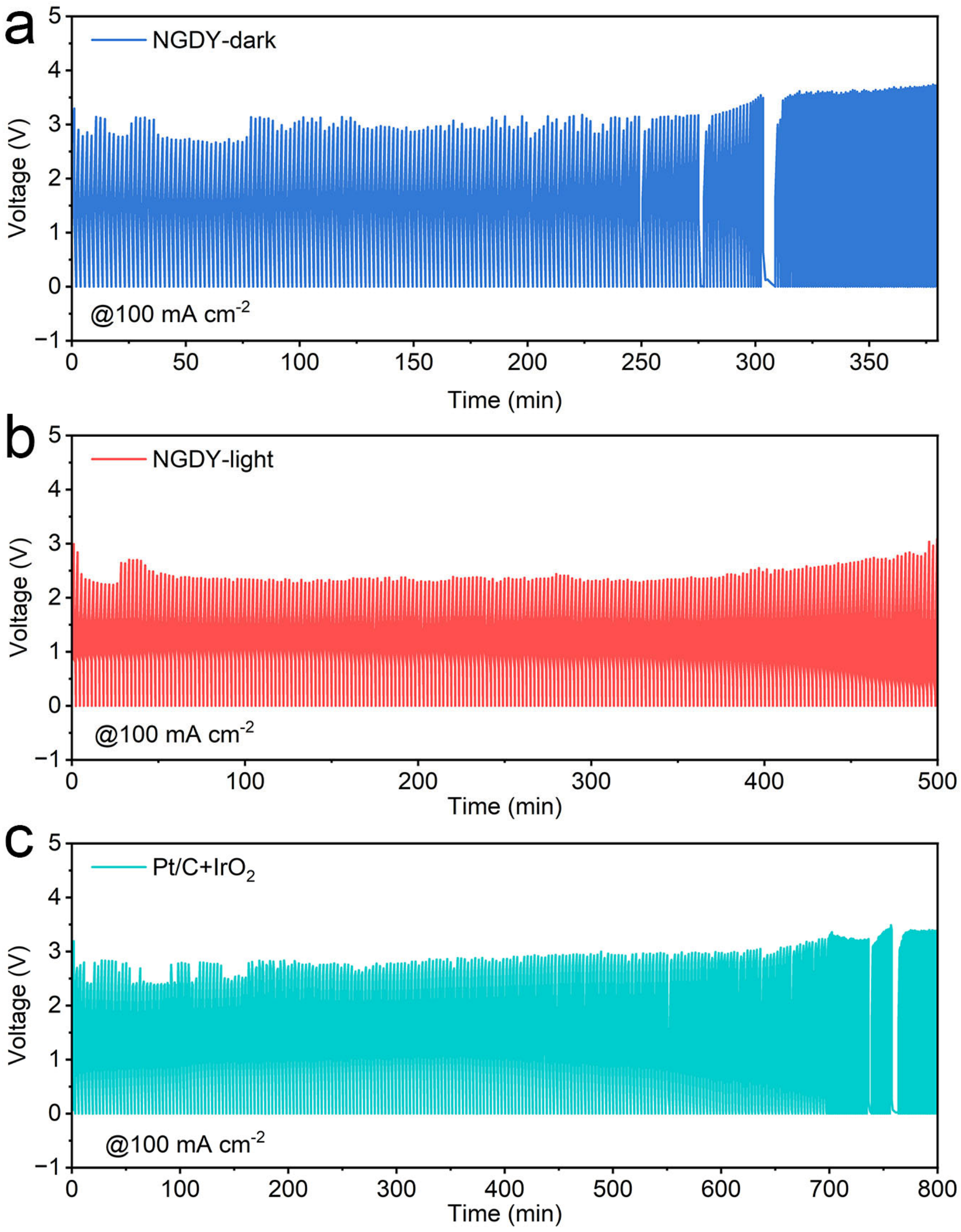

Supplementary Fig. 31. The cycling curves of PZAB based on (a and b) NGDY and (c) commercial Pt/C+IrO_2 at 100 mA cm^{-2}.

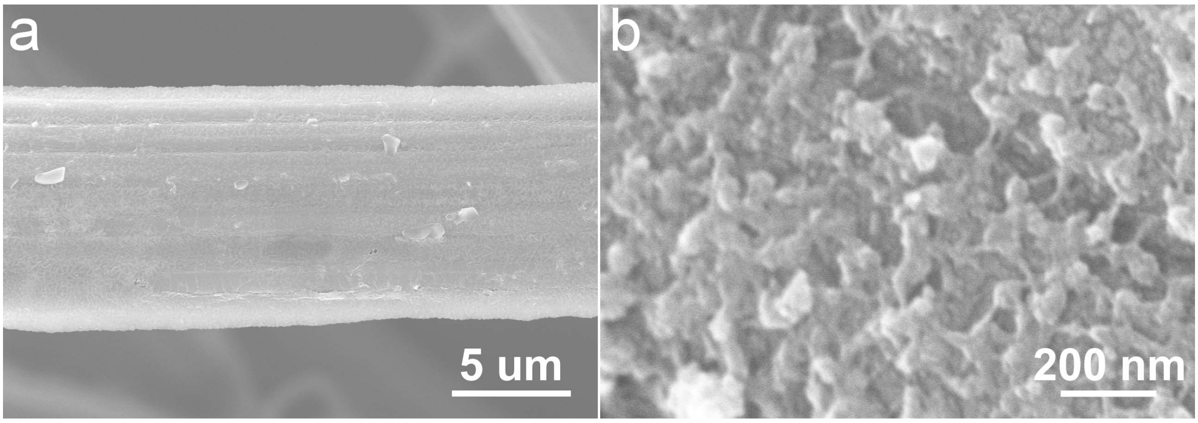

Supplementary Fig. 32. SEM images of NGDY/CC after cycling test (20 mA cm^{-2} after 180 cycles under light irradiation).

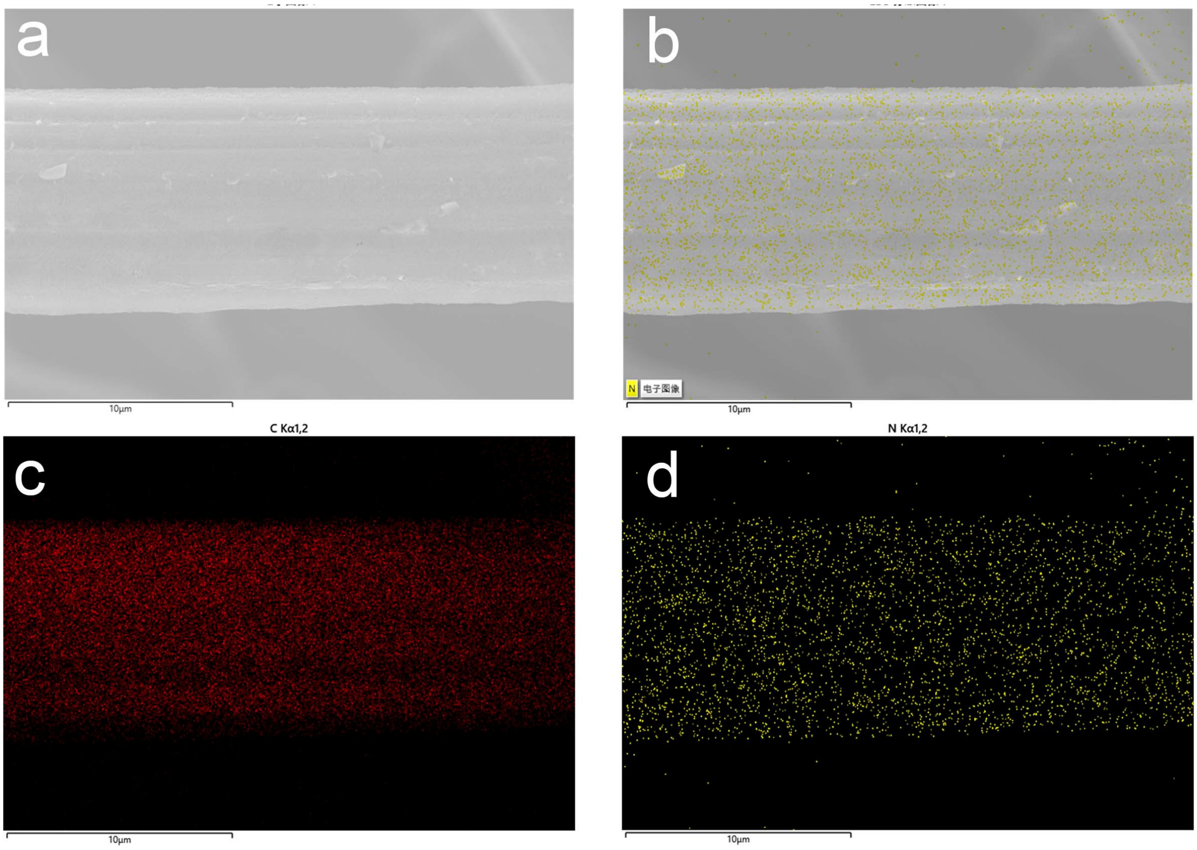

Supplementary Fig. 33. Elemental mapping of NGDY/CC after cycling test (20 mA cm^{-2} after 180 cycles under light irradiation).

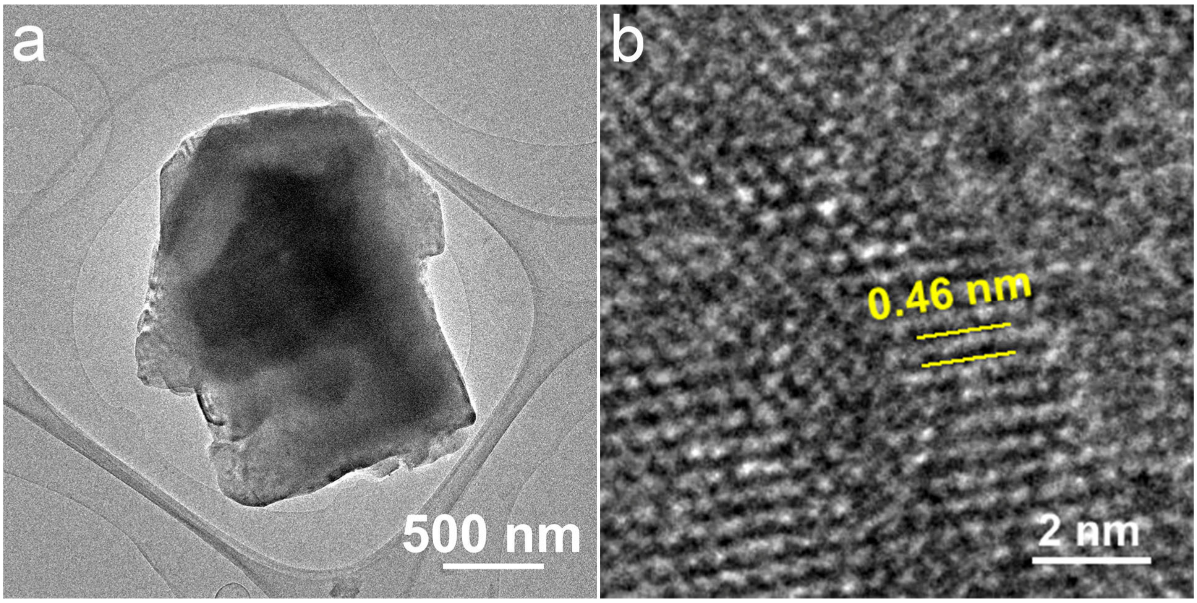

Supplementary Fig. 34. TEM images of NGDY after battery cycling tests (20 mA cm^{-2} after 180 cycles under light irradiation).

The structural stability was evaluated by SEM, EDS, TEM and XPS. After the long cycle, the morphology of NGDY remains intact without any apparent degradation or aggregation (Supplementary Fig. 32). Moreover, the EDS mapping shows that the elements C and N are still evenly distributed (Supplementary Fig. 33), indicating the structural stability of NGDY. Most pertinently, we performed post-cycling TEM/HRTEM analysis (Supplementary Fig. 34). The images clearly show that the layered crystalline structure of NGDY is retained. The measured lattice fringe spacing is 0.46 nm, and the preservation of clear lattice fringes confirms the robustness of the long-range ordered structure.

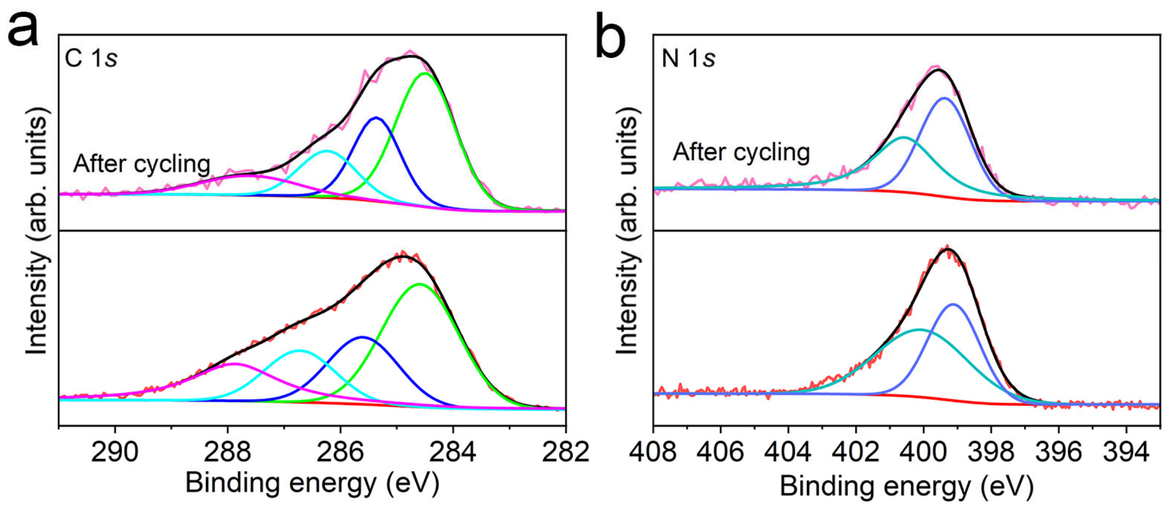

Supplementary Fig. 35. XPS (a) C 1*s* spectra and (b) N 1*s* spectra of NGDY before after battery cycling tests (20 mA cm^{-2} after 180 cycles under light irradiation).

XPS was performed before and after the battery cycling tests (Supplementary Fig. 35). The C 1*s* spectra demonstrate that the core chemical structure of the NGDY framework was not disrupted. The primary change observed was a slight change in N protonation, which suggests a benign surface adaptation to the electrochemical environment. Moreover, the atomic concentration of oxygen remained highly stable, with only minimal variations, and affirming the absence of significant oxidation or corrosion. The combined evidence from morphology, elemental distribution, and chemical bonding analysis unequivocally demonstrates the structural stability of the NGDY photo-coupled electrode under realistic working conditions.

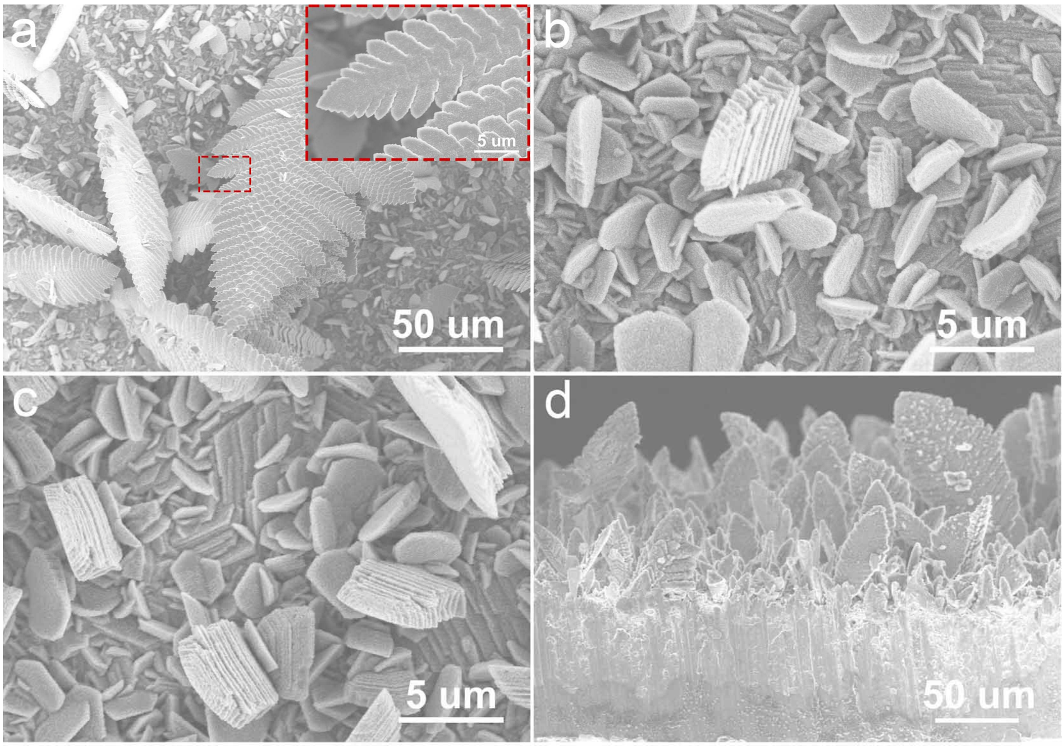

Supplementary Fig. 36. Top view (a-c) and side view (d) of SEM images of deposited Zn on Cu foil after charging at 100 mA cm^{-2} in the dark (deposited time: 10 min, deposited capacity: 16.67 mA h cm^{-2}).

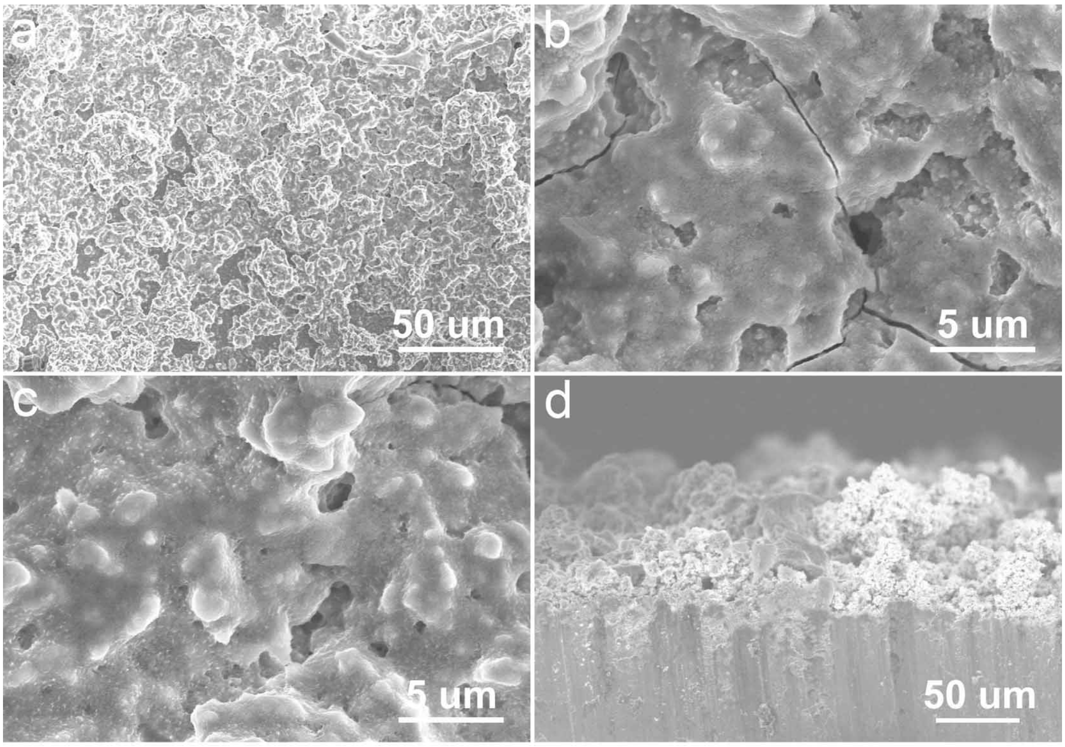

Supplementary Fig. 37. Top view (a-c) and side view (d) of SEM images of deposited Zn on Cu foil after charging at 100 mA cm^{-2} under light irradiation (deposited time: 10 min, deposited capacity: 16.67 mA h cm^{-2}).

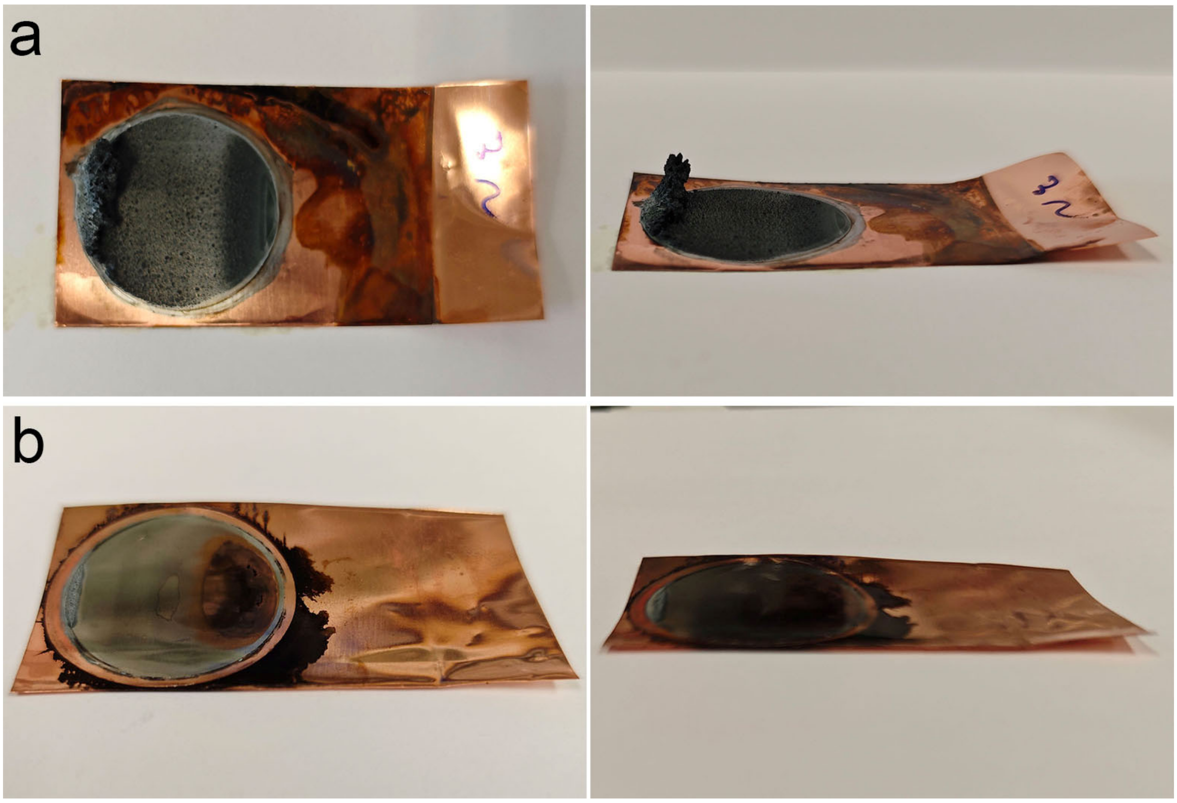

Supplementary Fig. 38. Optical images of Cu foil after long-term cycling test (230 cycles) at 100 mA cm^{-2} (a) in the dark and (b) under light irradiation.

After long-term cycling test at 100 mA cm^{-2} in the dark, visible zinc particles, tens of micrometers in diameter and easy to fall off, were deposited onto the copper foil, which can be confirmed by SEM images (Supplementary Fig. 36). Such nonuniform deposition resulted in the change of electrode shape and the detachment of large Zn dendrites and formation of "dead Zn", which is harmful to battery performance and causing irreparable capacity losses and battery short circuits, and finally the battery test was terminated (Supplementary Fig. 38).

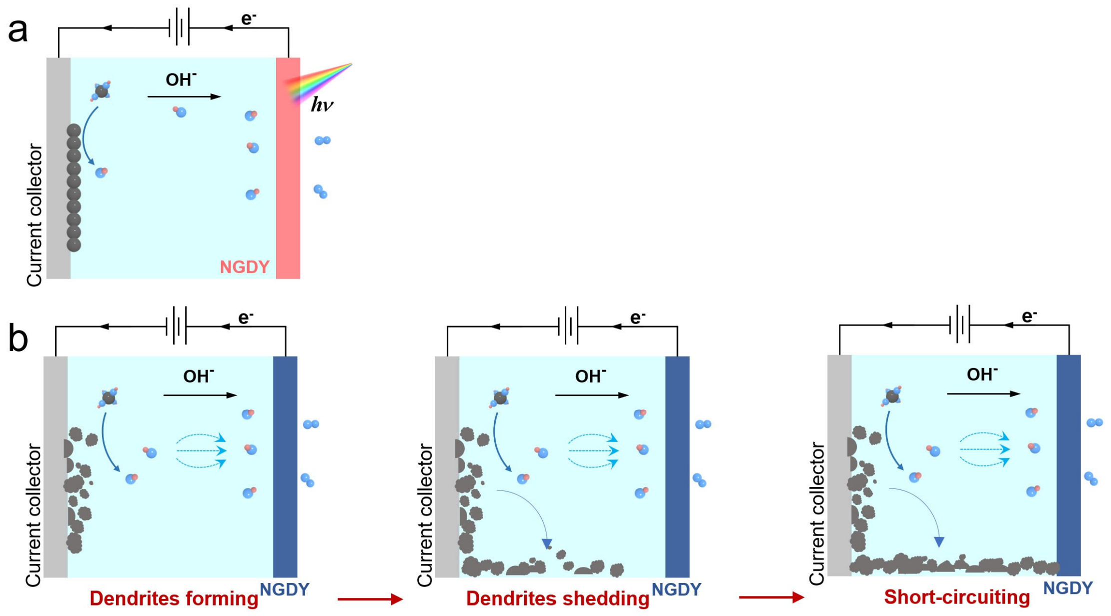

Supplementary Fig. 39. Schematic illustration of deposition mechanism of zinc during charging (a) under light irradiation and (b) in the dark.

1. Under conditions of higher overpotential (such as with NGDY in the dark or with the Pt/C+IrO_2 catalyst), zinc tends to deposit as dendrites.
2. These dendrites are mechanically unstable and can detach from the current collector (as illustrated in the provided schematic, stage "Dendrites shedding"). Once detached, the electrical connection is lost, leading to an immediate termination of that discharge phase (discharge time ~0, CE ~0%). This corresponds to the sharp dips in the discharge curve.
3. Subsequently, the detached dendritic particles may physically re-contact the electrode, leading to a renewed, sometimes prolonged, discharge period.
4. With continued cycling, accumulated detached dendrites can eventually bridge the electrodes, causing a short circuit and test termination.

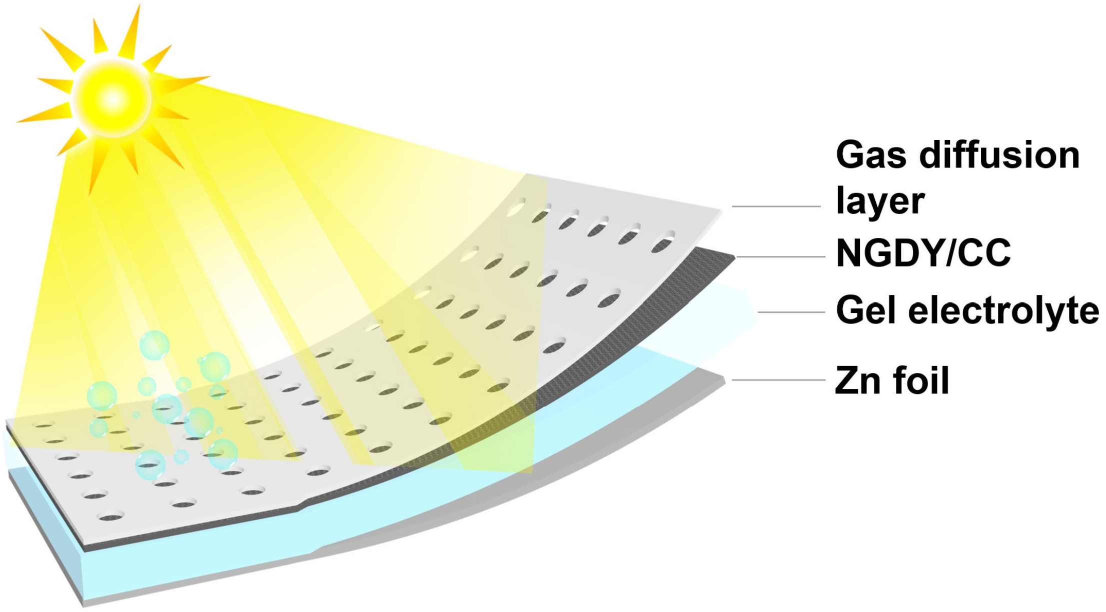

Supplementary Fig. 40. Schematic illustration of the structure of NGDY based solid state PZAB.

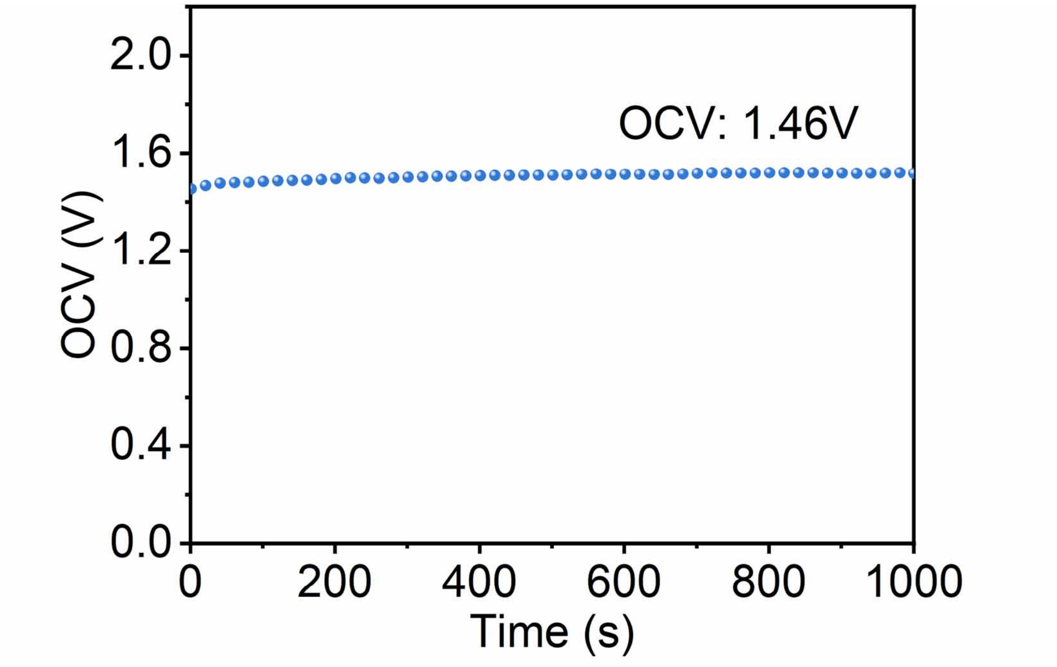

Supplementary Fig. 41. The OCV curves of NGDY based solid state PZAB.

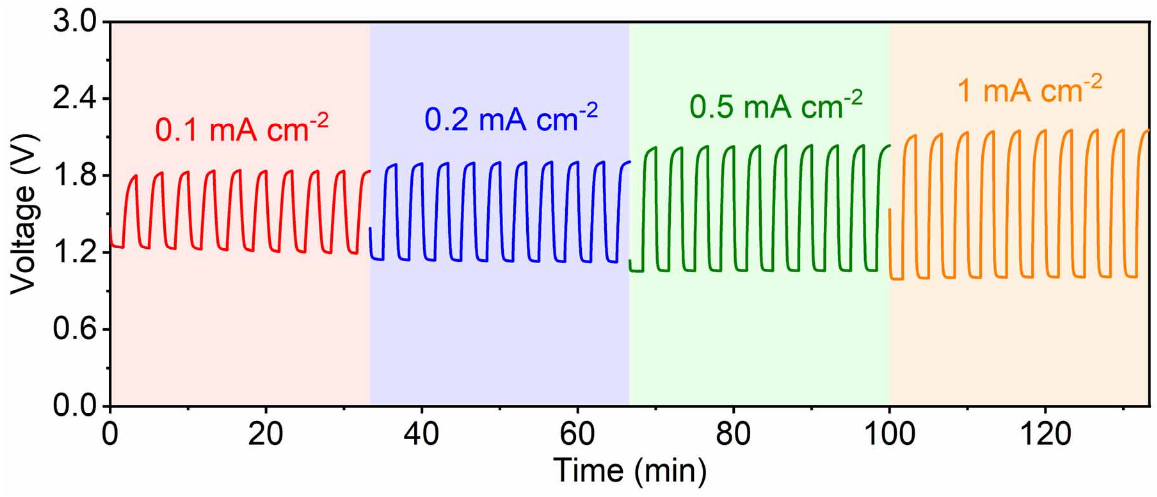

Supplementary Fig. 42. Cycling performance of NGDY based solid state PZAB at various current density.

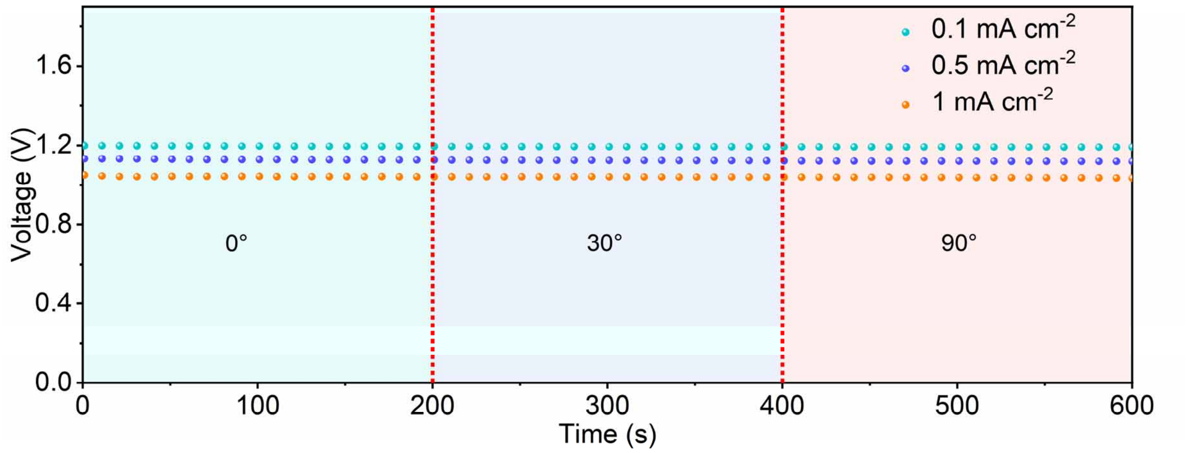

Supplementary Fig. 43. Galvanostatic discharge curves at current density form 0.1 mA cm^{-2} to 1mA cm^{-2} of NGDY based PZAB under different bending conditions.

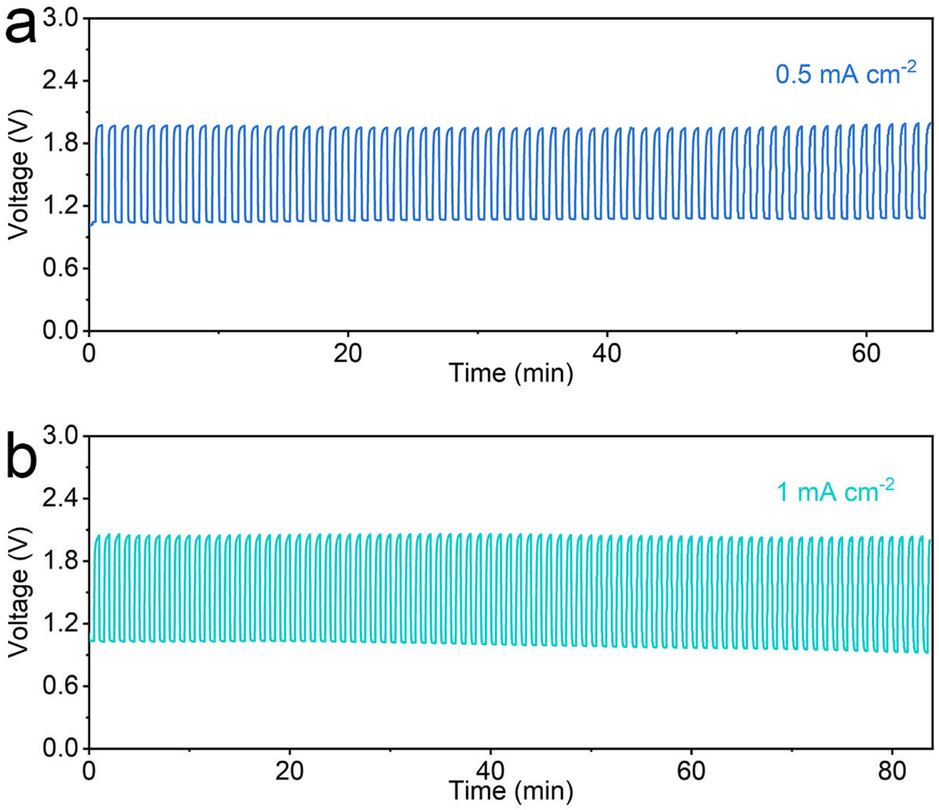

Supplementary Fig. 44. Galvanostatic discharge–charge cycling curves at (a) 0.5 and (b) 1 mA cm^{-2}.

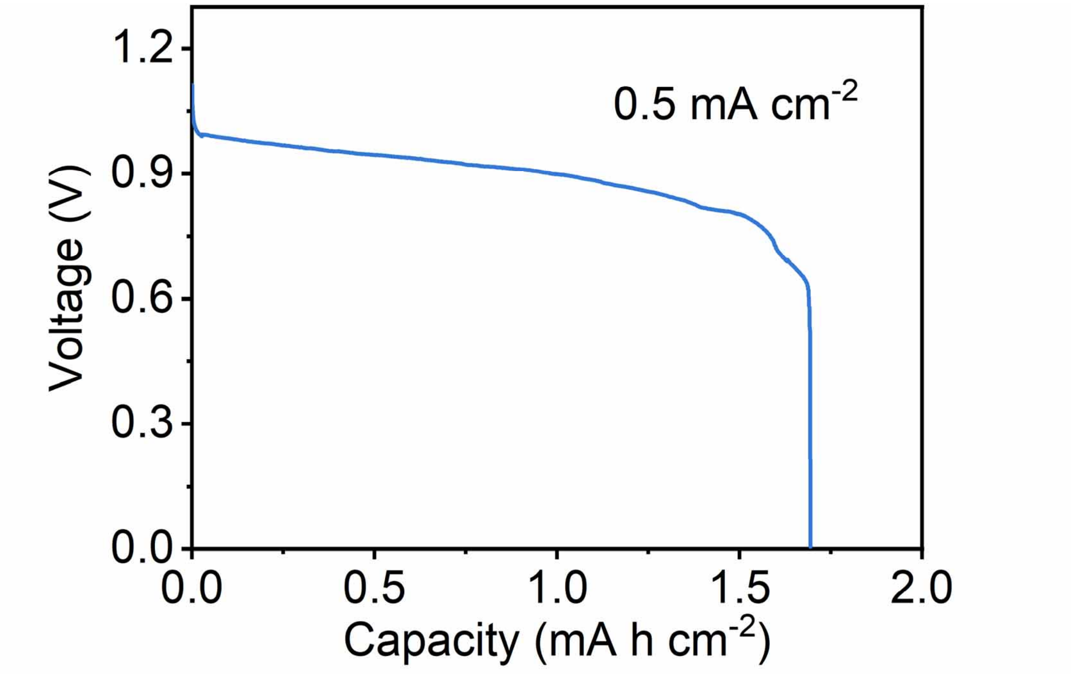

Supplementary Fig. 45. Energy density of NGDY based solid state PZAB at 0.5 mA cm^{-2}.

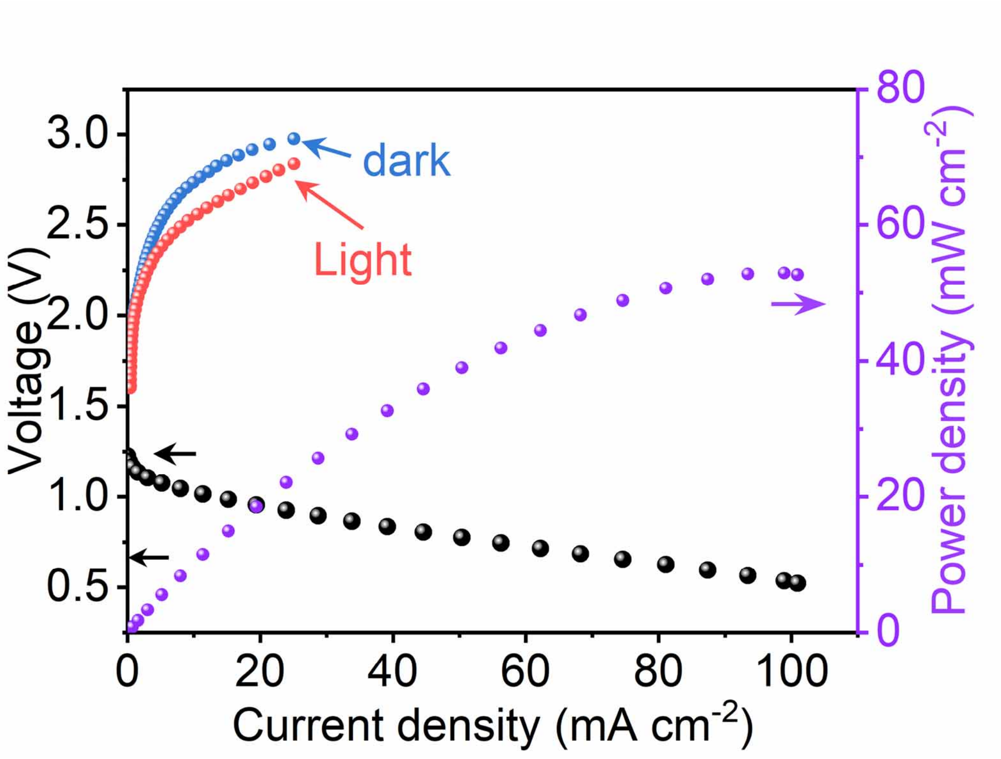

Supplementary Fig. 46. Polarization curves and corresponding power density curve of solid state PZAB assembled with NGDY.

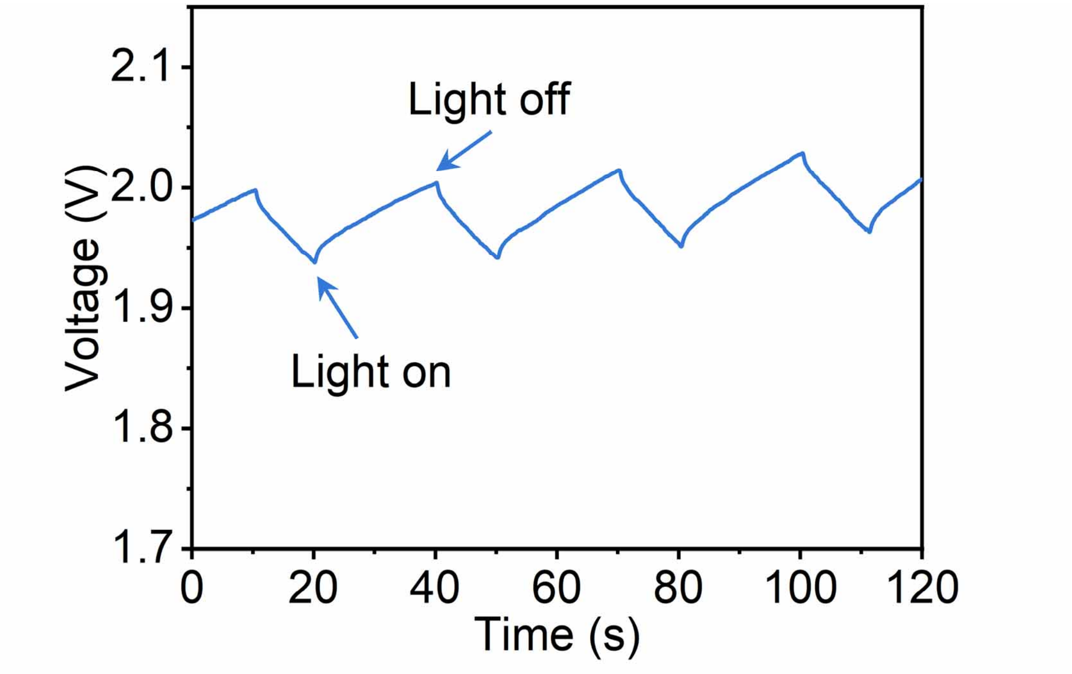

Supplementary Fig. 47. Charge curve of the solid-state PZAB under intermittent light irradiation.

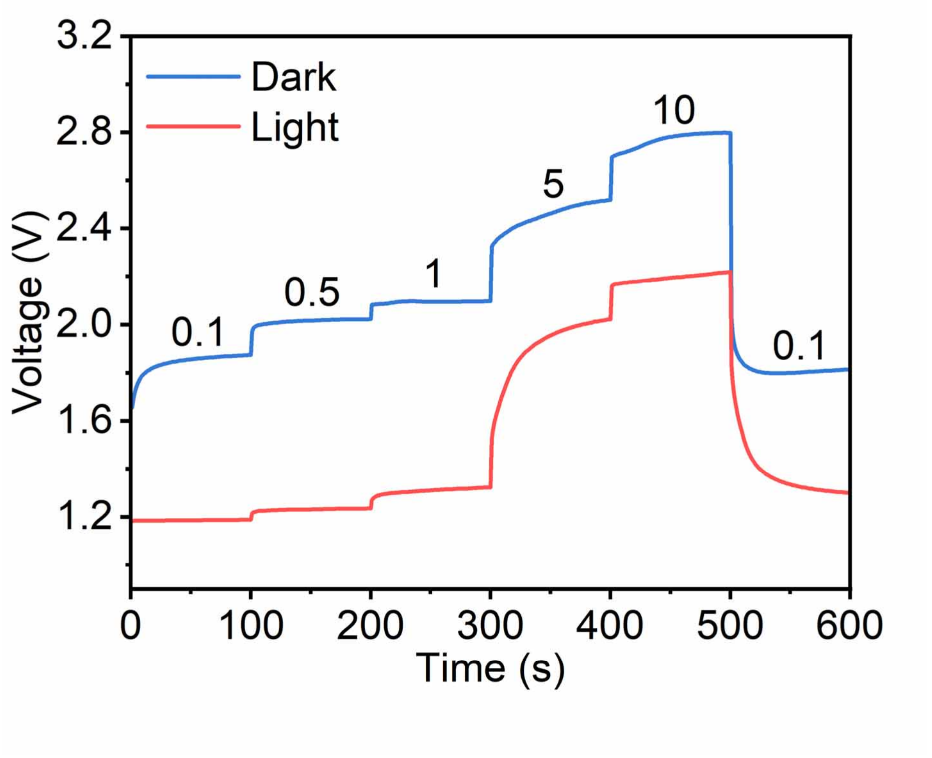

Supplementary Fig. 48. Charge curves of NGDY-based solid PZAB at current densities from 0.5 mA cm^{-2} to 10 mA cm^{-2}.

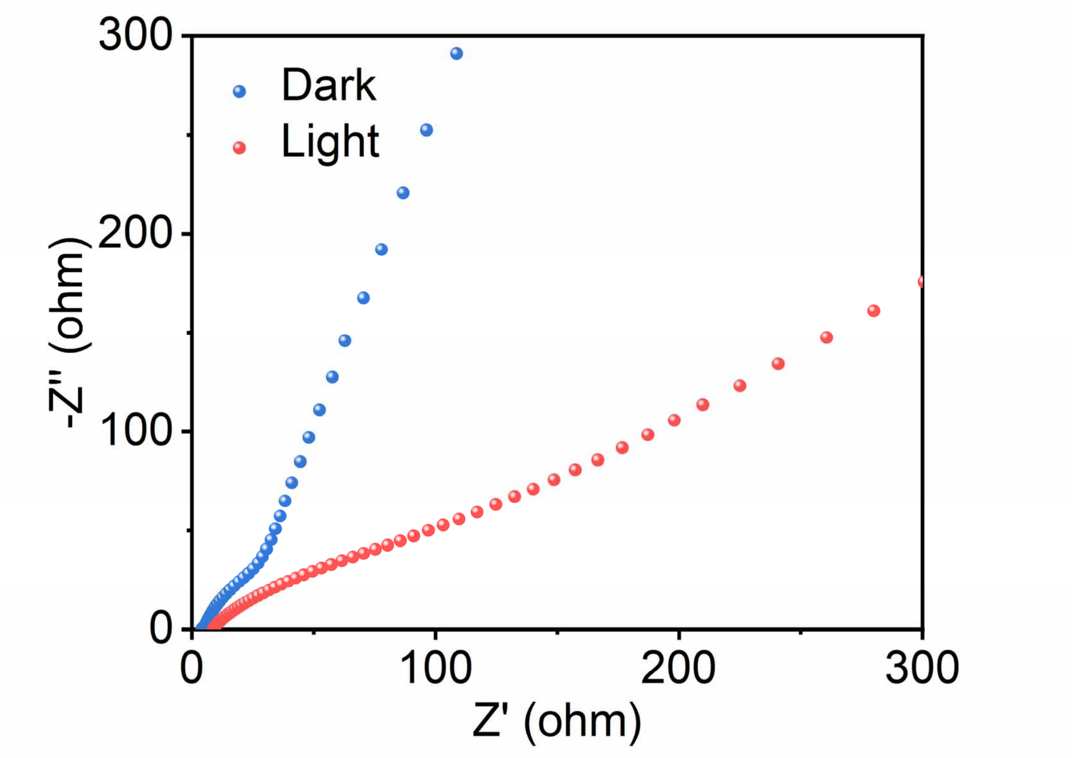

Supplementary Fig. 49. Electrochemical impedance spectroscopy of NGDY based solid Zn||air battery in the dark and illumination, respectively.

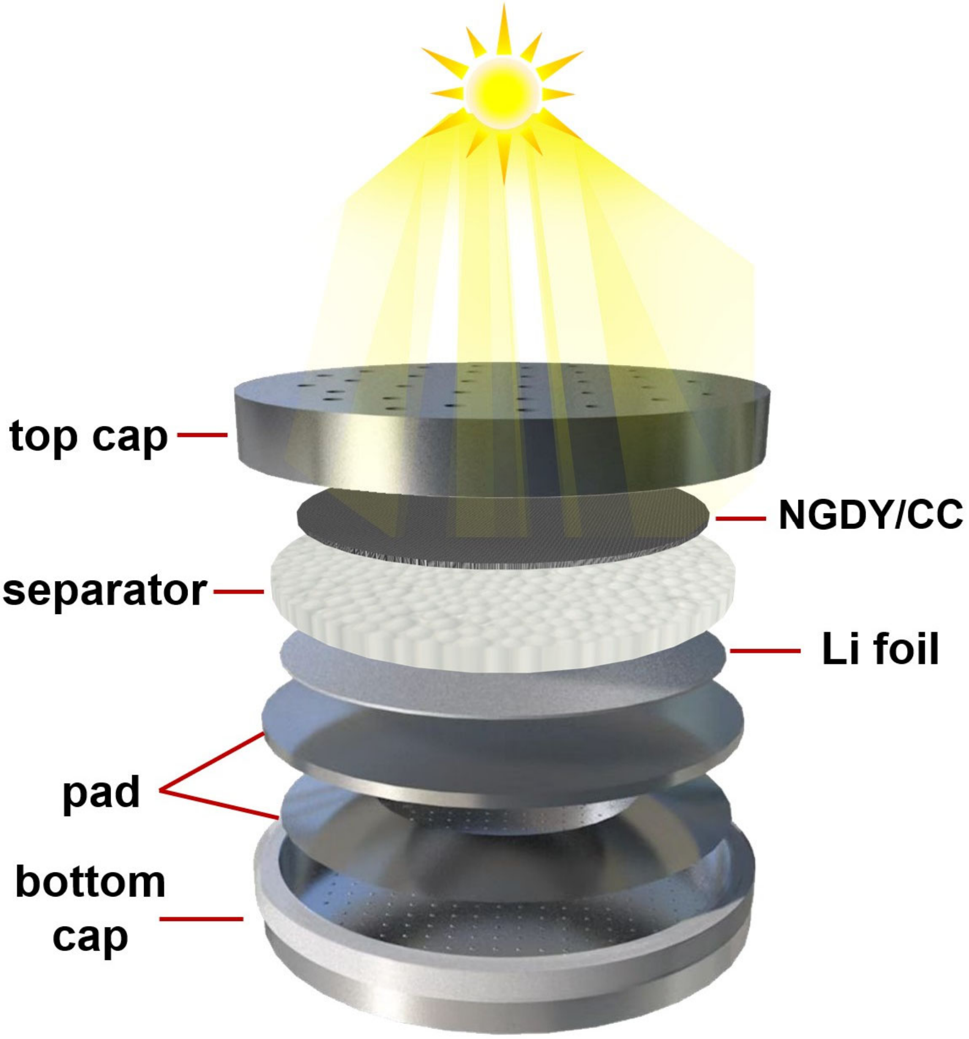

Supplementary Fig. 50. Schematic illustration of the structure of NGDY-based PLOB.

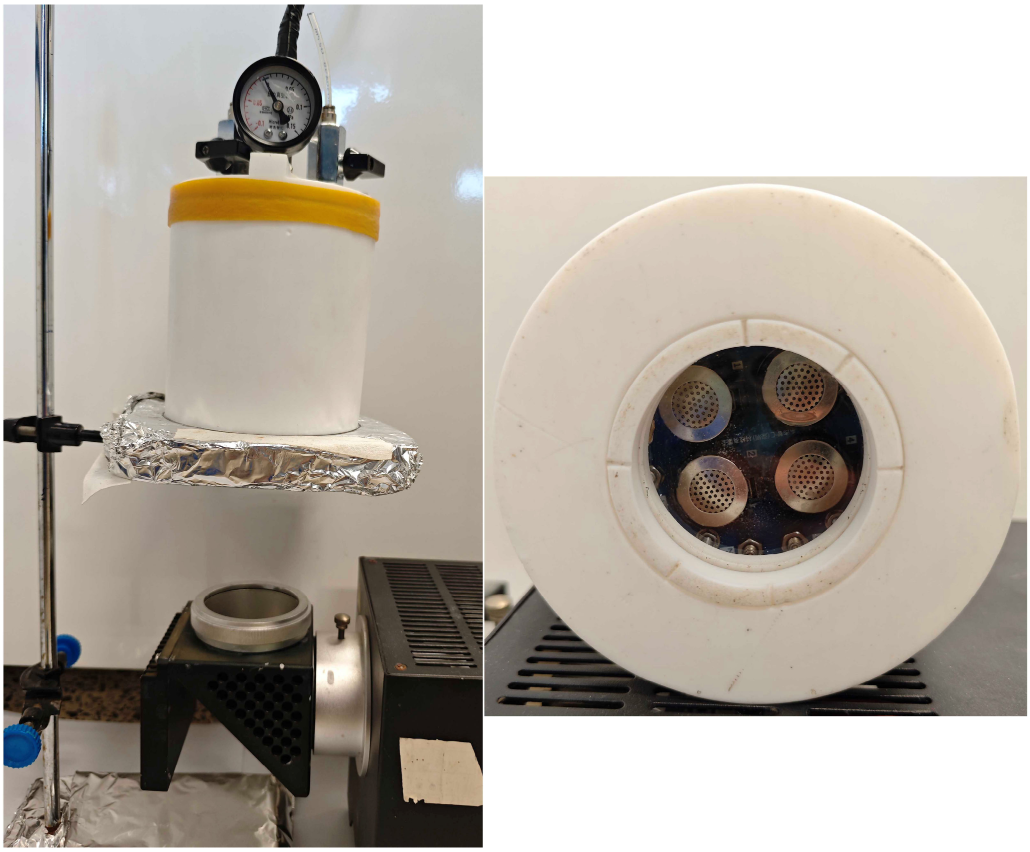

Supplementary Fig. 51. The optical photograph of the working device of PLOB.

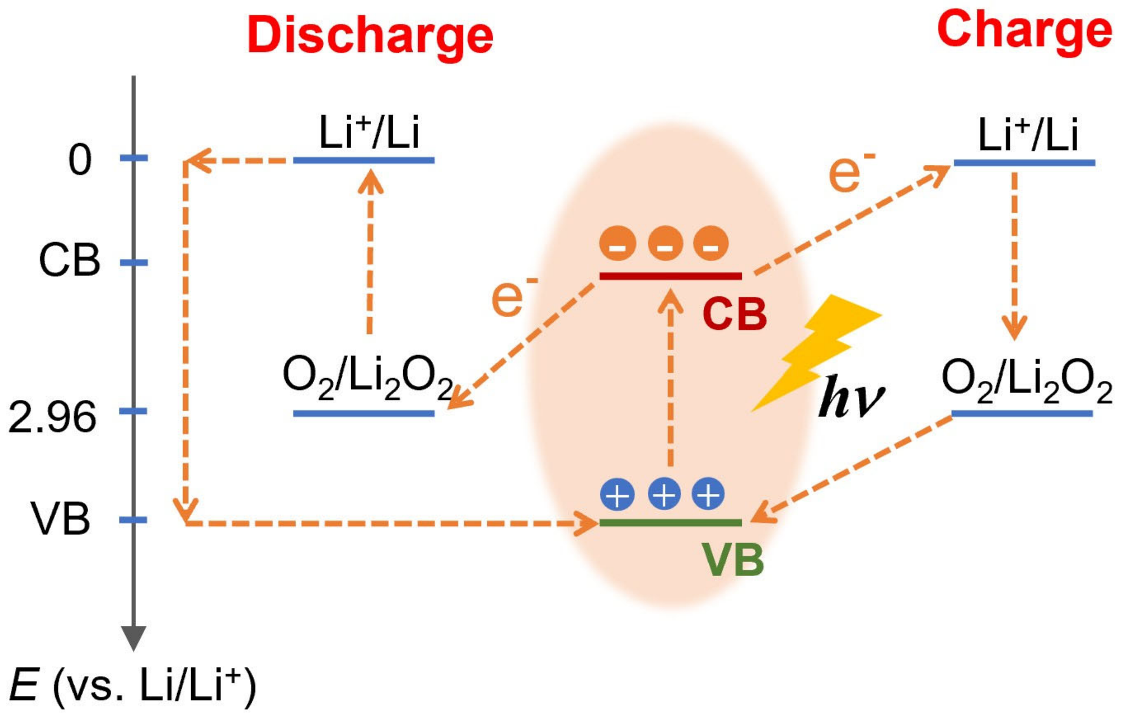

Supplementary Fig. 52. The proposed operating mechanism of PLOB based on NGDY.

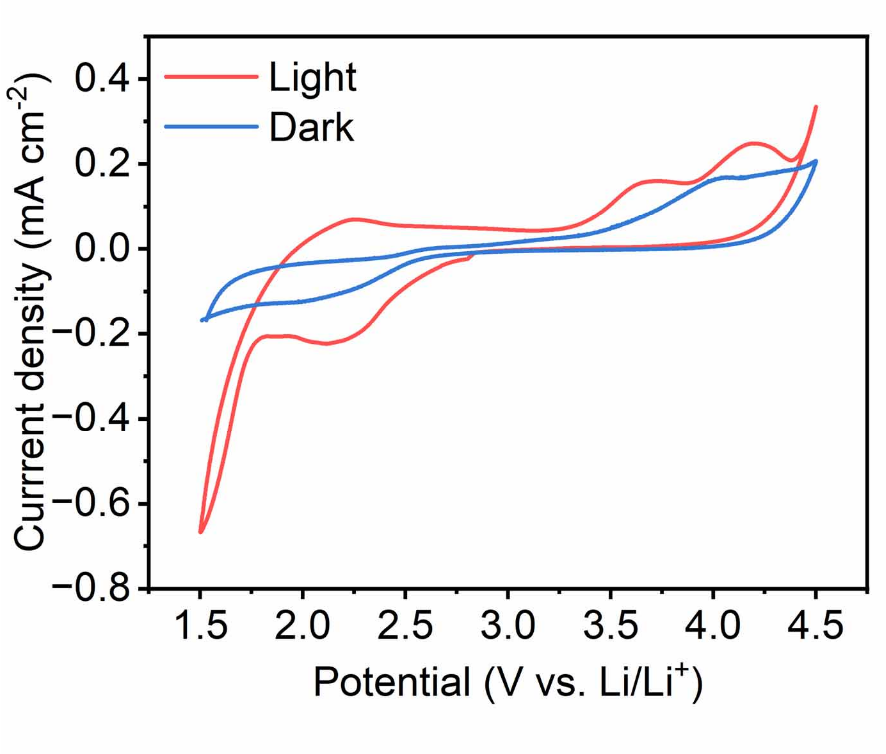

Supplementary Fig. 53. CV curves of PLOB based on NGDY with and without light irradiation.

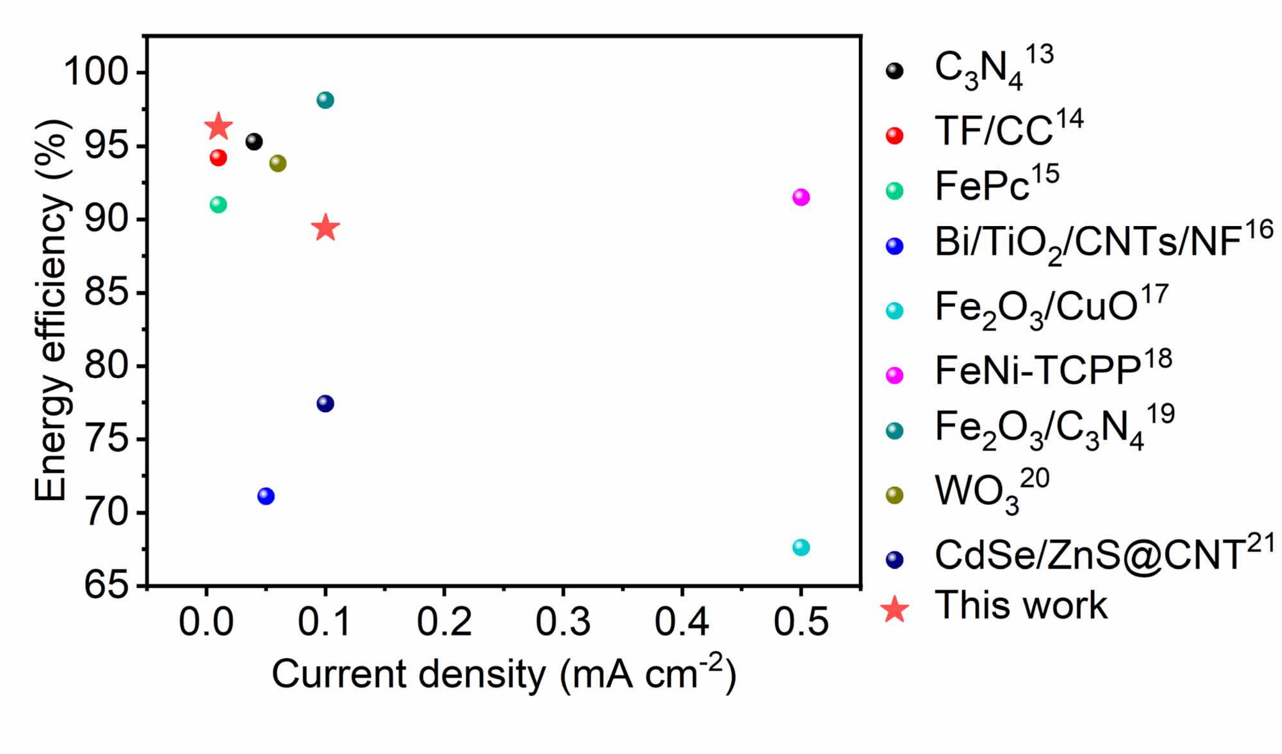

Supplementary Fig. 54. Comparation of energy efficiency and current density of NGDY based PLOB and other reported electrocatalysts based photo-coupled Li||O_2 battery systems.[13,14,15,16,17,18,19,20,21] The source of the literature data shown in this figure can be found in Supplementary Table 9.

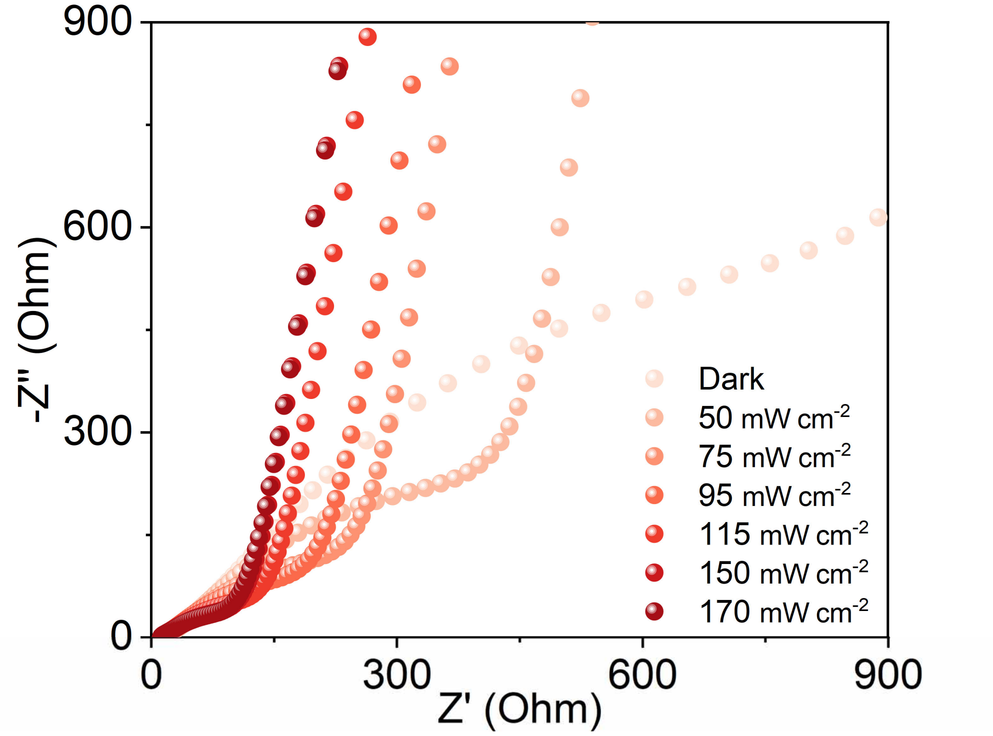

Supplementary Fig. 55. EIS of PLOB under different light intensity.

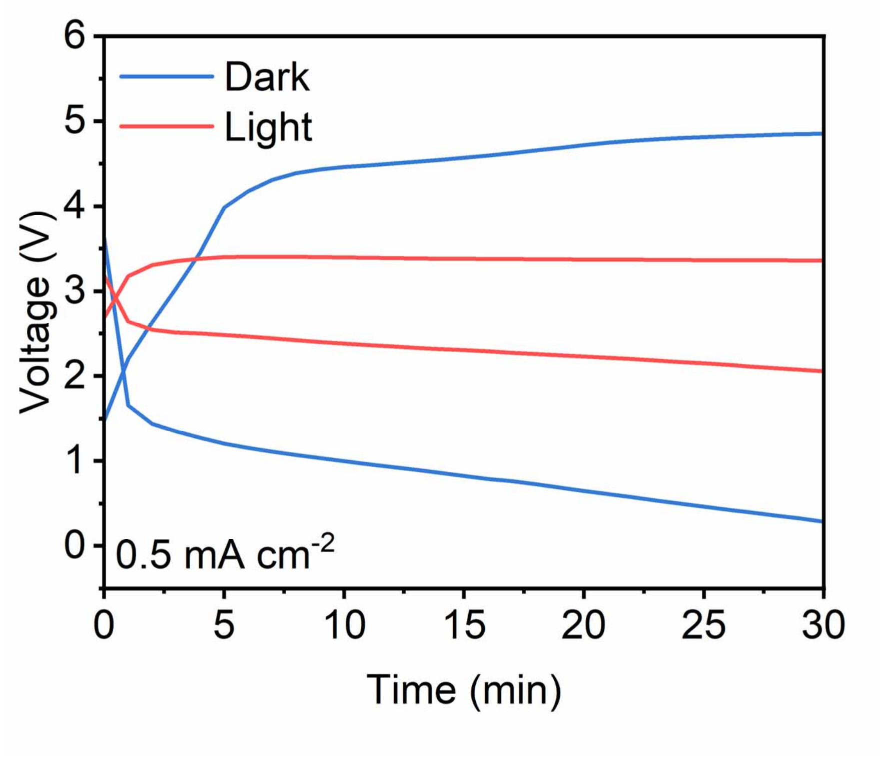

Supplementary Fig. 56. Discharge and charge profiles of the PLOB battery at 0.5 mA cm^{-2}.

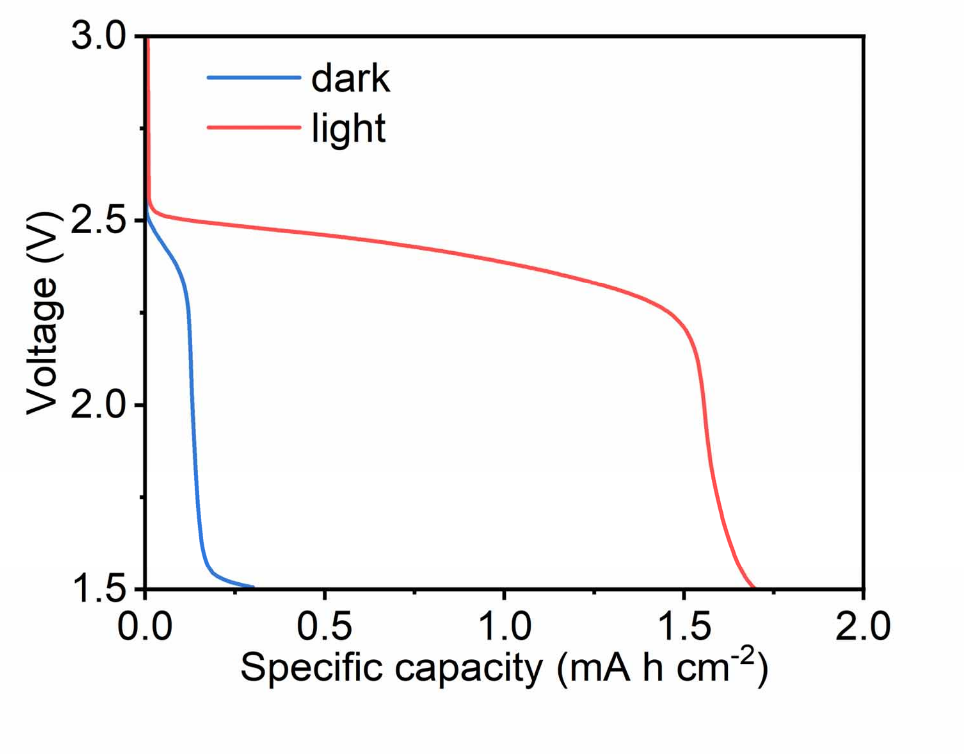

Supplementary Fig. 57. Discharge curves of NGDY at the current density of 0.2 mA cm^{-2} in the dark and under light irradiation.

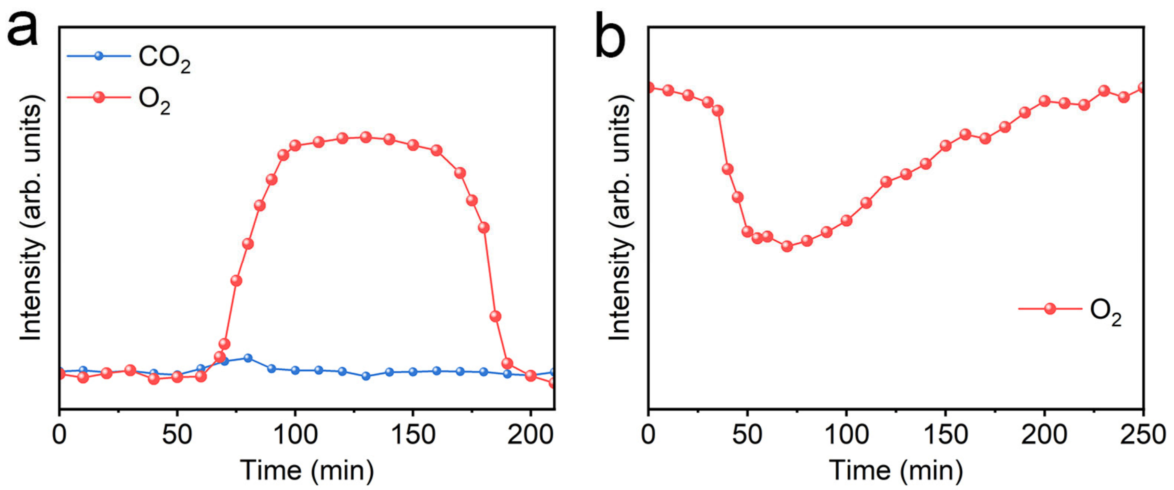

Supplementary Fig.58. DEMS analyses in the PLOB based on NGDY in discharge (a) and charge (b) processes.

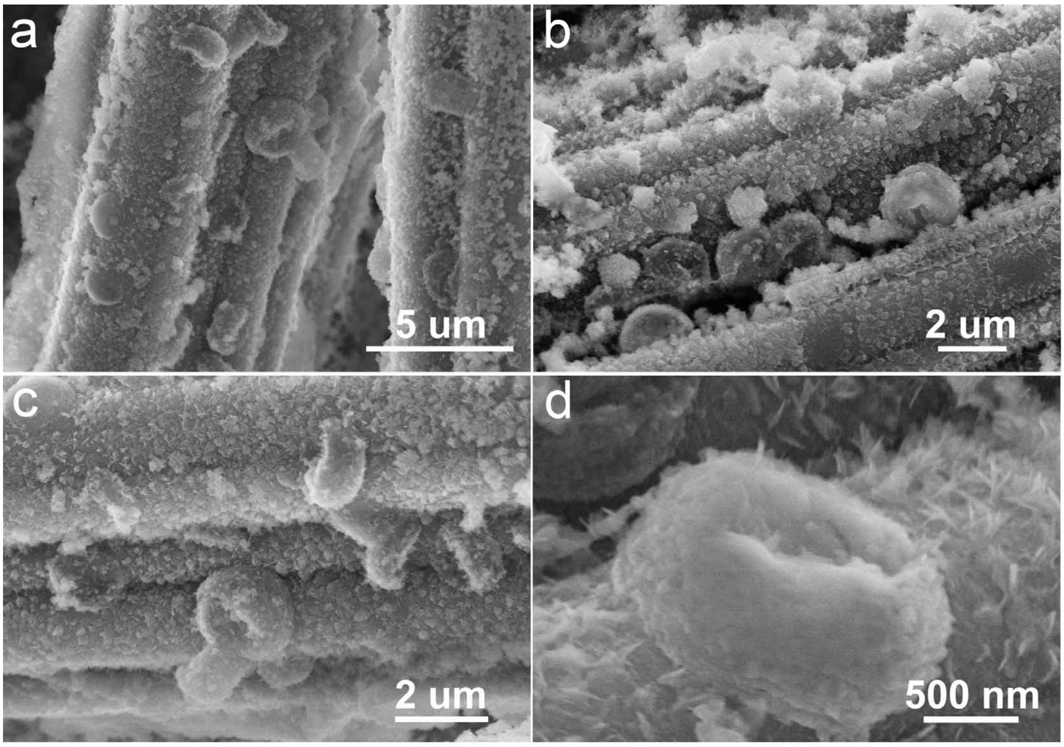

Supplementary Fig. 59. SEM images of the positive electrode of Li||O_2 battery after discharging based on CC (Current density: 0.1 mA cm^{-2}, discharging capacity: 0.5 mA h cm^{-2}).

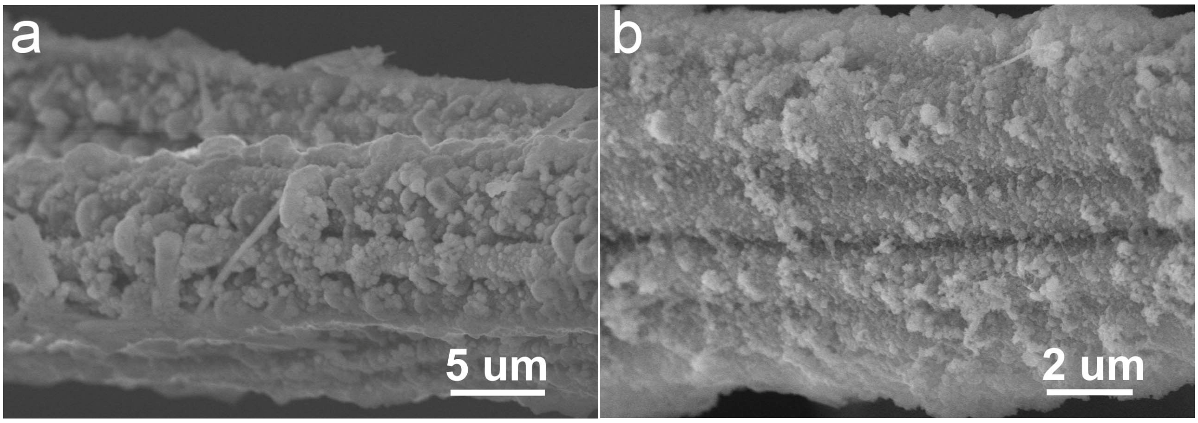

Supplementary Fig. 60. SEM images of the positive electrode of Li||O_2 battery after discharging in the dark based on NGDY/CC (Current density: 0.1 mA cm^{-2}, discharging capacity: 0.5 mA h cm^{-2}).

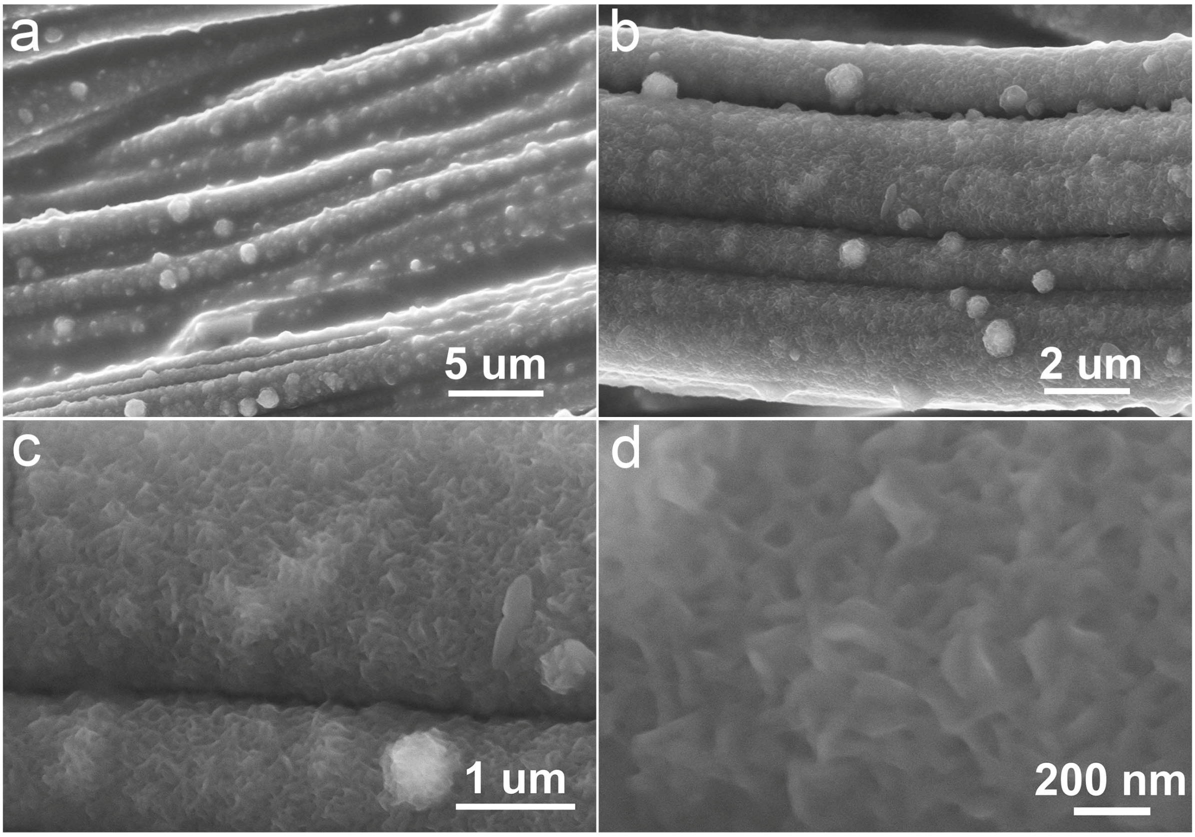

Supplementary Fig. 61. SEM images of the positive electrode of Li||O_2 battery after discharging under light irradiation based on NGDY/CC (Current density: 0.1 mA cm^{-2}, discharging capacity: 0.5 mA h cm^{-2}).

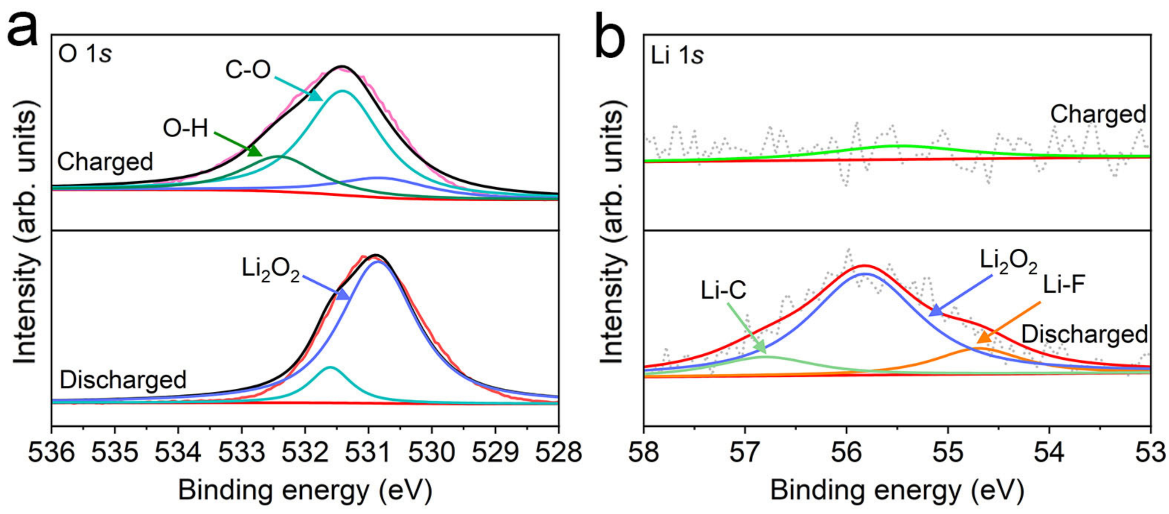

Supplementary Fig. 62. XPS (a) O 1*s* spectrum and (b) Li 1*s* spectrum of charged and discharged NGDY (Current density: 0.1 mA cm^{-2}, discharging capacity: 0.5 mA h cm^{-2}).

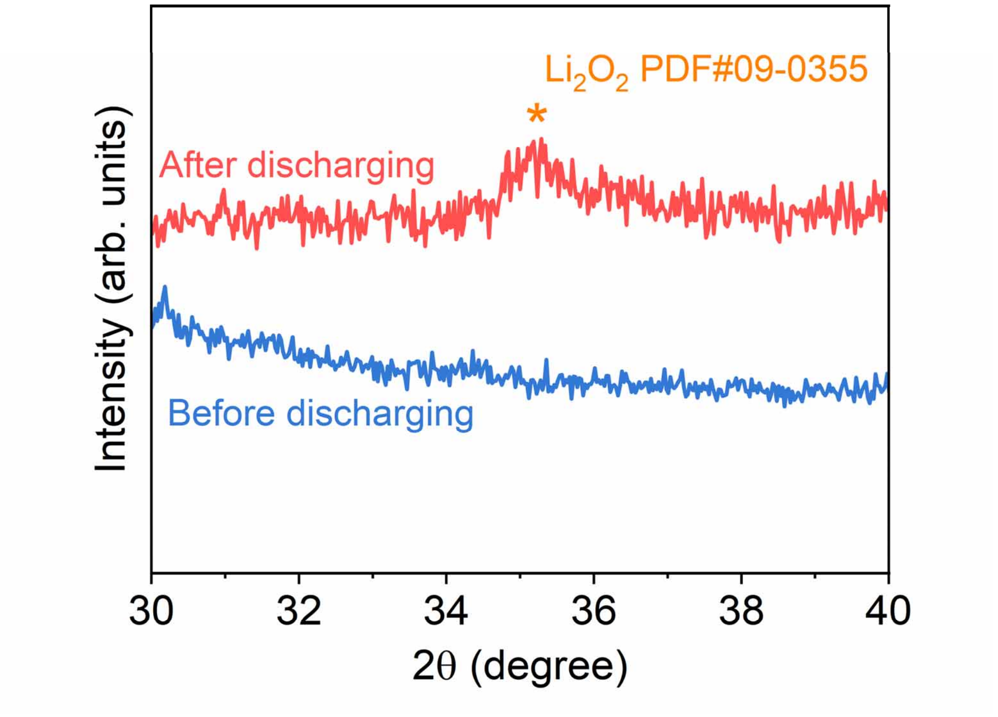

Supplementary Fig. 63. XRD spectrum of NGDY before discharging and after discharging (Current density: 0.1 mA cm^{-2}, discharging capacity: 0.5 mA h cm^{-2}).

We have performed additional XPS and XRD characterizations on the NGDY positive electrodes harvested after specific states of charge/discharge (Supplementary Fig. 62 to 63). The O 1*s* XPS spectrum of the discharged positive electrode shows a distinct peak at 530.85 eV, characteristic of Li_2O_2. This peak disappears after charging, with the spectrum reverting to show the C-O peak of the NGDY substrate. Correspondingly, the Li 1*s* spectrum of the discharged electrode displays a main peak at 55.83 eV (Li_2O_2), alongside minor peaks for Li–C and Li–F species. After charging, the Li 1*s* signal diminishes significantly, indicating the removal of lithium species from the positive electrode. Furthermore, ex situ XRD analysis of the discharged positive electrode reveals a new diffraction peak at approximately 35°, which is indexed to the (010) crystal plane of Li_2O_2. This peak is absent in the pristine and charged electrodes. Collectively, these results from XPS and XRD offer direct and complementary evidence that the primary discharge product is Li_2O_2.

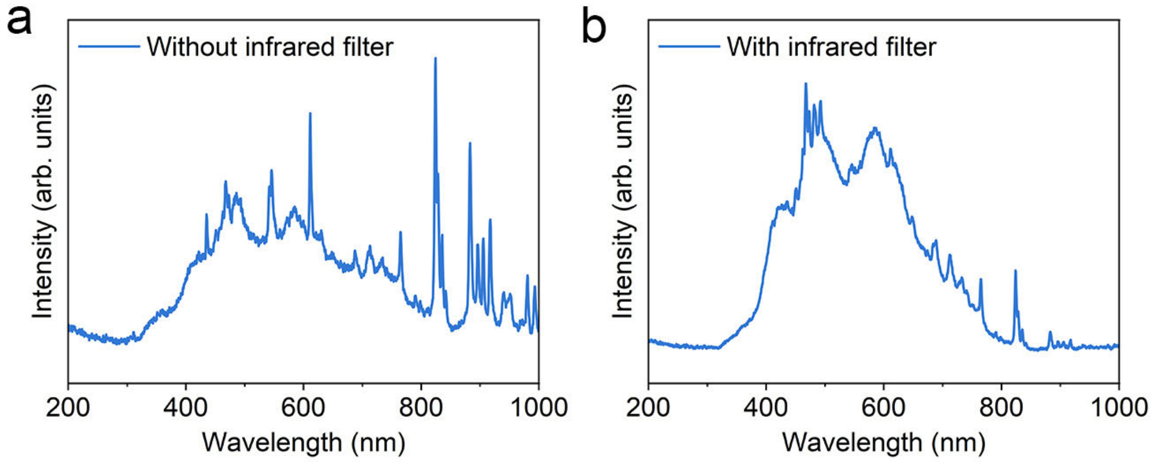

Supplementary Fig. 64. The output spectrum of the xenon light source (a) without infrared filter and (b) with infrared filter.

In all photo-coupled battery tests, an 800 nm infrared filter was employed. As illustrated in the Supplementary Fig. 64, this filter drastically attenuates the intense infrared emission band (800-1000 nm) of the xenon lamp, the primary source of radiative heating.

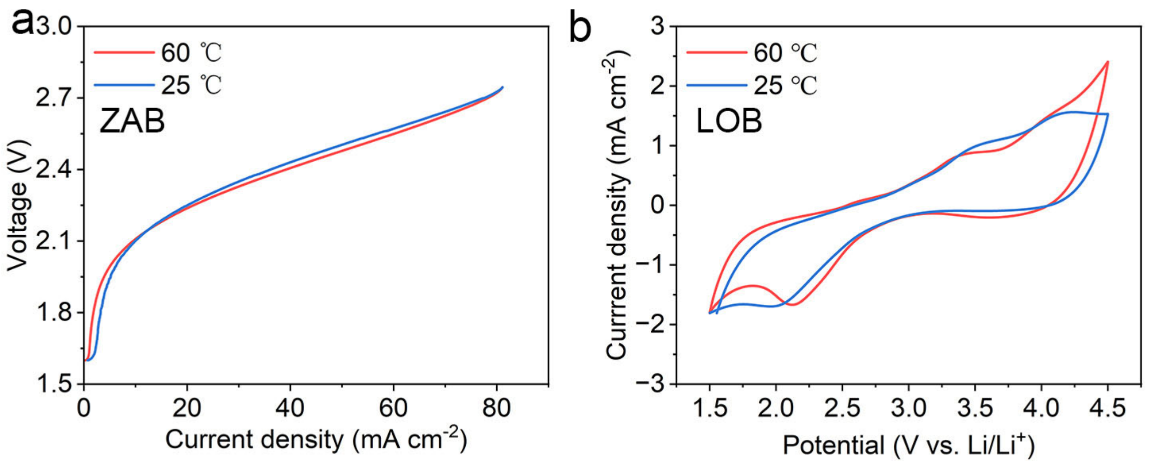

Supplementary Fig. 65. (a) Charge polarization curves of PZAB based on NGDY at 25°C and 60°C. (b) CV curves of PLOB based on NGDY at 25°C and 60°C.

We conducted a direct comparison by externally heating the battery in the dark. While elevated temperature can generally lower activation barriers and accelerate ion transport, it also risks promoting detrimental interfacial side reactions. In our system, the active cooling and electrolyte circulation maintained the operational temperature near 25°C during all light tests, ensuring that any bulk heating was not a variable.

To isolate the effect of temperature, we evaluated the Zn||air and Li||O_2 battery performance under dark conditions at controlled temperatures of 25°C and 60°C. For the Zn||air battery, external heating to 60°C induced only a modest improvement in the charging curve (Supplementary Fig. 65a). For the Li||O_2 battery, CV curves at 60°C show a slight increase in current density but no fundamental change in the reaction profile compared to 25°C (Supplementary Fig. 65b). The key finding is consistent across systems: the magnitude of improvement from heating alone is minor. It is fundamentally distinct from the dramatic reduction in charging voltage and overpotential achieved under light illumination at 25°C.

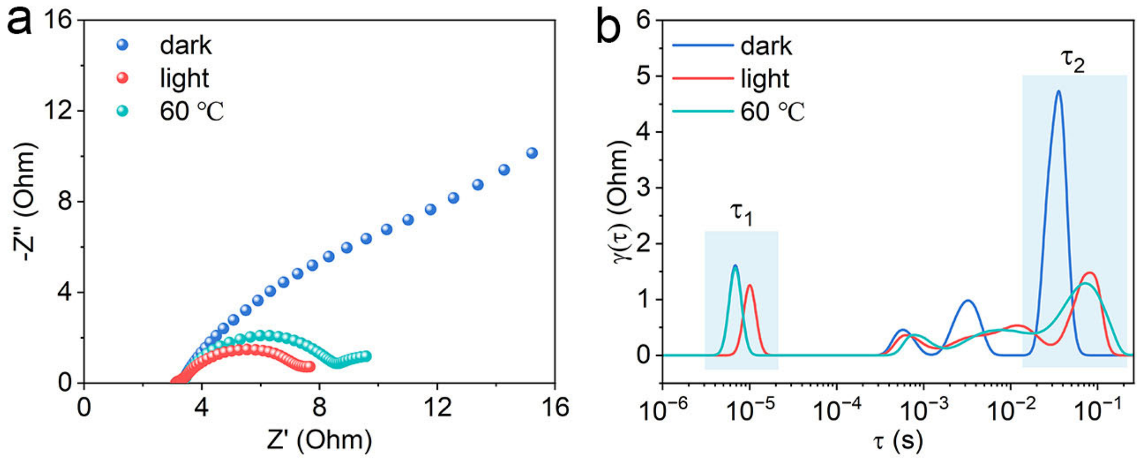

Supplementary Fig. 66. (a) EIS of PZAB in the dark, under light irradiation and at 60 °C. (b) DRT plots obtained by deconvolving the EIS.

We conducted electrochemical impedance spectroscopy (EIS) in the dark, under light irradiation and at 60 °C, and analyzed the data using distribution of relaxation time (DRT) method (Figure R4). In the DRT plot, each peak maximum represents a distinct electrochemical process contributing to the total polarization resistance of the battery, the time constant (τ) is characteristic of each polarization process, and the area under each peak corresponds to the polarization resistance contribution of that specific reaction to the total cell polarization. Therefore, changes in the DRT curves directly reflect variations in the nature and magnitude of the electrode reactions. The results reveal four main peaks in the DRT plot, corresponding to four major electrode processes or reactions. In the time constant region of 10^{-6} to 10^{-5} seconds (τ_1), which corresponds to electronic resistance, the peaks for dark at 25 °C and dark at 60 °C nearly overlap, while the peak under light is significantly reduced. This indicates that light excitation alters the electronic structure of NGDY, accelerating electron transport within the material. Heating alone at 60 °C in the dark does not produce this effect, confirming that the enhanced electronic transport is a genuine photoelectronic phenomenon, not a thermal artifact. In the time constant region of 10^{-2} to 10^{-1} seconds (τ_2), which relates to diffusion resistance, both light at 25 °C and dark at 60 °C show reduced peak areas compared to dark at 25 °C. This suggests that both light and heating enhance mass transport in the electrolyte, and the enhancement under light can be partially attributed to photothermal effects accelerating ion diffusion.

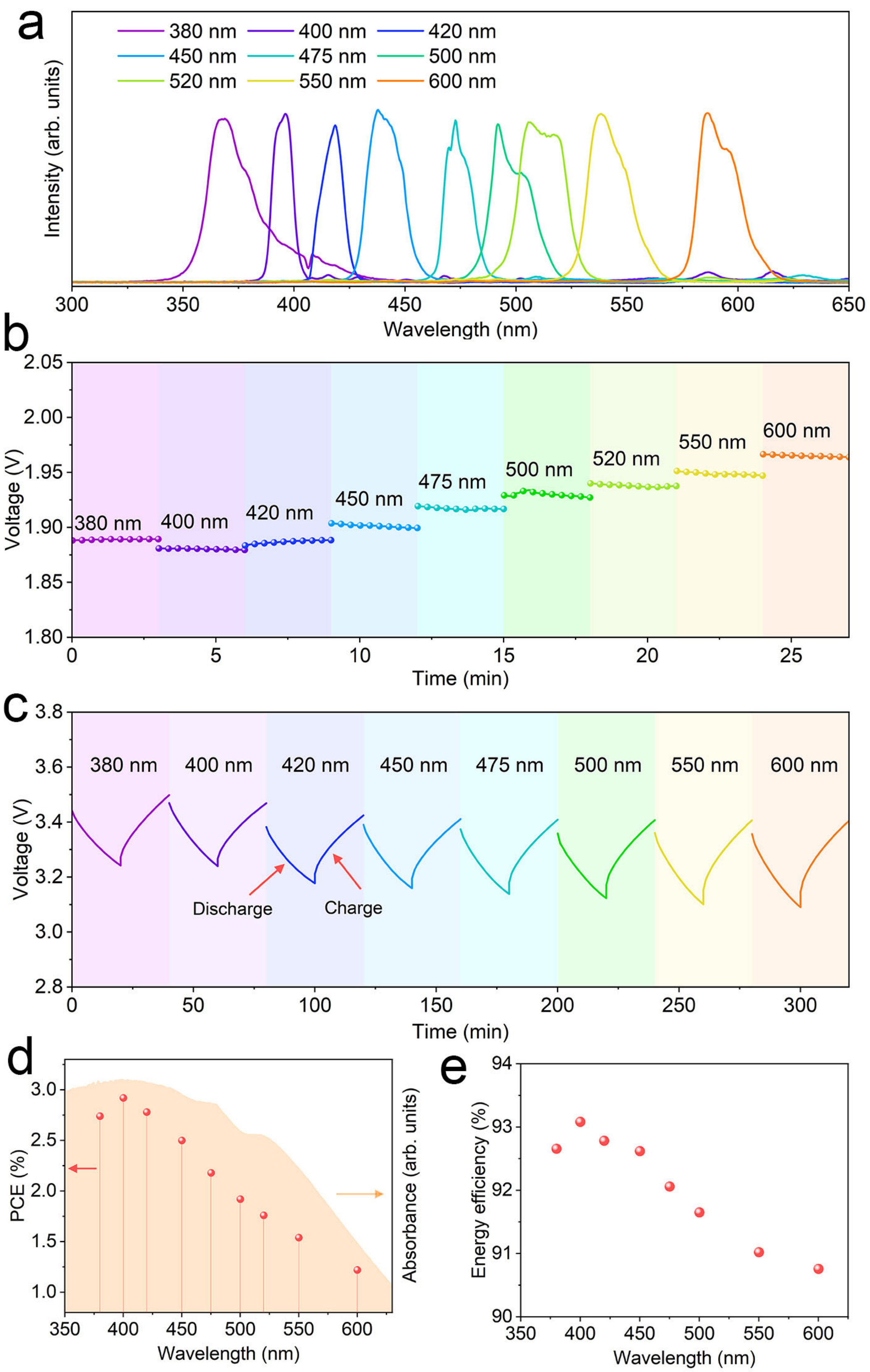

Supplementary Fig. 67. (a) The output spectrum of different wavelengths of light. (b) Charging curves of PZAB based on NGDY at different wavelengths of light (Light intensity: 5 mW cm^{-2}; current density: 1 mA cm^{-2}). (c) Discharging and charging curves of PLOB based on NGDY at different wavelengths of light (Current density:

0.01 mA cm^{-2}). (d) The PCE at different wavelengths of light (The background refers to the absorbance of NGDY). (e) The energy efficiency at different wavelengths of light.

To unequivocally demonstrate that the performance enhancement primarily originates from the intrinsic photoelectronic properties of NGDY—rather from a broadband thermal effect—we performed a critical wavelength-dependence study. A generic photothermal mechanism would predict a spectral response that correlates with the infrared absorption and heating efficiency of the system, typically increasing towards longer wavelengths.

We systematically measured the charging performance of the photo-coupled Zn||air battery under monochromatic illumination at equal light intensity (5 mW cm^{-2}), using narrowband filters for wavelengths from 380 to 600 nm (Supplementary Fig. 67a).

The results reveal a response pattern that is inconsistent with a photothermal origin but aligns perfectly with a photoelectronic mechanism:

i. The reduction in charging voltage and the calculated power conversion efficiency (PCE) show a pronounced, sharp maximum at 400 nm (PCE = 2.92%).

ii. The plots of PCE versus wavelength are closely mirrors the optical absorption profile of NGDY (Supplementary Fig. 67d), not a monotonically increasing function towards the infrared.

To further substantiate the photoelectrochemical origin of the enhancement, we investigated the Li||O_2 battery under monochromatic illumination. As presented in Supplementary Fig. 67e, the energy efficiency of the charging process exhibits a strong dependence on the incident wavelength, culminating in a sharp maximum of 93.1% at 400 nm. This distinct action spectrum aligns precisely with the characteristic optical absorption edge of NGDY (Fig. 2b). The observation of such a material-specific, resonant efficiency peak provides definitive evidence that the performance gains are governed by the intrinsic electronic excitation of NGDY.

The observed peak at 400 nm signifies a resonant light-matter interaction where the photon energy optimally matches the energy required for charge excitation within the NGDY framework. This specific, narrow-band resonance is a hallmark of a photoelectrochemical process governed by the material's electronic band structure. In contrast, a thermal-dominant response would not produce such a distinct peak at the material's specific absorption edge but would show a broader or different spectral dependence.

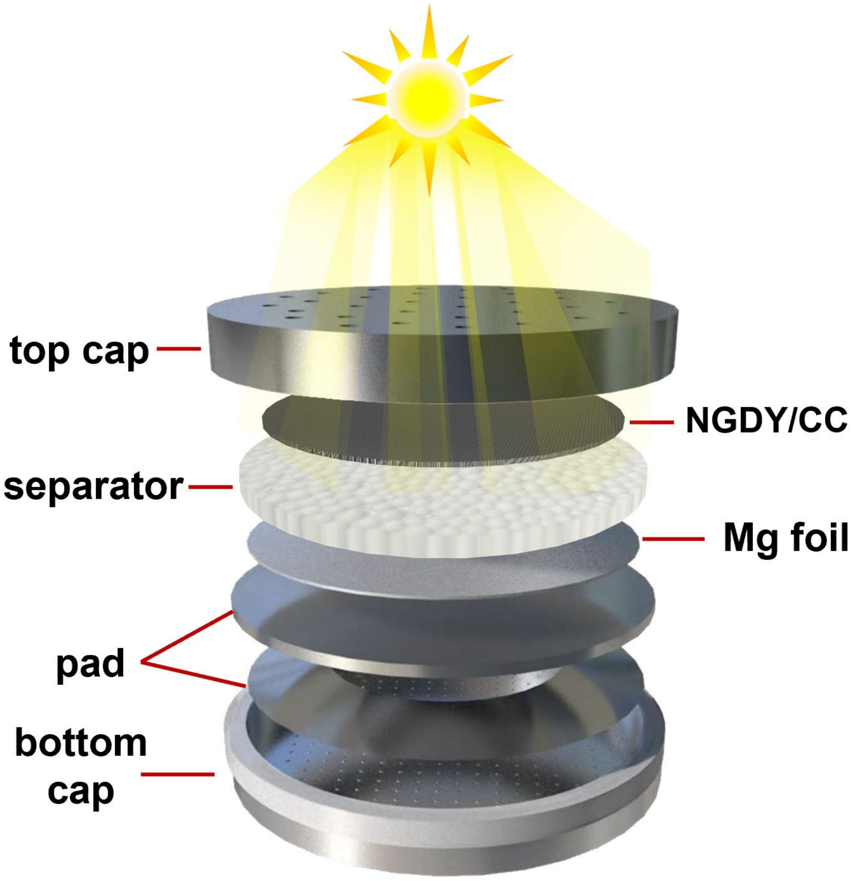

Supplementary Fig. 68. Schematic illustration of the structure of NGDY-based photo-coupled Mg||air battery.

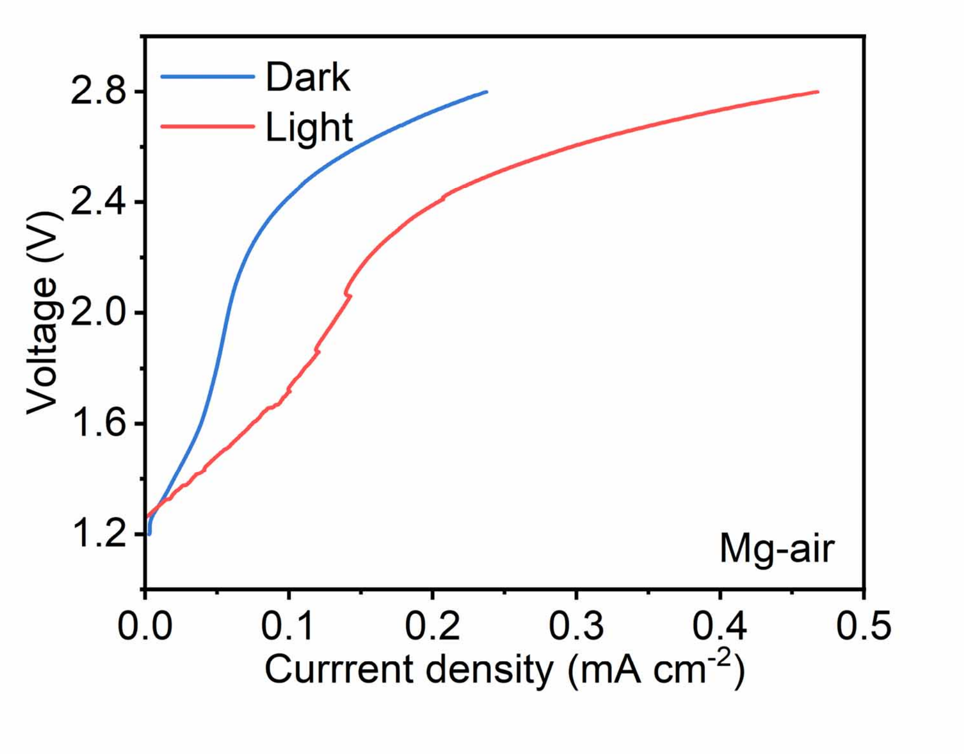

Supplementary Fig. 69. Charging polarization curves of NGDY-based photo-coupled Mg||air battery.

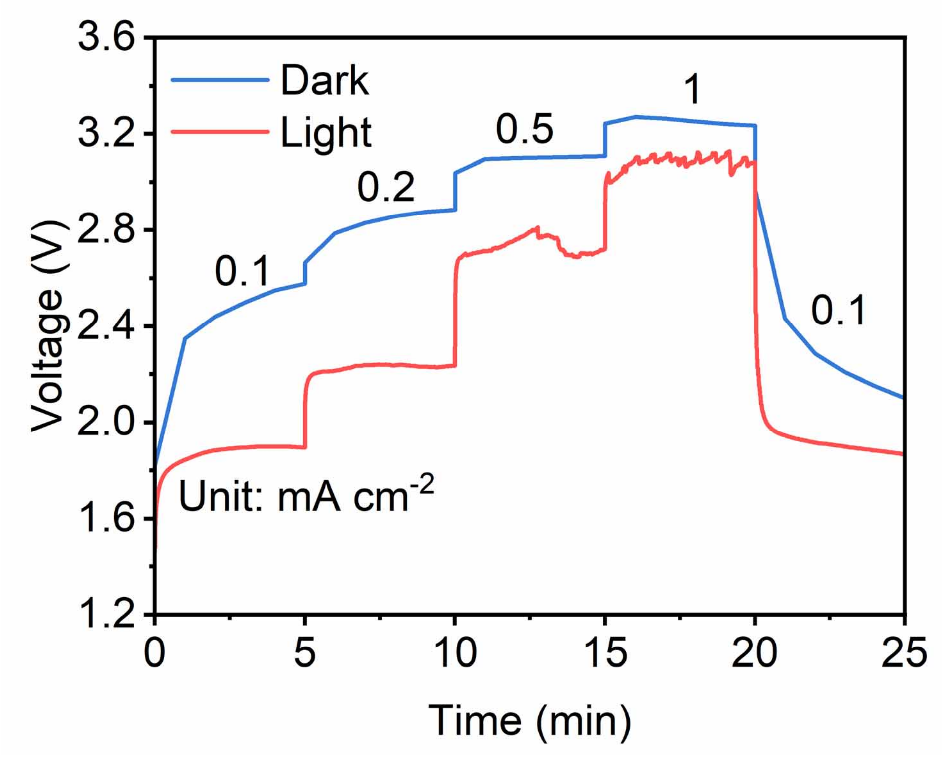

Supplementary Fig. 70. Charge curves of Mg||air battery at current densities from 0.1 mA cm^{-2} to 1 mA cm^{-2} based on NGDYs.

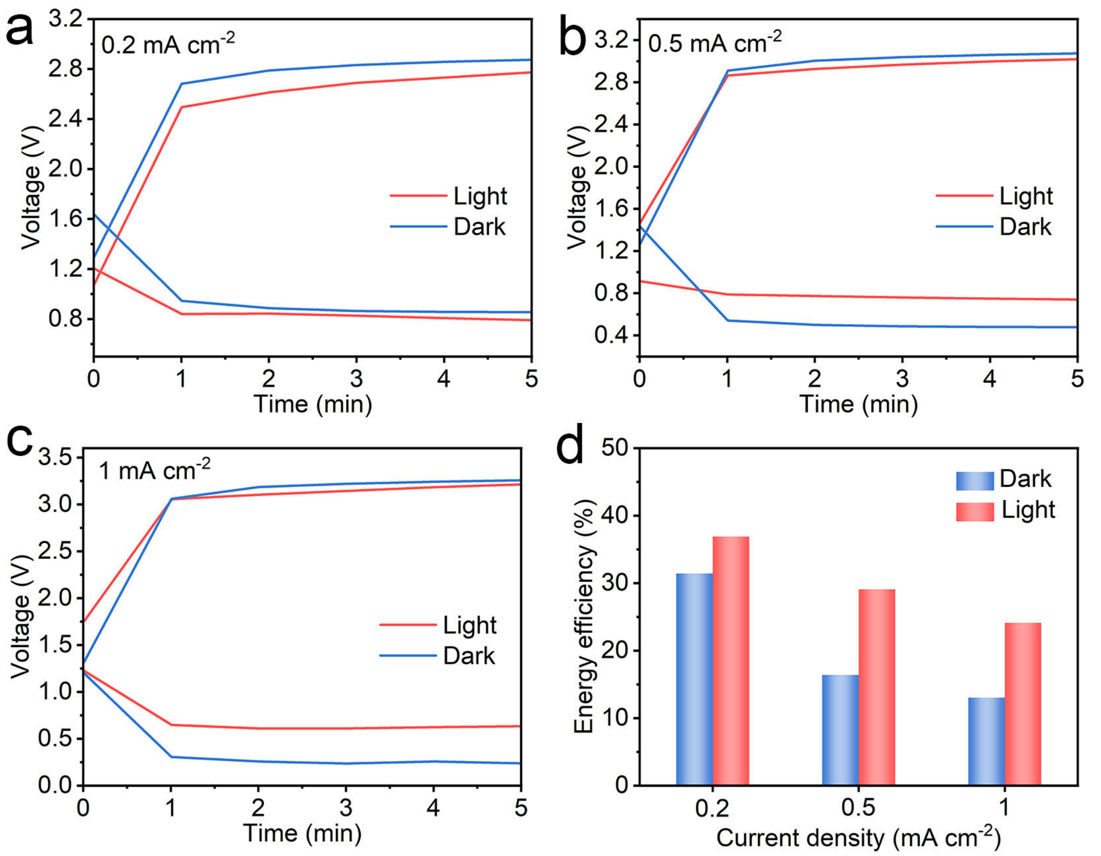

Supplementary Fig. 71. Discharge and charge curves of Mg||air battery with NGDY in the dark and under light irradiation at (a) 0.2 mA cm^{-2}, (b) 0.5 mA cm^{-2}, (c) 1 mA cm^{-2} and (d) corresponding energy efficiency.

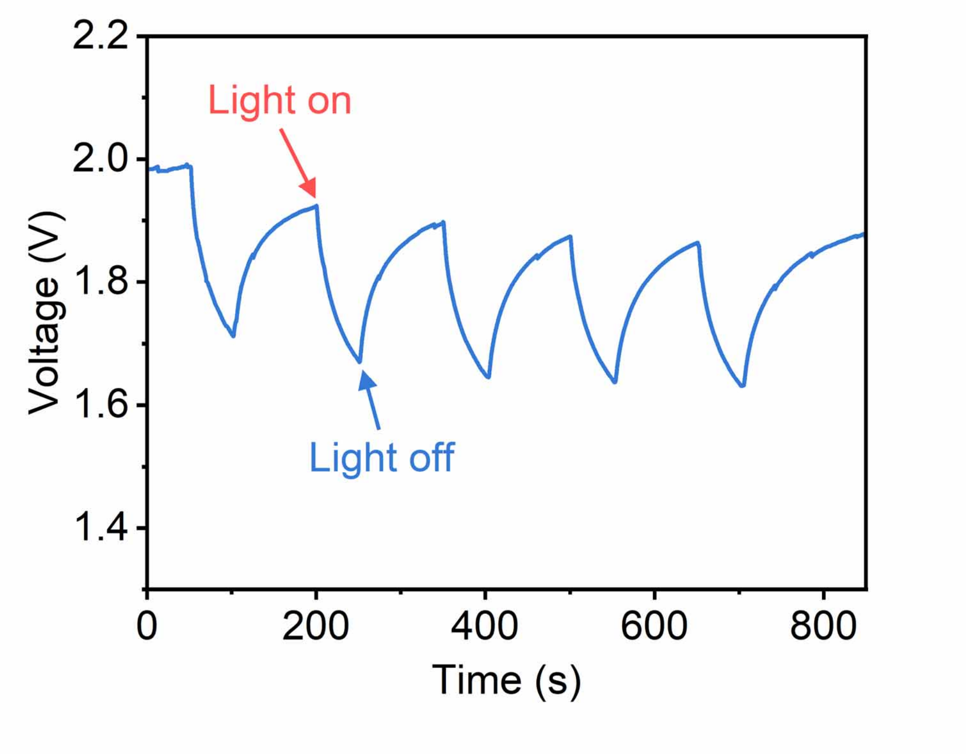

Supplementary Fig. 72. Charge curve of the Fe||air battery based on NGDY under intermittent light irradiation.

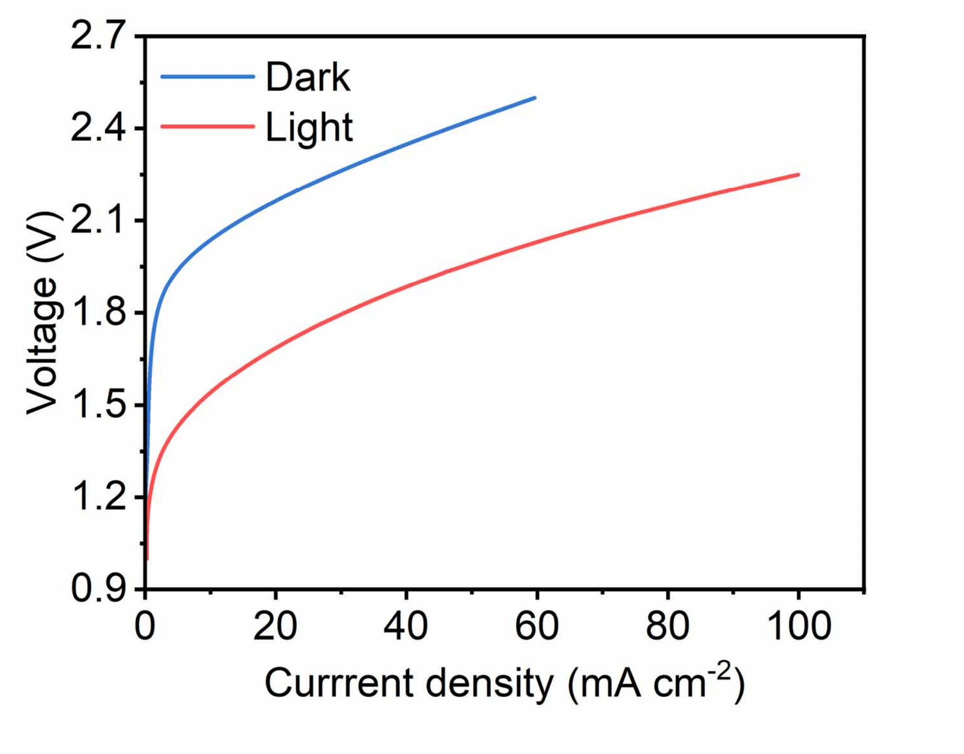

Supplementary Fig. 73. Charging polarization curves of NGDY-based photo-coupled Fe||air battery.

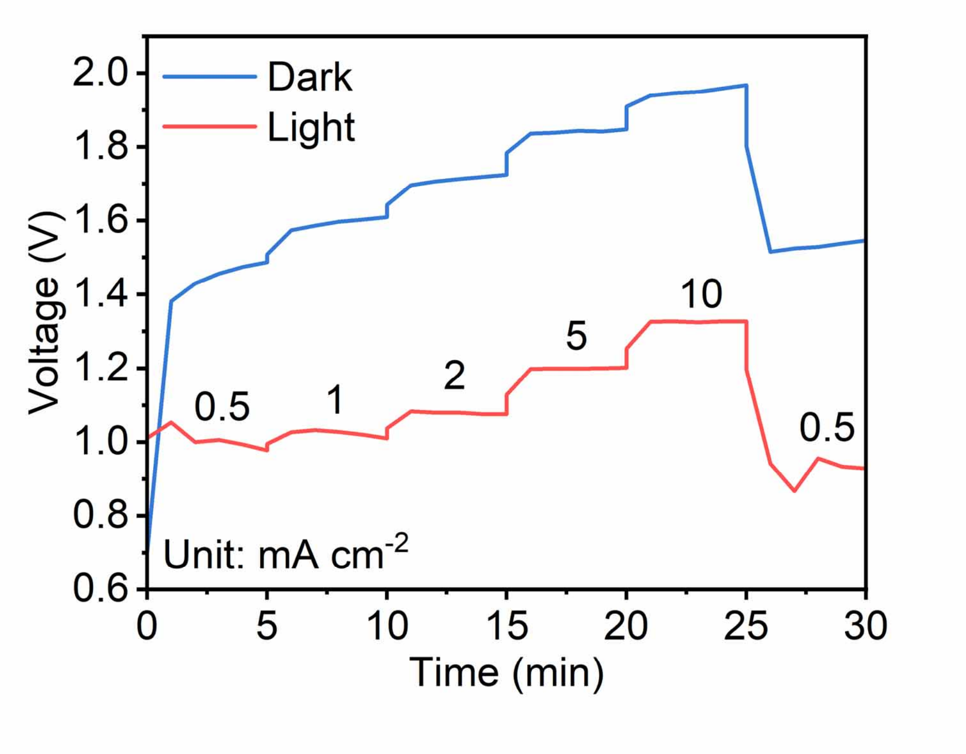

Supplementary Fig. 74. Charge curves of Fe||air battery at current densities from 0.5 mA cm^{-2} to 10 mA cm^{-2} based on NGDY.

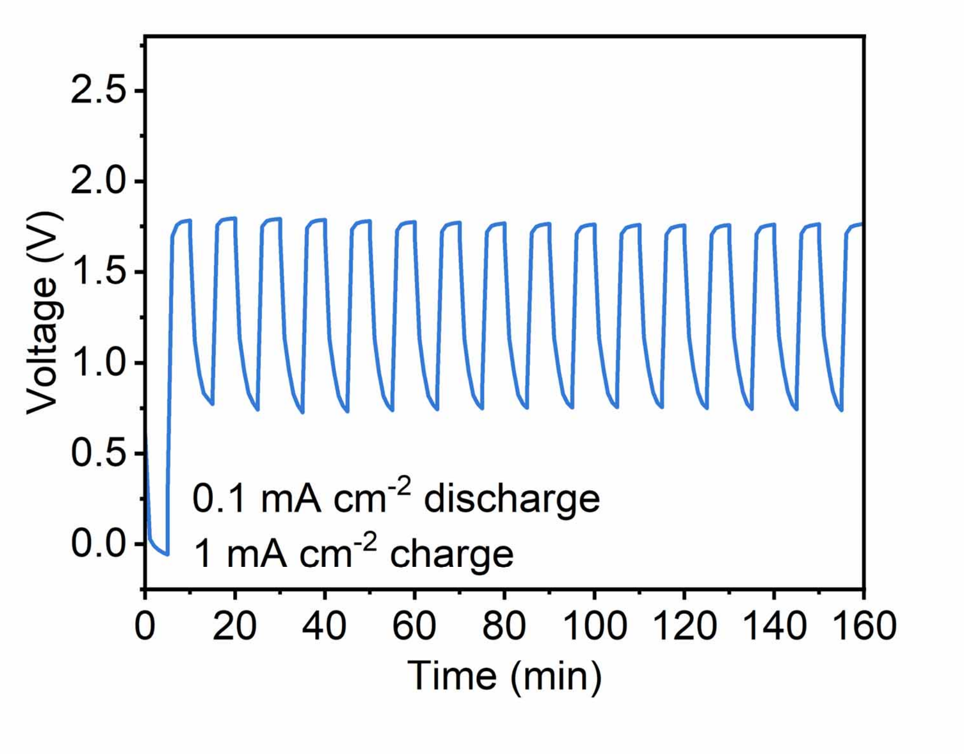

Supplementary Fig. 75. Galvanostatic discharge–charge cycling curves of NGDY-based Fe||air battery (discharging at 0.1 mA cm^{-2} and charging at 1 mA cm^{-2}).

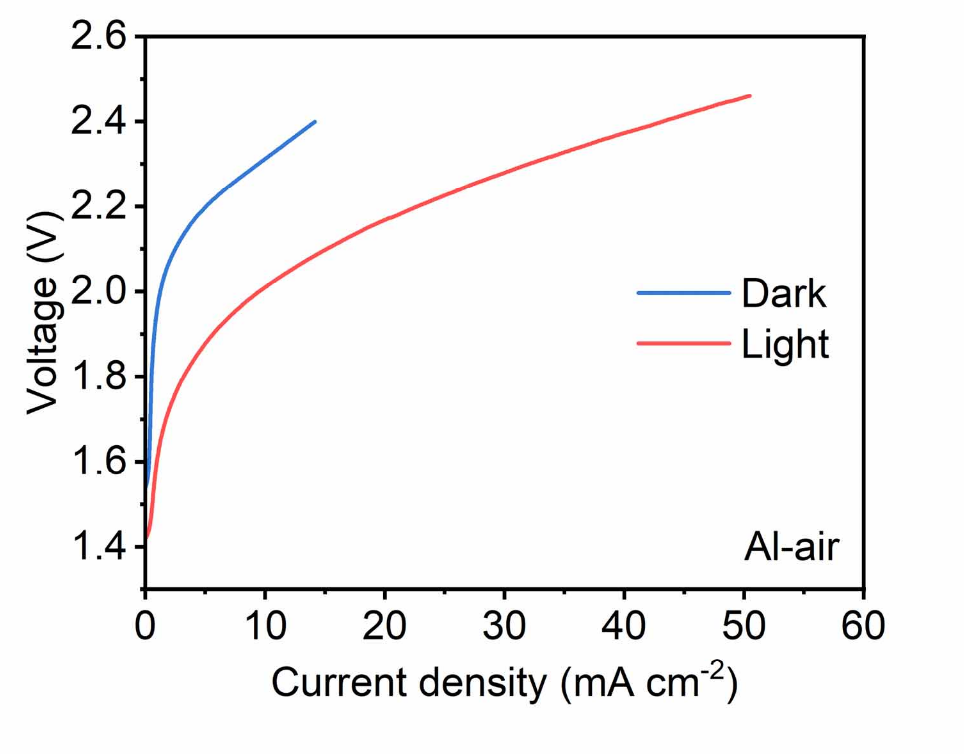

Supplementary Fig. 76. Charging polarization curves of NGDY-based photo-coupled Al||air battery.

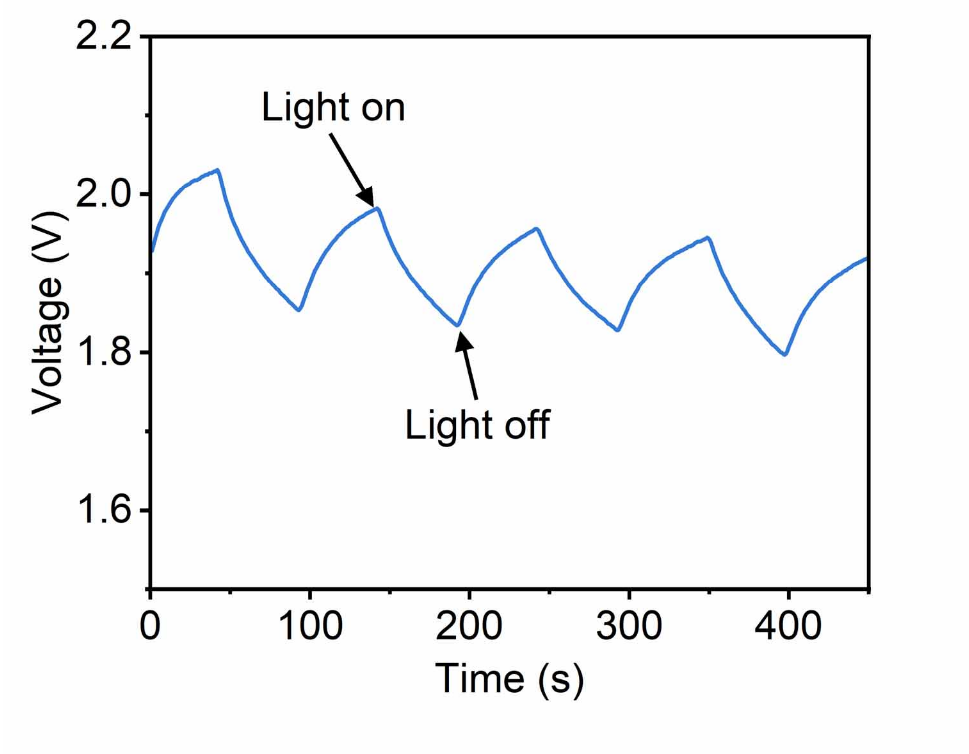

Supplementary Fig. 77. Charge curve of the Al||air battery based on NGDY under intermittent light irradiation.

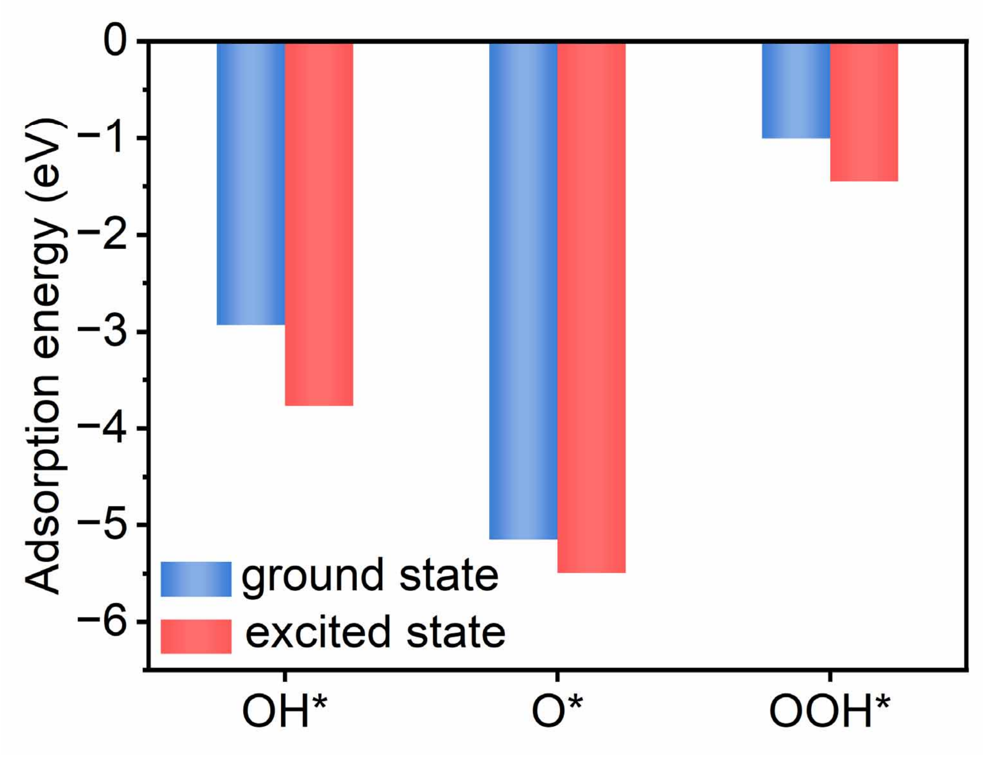

Supplementary Fig. 78. Adsorption energy of NGDY for OER intermediates in the ground state and the excited state.

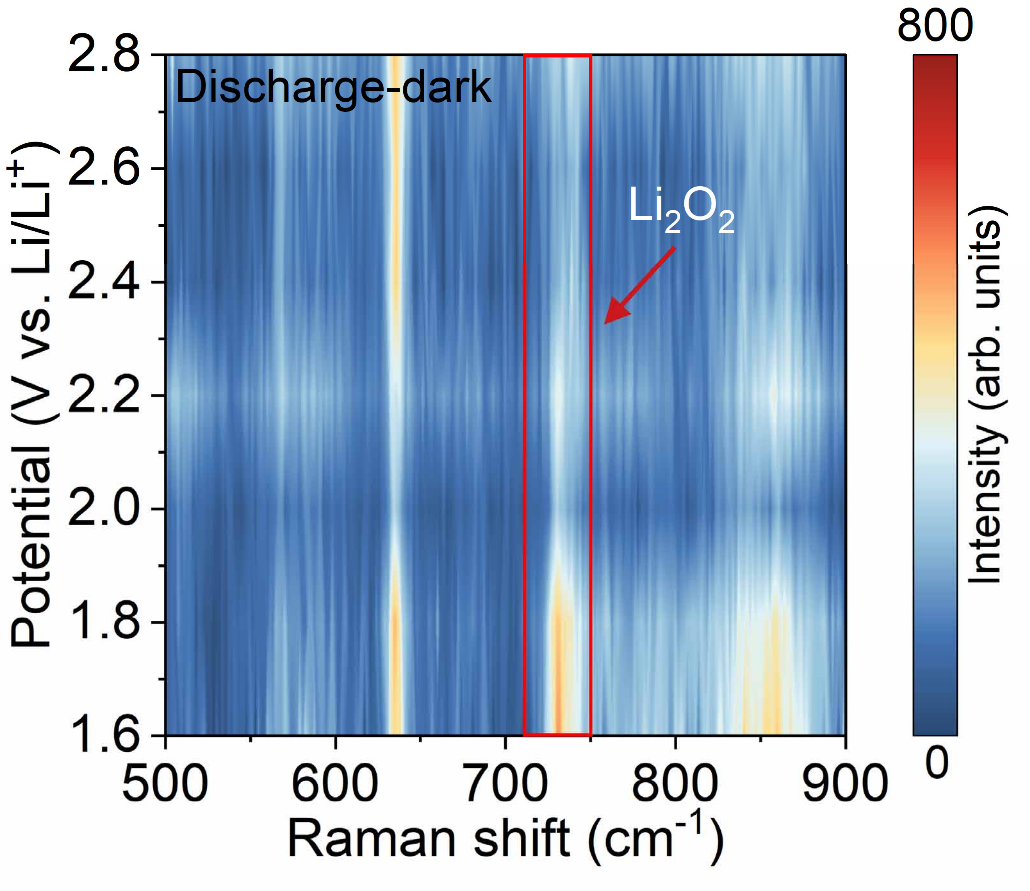

Supplementary Fig. 79. The 2D color-filled contour plot of the in-situ Raman spectra of NGDY-based PLOB during the discharging process in the dark.

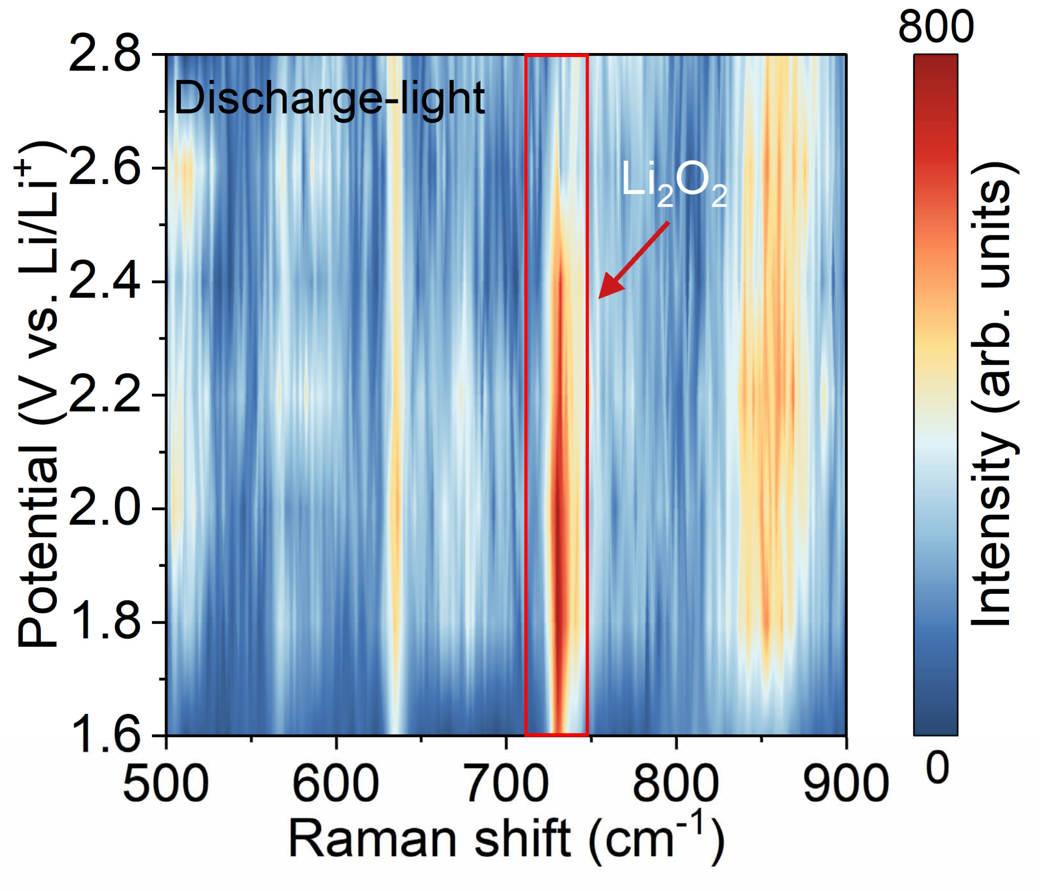

Supplementary Fig. 80. The 2D color-filled contour plot of the in-situ Raman spectra of NGDY-based PLOB during the discharging process under light irradiation.

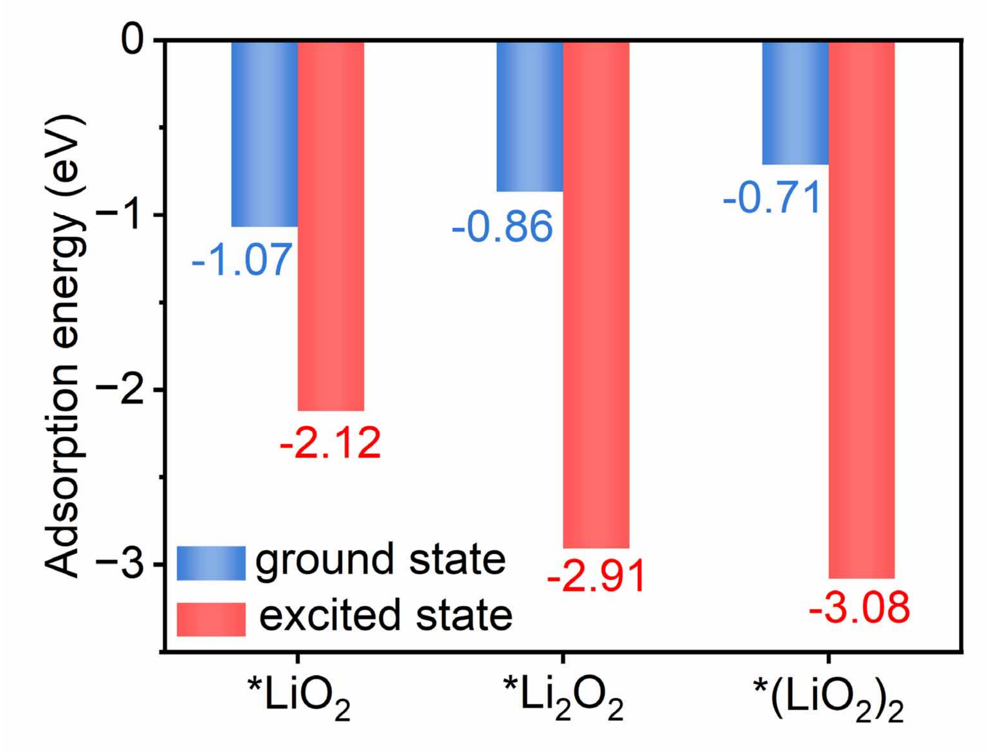

Supplementary Fig. 81. Adsorption energy of NGDY for Li||O_2 intermediates in the ground state and the excited state.

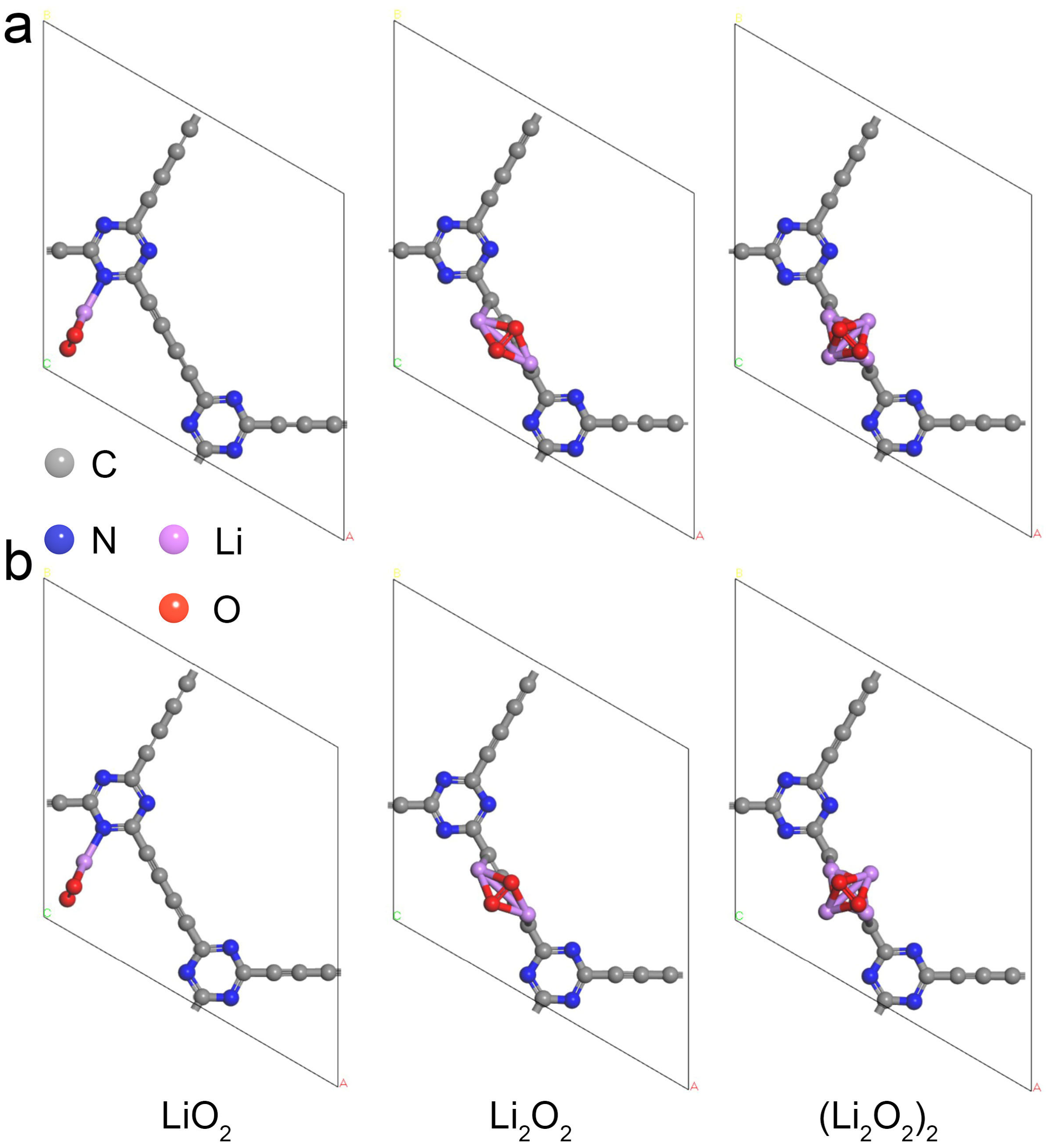

Supplementary Fig. 82. Top view of adsorption configurations of Li||O_2 intermediates at (a) ground state and (b) excited state.

Supplementary Tables

Supplementary Table 1. Comparison of the photocurrent and surface photovoltage of NGDY with other reported carbon-based semiconductors.

**Material**	**Photocurrent ($mA\ cm^{-2}$)**	**Surface photovoltage (mV)**	**Reference**
MoS_2-ONT	9.6	300	*Adv. Mater.* **2024**, 36, 2307790.
PGH	35.3	18.921	*Adv. Mater.* **2025**, 37, 2415608.
PBH	21.9	17.243	*Adv. Mater.* **2025**, 37, 2415608.
HTCN	3.7	306	*Adv. Mater.* **2023**, 35, 2306831.
**NGDY**	**130.5**	**450**	**This work**

Supplementary Table 2. Solid fluorescence lifetime fitting data.

	t_1	B_1	Rel%	t_2	B_2	Rel%
GDY	0.5983	163.399	97.87	7.6175	0.279	2.13
NGDY	0.5308	206.815	96.71	5.6243	0.664	3.29

Supplementary Table 3. Summary of charge voltage, battery efficiency, power conversion efficiency of NGDY based PZAB at different current densities.

J **(mA cm^{-2})**	**Charge voltage in dark (V)**	**Charge voltage in light (V)**	**$\eta_{battery}$ in dark (%)**	**$\eta_{battery}$ in light (%)**	**PCE (%)**
1	1.96	1.329	61.7	96.9	0.1244
2	2.05	1.341	57.1	92.5	0.2836
5	2.14	1.468	53.7	81.7	0.672
10	2.23	1.575	50.2	74.3	1.31
20	2.33	1.707	46.4	66.2	2.492

Supplementary Table 4. Summary of charge voltage, and power conversion efficiency of NGDY based photo-coupled solid PZAB at different current densities.

J (mA cm^{-2})	Charge voltage in dark (V)	Charge voltage in light (V)	PCE (%)
0.1	1.86	1.19	0.013
0.5	2.02	1.23	0.079
1	2.10	1.31	0.158
5	2.47	1.98	0.49
10	2.78	2.19	1.18

Supplementary Table 5. Summary of discharge/charge voltage, voltage gap and energy efficiency of NGDY based PLOB at different current densities.

J (mA cm^{-2})	Discharge/ charge voltage (V)	Voltage gap (V)	Energy Efficiency (%)
0.01	3.13/3.25	0.12	96.3
0.1	3.28/3.67	0.39	89.4
0.2	3.08/3.69	0.61	83.5
0.5	2.35/3.38	1.03	69.5

Supplementary Table 6. Summary of charge voltage, and power conversion efficiency of NGDY based photo-coupled Mg||air battery at different current densities.

J **(mA cm^{-2})**	**Charge voltage in dark (V)**	**Charge voltage in light (V)**	**PCE (%)**
0.1	2.53	2.20	0.033
0.2	2.85	2.24	0.122
0.5	3.10	2.75	0.175
1	3.25	3.06	0.19

Supplementary Table 7. Summary of charge voltage, and power conversion efficiency of NGDY based photo-coupled Fe||air battery at different current densities.

***J* (mA cm^{-2})**	**Charge voltage in dark (V)**	**Charge voltage in light (V)**	**PCE (%)**
0.5	1.45	1.00	0.045
1	1.59	1.02	0.114
2	1.71	1.08	0.252
5	1.84	1.2	0.64
10	1.95	1.33	1.24

Supplementary Table 8. Comparison of the performance of NGDY based PZAB with other reported photo-coupled Zn||air batteries.

Photo-electrode	*J* (mA cm^{-2})	$\eta_{battery}$ (%)	Specific capacity (mAh g_{Zn}^{-1})	Cycling stability	Reference
Ru@TS@C	0.2	~84	-	>50 cycles (33 h)	*Angew. Chem. Int. Ed.* **2025**, e202512477.
PGH	0.015	93.9	-	12 cycles (24 h)	*Adv. Mater.* **2025**, 37, 2415608.
M-TiO_2/HEAs	10	62	809	200 h	*Adv. Mater.* **2025**, 37, 2504099.
α-Fe_2O_3	0.5	~70.3	598.7	75 cycles (50 h)	*Nat. Commun.* **2019,** *10* (1), 4767.
$BiVO_4$	0.5	~50.5	538.5	9 cycles (6 h)	*Nat. Commun.* **2019,** *10* (1), 4767.
Co-doped δ-MnO_2	10	-	686.4	30 cycles (5 h)	*Mater. Today Energy* **2021,** *19*, 100612.
$Ni_{12}P_5$@ NCNT	10	64.2	640	500 cycles (83.3 h)	*Nano Energy* **2018,** *43*, 130-137.
Co_2O_3	2	~60	769	17 cycles (83.4 h)	*Chem. Commun.* **2019,** *55* (42), 5855-5858.
$NiCo_2S_4$	2	68.8	734	230 cycles (38.3 h)	*Batteries & Supercaps* **2020,** *3* (6), 541-547.
ZnO/Cu_2O	0.1	85.3	333.5	220 cycles (22 h)	*Chem. Eng. J.* **2022,** *433*, 133559.
pTTh	0.1	~86.8	-	96 cycles (64 h)	*Angew. Chem. Int. Ed.* **2019,** *58* (36), 12460-12464.

PEDOT/PEO/ CNTs	1	53.2	-	-	*Angew. Chem. Int. Ed.* **2019,** *58* (27), 9248-9253.
**NGDY**	**1**	**96.9**	**792.84**	**-**	**This work**
	**20**	**66.2**		**180 cycles (30 h)**	

Supplementary Table 9. Comparison of the performance of NGDY based PLOB with other reported photo-coupled Li||O_2 batteries.

Photo-electrode	*J* (mA cm^{-2})	Discharge/ charge voltage (V)	Voltage gap (V)	Energy Efficiency (%)	Cycling Stability (h)	Ref.
C_3N_4	0.04	3.22/3.38	0.16	95.3	-	*Angew. Chem. Int. Ed.* **2019**, *58*, 19021–19026.
TF/CC	0.01	3.1/3.29	0.19	94.2	20	*Adv. Mater.* **2020**, *32*, 1907098.
FePc	0.01	3.33/3.66	0.33	91.0	100	*Adv. Funct. Mater.* **2024**, *34*, 2405222.
Bi/TiO_2/CNTs/NF	0.05	2.63/3.7	1.07	71.1	140	*Electrochimi. Acta.* **2024**, *503*, 144833.
Fe_2O_3/CuO	0.5	2.55/3.77	1.22	67.6	140	*Appl. Surf. Sci.* **2025**,*703*,163396.
FeNi-TCPP	0.5	3.02/3.3	0.28	91.5	45	*Adv. Mater.* **2024**, *36*, 2405440.
Fe_2O_3/C_3N_4	0.1	3.13/3.19	0.06	98.1	50	*Angew. Chem. Int. Ed.* **2022**, *61*, e202116699.
WO_3 nanowire	0.06	3.05/3.25	0.2	93.8	200	*ACS Sustainable Chem. Eng.* **2019**, *7*, 5931−5939.
CdSe/ZnS@CNT	0.1	2.75/3.55	0.8	77.4	100	*Chem. Eng. J.* **2018**, *349*, 235–240.
**NGDY**	**0.1**	**3.28/3.67**	**0.39**	**89.4**	**-**	**This work**
	**0.01**	**3.13/3.25**	**0.12**	**96.3**	**80**	

References

1. Shen, H. *et al.* Nitrogen-doped graphdiyne for effective metal deposition and heterogeneous Suzuki-Miyaura coupling catalysis. *Appl. Catal. A-Gen.* **623**, 118244, (2021).
2. Liang, S. *et al.* Accelerated Confined Mass Transfer of MoS_2 1D Nanotube in Photo-Assisted Metal-Air Batteries. *Adv. Mater.*, e2307790, (2023).
3. Liu, X. *et al.* Utilizing solar energy to improve the oxygen evolution reaction kinetics in zinc-air battery. *Nat. Commun.* **10**, 4767, (2019).
4. Lv, J. *et al.* A photo-responsive bifunctional electrocatalyst for oxygen reduction and evolution reactions. *Nano Energy* **43**, 130-137, (2018).
5. Fang, Z. *et al.* Tactile UV- and Solar-Light Multi-Sensing Rechargeable Batteries with Smart Self-Conditioned Charge and Discharge. *Angew. Chem. Int. Ed.* **58**, 9248-9253, (2019).
6. Tomon, C., Sarawutanukul, S., Duangdangchote, S., Krittayavathananon, A. & Sawangphruk, M. Photoactive Zn-air batteries using spinel-type cobalt oxide as a bifunctional photocatalyst at the air cathode. *Chem. Commun.* **55**, 5855-5858, (2019).
7. Sarawutanukul, S., Tomon, C., Duangdangchote, S., Phattharasupakun, N. & Sawangphruk, M. Rechargeable Photoactive Zn-Air Batteries Using $NiCo_2S_4$ as an Efficient Bifunctional Photocatalyst towards OER/ORR at the Cathode. *Batteries & Supercaps* **3**, 541-547, (2020).
8. Ge, H. *et al.* Polyoxometallate Cluster Induced High-Entropy Oxide Sub-1 nm Nanosheets as Photoelectrocatalysts for Zn-Air Batteries. *J. Am. Chem. Soc.* **146**, 10735–10744, (2024).
9. Bu, D. *et al.* Rechargeable sunlight-promoted Zn-air battery constructed by bifunctional oxygen photoelectrodes: Energy-band switching between ZnO/Cu2O and ZnO/CuO in charge-discharge cycles. *Chem. Eng. J.* **433**, 133559, (2022).
10. Liang, S. *et al.* Efficient Carrier Separation via Ru@TS@C Zeolite: Enabling Photo-Cathodes for High-Efficiency Photo-Assisted Metal−Air Batteries. *Angew. Chem. Int. Ed.* **64**, e202512477, (2025).
11. Cui, S. *et al.* Nanosecond Laser Synthesis of MXene-Derived TiO_2/High-Entropy Alloys for Photo-Assisted Zinc–Air Batteries. *Adv. Mater.* **37**, 2504099, (2025).
12. Lin, Y., Yang, F., Wang, X., Zhong, L. & Yu, D. Organic Gradient Homojunction via D-A Engineering Enables Photoelectric/Photothermal Dual-Assisted Catalysis Toward Full Spectrum Light-Coupled Low-Temperature Seawater Batteries. *Adv. Mater.* **37**, 2415608, (2025).
13. Zhu, Z., Shi, X., Fan, G., Li, F. & Chen, J. Photo-energy Conversion and Storage in an Aprotic Li-O_2 Battery. *Angew. Chem. Int. Ed.* **58**, 19021-19026, (2019).
14. Li, M. *et al.* A Bifunctional Photo-Assisted Li-O2 Battery Based on a Hierarchical Heterostructured Cathode. *Adv. Mater.* **32**, e1907098, (2020).
15. Wang, Y. *et al.* An Enhanced Extend Interface via the π-π Interaction to Achieve the Soluble Light-Assisted Lithium-Oxygen Batteries. *Adv. Funct. Mater.* **34**, 2405222, (2024).
16. Yang, S., Sun, H., Xue, Z., Li, Q. & Yu, M. Boosting catalytic performance with bismuth for extended lifespan of light-assisted lithium-oxygen batteries. *Electrochimica Acta* **503**, 144833, (2024).
17. Bai, M. *et al.* Binder-free Fe_2O_3/CuO nanorod arrays for high-efficiency and stable photo-assisted lithium-oxygen batteries. *Applied Surface Science* **703**, 163396, (2025).
18. Wen, B. *et al.* Exciton Dissociation into Charge Carriers in Porphyrinic Metal-Organic Frameworks for Light-Assisted Li-O(2) Batteries. *Adv. Mater.* **36**, e2405440, (2024).

19. Zhu, Z. *et al.* Internal Electric Field and Interfacial Bonding Engineered Step-Scheme Junction for a Visible-Light-Involved Lithium-Oxygen Battery. *Angew. Chem. Int. Ed.* **61**, e202116699, (2022).
20. Feng, Y. *et al.* Enhanced Li_2O_2 Decomposition in Rechargeable Li–O_2 Battery by Incorporating WO_3 Nanowire Array Photocatalyst. *ACS Sustainable Chemistry & Engineering* **7**, 5931-5939, (2019).
21. Veeramani, V. *et al.* CdSe/ZnS QD@CNT nanocomposite photocathode for improvement on charge overpotential in photoelectrochemical Li-O2 batteries. *Chem. Eng. J.* **349**, 235-240, (2018).
