# Supplementary material for: Direct solar energy charging of metal||air batteries enabled by photo-coupled electrodes
Source: Nat Commun. 2026 Jun 2;17:7060. doi: 10.1038/s41467-026-73926-z (PMC13392032; doi:10.1038/s41467-026-73926-z)
Supplement: Supplementary file 2 — Description of Additional Supplementary Files [file 41467_2026_73926_MOESM2_ESM.pdf]

Description of Additional Supplementary Files

Supplementary Data 1. The optimized DFT calculation model of the ground-state NGDY.

Supplementary Data 2. The optimized DFT calculation model of the excited-state NGDY.

Supplementary Data 3. The optimized DFT calculation model of the ground state of the Li||O_2 battery intermediate LiO_2.

Supplementary Data 4. The optimized DFT calculation model of the excited state of the Li||O_2 battery intermediate LiO_2.

Supplementary Data 5. The optimized DFT calculation model of the ground state of the Li||O_2 battery intermediate Li_2O_2.

Supplementary Data 6. The optimized DFT calculation model of the excited state of the Li||O_2 battery intermediate Li_2O_2.

Supplementary Data 7. The optimized DFT calculation model of the ground state of the Li||O_2 battery intermediate $(Li_2O_2)_2$.

Supplementary Data 8. The optimized DFT calculation model of the excited state of the Li||O_2 battery intermediate $(Li_2O_2)_2$.
