# Supplementary material for: Direct solar energy charging of metal||air batteries enabled by photo-coupled electrodes
Source: Nat Commun. 2026 Jun 2;17:7060. doi: 10.1038/s41467-026-73926-z (PMC13392032; doi:10.1038/s41467-026-73926-z)
Supplement: Supplementary file 4 — Transparent Peer Review file [file 41467_2026_73926_MOESM4_ESM.pdf]

nature portfolio

Peer Review File

Direct solar energy charging of metal||air batteries enabled by photo-coupled electrodes

Corresponding Author: Professor Yuliang Li

This file contains all reviewer reports in order by version, followed by all author rebuttals in order by version.

Version 0:

Reviewer comments:

Reviewer #1

(Remarks to the Author)
The author directly excited the dynamic reconstruction of sp hybrid carbon networks through light, and proposed nitrogen-substituted graphite diyne (NGDY) as a metal-free anode for integrated solar charging in rechargeable batteries. Based on NGDY, the photo-coupled cathode exhibited excellent performance in various metal-air batteries (Zn-air, Li-O2, Mg-air, Fe-air, and Al-air), including a record-low charging voltage of 1.33 V for the Zn-air battery and an energy efficiency of 96.9%, as well as outstanding stability (230 cycles at 100 mA cm-2, with a Coulombic efficiency > 96%). The efficiency of the Li-O2 battery reached 96.3%, while the charging voltages of the Mg-air, Fe-air, and Al-air systems were significantly reduced. However, there are many issues that need to be addressed and corrected in the manuscript. Hence, this manuscript should undergo significant revisions before re-considering.

1. In the manuscript, the phase of NGDY was determined solely through nuclear magnetic spectroscopy. This is different from the conventional use of X-ray diffraction analysis (XRD). Could XRD spectra be used to confirm the phase of NGDY? If not, please explain the reason.
2. The author presents a large number of TEM images belonging to NGDY in Fig. 1. However, the lattice fringes in these images are all different. Please explain the reasons for these varying lattice spacings.
3. In the manuscript, Fig. 2i compares the photocurrents of different semiconductors in the chop light. However, the carbon cloth is not a semiconductor. So, what is the source of the photocurrent shown in the figure for the carbon cloth?
4. In Fig. 3c, the author demonstrated the C≡C cleavage through the ratio of C=N to C≡C. This seems to be problematic. The change in the ratio of C=N to C≡C cannot determine whether it is C≡C cleavage or an increase in C=N. Should the cleavage of C≡C be proven by the decrease in the proportion of C≡C throughout the C peak?
5. In Fig. 3d of the manuscript, the alkyne bonds at 2180 cm-1 are almost indistinguishable and cannot prove any changes in the alkyne bonds under light conditions.
6. In the long-cycle test at 100 mA cm-2 in the optically coupled zinc-air battery, as shown in Supplementary Fig. 26, abnormal discharge occurred at 300 minutes in the NGDY-dark condition and at 750 minutes in the commercial Pt/C + IrO2 condition. This makes the data in this group unreliable. Please check the reproducibility of this data.
7. According to Fig. 5e, the reaction potential energy barrier at the C3 site is significantly lower than that at the C2 site, regardless of whether the C2 site is excited or not. This is contrary to the author's assertion that the C2 site is the active site for catalyzing the OER process.
8. In the in-situ Raman test, the charging and discharging curves of the battery are missing, which makes the pictures difficult to understand. Let's assume that the bottom-up approach represents the battery's discharge-charging process. Then, at the base state of the battery (i.e., the lowest position of the in-situ Raman spectrum), why are there peaks belonging to Li2O2 and LiO2 present?
9. In general, the existence of LiO2 usually means the low charge overpotentials. However, the charge overpotential of NGDY-based PLOB is very high in the dark. It is unreasonable.
10. Please detect the gas evolution of NGDY-based PLOB during the discharging process and during the charging process after cycles to confirm its reversibility.
11. Why does the authors choose nitrogen-substituted graphdiyne as the photocathode? How about N-substituted graphene, N-substituted porous carbon and C3N4?

Reviewer #2

(Remarks to the Author)
The article entitled "Direct solar energy charging applied to photo-coupled electrodes" by Fu et al. discusses the role of nitrogen-substituted graphdiyne (NGDY) in direct solar rechargeable batteries - here in particular different air batteries. The work is detailed with several analyses and correlation, but largely lacking in terms of the newness of of the materials or concept - as it has been already demonstrated by several researchers the "effect of light in battery charging" (including metal ion batteries) with different materials. NGDY is also not novel and it has been already shown for various catalytic applications etc. due to its conductivity and activity towards radical stabilisation and hence kinetics tuning. Apart from the novelty, one of the main concerns, effect of temperature in such systems and it is very apparent from the light response (in aqueous vs non-aqueous electrolyte systems) that heat is playing a seminal role in the voltage enhancement. This has been recently studied by different researcher groups such as by Prof Volder's group at Cambridge (ACS Energy Lett. 2023, 8, 11, 4625–4633) and Prof Narayanan's group at TIFR (Nano Lett. 2025, 25, 2, 628–634). This effect is particularly prominent is aqueous batteries and hence needs to be carefully dissected. This has not been done by the researchers here. The obtained different photo voltages in different cases are also establishing this fact. This can not be interpreted as a simple quasi level shifting with the light irradiation. If it is due to the OER energetics differences (not simply photocurrent but voltage), the OER under light and dark is not showing such a difference. Hence, I would rather interpret the enhanced light performance of the batteries as a heat + photocurrent effect rather than a photocharging effect. The authors also need to prove with different energies (wavelength) of light and see how the voltage varies according to the nature of light. There are several typos and also unclear details of experiments including the way the photo charging has been carried out. Hence, overall, I can not recommend the manuscript for publication in a forefront journal like Nature Communication due to lack of novelty.

Reviewer #3

(Remarks to the Author)
The manuscript by Li and co-workers presents a new class of photocathode where the catalytic activity is directly governed by light. The authors revealed the unique photoexcitation mechanism of nitrogen-substituted graphdiyne (NGDY), which involves the direct photoexcitation of sp-hybridized carbon domains to dynamically reconstruct the electronic structure, generating transient and highly active catalytic centers. This is fundamentally different from the formation of traditional semiconductors with static active sites. This light-defined catalytic function is confirmed through in-situ spectroscopic techniques, which capture the intramolecular charge transfer and strengthened intermediate adsorption upon illumination. This intrinsic activation enables universally superior performance across Zn-air, Li-O2, Mg-air, Fe-air, and Al-air batteries. The photo-coupled Zn-air battery achieves a low charging voltage of 1.33 V, an energy efficiency of 96.9%, and exceptional stability over 230 cycles at an ultra-high current density of 100 mA cm-2, while the Li-O2 system reaches a breakthrough efficiency of 96.3%. The work represents a significant advance in employing light as a direct tool for engineering catalytic sites, offering a transformative pathway for the next generation of high-efficiency, photo-coupled energy systems. Therefore, I recommend publishing this paper after addressing the following issues.
1. The authors propose the core highlight of light-induced dynamic reconfiguration of high-activity sites, which is different from the conventional approaches that rely on photo-generated holes as static sites. But the essential difference between this dynamic reconfiguration and the mechanism of static sites needs to be explained more clearly.
2. Although the authors show the stability of the electrode morphology after cycling, further characterization is needed to investigate the possible changes in the chemical structure of the electrode after cycling.
3. PZAB based on photo-excited NGDY exhibits better stability than commercial Pt/C+IrO2. This is a key and attractive application advantage, but the root of the enhanced stability under photo-excitation needs to be further explored.
4. The authors summarized the performance of PZAB and photo-coupled Mg-air batteries, but it lacks a summary of the performance of solid PZAB, PLOB, etc., which needs further supplementation.
5. Although in situ Raman spectroscopy revealed the evolution of charge and discharge products during the PLOB cycling process, more direct evidence (e.g., XPS, XRD) is needed to prove the structure of the discharge products.
6. A more detailed description of the parameter information for experimental devices (such as battery assembly, in situ Raman spectroscopy) should be provided to offer readers clearer guidance.
7. The authors mentioned performance several times and uses ‘energy efficiency’ in the contexts. However, when describing PLOB, it mentioned ‘round-trip efficiency’, and in the Methods part, it mentioned ‘battery efficiency’. Are there any differences among these three? If so, please explain. If not, a unified statement needs to be made.

Reviewer #4

(Remarks to the Author)
This study presents a novel strategy for solar-rechargeable metal–air batteries via direct photoexcitation of sp-hybridized carbon units. By synthesizing nitrogen-substituted graphdiyne (NGDY) as a metal-free photocathode, the authors demonstrate significant performance enhancements across Zn-, Li-, Mg-, Fe-, and Al–air systems. The work provides compelling experimental and theoretical evidence regarding light-induced intramolecular charge redistribution and dynamic active site generation, offering valuable mechanistic insights into photo-coupled energy storage. Although the study is well-structured and supported by convincing data, certain aspects of the presentation and discussion require further clarification. Consequently, I recommend publication after minor revision.

1. The direct photoexcitation of sp-C is a significant innovation. I suggest adding a schematic diagram contrasting this mechanism with traditional carrier separation to elucidate the distinction and enhance readability.

2. The description of key illumination parameters—such as light intensity, spectral range, and spatial uniformity—is insufficient. It is recommended to provide these details in the Methods section to ensure experimental reproducibility and

clarity.

3. The Tauc plot in Figure 2c indicates a bandgap of 1.87 eV, whereas DFT calculations yield 2.52 eV for the monolayer and 2.11 eV for the nine-layer model. Given the significant discrepancy between the experimental and computational values, the authors should discuss the potential origins of this difference.

4. In Figure 2j, the authors report a photocurrent density of 120.2 μA cm−2 for the NGDY electrode without applied bias voltages. However, the key experimental parameters such as the illumination intensity, spectral range of the light source, and electrolyte composition are not provided. Additionally, a stability test should be included to demonstrate the robustness of the photoactive material.

5. Although NGDY exhibits a coulombic efficiency >96% after 230 cycles at 100 mA cm−2, post-cycling structural characterization (e.g., XPS, TEM) is necessary to confirm material integrity. Specifically, the authors should address the stability of the sp-C bonds under prolonged photo-electrochemical coupling conditions?

6. The data in Supplementary Fig. 13 appear to be unprocessed. Please reprocess this figure appropriately.

7. In large-scale applications, batteries are typically constructed as multilayer pouch or cylindrical battery. The authors should provide a reasonable model demonstrating how the proposed photocells can be effectively integrated into such configurations.

Version 1:

Reviewer comments:

Reviewer #1

(Remarks to the Author)
The author has provided a good response and revision to the proposed opinions. However, there are still some details that need to be corrected. I suggest that the manuscript should undergo minor revisions again before it is officially accepted.

1. In Figure R9, the Gibbs free energy of the rate-limiting step at the C1 site is marked incorrectly. Please correct it.
2. In the calculation of the carrier mobility rate in the transient fluorescence photoluminescence spectrum, the author used a three-exponential decay fitting to obtain the average carrier mobility rate. Please provide the three carrier mobility rates obtained by the fitting and their corresponding proportions.
3. In Supplementary Fig. 11 and Figure 2c, the Eg between the theoretical calculation results and the actual obtained GDY band gap has increased. Can you explain the reason for this ?
4. In the models shown in Figure 1k and 1i, the elements represented by each color of the balls are not indicated. Please mark them.

Reviewer #2

(Remarks to the Author)
The revised manuscript "Direct solar energy charging applied to photo-coupled electrodes" tried to address the queries in detail, I would say, partially the authors could able to do so. But many things are still unanswered and a few concerns becoming more serious.

Before going to the already raised concerns, one of the claims by the authors is high Coulombic efficiency, exceptionally high above 90%. This has to be really questioned as the air batteries (particularly Zn, Al etc. in alkaline medium), are known for their parasitic processes such as HER, ORR etc. in high alkaline medium, have low coulombic efficiency (unlike the conventional ion batteries). The details of Coulombic efficiency are missing from the article (or SI). Considering this as a known issue with conventional air batteries (though some reports say high Coulombic efficiency with MABs, but these are by addressing such parasitic issues by other means), this issue has not been well studied and mentioned in the manuscript, particularly the authors bring this as a novelty claim.

Different metal batteries have different set of issues in terms of capacity or energy density enhancement. Li air has cathode discharge product (LiO_2/Li_2O_2) storing/stabilising related issues while Zn air battery has issues related to sluggish kinetics of ORR/OER. Hence a study showing enhancement with all the air batteries, if it is true, may not help to prove the mechanism and role of NGDY.
Again, being an established material, the novelty of it is again needs to be questioned.

As I mentioned before, the thermal effect can happen in two ways: active and passive heating. By filtering the IR components in a spectrum, one may able to isolate the active heating. But the passive heating due to the absorption of the light by carbon or other components can still occur. This effect can be severe and the temperature can go to as higher as 55 degree celsius. Such an effect can be very different from the overall heating of the cell where, particularly in an aqueous battery, convection based mass transfer and overall kinetics can be improved and hence having much pronounced effect.

The wavelength dependent study shown in the revised manuscript is also not matched with the observed performance and the voltage enhancement is not coorelating. The absorption (quasi Fermi level shifting) with the observed battery performance needs to be discussed. I strongly believe that role of thermal effects is more pronounced than light effect, particularly in aqueous system, since the cathodic kinetics is known to be changed with temperature. The references need to be expanded with those addressed such thermal effects (as mentioned before from Prof Volder's groups and Prof Narayanan's group).

Since each battery system (air battery) has its own issues, my suggestion is to focus on one particular system in detail and establish the role of NGDY rather than showing everything but bot going to the details, which leave several doubts and concerns.

Reviewer #3

(Remarks to the Author)
All of the issues raised in my previous review have been adequately addressed and resolved. I have no further concerns regarding this manuscript. In its current form, the manuscript is suitable for publication and can be accepted, provided that no additional major issues are raised by the other reviewers.

Reviewer #4

(Remarks to the Author)
Authors answered all of my questions. I recommend its publication at the present state.

Version 2:

Reviewer comments:

Reviewer #2

(Remarks to the Author)
The authors have successfully addressed all the concerns I had.

Open Access This Peer Review File is licensed under a Creative Commons Attribution 4.0 International License, which permits use, sharing, adaptation, distribution and reproduction in any medium or format, as long as you give appropriate credit to the original author(s) and the source, provide a link to the Creative Commons license, and indicate if changes were made.
In cases where reviewers are anonymous, credit should be given to 'Anonymous Referee' and the source.
The images or other third party material in this Peer Review File are included in the article’s Creative Commons license, unless indicated otherwise in a credit line to the material. If material is not included in the article’s Creative Commons license and your intended use is not permitted by statutory regulation or exceeds the permitted use, you will need to obtain

permission directly from the copyright holder.
To view a copy of this license, visit https://creativecommons.org/licenses/by/4.0/

Point by Point Response to the Reviewers' Comments:

Reviewer #1 (Remarks to the Author):

The author directly excited the dynamic reconstruction of sp hybrid carbon networks through light, and proposed nitrogen-substituted graphdiyne (NGDY) as a metal-free anode for integrated solar charging in rechargeable batteries. Based on NGDY, the photo-coupled cathode exhibited excellent performance in various metal-air batteries (Zn-air, Li-O_2, Mg-air, Fe-air, and Al-air), including a record-low charging voltage of 1.33 V for the Zn-air battery and an energy efficiency of 96.9%, as well as outstanding stability (230 cycles at 100 mA cm^{-2}, with a Coulombic efficiency > 96%). The efficiency of the Li-O_2 battery reached 96.3%, while the charging voltages of the Mg-air, Fe-air, and Al-air systems were significantly reduced. However, there are many issues that need to be addressed and corrected in the manuscript. Hence, this manuscript should undergo significant revisions before re-considering.

Response. We are grateful for the reviewer's constructive and detailed comments, which have been invaluable in strengthening our manuscript. We have carefully addressed every point raised in the review. All necessary corrections, clarifications, and additional data analyses have been incorporated into the revised manuscript and supplementary information.

1. In the manuscript, the phase of NGDY was determined solely through nuclear magnetic spectroscopy. This is different from the conventional use of X-ray diffraction analysis (XRD). Could XRD spectra be used to confirm the phase of NGDY? If not, please explain the reason.

Response. We thank the reviewer for this valuable comment. We agree with the reviewer that X-ray diffraction (XRD) is a standard technique for phase identification.

However, for NGDY this is particularly challenging because it is a low-dimensional carbon allotrope with variable stacking, the application of XRD to NGDY is limited by the fact that NGDY is a nanostructured carbon material with weak long-range order. Its XRD pattern typically shows only a broad peak originating from the conjugated carbon framework, and the peak position is sensitive to layer number, stacking mode and defects rather than to the specific local bonding motif introduced by nitrogen doping.

We would like to clarify that the nuclear magnetic resonance (NMR) spectroscopy in the manuscript was exclusively used to confirm the molecular structure and purity of the precursor (2,4,6-(trimethylsilyl)ethynyl-1,3,5-triazine), prior to the Glaser–Hay coupling reaction. This is a standard practice in synthesis to ensure the purity. The crystalline structure of the final NGDY material were definitively characterized by transmission electron microscopy (TEM). This approach is particularly suitable and standard for 2D carbon-based materials. High-resolution TEM (HRTEM) in Fig. 1c directly resolves the in-plane lattice fringes with a spacing of 0.46 nm, corresponding to the (110) plane of the graphdiyne lattice, providing real-space evidence of crystallinity. The selected area electron diffraction (SAED) pattern in Fig. 1d shows sharp, hexagonal symmetry, which is unambiguous proof of the crystalline order and the characteristic six-fold rotational symmetry of the graphdiyne framework.

In direct response to this comment, we have performed XRD analysis on the exfoliated NGDY powder (Figure R1) and to show the material’s general crystallinity and to support the morphological analysis, which was added in the revised Supplementary Information (Supplementary Fig. 9). The XRD pattern exhibits a broad peak centered around 20°, which corresponds to the characteristic interlayer stacking distance (d-spacing) of the graphdiyne-based layered structure. This observation aligns with the interlayer spacing of ~0.365 nm measured directly by cross-sectional HRTEM (Fig. 1i-j) and provides complementary, bulk-scale evidence of the material's layered nature.

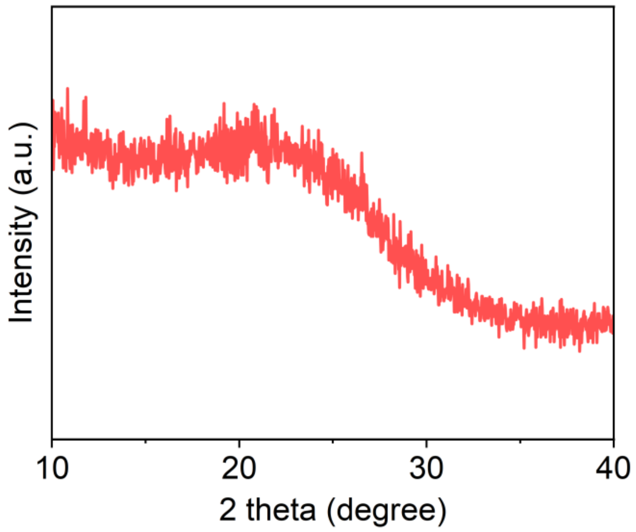

Figure R1. XRD pattern of NGDY.

Therefore, the combination of HRTEM and SAED constitutes a robust and widely accepted methodology for determining the crystal structure of 2D polymeric materials like NGDY.

2. The author presents a large number of TEM images belonging to NGDY in Fig. 1. However, the lattice fringes in these images are all different. Please explain the reasons for these varying lattice spacings.

Response. We thank the reviewer for their careful observation regarding the lattice fringes in the TEM images. Upon re-examination, we have confirmed and corrected the lattice spacing in Fig. 1c to be 0.46 nm (precisely 0.4587 nm, Figure R2). This spacing corresponds to the (110) interplanar distance of the in-plane hexagonal lattice within a single-layer NGDY framework, which is a characteristic feature of graphdiyne-family materials.

The other measured spacing of ~0.365 nm (Fig. 1i-j) represents the interlayer distance between adjacent NGDY sheets in the stacked structure. Therefore, the two distinct measurements are not inconsistent; they reflect different structural dimensions: the 0.46 nm characterizes the lattice spacing of in-plane atomic ordering, while the 0.365 nm

shows the layer spacing of out-of-plane layer stacking. We have clarified this point in the revised Figure 1 caption and the corresponding main text.

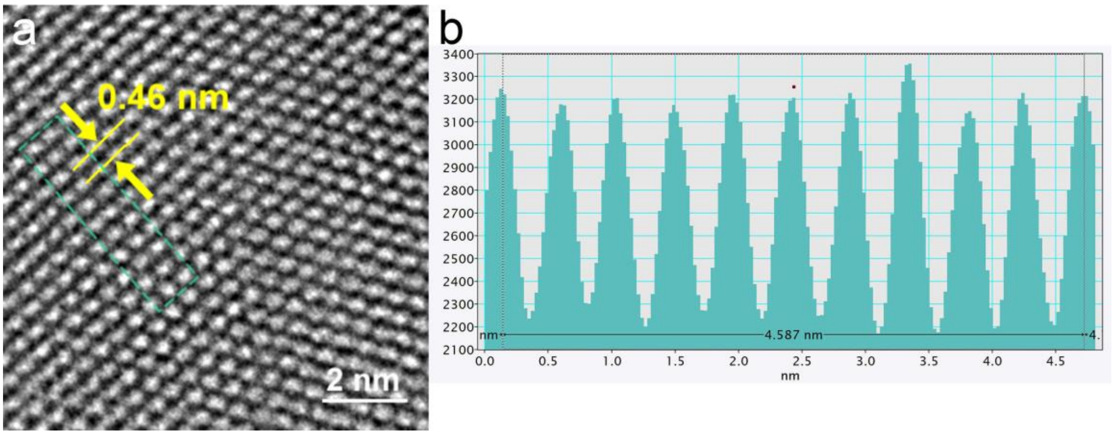

Figure R2. (a) HR-TEM images of NGDY and (b) the cross-sectional analysis along the green box.

3. In the manuscript, Fig. 2i compares the photocurrents of different semiconductors in the chop light. However, the carbon cloth is not a semiconductor. So, what is the source of the photocurrent shown in the figure for the carbon cloth?

Response. The pristine carbon cloth (CC) is not a semiconductor, and the observed weak photocurrent under illumination is attributed to a minor photothermal effect. To decisively isolate and quantify this effect, and to unequivocally demonstrate that the significant photocurrent in GDY and NGDY originates from intrinsic photoexcitation, we conducted a key control experiment using an 800 nm infrared filter. This filter removes a substantial portion of the infrared spectrum, thereby drastically reducing the heating component of the incident light. The new data (Figure R3) clearly shows that with the infrared component filtered out:

1. The transient current signal from the bare CC substrate diminishes to near-zero.
2. In contrast, the stable, significant photocurrents from both GDY and NGDY samples remain largely unchanged. This difference can be attributed to their distinct structures: the bulk carbon cloth substrate, with its larger thermal mass and surface area, is more susceptible to generating a measurable photothermal current.

In contrast, the NGDY forms only a thin coating on the CC; its photocurrent is dominated by the intrinsic semiconductor photo-response, with minimal contribution from the thin layer's negligible photothermal effect.

This result provides direct experimental evidence that:

1. The CC signal is a parasitic photothermal artifact.
2. The photo-responses of GDY and NGDY are genuine photoelectric effects arising from their intrinsic semiconductor properties, independent of heating.

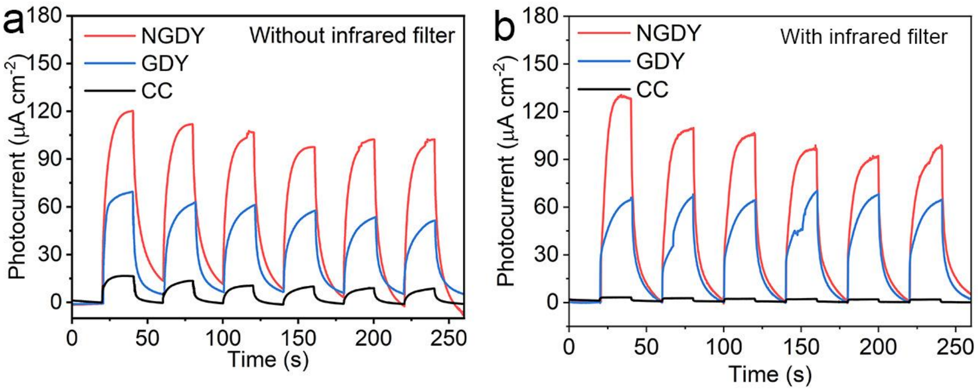

Figure R3. Photocurrent densities under intermittent light irradiation (a) without 800 nm infrared filter and (b) with 800 nm infrared filter.

Furthermore, we clarify that in our initial study, we were cognizant of the potential influence of thermal effects. For all photo-coupled battery performance tests reported in the manuscript, an 800 nm infrared filter was routinely employed to mitigate IR-induced heating. We acknowledge that this crucial methodological detail was not explicitly stated in the original submission. Prompted by the reviewer's valid concern, we have now comprehensively revised the "Methods" section to include a detailed description of the light source, spectral filtering, and the rationale for its use. We have also integrated the new control data and its interpretation into the main text to reinforce the validity of our photocurrent measurements.

We have placed the photocurrent data without infrared filters in the supplementary information, and have also provided an explanation.

This control experiment presents the photocurrent response without the 800 nm infrared filter. Under full-spectrum illumination, the bare carbon cloth (CC) substrate exhibits a detectable transient current, which is primarily attributed to photothermal effects arising from infrared absorption. In stark contrast, when the infrared component is filtered out (Fig. 2j), this signal in CC diminishes to a negligible level (see main text).

Crucially, the photocurrents of both GDY and NGDY remain substantial and stable regardless of the presence of the IR filter, demonstrating that their response originates from intrinsic photoexcitation rather than thermal artifacts. This direct comparison underscores the necessity of spectral filtering to isolate genuine photoelectrochemical activity and confirms that the performance enhancements reported in this work are driven by the semiconductor properties of the materials, not by incidental heating.

4. In Fig. 3c, the author demonstrated the C≡C cleavage through the ratio of C=N to C≡C. This seems to be problematic. The change in the ratio of C=N to C≡C cannot determine whether it is C≡C cleavage or an increase in C=N. Should the cleavage of C≡C be proven by the decrease in the proportion of C≡C throughout the C peak?

Response. Our initial analysis using the sp-C/sp^2-C ratio was intended to intuitively illustrate the relative electronic population shift between these two hybridization states under photoexcitation, as inferred from spectral fitting. To provide a direct and unambiguous quantitative measure as suggested, we have conducted a further analysis by deconvoluting the entire C 1s spectrum to determine the atomic percentage of the sp-C component relative to the total carbon signal.

The results (Figure R4) demonstrate a clear and reversible decrease in the proportion of sp-C under illumination, which recovers upon returning to dark conditions. This trend is consistent with the direction of change shown by the C≡C/ C=N ratio. Therefore, the combined data from both analyses converge to the same conclusion: photoexcitation leads to a reversible depletion of electron density specifically from the sp-hybridized carbon (C≡C) domains, facilitating intramolecular charge transfer to nitrogen sites.

Figure R4 has been put in our revised Supplementary information.

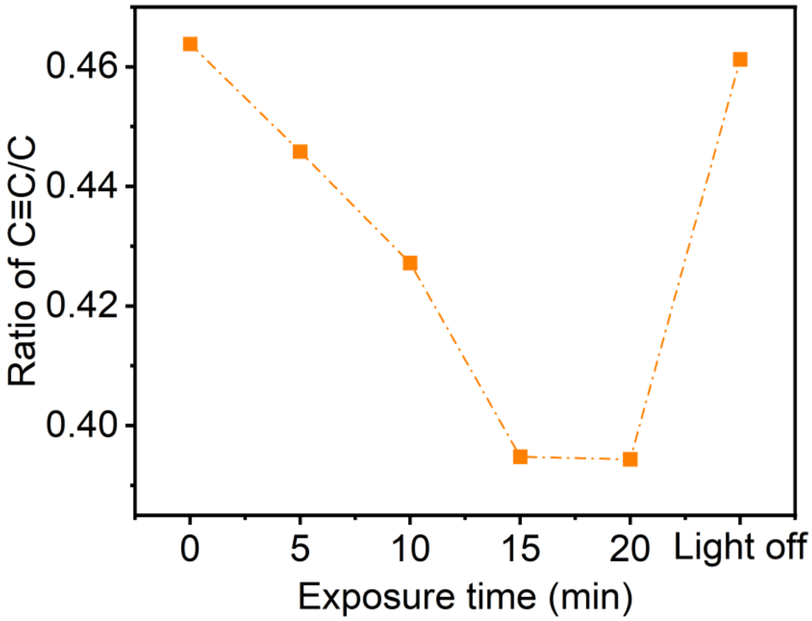

Figure R4. The changes in the proportion of sp-C throughout the C peak under light irradiation.

5. In Fig. 3d of the manuscript, the alkyne bonds at 2180 cm^{-1} are almost indistinguishable and cannot prove any changes in the alkyne bonds under light conditions.

Response. We re-performed the Raman spectroscopy tests on NGDY under dark and illuminated conditions. To precisely resolve and visualize the dynamic evolution of this key spectral feature under illumination, we specifically presented the two-dimensional (2D) color-filled contour plot in Fig. 3e. This plotting technique is highly effective in magnifying subtle spectral changes that may be less distinct in a standard line-plot format (Fig. 3d). The contour plot in Fig. 3e clearly shows the reversible disappearance and recovery of the alkyne bond signal (~2180 cm^{-1}) as a function of light on/off cycles, providing direct spectroscopic evidence for the light-modulated electronic state of the sp-C units. Furthermore, we have now provided a magnified view of the 2000–2400 cm^{-1} region in the supplementary materials (Figure R5), offering a direct line-profile

comparison that clearly shows the reversible change of the ~2180 cm^{-1} peak. This additional data, together with the contour plot, provides conclusive evidence for the light-modulated electronic state of the sp-C units.

To further improve clarity, we have added a label explicitly pointing to the alkyne bond region in Fig. 3d (Figure R6). We appreciate this comment, which has helped us enhance the presentation of this critical data.

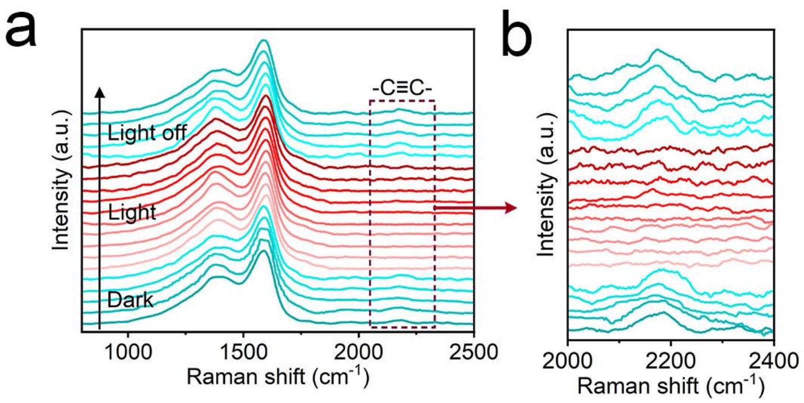

Figure R5. The enlarged view at 2000 - 2400 cm^{-1} in Fig. 3d.

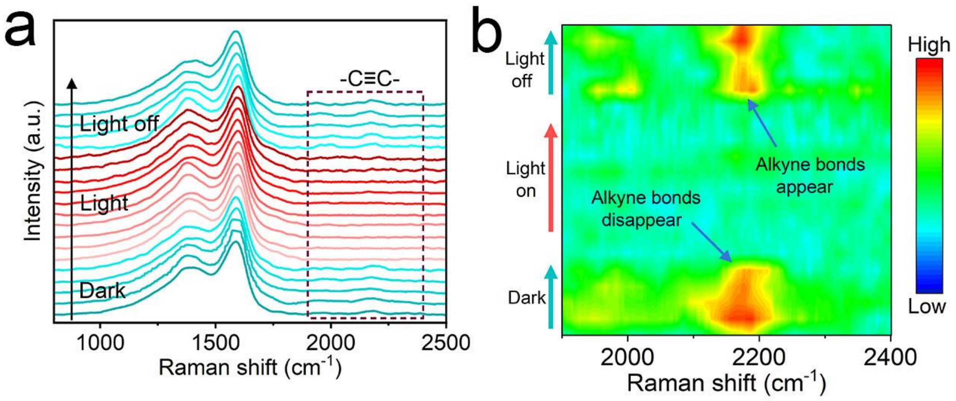

Figure R6. (a) Raman spectra of NGDY in the dark and under light irradiation. (b) The 2D color-filled contour plot of the Raman spectra magnified in the dashed box in (a).

6. In the long-cycle test at 100 mA cm^{-2} in the optically coupled zinc-air battery, as shown in Supplementary Fig. 26, abnormal discharge occurred at 300 minutes in the NGDY-dark condition and at 750 minutes in the commercial Pt/C + IrO_2 condition. This makes the data in this group unreliable. Please check the reproducibility of this

data.

Response. We appreciate the reviewer's attention to detail, which has allowed us to clarify an important aspect of our experimental design. The observed discontinuities in the discharge curves (e.g., the shortened discharge at ~300 min for NGDY-dark and ~750 min for Pt/C+IrO_2) are not due to experimental error or irreproducible data. On the contrary, they are expected phenomena under our specific testing protocol, designed to accurately evaluate Coulombic efficiency (CE) and zinc deposition behavior at a high current density of 100 mA cm^{-2}.

To directly measure the CE for each cycle, we used a copper foil as the current collector at the anode instead of a standard zinc foil. In each cycle, zinc was deposited onto the copper during the charging period and was then stripped during discharge until the deposited zinc was completely exhausted (voltage dropping to 0 V). This method allows for the simultaneous monitoring of charge overpotential and the direct calculation of cycle-specific CE.

The "abnormal" short or intermittent discharges are precisely caused by the formation, detachment, and re-contact of zinc dendrites, which is a key process we aimed to capture (Figure R7):

1. Under conditions of higher overpotential (such as with NGDY in the dark or with the Pt/C+IrO_2 catalyst), zinc tends to deposit as dendrites.
2. These dendrites are mechanically unstable and can detach from the current collector (as illustrated in the provided schematic, stage "Dendrites shedding"). Once detached, the electrical connection is lost, leading to an immediate termination of that discharge phase (discharge time ~0, CE ~0%). This corresponds to the sharp dips in the discharge curve.
3. Subsequently, the detached dendritic particles may physically re-contact the electrode, leading to a renewed, sometimes prolonged, discharge period (as seen between ~304-308 min).

4. With continued cycling, accumulated detached dendrites can eventually bridge the electrodes, causing a short circuit and test termination—a failure mechanism we document (Supplementary Fig. 38 provided optical images of Cu foil after long-term cycling test at 100 mA cm^{-2}).

We have confirmed the reproducibility of this process through repeated tests. The observed patterns are consistent and diagnostically valuable, highlighting the inferior stability and dendrite-prone nature of the compared systems under high-current cycling. To prevent misunderstanding, we have revised the Methods Section in the manuscript to explicitly describe this copper-collector-based CE measurement protocol.

To accurately assess the long-term operational stability, zinc deposition morphology evolution, and, crucially, the Coulombic efficiency (CE) of the Zn-air batteries at a high current density of 100 mA cm^{-2}, a specific testing protocol employing a copper foil as the anode current collector was adopted. This method replaces the conventional zinc foil and allows for the direct evaluation of zinc plating/stripping reversibility per cycle. The procedure is as follows: a polished copper foil was assembled as the working electrode (anode). In each cycle, the battery was first charged at a constant current density of 100 mA cm^{-2} for 60 s to deposit zinc onto the copper substrate. Subsequently, it was immediately discharged at the same current density until the deposited zinc was completely stripped, indicated by the cell voltage dropping to approximately 0 V, and the discharge time was recorded. The CE for each individual cycle was then calculated using the formula:

$$CE = (t_{discharge} / t_{charge}) \times 100\% \qquad (4)$$

Furthermore, we have added a new schematic diagram (Figure R8) to visually illustrate the zinc deposition, dendrite shedding, and re-contact process during cycling.

Therefore, the data in this group are reliable and purposefully reflect the challenging dendrite-related issues at high current densities. The presented analysis provides a more comprehensive evaluation of catalyst performance by including anode stability and Coulombic efficiency metrics. We hope this clarification addresses the concern. The

relevant revisions have been made in the manuscript.

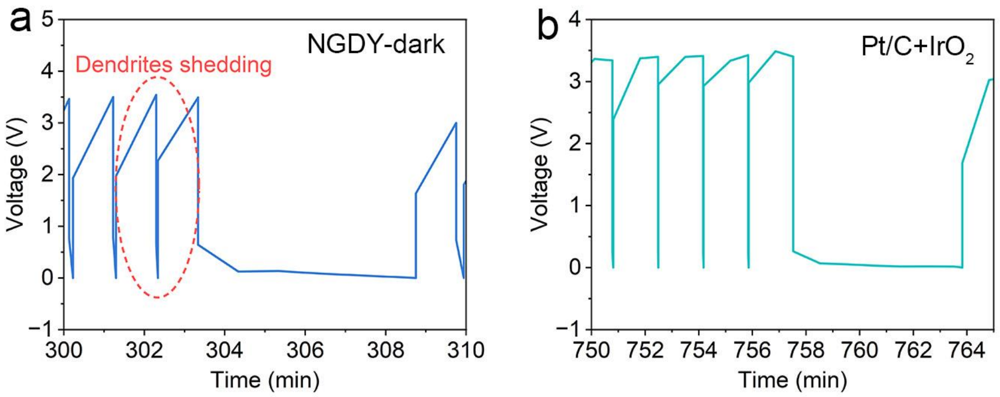

Figure R7. (a) Enlarged view at 300-310 min cycling curves of PZAB based on NGDY in the dark at 100 mA cm^{-2}. (b) Enlarged view at 750-765 min cycling curves of PZAB based on NGDY in the dark at 100 mA cm^{-2}.

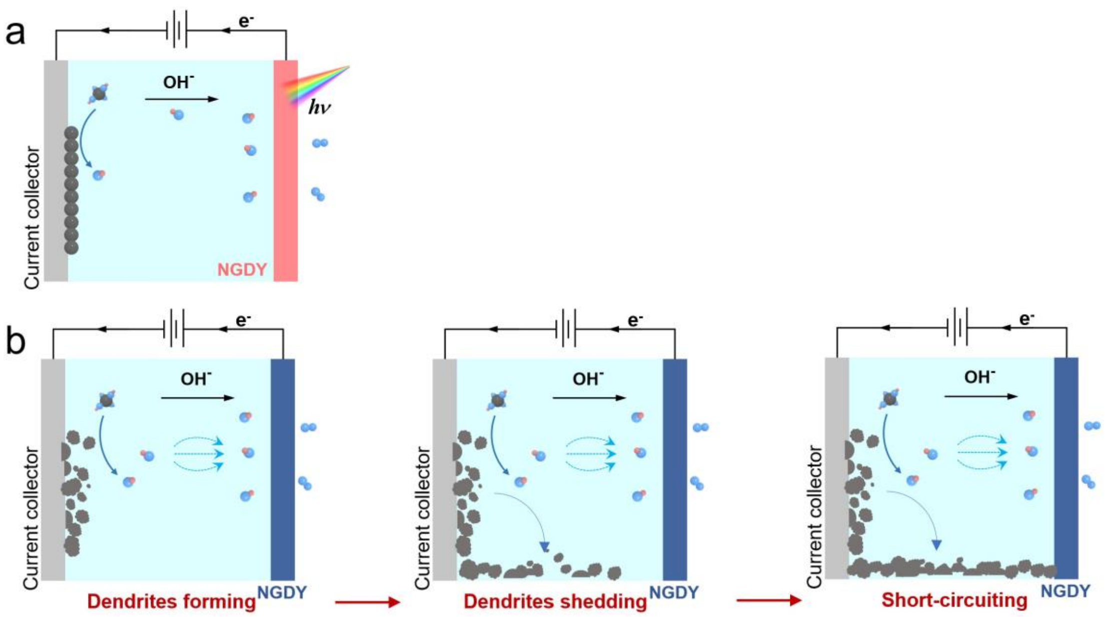

Figure R8. Schematic illustration of deposition mechanism of zinc during charging (a) under light irradiation and (b) in the dark.

7. According to Fig. 5e, the reaction potential energy barrier at the C_3 site is significantly lower than that at the C_2 site, regardless of whether the C_2 site is excited or not. This is contrary to the author's assertion that the C_2 site is the active site for catalyzing the OER process.

Response. We thank the reviewer for identifying this error in our original manuscript

regarding the assignment of the OER active site. Based on our calculated Gibbs free energy diagrams (Fig. 5e), the C_3 site possesses a lower theoretical overpotential for the oxygen evolution reaction (OER) compared to the C_2 site. Our initial statement incorrectly identifying C_2 as the primary OER site was an error in textual description and figure annotation.

To clarify and correct this:

1. The C_3 site is the calculated active site for the OER.
2. Concurrently, the C_2 site is identified as the active site for the oxygen reduction reaction (ORR).
3. We have recalculated and now provide the OER free energy diagram for the C_3 site under photoexcitation in the revised figure, which confirms a significant reduction in the reaction barrier upon light excitation, reinforcing our core mechanistic claim.

We have thoroughly revised the manuscript to correct all relevant text, the caption for Fig. 5e, and the associated discussion to accurately reflect that the C_3 site is the primary active site for catalyzing OER (Figure R9).

The Gibbs free energy of OER at different sites in NGDY indicates that the C_3 site is the active site for catalyzing OER (Fig. 5e). In the excited state, due to the generation of photo-generated holes at the C_3 site under photoexcitation, the free energy for catalyzing OER is further reduced.

The central conclusion of our work—that photoexcitation dynamically reduces the critical kinetic barriers at the relevant active sites—remains fully supported by the corrected data. We appreciate the reviewer's meticulous review, which was essential for ensuring the accuracy of this key point.

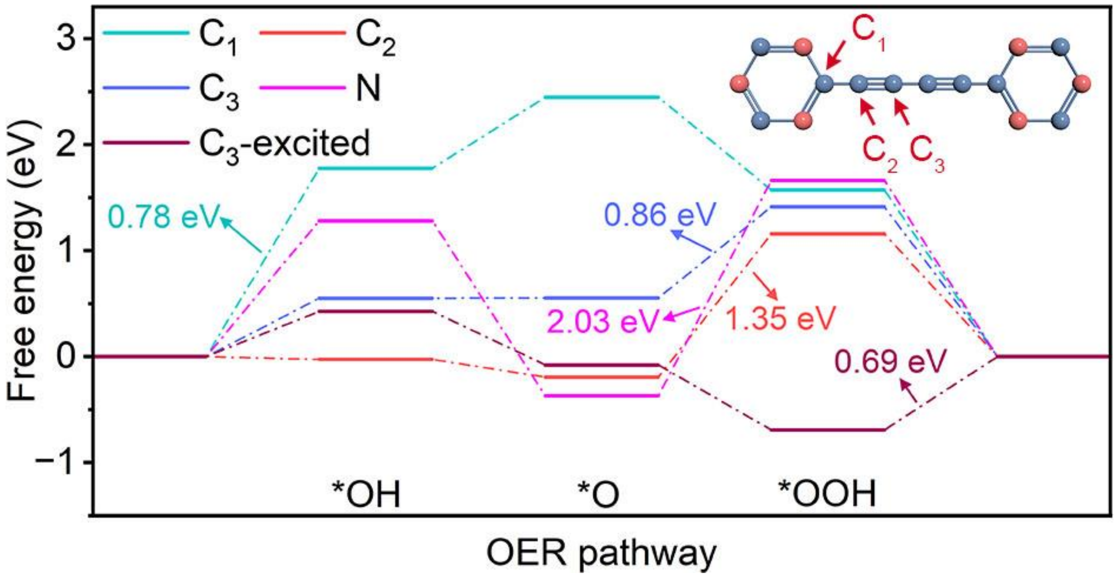

Figure R9. Free energy diagrams of different sites for OER.

8. In the in-situ Raman test, the charging and discharging curves of the battery are missing, which makes the pictures difficult to understand. Let's assume that the bottom-up approach represents the battery's discharge-charging process. Then, at the base state of the battery (i.e., the lowest position of the in-situ Raman spectrum), why are there peaks belonging to Li_2O_2 and LiO_2 present?

Response. We apologize for the lack of clarity in our initial description. The in-situ Raman test for the PLOB was conducted over a complete discharge-charge cycle. The experiment proceeded as follows: Starting from an initial state of 2.8 V, the battery was discharged to 1.6 V (spectral evolution shown in the lower portion of Fig. 5f and 5g). Following this discharge, the battery was charged from 3.0 V to 4.2 V (spectral evolution shown in the upper portion of the same figures). To facilitate readers' understanding and provide clear electrochemical context without altering the original data presentation, we have revised Fig. 5f and 5g by adding arrows on the right side that explicitly indicate the corresponding potential (V vs. Li/Li^+) for key stages of this process. Furthermore, we have revised the "Methods" section to provide a step-by-step account of this testing protocol, ensuring full transparency and reproducibility.

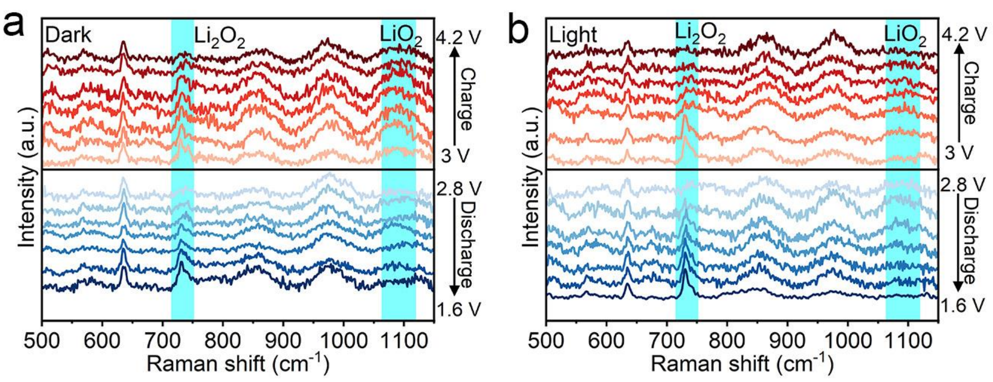

Figure R10. (a) In situ Raman spectra of NGDY-based PLOB in the dark. (b) In situ Raman spectra of NGDY-based PLOB under light irradiation.

Regarding the presence of Li_2O_2/LiO_2 peaks at the spectral "base state":

The "base state" (the lowest position of the in-situ Raman spectrum in Fig. 5f and 5g) corresponds precisely to the end of the discharge process at 1.6 V. At this point, the final discharge product Li_2O_2 is expected and present, which explains its distinct Raman signal.

The key difference observed between the dark (Fig. 5f) and light (Fig. 5g) conditions pertains to the intermediate LiO_2:

i. In the dark, the catalytic activity of the NGDY cathode is not fully activated. Consequently, the intermediate LiO_2 generated during discharge is not completely consumed and remains detectable as a weak signal at the end of discharge.

ii. Under light excitation, the catalytic activity of NGDY is significantly enhanced. This promotes the rapid and efficient conversion of LiO_2 to Li_2O_2 during the discharge process, leaving virtually no detectable LiO_2 signal at the 1.6 V endpoint. This aligns perfectly with the results in Fig. 5f, where subsequent charging from 3.0 V in the dark shows signals for both accumulated Li_2O_2 and residual LiO_2.

In summary, the spectral "base state" reflects the chemical composition at the discharge endpoint, and the presence or absence of the LiO_2 signal at this point provides direct, comparative evidence of the light-enhanced reaction kinetics during the discharge phase itself.

9. In general, the existence of LiO_2 usually means the low charge overpotentials. However, the charge overpotential of NGDY-based PLOB is very high in the dark. It is unreasonable.

Response. The Raman spectra suggests that in the dark, although the LiO_2 intermediate forms, the ground-state NGDY surface lacks sufficient catalytic activity to efficiently catalyze its subsequent decomposition or oxidation. The Raman spectra (Fig. 5f, h) show that LiO_2 (and Li_2O_2) signals persist. This indicates that LiO_2 is essentially "inert" or kinetically stalled on the inactive dark electrode, failing to complete the oxidation pathway and thus resulting in the observed high overpotential. We speculate that the electron-rich diyne (–C≡C–) linkages in NGDY may contribute to this stabilization by weakly coordinating with or donating electron density to LiO_2, rendering it inert on the inactive surface and leading to the observed high overpotential.

In stark contrast, under illumination, the photoexcited NGDY surface becomes highly catalytically active. It not only facilitates the formation of LiO_2, but critically promotes its immediate consumption. This is evidenced by the conspicuously weak and transient LiO_2 signal in the Raman spectra under light (Fig. 5g, h), where both LiO_2 and Li_2O_2 are completely cleared by ~3.8 V. This efficient decomposition pathway is directly responsible for the drastically lowered charge overpotential (as low as 0.05 V in theory, Fig. 5j).

Therefore, the central point is: The mere presence of LiO_2 does not guarantee low overpotential; its fate is governed by the catalyst's activity. Photoexcitation switches NGDY from an inert state to an active one, thereby unlocking the efficient, low-overpotential LiO_2-mediated pathway. The dark-state data provides the essential baseline that highlights this light-triggered transformation.

Upon further reflection, we agree that delving into the specific role and stability of the LiO_2 intermediate is insightful, but introduces a layer of complexity that is not central to proving our core hypothesis. Our primary mechanistic evidence for photo-

enhancement rests on the accelerated formation and, most crucially, the complete decomposition of the final discharge product, Li_2O_2, which is directly and unequivocally observed in both our spectroscopic and electrochemical data.

Therefore, to prevent any potential misunderstanding and to sharpen the narrative, we have revised the relevant discussion in the manuscript. We have chosen to focus squarely on the light-driven kinetics of Li_2O_2, which is the principal contributor to the voltage hysteresis and provides the most direct and unambiguous evidence for the catalytic enhancement of photoexcited NGDY. This refocusing strengthens our central argument without engaging in speculative discussion about an intermediate species whose behavior, while consistent with our data, is challenging to definitively prove in this system.

The corresponding modifications are as follows:

Throughout charge-discharge cycling, the characteristic peak at 730 cm-1 is assigned to the O-O stretch of Li_2O_2, exhibited regular intensity variations, while peaks at 635 cm^{-1} and 850 cm^{-1} were attributed to the electrolyte LiTFSI in TEGDME (Fig. 5f-g). During discharge, the Li_2O_2 peak gradually intensified as the voltage decreased (Fig. 5f and Supplementary Fig. 78). Upon charging, the Li_2O_2 peak continuously decreased. Strikingly, when charging under light irradiation, the Li_2O_2 peak almost completely vanished by 4.0 V (Fig. 5g-h). In contrast, during charging in the dark, peak for Li_2O_2 still persisted at 4.2 V (Fig. 5f and 5h).

The corresponding Figures have also been modified.

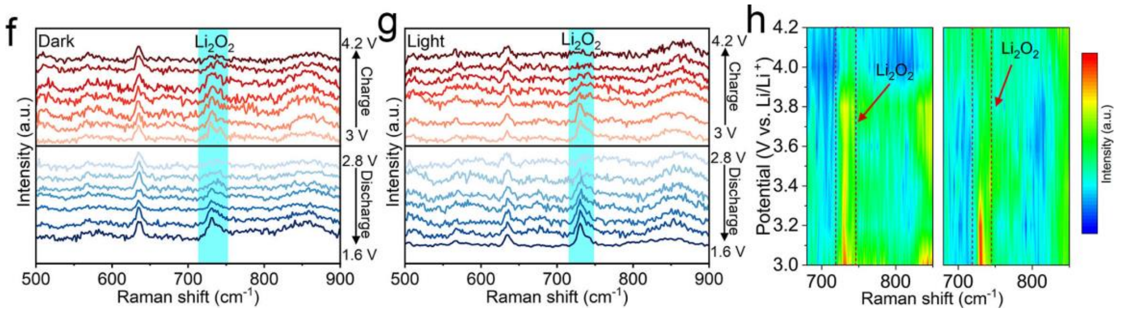

Fig. 5. (f) In situ Raman spectra of NGDY-based PLOB in the dark. (g) In situ Raman

spectra of NGDY-based PLOB under light irradiation. (h) The corresponding 2D color-filled contour plot of the in-situ Raman spectra of NGDY-based PLOB during the charging process (Left: in the dark. Right: under light irradiation).

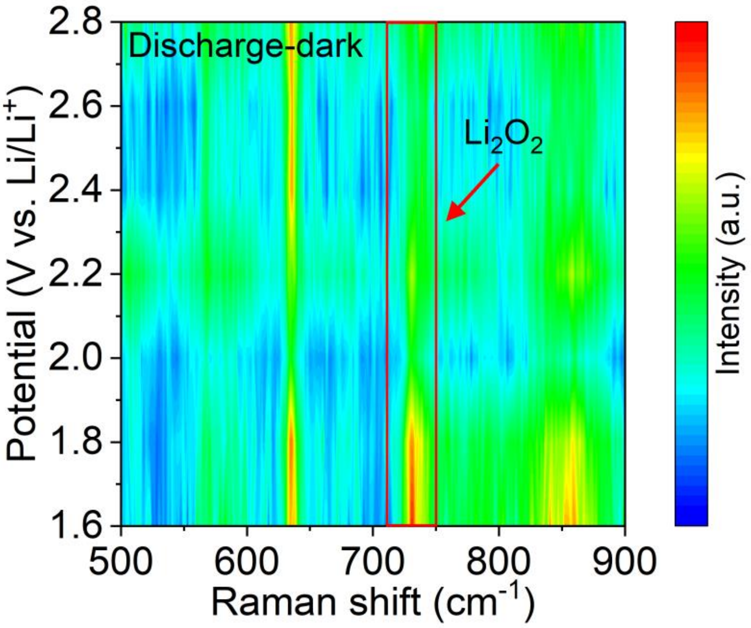

Supplementary Fig. 78. The 2D color-filled contour plot of the in-situ Raman spectra of NGDY-based PLOB during the discharging process in the dark.

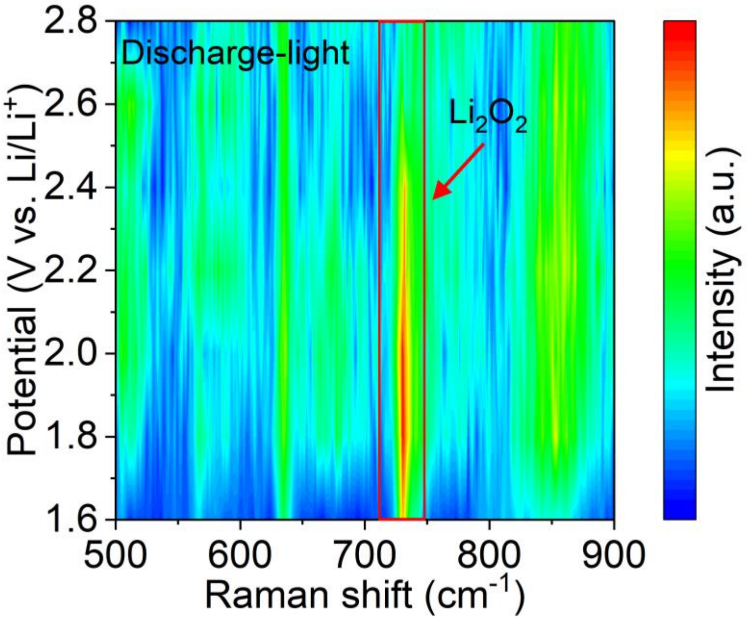

Supplementary Fig. 79. The 2D color-filled contour plot of the in-situ Raman spectra of NGDY-based PLOB during the discharging process under light irradiation.

10. Please detect the gas evolution of NGDY-based PLOB during the discharging process and during the charging process after cycles to confirm its reversibility.

Response. We performed differential electrochemical mass spectrometry (DEMS) to directly monitor gas evolution. The results (Figure R11) clearly show the expected reversible O_2 consumption during discharge and O_2 evolution during charge, with good quantitative correspondence. This provides direct, gas-phase proof that the dominant process is the reversible formation and decomposition of Li_2O_2, as proposed in our mechanism. Figure R11 has been put in our revised Supplementary information.

However, the exceptional long-term electrochemical cycling stability of the PLOB (main text Fig. 4h, >80 h with minimal voltage decay) provides strong indirect evidence that this reversible reaction pathway is maintained over many cycles, as capacity fade and voltage hysteresis would increase significantly if parasitic gas-evolving reactions became dominant.

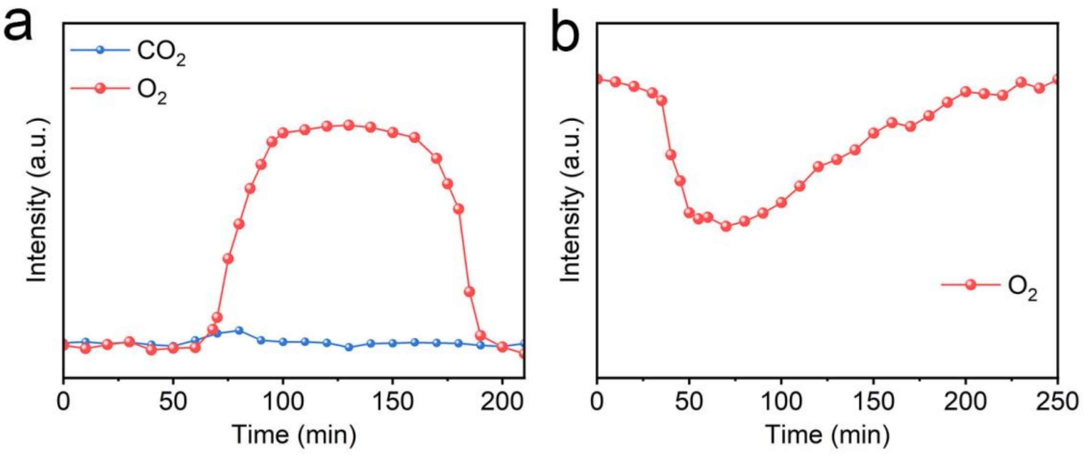

Figure R11. DEMS analyses in the PLOB based on NGDY in discharge (a) and charge (b) processes.

11. Why does the authors choose nitrogen-substituted graphdiyne as the photocathode? How about N-substituted graphene, N-substituted porous carbon and C_3N_4?

Response. We thank the reviewer for those good suggestions, which allow us to clarify the strategic rationale behind our material selection. Excellent photo-electrocatalytic performances have been reported for materials like N-doped carbon material, [1] C_3N_4 [2-7] and C_4N [8]. Our choice of nitrogen-substituted graphdiyne (NGDY) was driven by the goal of establishing and validating a new mechanistic paradigm, rather than solely optimizing for the highest possible initial performance metric.

While the materials mentioned by the reviewer are promising, they predominantly operate within the conventional framework of band excitation in sp^2-carbon or heptazine-based semiconductors, where performance is often optimized through maximizing surface area, conductivity, or defect engineering. However, they lack the well-defined, photoexcitable sp-hybridized carbon (–C≡C–) motifs which are the central subject of our hypothesis. The core scientific question of this work is whether the direct photoexcitation of such specific sp-C units can serve as a novel and effective mechanism for driving electrochemical energy storage.

Therefore, NGDY was selected as the ideal model material to test this hypothesis because its structure provides a crystalline, well-defined platform where sp-C networks are intrinsic, periodic, and integrated with nitrogen heteroatoms. This enabled us to directly observe the sp-C-specific charge transfer dynamics using in situ spectroscopy (e.g., the reversible quenching of the diyne Raman peak at ~2180 cm^{-1} in Fig. 3), which would be impossible in materials without such distinct signatures. It allowed us to distinguish the contribution of the sp-C photoexcitation mechanism from other factors like extreme porosity or exceptionally high conductivity. Moreover. The performance achieved with NGDY—such as the record-low charging voltage and high energy efficiency—validates the "photoexcited sp-carbon" concept. This success establishes a new design principle for photoelectrodes, inviting future exploration into other materials incorporating designed sp-C structures, which may eventually surpass the performance of current state-of-the-art materials.

In summary, we chose NGDY not because it is the only material that can function, but because it is the most structurally definitive material to prove the concept. Its

performance, coupled with the clear mechanistic evidence we provide, opens a new pathway for material design. Future work can certainly explore hybridizing this sp-C photoexcitation concept with the advantageous features of other carbon architectures for further enhancement.

References:

1. Lv, J. *et al.* A photo-responsive bifunctional electrocatalyst for oxygen reduction and evolution reactions. *Nano Energy* **43**, 130-137, (2018).
2. Liu, Y. *et al.* Reducing the charging voltage of a Li–O2 battery to 1.9 V by incorporating a photocatalyst. *Energy Environ. Sci.* **8**, 2664-2667, (2015).
3. Liu, Y. *et al.* Lowering the charge voltage of Li–O2 batteries via an unmediated photoelectrochemical oxidation approach. *J. Mater. Chem. A* **4**, 12411-12415, (2016).
4. Zhu, Z., Shi, X., Fan, G., Li, F. & Chen, J. Photo-energy Conversion and Storage in an Aprotic Li-O2 Battery. *Angew. Chem. Int. Ed.* **58**, 19021-19026, (2019).
5. Zhu, Z. *et al.* Surface plasmon mediates the visible light–responsive lithium–oxygen battery with Au nanoparticles on defective carbon nitride. *Proc. Natl. Acad. Sci. USA* **118**, e2024619118, (2021).
6. Kumar, S. *et al.* Cobalt Diselenide Nanorods Grafted on Graphitic Carbon Nitride: A Synergistic Catalyst for Oxygen Reactions in Rechargeable Li−O2 Batteries. *ChemElectroChem* **5**, 29-35, (2018).
7. Zhu, Z. *et al.* Internal Electric Field and Interfacial Bonding Engineered Step-Scheme Junction for a Visible-Light-Involved Lithium–Oxygen Battery. *Angew. Chem. Int. Ed.* **61**, e202116699, (2022).
8. Fang, Z. *et al.* Capturing Visible Light in Low-Band-Gap C_4N-Derived Responsive Bifunctional Air Electrodes for Solar Energy Conversion and Storage. *Angew. Chem. Int. Ed.* **60**, 17615-17621, (2021).

Reviewer #2 (Remarks to the Author):

The article entitled "Direct solar energy charging applied to photo-coupled electrodes" by Fu et al. discusses the role of nitrogen-substituted graphdiyne (NGDY) in direct solar rechargeable batteries - here in particular different air batteries. The work is detailed with several analyses and correlation, but largely lacking in terms of the newness of the materials or concept - as it has been already demonstrated by several researchers the "effect of light in battery charging" (including metal ion batteries) with different materials. NGDY is also not novel and it has been already shown for various catalytic applications etc. due to its conductivity and activity towards radical stabilisation and hence kinetics tuning. Apart from the novelty, one of the main concerns, effect of temperature in such systems and it is very apparent from the light response (in aqueous vs non-aqueous electrolyte systems) that heat is playing a seminal role in the voltage enhancement. This has been recently studied by different researcher groups such as by Prof Volder's group at Cambridge (ACS Energy Lett. 2023, 8, 11, 4625–4633) and Prof Narayanan's group at TIFR (Nano Lett. 2025, 25, 2, 628–634). This effect is particularly prominent is aqueous batteries and hence needs to be carefully dissected. This has not been done by the researchers here. The obtained different photo voltages in different cases are also establishing this fact. This can not be interpreted as a simple quasi level shifting with the light irradiation. If it is due to the OER energetics differences (not simply photocurrent but voltage), the OER under light and dark is not showing such a difference. Hence, I would rather interpret the enhanced light performance of the batteries as a heat + photocurrent effect rather than a photocharging effect. The authors also need to prove with different energies (wavelength) of light and see how the voltage varies according to the nature of light. There are several typos and also unclear details of experiments including the way the photo charging has been carried out. Hence, overall, I can not recommend the manuscript for publication in a forefront journal like Nature Communication due to lack of novelty.

Response. We sincerely thank the reviewer for the time and insightful comments on

our manuscript. The points raised, particularly regarding the novelty of the concept and the critical need to decouple thermal from photoelectrochemical effects, are essential for strengthening the mechanistic foundation of our work. To address the reviewer's primary concerns comprehensively, we have substantially revised the manuscript with additional experiments, analyses, and clarifications. Our point-by-point response is structured as follows, aiming to resolve the issues the reviewer have raised:

1. Novelty and Core Concept

The general idea of utilizing light in batteries[1-3] and the catalytic properties of NGDY[4,5] have been explored separately, which has also been mentioned in the Introduction section. However, the novelty of our work lies not in simply observing a "light effect," but in advancing the field from a conceptual stage toward practical performance by introducing a new working mechanism and a rationally designed material platform.

We have been following the research progress of photo-coupled metal-air batteries. Despite the possible future widespread coverage, photo-coupled metal-air batteries are still at the conceptual stage and require significant effort to meet practical performance requirements. The currently reported photo-coupled metal-air batteries mainly exhibit the following characteristics:

i. **Structural and Material Complexity.** The design of the structure and material of the working electrode for reported photo-coupled metal-air batteries are complicated. Many studies have used two independent photocathodes composed by separate ORR and OER functional electrodes.[6-8] This will lead to complex configurations and operations, which are impractical for long-term cyclic testing and practical applications. Some studies use a mixture of active materials and photocatalysts to prepare electrodes with different components,[9-11] where each material only has a single function. The consequence of this design is the complexity of the material structure.

ii. **Insufficient Practical Performance.** Currently, there are still many obstacles to the performance of photo-coupled metal-air batteries. most reported batteries

exhibit poor rate capability (operating current between 0.01 - 0.5 mA cm^{-2}) and short lifespan under light illumination,[2,6,9] due to the limited light absorption of photocatalysts, severe carrier recombination, and poor electrocatalytic activity. Therefore, the rationally designed photoelectrodes are crucial for effective charge transfer and separation.[3,13]

iii. **Lack of In-depth Mechanistic Understanding.** The working mechanisms of photo-coupled metal-air batteries has not been thoroughly studied. Critical questions remain regarding how light influences thermodynamic and kinetic behaviors as well as the long-term stability of the batteries, and how to better understand the specific reaction pathways of photocatalysts at the atomic scale. These are all unresolved challenges, making rational design difficult.

iv. **Overlooked Practical Factors.** Crucial evaluation metrics for solar energy utilization (e.g., power conversion efficiency, PCE) and the systematic management of parasitic effects like heating are frequently not addressed comprehensively.[14-16]

The advantages and innovations of this work: we break from the traditional paradigm by introducing nitrogen-substituted graphdiyne (NGDY) as a metal-free photocathode, of which the active sites are dynamically reconfigured via direct photoexcitation of sp-hybridized carbon domains (Figure R12). Unlike conventional semiconductors, NGDY exhibits light-induced electron redistribution that transforms the catalytic centers themselves, generating transient highly-active sites. This method greatly simplified the structure of the photo-coupled metal-air battery, optimized the performance, especially realized and demonstrated the efficient way to charge batteries from solar energy. Notably, we showed in our work that NGDY-based photo-coupled cathodes enable universally superior performance across multiple metal–air systems, including Zn–air, Li–O_2, Mg–air, Fe–air, and Al–air batteries.

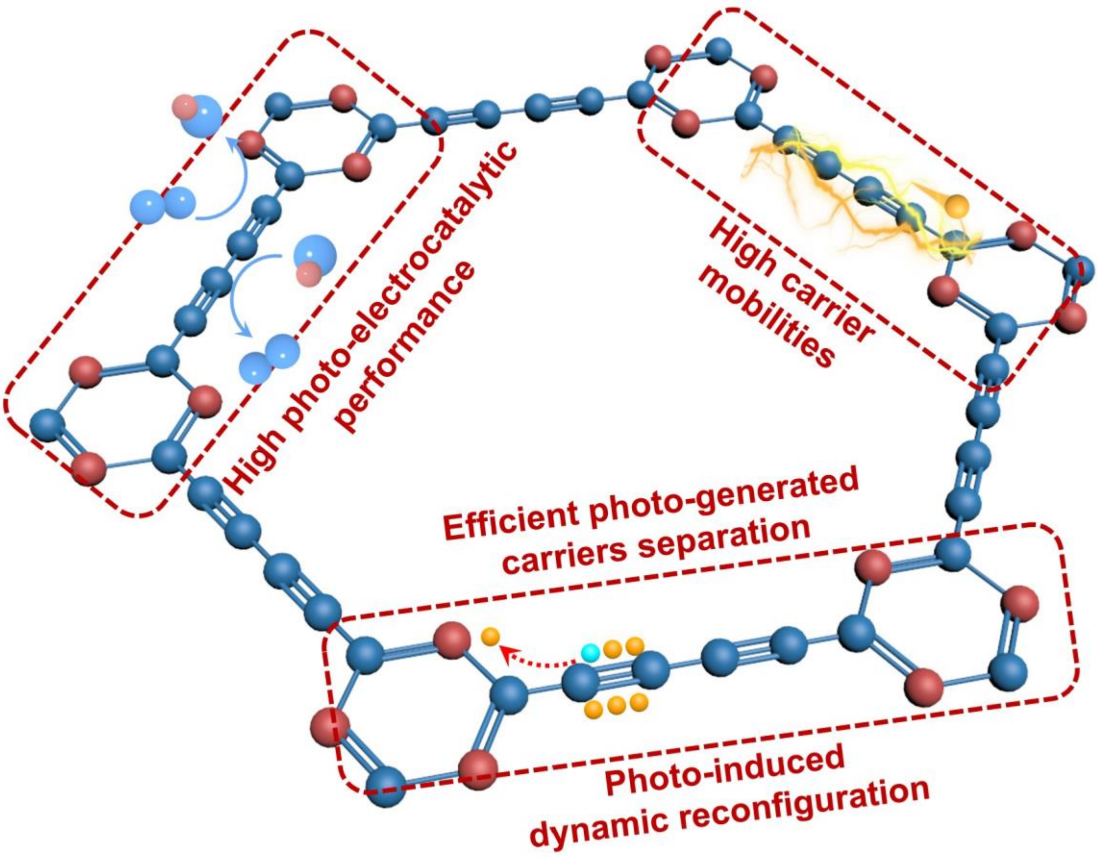

Figure R12. Schematic diagram of the advantages of NGDY.

i. **Novel Material Function and Simplified Design via a Single-Component Photocathode.** Unlike complex composites or dual-electrode designs, we engineered a single, metal-free material to function simultaneously as an efficient light harvester, a charge separator, and a bifunctional (ORR/OER) electrocatalyst. This integrated multifunctionality dramatically simplifies the device architecture while enabling exceptional performance.

ii. **Introduction and Validation of a New "Photoexcited sp-Carbon" Operating Mechanism.** This is the **core conceptual** novelty. While NGDY has been used in catalysis, we are the first to leveraging the direct photoexcitation of its sp-hybridized carbon (sp-C) domains to dynamically reconfigure catalytic active sites in real-time. This mechanism, distinctly different from using light merely to generate carriers that migrate to static sites, is experimentally proven by our in situ spectroscopic observations of intramolecular charge transfer (Fig. 3). It provides a

new design principle for photoelectrodes: using light to program the catalyst's electronic state.

iii. **Breakthrough Performance Demonstrating Practical Potential.** Our NGDY-based photo-coupled cathodes deliver performance parameters that address the limitations above. PZAB based on NGDY achieves a low charging voltage of 1.33 V and an energy efficiency of 96.9%, and demonstrates exceptional cycling stability over 230 cycles at an ultra-high current density of 100 mA cm^{-2}, a regime where most prior systems fail. This performance enhancement is demonstrated across five different metal-air systems (Zn, Li, Mg, Fe, Al), proving the mechanism targets the universal oxygen electrode chemistry. Moreover, we show that the photo-enhanced kinetics lead to dendrite-free metal plating, addressing a key safety and longevity concern in metal batteries.

iv. **Comprehensive Mechanistic and System-Level Analysis.** Beyond reporting performance, we provide a deep mechanistic study through operando spectroscopy (Fig. 5) and DFT calculations (Figs. 2, 5), elucidating the "light-defined" reaction pathways.

2. Systematic Analysis and Exclusion of Thermal Effects

We have conducted a series of targeted experiments and analyses that systematically exclude photothermal heating as the dominant contribution, thereby confirming that the performance enhancement stems from the intrinsic photoelectrochemical response of NGDY.

2.1. Purposeful Experimental Design to Minimize Thermal Effects

We were aware of this potential influence from the outset; however, as our core focus was on elucidating a new photoelectrochemical mechanism, we regrettably under-reported these essential experimental details, which led to understandable confusion. Our experimental design intentionally incorporated specific measures to isolate and minimize photothermal contributions:

i. **Spectral Filtering**: In all photo-coupled battery tests, an 800 nm infrared filter was

employed. As illustrated in the Figure R13, this filter drastically attenuates the intense infrared emission band (800-1000 nm) of the xenon lamp, the primary source of radiative heating.

ii. **Temperature Control**: A cooling system with electrolyte circulation was used to maintain the battery temperature at room temperature, preventing bulk thermal effects.

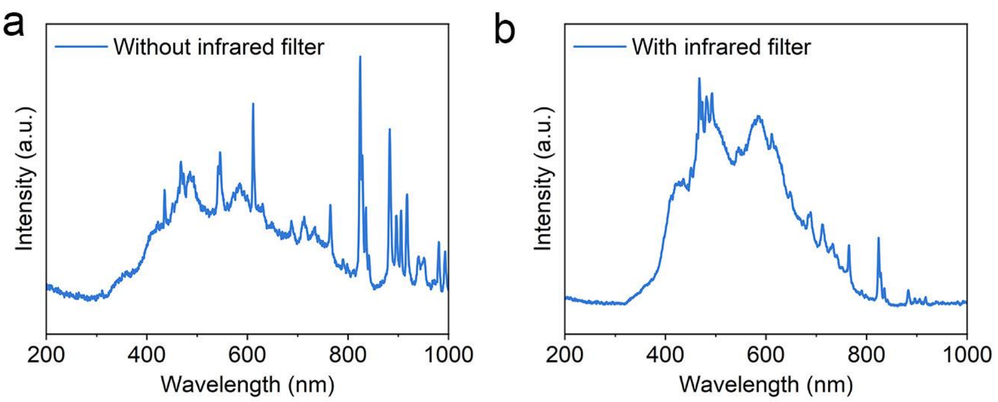

Figure R13. The output spectrum of the xenon light source (a) without infrared filter and (b) with infrared filter.

The critical importance of this spectral filtering is directly demonstrated by the control experiment the reviewer referenced. As shown in Figure R3. Without the IR filter, the bare carbon cloth substrate exhibits a weak, transient current attributable to photothermal artifacts. With the IR filter in place, this parasitic signal from carbon cloth is suppressed to a negligible level. Crucially, under the same filtered illumination, the stable and significant photocurrents from GDY and NGDY remain strongly evident. This clear contrast confirms that the photo responses of our materials are intrinsic photoelectrochemical signals, distinct from and not dominated by photothermal effects. The combination of spectral filtering and active temperature control formed the foundational, controlled environment for all our battery performance evaluations.

2.2. temperature effect

We conducted a direct comparison by externally heating the battery in the dark. While

elevated temperature can generally lower activation barriers and accelerate ion transport, it also risks promoting detrimental interfacial side reactions.[17] In our system, the active cooling and electrolyte circulation maintained the operational temperature near 25°C during all light tests, ensuring that any bulk heating was not a variable.

To isolate the effect of temperature, we evaluated the Zn-air and Li-O_2 battery performance under dark conditions at controlled temperatures of 25°C and 60°C. For the Zn-air battery, external heating to 60°C induced only a modest improvement in the charging curve (Figure R14a). For the Li-O_2 battery, CV curves at 60°C show a slight increase in current density but no fundamental change in the reaction profile compared to 25°C (Figure R14b). The key finding is consistent across systems: the magnitude of improvement from heating alone is minor. It is fundamentally distinct from the dramatic reduction in charging voltage and overpotential achieved under light illumination at 25°C.

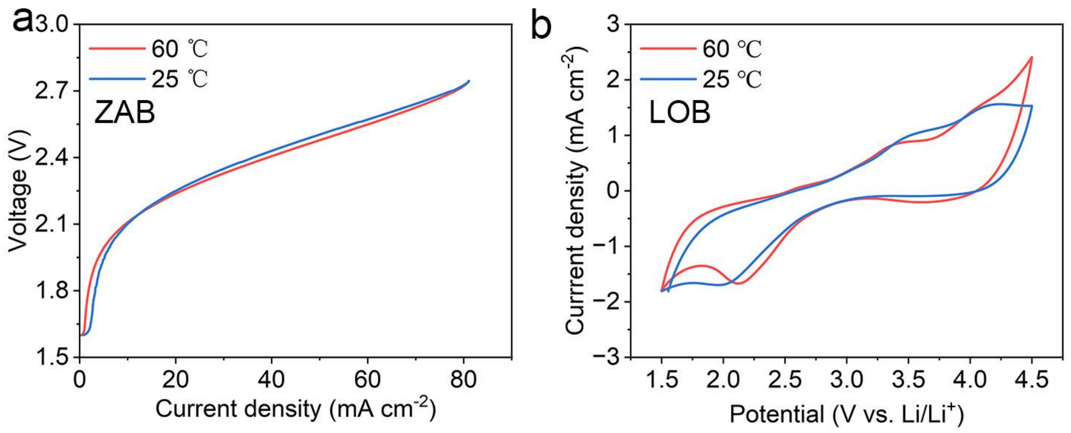

Figure R14. (a) Charge polarization curves of PZAB based on NGDY at 25°C and 60°C. (b) CV curves of PLOB based on NGDY at 25°C and 60°C.

This controlled comparison provides direct evidence that the substantial voltage drop and efficiency gains reported in our work are not a consequence of incidental heating. Instead, they are driven by the specific photoelectrochemical mechanism—the light-induced electronic excitation of NGDY.

2.3. Wavelength-dependent studies

To unequivocally demonstrate that the performance enhancement originates from the

intrinsic photoelectronic properties of NGDY—rather from a broadband thermal effect—we performed a critical wavelength-dependence study. A generic photothermal mechanism would predict a spectral response that correlates with the infrared absorption and heating efficiency of the system, typically increasing towards longer wavelengths.

We systematically measured the charging performance of the photo-coupled Zn-air battery under monochromatic illumination at equal light intensity (5 mW cm^{-2}), using narrowband filters for wavelengths from 380 to 600 nm (Figure R15a).

The results reveal a response pattern that is inconsistent with a photothermal origin but aligns perfectly with a photoelectronic mechanism:

i. The reduction in charging voltage and the calculated power conversion efficiency (PCE) show a pronounced, sharp maximum at 400 nm (PCE = 2.92%).
ii. The plots of PCE versus wavelength are closely mirrors the optical absorption profile of NGDY (Figure R15b), not a monotonically increasing function towards the infrared.

To further substantiate the photoelectrochemical origin of the enhancement, we investigated the Li-O_2 battery under monochromatic illumination. As presented in Figure R16, the energy efficiency of the charging process exhibits a strong dependence on the incident wavelength, culminating in a sharp maximum of 93.1% at 400 nm. This distinct action spectrum aligns precisely with the characteristic optical absorption edge of NGDY (Fig. 2b). The observation of such a material-specific, resonant efficiency peak provides definitive evidence that the performance gains are governed by the intrinsic electronic excitation of NGDY.

The observed peak at 400 nm signifies a resonant light-matter interaction where the photon energy optimally matches the energy required for charge excitation within the NGDY framework. This specific, narrow-band resonance is a hallmark of a photoelectrochemical process governed by the material's electronic band structure. In contrast, a thermal-dominant response would not produce such a distinct peak at the

material's specific absorption edge but would show a broader or different spectral dependence.

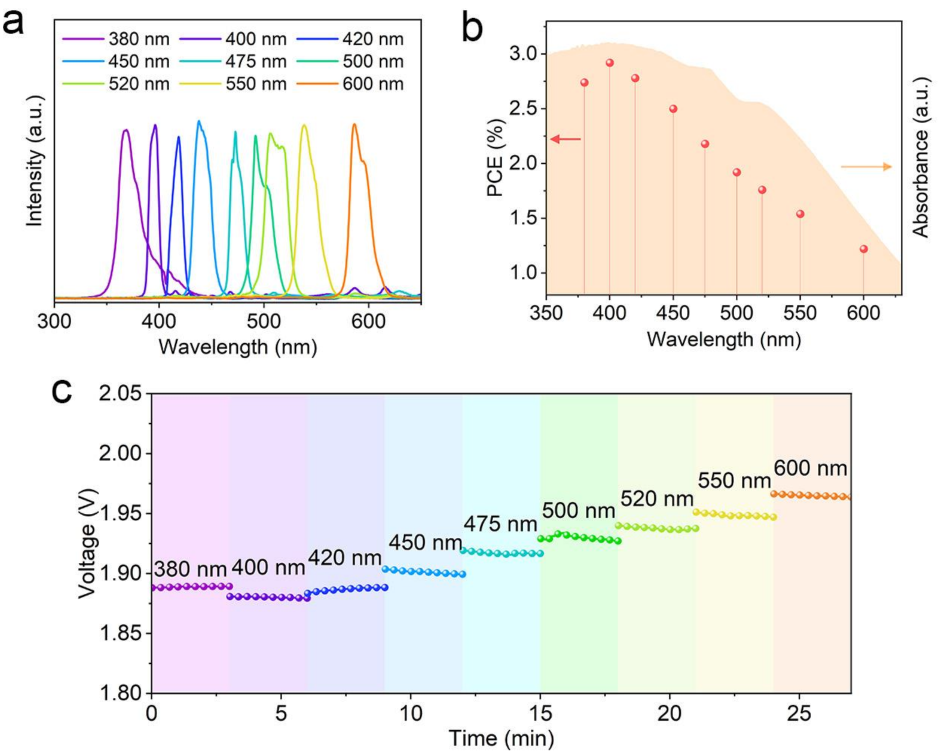

Figure R15. (a) The output spectrum of different wavelengths of light. (b) The PCE at different wavelengths of light (The background refers to the absorbance of NGDY). (c) Charging curves of PZAB based on NGDY at different wavelengths of light (Light intensity: 5 mW cm^{-2}; current density: 1 mA cm^{-2}).

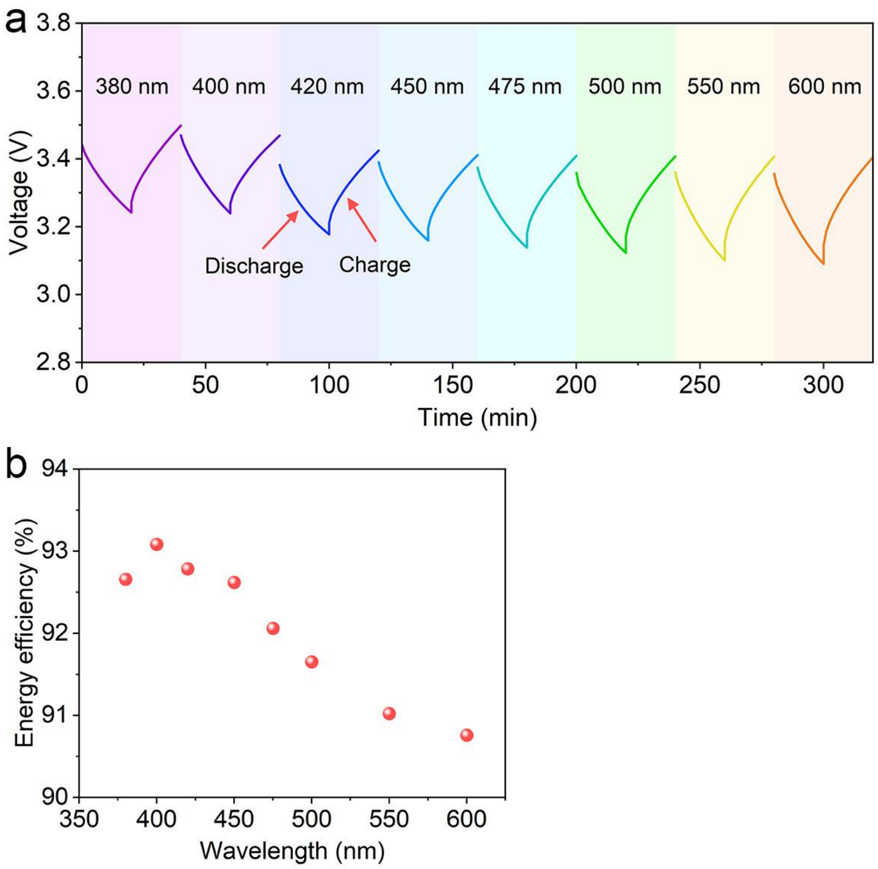

Figure R16. (a) Discharging and charging curves of PLOB based on NGDY at different wavelengths of light (Current density: 0.01 mA cm^{-2}). (b) The energy efficiency at different wavelengths of light.

Therefore, this definitive correlation between the battery's functional output and the material's fundamental absorption characteristic provides conclusive evidence that the enhancement is a genuine, material-specific photoelectrochemical effect, robustly supporting our proposed mechanism of light-driven activity centered on the photoexcitation of sp-C domains.

To directly address whether photoexcitation modifies the OER kinetic barrier, rather than merely adding a photocurrent or thermal effect, we performed detailed Tafel analysis under controlled temperature conditions (25 °C, using a thermostated electrochemical cell to eliminate heating effects).

The Tafel slopes decreased form 196.5 mV dec^{-1} in the dark to 175.3 mV dec^{-1} under

light irradiation (Figure R17), which is a clear indicator of accelerated OER kinetics. The decrease in Tafel slope signifies a lower energetic barrier for the rate-determining step and reflects a change in the reaction pathway facilitated by photoexcitation. Importantly, such a change in Tafel slope cannot be explained by a simple thermal effect or an additive photocurrent, as a purely thermal contribution would typically shift the entire polarization curve to lower overpotentials but would not substantially alter the Tafel slope. And a mere photocurrent addition would increase the current at a given potential without modifying the kinetic parameter (b value).

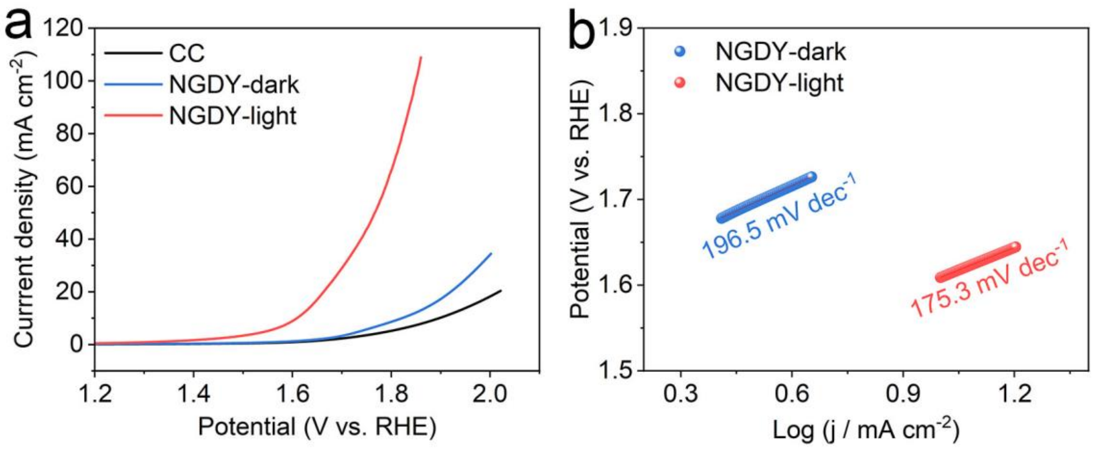

Figure R17. (a) LSV curves of CC and N-GDY for OER with or without illumination and (b) the corresponding Tafel slopes.

Therefore, the observed lowering of the Tafel slope provides direct electrochemical evidence that light reshapes the OER energetics on NGDY, consistent with our proposed mechanism of photoinduced charge redistribution and transient active-site generation.

This kinetic evidence complements our other findings that in-situ Raman spectroscopy shows earlier emergence of OER intermediates (*OOH, *O) at lower potentials under light (Fig. 5) and that DFT calculations reveal strengthened intermediate adsorption and reduced stepwise energy barriers in the excited state (Fig. 5e). Together, these data substantiate that the performance enhancement stems from a genuine photocatalytic modification of OER kinetics, not merely from thermal or photocurrent contributions. We have incorporated this explanation into the revised Supplementary information.

3. Improving Experimental Clarity and Text

We have thoroughly revised the manuscript to address this concern, with a focus on the "Methods" section, to ensure all procedures are described with sufficient detail for reproducibility.

Key improvements include:

i. **Detailed Illumination Parameters:** We have expanded the description of the light source, specifying the model (300 W xenon lamp), the use of an 800 nm long-pass filter to minimize infrared heating, the calibration of light intensity (500 mW cm^{-2}) using a photo radiometer, and the method to ensure spatial uniformity.

ii. **Clarified Battery Assembly and Testing:** The procedures for assembling all photo-coupled metal-air batteries (Zn-air, Li-O_2, Mg-air, Fe-air, Al-air) have been detailed, including specifications for electrodes, electrolytes, separators, and the configurations for operando tests (e.g., use of quartz windows).

iii. **Enhanced Data Presentation Context:** We have added necessary context to figures. For instance, the corresponding voltage-time profile has been added alongside the operando Raman contour plots (Fig. 5h), and the rationale for starting Raman measurements from a pre-discharged state is now explicitly stated in the caption.

iv. **Comprehensive Proofreading:** The entire manuscript has undergone careful proofreading to correct typographical errors, improve sentence fluency, and ensure consistent use of scientific terminology.

We believe these revisions have significantly enhanced the precision, clarity, and overall professionalism of the manuscript, fully addressing the reviewer's concerns.

References:

1. Li, J., Zhang, K., Wang, B. & Peng, H. Light-Assisted Metal-Air Batteries: Progress, Challenges, and Perspectives. *Angew. Chem. Int. Ed.*, e202213026,

(2022).
2. Liu, X. *et al.* Utilizing solar energy to improve the oxygen evolution reaction kinetics in zinc-air battery. *Nat. Commun.* **10**, 4767, (2019).
3. Xue, H., Gong, H., Yamauchi, Y., Sasaki, T. & Ma, R. Photo-enhanced rechargeable high-energy-density metal batteries for solar energy conversion and storage. *Nano Res. Energy* 1, e9120007, (2022).
4. Wang, M. *et al.* Modulation of electronic structure of metal-free graphdiyne via precise nitrogen modification for oxygen evolution reaction. *ChemPhysMater* 4, 289-295, (2025).
5. Shen, H. *et al.* Nitrogen-doped graphdiyne for effective metal deposition and heterogeneous Suzuki-Miyaura coupling catalysis. *Appl. Catal. A-Gen.* 623, 118244, (2021).
6. Z. Fang *et al.*, Capturing Visible Light in Low-Band-Gap C_4N-Derived Responsive Bifunctional Air Electrodes for Solar Energy Conversion and Storage. *Angew. Chem. Int. Ed.* 60, 17615-17621 (2021).
7. D. Du, S. Zhao, Z. Zhu, F. Li, J. Chen, Photo-excited Oxygen Reduction and Oxygen Evolution Reactions Enable a High-Performance Zn-Air Battery. *Angew. Chem. Int. Ed.* 59, 18140-18144 (2020).
8. L. Kong *et al.*, Realizing Unassisted Photo-Charging of Zinc–Air Batteries by Anisotropic Charge Separation in Photoelectrodes. *Adv. Mater.* 10.1002/adma.202304669 (2023).
9. D. Bu *et al.*, Rechargeable sunlight-promoted Zn-air battery constructed by bifunctional oxygen photoelectrodes: Energy-band switching between ZnO/Cu_2O and ZnO/CuO in charge-discharge cycles. *Chem. Eng. J.* 433, 133559 (2022).
10. J. Lv *et al.*, A photo-responsive bifunctional electrocatalyst for oxygen reduction and evolution reactions. *Nano Energy* 43, 130-137 (2018).
11. Z. Fang *et al.*, Tactile UV- and Solar-Light Multi-Sensing Rechargeable Batteries with Smart Self-Conditioned Charge and Discharge. *Angew. Chem. Int. Ed.* 58, 9248-9253 (2019).
12. H. Xue, H. Gong, Y. Yamauchi, T. Sasaki, R. Ma, Photo-enhanced rechargeable high-energy-density metal batteries for solar energy conversion and storage. *Nano Res. Energy* **1**, e9120007 (2022).

13. Z. Yuan, H. Mao, D. Yu, X. Chen, Photo-Assisted Metal-Air Batteries: Recent Progress, Challenges and Opportunities. *Chem. Eur. J.* **29**, e202202920 (2022).
14. Ola, N. Wang, G. Walker, Y. Zhu, D. Grant, Engineering the next generation of photorechargeable zinc-air batteries. *Curr. Opin. Electroche.* **35**, 101040 (2022).
15. Z. Fang, X. Hu, D. Yu, Integrated Photo-Responsive Batteries for Solar Energy Harnessing: Recent Advances, Challenges, and Opportunities. *Chempluschem* **85**, 600-612 (2020).
16. Y.-X. Tan, X. Zhang, J. Lin, Y. Wang, A perspective on photoelectrochemical storage materials for coupled solar batteries. *Energy Environ. Sci.* **16**, 2432-2447 (2023).
17. Y. Feng et al., Challenges and advances in wide-temperature rechargeable lithium batteries. *Energy Environ. Sci.* **15**, 1711-1759 (2022).

Reviewer #3 (Remarks to the Author):

The manuscript by Li and co-workers presents a new class of photocathode where the catalytic activity is directly governed by light. The authors revealed the unique photoexcitation mechanism of nitrogen-substituted graphdiyne (NGDY), which involves the direct photoexcitation of sp-hybridized carbon domains to dynamically reconstruct the electronic structure, generating transient and highly active catalytic centers. This is fundamentally different from the formation of traditional semiconductors with static active sites. This light-defined catalytic function is confirmed through in-situ spectroscopic techniques, which capture the intramolecular charge transfer and strengthened intermediate adsorption upon illumination. This intrinsic activation enables universally superior performance across Zn-air, Li-O_2, Mg-air, Fe-air, and Al-air batteries. The photo-coupled Zn-air battery achieves a low charging voltage of 1.33 V, an energy efficiency of 96.9%, and exceptional stability over 230 cycles at an ultra-high current density of 100 mA cm-2, while the Li-O_2 system reaches a breakthrough efficiency of 96.3%. The work represents a significant advance

in employing light as a direct tool for engineering catalytic sites, offering a transformative pathway for the next generation of high-efficiency, photo-coupled energy systems. Therefore, I recommend publishing this paper after addressing the following issues.

Response: We sincerely thank the reviewer for their positive assessment of the novelty and mechanistic insight of our work. All constructive suggestions have been carefully addressed in the revised manuscript. We appreciate their valuable time and feedback, which have strengthened the final paper.

1. The authors propose the core highlight of light-induced dynamic reconfiguration of high-activity sites, which is different from the conventional approaches that rely on photo-generated holes as static sites. But the essential difference between this dynamic reconfiguration and the mechanism of static sites needs to be explained more clearly.

Response: The fundamental distinction lies in the object and consequence of photoexcitation. In conventional semiconductor photoelectrodes, light generates mobile charge carriers (holes and electrons) that migrate to pre-existing, static catalytic sites (e.g., metal cations or defects). The electronic structure and intrinsic activity of these sites remain unchanged; the holes simply act as external oxidants. The performance is limited by carrier recombination and the fixed activity of these static sites.

In stark contrast, for NGDY, the sp-hybridized carbon are the catalytic sites. Photoexcitation does not just generate mobile holes, but also causes an instantaneous, intramolecular redistribution of electrons within the site itself, as directly evidenced by the reversible N 1s XPS shift and the quenching of the diynyl Raman signal (Figure 3). This light-induced electronic reconfiguration transiently alters the very nature of the active center, optimizing its adsorption properties (as shown by DFT, Figure 5) and intrinsic catalytic capability. Thus, enhancement stems not from delivering energy to a static site, but from using light to dynamically reprogram the site's reactivity in real

time.

We have incorporated this explanation into the revised manuscript as follows:

The fundamental distinction of our system lies in the object of photoexcitation. In conventional photoelectrodes, light generates charge carriers that migrate to static, pre-existing active sites without altering their intrinsic electronic structure. In contrast, in NGDY, the sp-C moieties themselves are the active sites; photoexcitation directly and reversibly reconfigures their electronic structure, thereby dynamically optimizing their intrinsic catalytic properties in real time.

2. Although the authors show the stability of the electrode morphology after cycling, further characterization is needed to investigate the possible changes in the chemical structure of the electrode after cycling.

Response: The structural stability was evaluated by SEM, EDS and XPS. After the long cycle, the morphology of NGDY remains intact without any apparent degradation or aggregation (Supplementary Fig. 32). Moreover, the EDS mapping shows that the elements C and N are still evenly distributed (Supplementary Fig. 33), indicating the structural stability of N-GDY.

XPS was performed before and after the battery cycling tests (Figure R18). The C 1s spectra demonstrate that the core chemical structure of the N-GDY framework was not disrupted. The primary change observed was a slight change in N protonation, which suggests a benign surface adaptation to the electrochemical environment. Moreover, the atomic concentration of oxygen remained highly stable, with only minimal variations, and affirming the absence of significant oxidation or corrosion. The combined evidence from morphology, elemental distribution, and chemical bonding analysis unequivocally demonstrates the structural stability of the NGDY photo-coupled electrode under realistic working conditions. Figure R18 has been put in our revised Supplementary information.

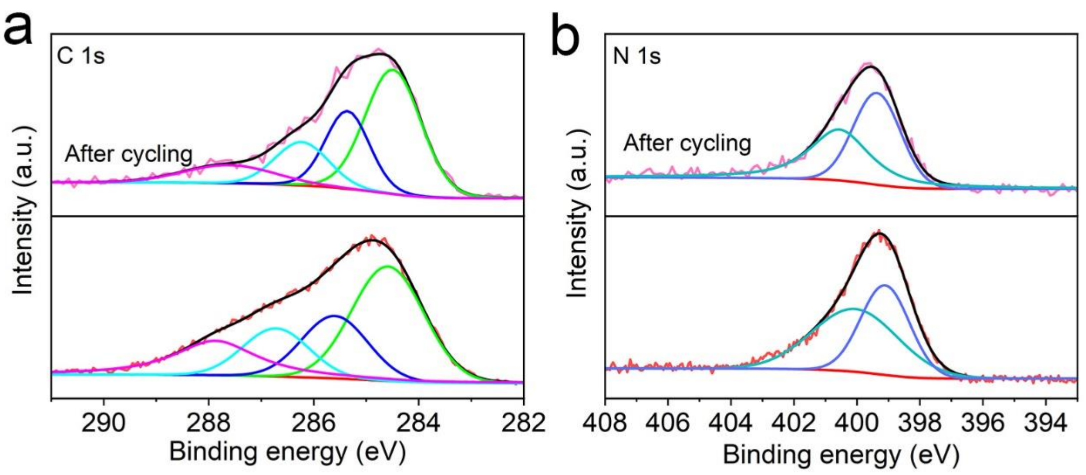

Figure R18. XPS (a) C 1s spectra and (b) N 1s spectra of NGDY before after battery cycling tests.

3. PZAB based on photo-excited NGDY exhibits better stability than commercial Pt/C+IrO_2. This is a key and attractive application advantage, but the root of the enhanced stability under photo-excitation needs to be further explored.

Response: This significantly enhanced stability originates from the fundamentally milder operating conditions enabled by photoexcitation, which mitigates key degradation pathways. The core is the drastically reduced charging overpotential. By supplying photon energy to directly drive the oxygen evolution reaction (OER) at the NGDY cathode, the required electrical charging voltage is lowered substantially (e.g., to 1.33 V). This means the entire battery operates in a more moderate electrochemical window, close to its thermodynamic equilibrium. Consequently, this minimizes high-voltage-driven side reactions, such as electrolyte decomposition and oxidative corrosion of the carbon-based electrode, which are major causes of capacity fade and electrode deterioration in conventional systems.

Furthermore, the kinetic benefit extends to the Zn anode. The efficient charge transfer and lower overall cell voltage under illumination promote a more homogeneous and controlled Zn plating/stripping process (Supplementary Fig. 36 to 37). This is directly evidenced by the smooth, dendrite-free Zn morphology observed after cycling under

light, contrasting with the severe dendrite formation in the dark. Therefore, the improved stability is not merely a surface passivation effect but a system-level consequence of photoexcitation optimizing reaction thermodynamics and kinetics at both electrodes, leading to more reversible electrochemistry.

We have incorporated this explanation into the revised manuscript as follows:

The significantly enhanced stability originates from the milder operating conditions brought about by photo-excitation. Specifically, the lower charging overpotential reduces the degradation of the high-voltage driving electrode/electrolyte in the cathode, while promoting a uniform, dendrite-free zinc coating on the anode.

4. The authors summarized the performance of PZAB and photo-coupled Mg-air batteries, but it lacks a summary of the performance of solid PZAB, PLOB, etc., which needs further supplementation.

Response: We have further supplemented the summary of the performance of solid PZAB, PLOB, and added it to the revised supplementary information.

Table R1. Summary of charge voltage, and power conversion efficiency of NGDY based photo-coupled solid PZAB at different current densities.

J (mA cm^{-2})	Charge voltage in dark (V)	Charge voltage in light (V)	PCE (%)
0.1	1.86	1.19	0.013
0.5	2.02	1.23	0.079
1	2.10	1.31	0.158
5	2.47	1.98	0.49
10	2.78	2.19	1.18

Table R2. Summary of discharge/charge voltage, voltage gap and energy efficiency of NGDY based PLOB at different current densities.

J (mA cm^{-2})	Discharge/ charge voltage (V)	Voltage gap (V)	Energy Efficiency (%)
0.01	3.13/3.25	0.12	96.3
0.1	3.28/3.67	0.39	89.4
0.2	3.08/3.69	0.61	83.5
0.5	2.35/3.38	1.03	69.5

5. Although in situ Raman spectroscopy revealed the evolution of charge and discharge products during the PLOB cycling process, more direct evidence (e.g., XPS, XRD) is needed to prove the structure of the discharge products.

Response: We have performed additional XPS and XRD characterizations on the NGDY cathodes harvested after specific states of charge/discharge (Figure R19 and Figure R20). The O 1s XPS spectrum of the discharged cathode shows a distinct peak at 530.85 eV, characteristic of Li_2O_2. This peak disappears after charging, with the spectrum reverting to show the C-O peak of the NGDY substrate. Correspondingly, the Li 1s spectrum of the discharged electrode displays a main peak at 55.83 eV (Li_2O_2), alongside minor peaks for Li–C and Li–F species. After charging, the Li 1s signal diminishes significantly, indicating the removal of lithium species from the cathode. Furthermore, ex situ XRD analysis of the discharged cathode reveals a new diffraction peak at approximately 35°, which is indexed to the (010) crystal plane of Li_2O_2. This peak is absent in the pristine and charged electrodes. Collectively, these results from XPS and XRD offer direct and complementary evidence that the primary discharge product is Li_2O_2. We have added this discussion and the corresponding data to the revised supplementary information.

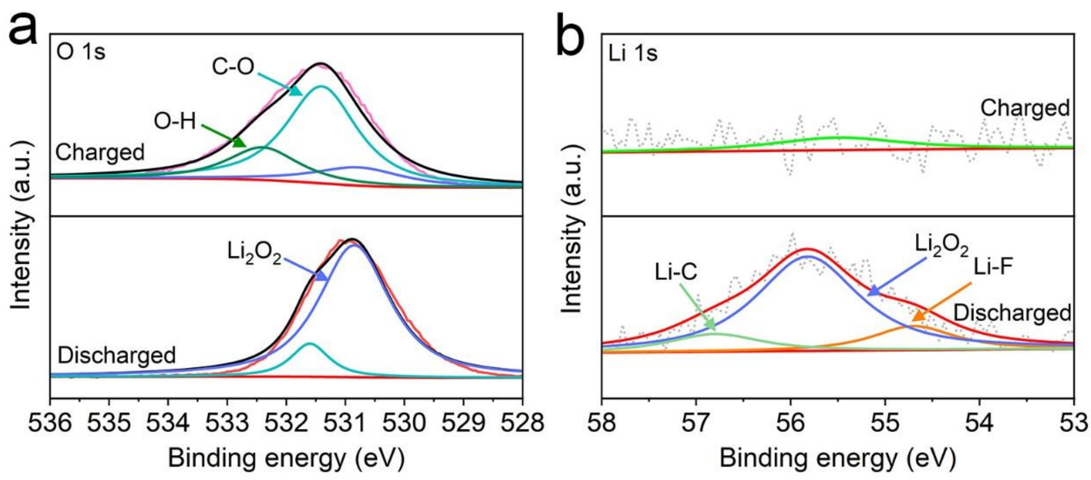

Figure R19. XPS (a) O 1s spectrum and (b) Li 1s spectrum of charged and discharged NGDY.

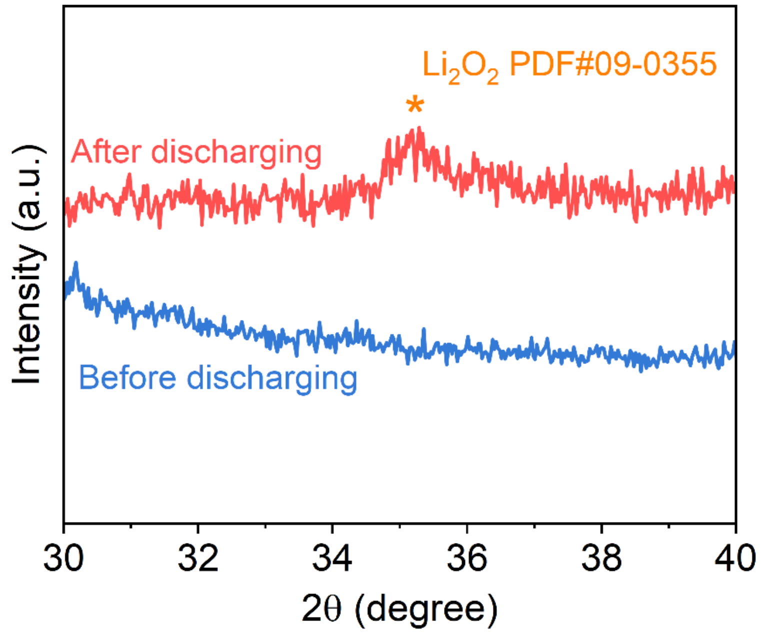

Figure R20. XRD spectrum of NGDY before discharging and after discharging.

6. A more detailed description of the parameter information for experimental devices (such as battery assembly, in situ Raman spectroscopy) should be provided to offer readers clearer guidance.

Response. We revised the "Methods" section and added new supplementary data to

provide clear and step-by-step guidance on all key experimental setups, especially the specific parameters for battery assembly and testing, as well as the specific parameters for in-situ Raman characterization.

A 300 W xenon lamp (PLS-SXE 300, Perfectlight, China) served as the full-spectrum light source. For all photo-coupled battery tests, an 800 nm infrared filter (to attenuate infrared heating) was employed to tailor the incident spectrum to the ~320–800 nm range. The light intensity at the sample position was calibrated to 500 mW cm^{-2} using a photoradiometer (CEL-NP2000, Ceaulight).

To accurately assess the long-term operational stability, zinc deposition morphology evolution, and, crucially, the Coulombic efficiency (CE) of the Zn-air batteries at a high current density of 100 mA cm^{-2}, a specific testing protocol employing a copper foil as the anode current collector was adopted. This method replaces the conventional zinc foil and allows for the direct evaluation of zinc plating/stripping reversibility per cycle. The procedure is as follows: a polished copper foil was assembled as the working electrode (anode). In each cycle, the battery was first charged at a constant current density of 100 mA cm^{-2} for 60 s to deposit zinc onto the copper substrate. Subsequently, it was immediately discharged at the same current density until the deposited zinc was completely stripped, indicated by the cell voltage dropping to approximately 0 V, and the discharge time was recorded. The CE for each individual cycle was then calculated using the formula:

$$CE = (t_{discharge} / t_{charge}) \times 100\% \quad (4)$$

In-situ Raman characterization

In-situ Raman characterization was conducted using a customized electrochemical cell equipped with a quartz optical window to allow laser access and, where applicable, simultaneous light irradiation. A Horiba confocal Raman microscope with a 532 nm laser was used for all measurements. For experiments under photoexcitation, light from a 300 W xenon lamp (CME-X305, Microenerg Beijing Technology Co., Ltd) was focused onto the working electrode surface through an optical fiber.

For the Zn-air battery measurements, the anode consisted of a polished zinc foil, and the cathode was the NGDY grown on carbon cloth (NGDY/CC). The electrodes were separated by a glass fiber membrane (Whatman), and 150 µL of electrolyte (6 M KOH + 0.2 M zinc acetate) was added. The cell was connected to a CHI 660E electrochemical workstation. Raman spectra were collected sequentially while charging the battery from 1.6 V to 2.4 V, with a spectrum acquired at every 0.1 V interval.

For the Li-O_2 battery measurements, the cell comprised a lithium foil anode, a NGDY/CC cathode, and a glass fiber separator soaked with 150 µL of electrolyte (1 M LiTFSI in TEGDME). The cathode was sealed against a quartz window, and the cell body was connected to an O_2 gas bag. The electrochemical protocol involved a full cycle: first, discharging from 2.8 V to 1.6 V, followed by charging from 3.0 V to 4.2 V. Raman spectra were collected at 0.2 V intervals throughout both the discharge and charge processes.

7. The authors mentioned performance several times and uses 'energy efficiency' in the contexts. However, when describing PLOB, it mentioned 'round-trip efficiency', and in the Methods part, it mentioned 'battery efficiency'. Are there any differences among these three? If so, please explain. If not, a unified statement needs to be made.

Response. We appreciate the reviewer for this insightful observation regarding the terminology for efficiency. In this work, "energy efficiency," "round-trip efficiency," and "battery efficiency" refer to the same core metric: the ratio of the total energy output during discharge to the total energy input during charge. To ensure clarity and consistency throughout the manuscript, we have adopted "energy efficiency" as the unified term. All instances in the main text, figure captions, and the methods section have been revised accordingly.

Reviewer #4 (Remarks to the Author):

This study presents a novel strategy for solar-rechargeable metal–air batteries via direct photoexcitation of sp-hybridized carbon units. By synthesizing nitrogen-substituted graphdiyne (NGDY) as a metal-free photocathode, the authors demonstrate significant performance enhancements across Zn-, Li-, Mg-, Fe-, and Al–air systems. The work provides compelling experimental and theoretical evidence regarding light-induced intramolecular charge redistribution and dynamic active site generation, offering valuable mechanistic insights into photo-coupled energy storage. Although the study is well-structured and supported by convincing data, certain aspects of the presentation and discussion require further clarification. Consequently, I recommend publication after minor revision.

Response. We appreciate the reviewer acknowledging the originality of our work. We have thoroughly considered all the helpful comments and have revised the manuscript and figures accordingly.

1. The direct photoexcitation of sp-C is a significant innovation. I suggest adding a schematic diagram contrasting this mechanism with traditional carrier separation to elucidate the distinction and enhance readability.

Response. In the revised manuscript, we introduce a new Fig. 3j (Figure R21) that contrasts the two paradigms across three fundamental aspects:

i. **Locus of Excitation:** Bulk band-gap generation of mobile carriers (conventional) vs. localized photoexcitation of specific sp-C motifs (this work).

ii. **Nature of the Active Site:** Pre-existing, static sites with unchanged electronic structure (conventional) vs. dynamic sites that are reconfigured in real-time by light (this work).

iii. **Origin of Enhancement:** Supplying external energetic oxidants (holes) to a static site (conventional) vs. intrinsically optimizing the catalytic property of the site itself (this work).

This schematic visually underscores the paradigm shift from using light as an external energy source to using it as a direct tool to reprogram the catalytic center.

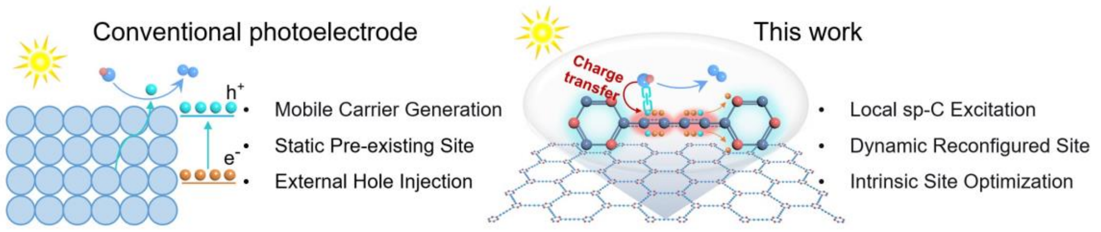

Figure R21. Schematic illustration of conventional photoelectrode and our proposed light-induced dynamic reconfiguration of sp-C.

2. The description of key illumination parameters—such as light intensity, spectral range, and spatial uniformity—is insufficient. It is recommended to provide these details in the Methods section to ensure experimental reproducibility and clarity.

Response. We have revised the Methods section to detail the key illumination parameters. We now specify the light source model, calibrated light intensity (measured with a photoradiometer) and spectral output range.

A 300 W xenon lamp (PLS-SXE 300, Perfectlight, China) served as the full-spectrum light source. For all photo-coupled battery tests, an 800 nm infrared filter (to attenuate infrared heating) was employed to tailor the incident spectrum to the ~320–800 nm range. The light intensity at the sample position was calibrated to 500 mW cm^{-2} using a photoradiometer (CEL-NP2000, Ceaulight).

Furthermore, we have provided the spectral profile of the light source (Supplementary Fig. 64) and photographs illustrating the illumination setup (Figure R22 to R23). These additions ensure all illumination conditions are clearly defined for replication.

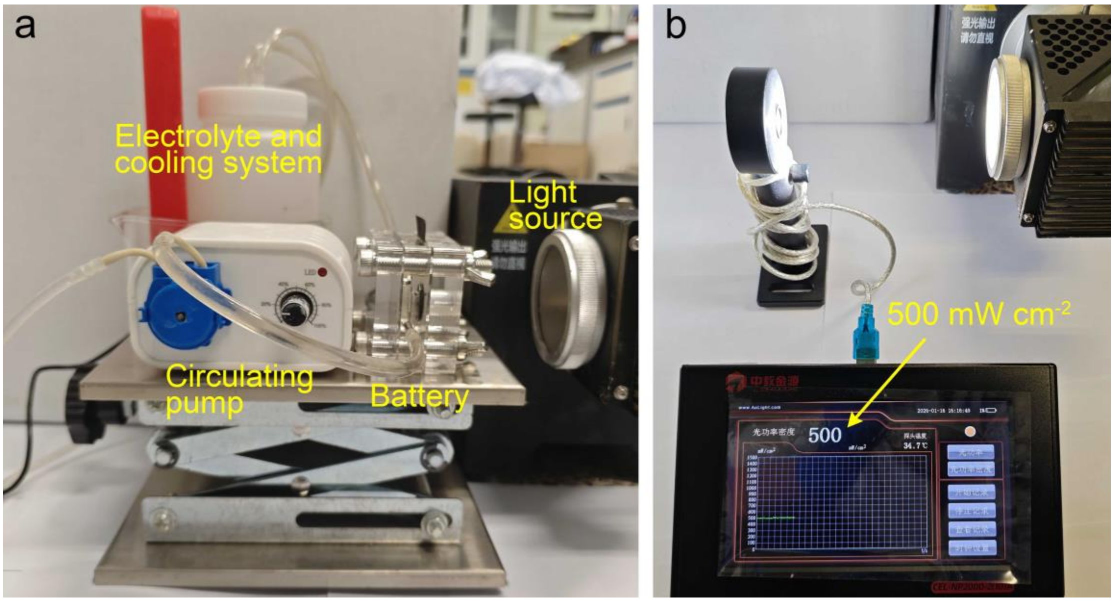

Figure R22. (a) The optical photograph of the working device of PZAB. (b) The light intensity was calibrated to 500 mW cm^{-2} using a photo radiometer.

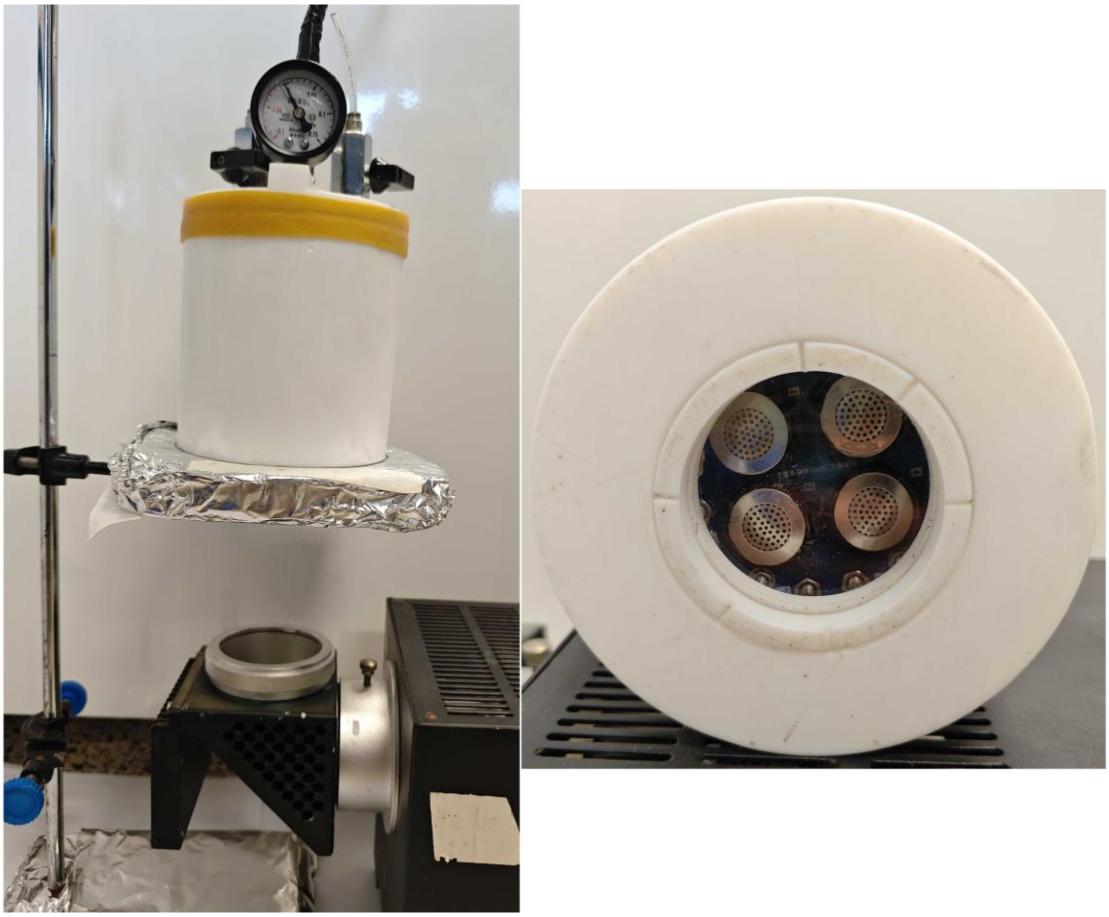

Figure R23. The optical photograph of the working device of PLOB.

3. The Tauc plot in Figure 2c indicates a bandgap of 1.87 eV, whereas DFT calculations yield 2.52 eV for the monolayer and 2.11 eV for the nine-layer model. Given the significant discrepancy between the experimental and computational values, the authors should discuss the potential origins of this difference.

Response. We have refined our DFT calculations by employing much denser k-points grid of 9×9×1 and much higher plane-wave cutoff energy of 500 eV to improve accuracy. The recalculated bandgaps are 2.35 eV for the monolayer model and 1.94 eV for the nine-layer stacked model (Figure R24), which are much closer with the experimental value. The remaining difference can be attributed to the following factors:

The experimental value (1.87 eV) is an optical bandgap derived from UV-vis absorption, which corresponds to the onset of direct or indirect electronic transitions that are dipole-allowed. The DFT-calculated value is the fundamental electronic bandgap. Differences between these two are common, especially in materials with complex excitonic effects or indirect band structures.

We have added a brief discussion of these points in the revised manuscript.

The remaining minor discrepancy between DFT-calculated bandgap and experimental bandgap arises from the differences between the fundamental electronic bandgap and the optically measured transition onset.

Importantly, the trend revealed by both theory and experiment—that nitrogen substitution widens the bandgap of graphdiyne—remains robust and central to our discussion on band engineering. The refined computational results (1.94 eV) now align reasonably with the experimental data within the expected margin of error for this level of theory, strengthening the consistency of our analysis.

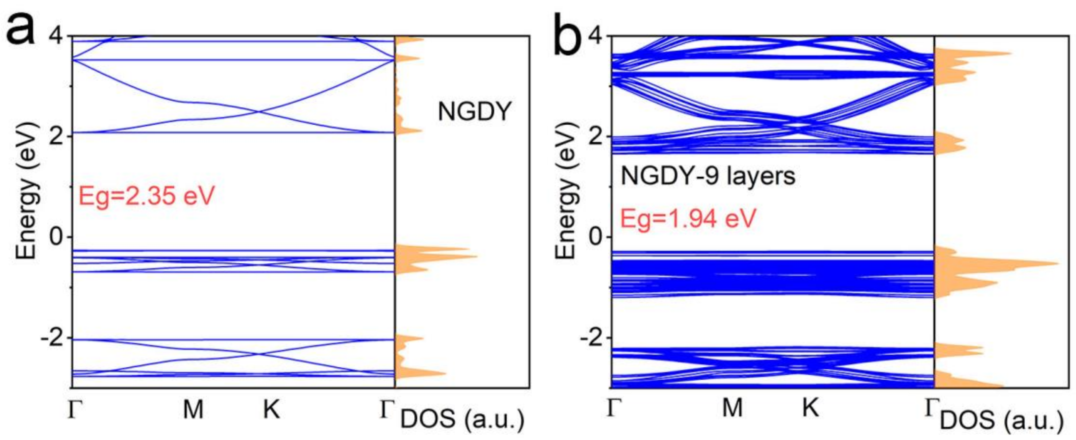

Figure R24. The calculated band structure and density of states of NGDY based on (a) monolayer and (b) 9-layer stacked model.

4. In Figure 2j, the authors report a photocurrent density of 120.2 μA cm^{-2} for the NGDY electrode without applied bias voltages. However, the key experimental parameters such as the illumination intensity, spectral range of the light source, and electrolyte composition are not provided. Additionally, a stability test should be included to demonstrate the robustness of the photoactive material.

Response. The photocurrent was measured in 1 M KOH electrolyte under zero applied bias with intermittent light irradiation. The illumination was provided by a 300 W xenon lamp with a spectral range of approximately 320–800 nm, and the light intensity was calibrated to 500 mW cm^{-2} at the electrode surface.

In the original submission, a short-term stability test of the NGDY electrode under intermittent illumination was included (Supplementary Fig. 20), and current–time curve showing stable operation for over 90 min after illumination. This demonstrates the material's robustness in the tested photoelectrochemical environment.

We have revised the Methods accordingly to incorporate the above clarifications and ensure full reproducibility. The updated text will read as follows:

The photocurrent was tested in 1 M KOH without applied potential and with

intermittence light irradiation. A 300W xenon lamp (PLS-SXE 300, Perfect Light, Beijing) was used as the light source. An 800 nm infrared filter was employed to tailor the incident spectrum to the ~320–800 nm range. The light intensity at the sample position was calibrated to 500 mW cm^{-2} using a photoradiometer.

5. Although NGDY exhibits a coulombic efficiency >96% after 230 cycles at 100 mA cm^{-2}, post-cycling structural characterization (e.g., XPS, TEM) is necessary to confirm material integrity. Specifically, the authors should address the stability of the sp-C bonds under prolonged photo-electrochemical coupling conditions?

Response. We thank the reviewer for the constructive suggestion to further validate the structural integrity, especially of the sp-C framework, under prolonged operation. We have conducted additional post-cycling characterizations to address this point comprehensively. XPS after battery cycling tests (Supplementary Fig. 35) confirms that the core chemical structure of the NGDY framework, including the characteristic signatures of its carbon network, remains intact after cycling at 100 mA cm^{-2}.

As presented in Supplementary Fig. 32 and 33, SEM and corresponding EDS mapping after long-term cycling show that the electrode morphology is preserved and the homogeneous distribution of C and N elements is maintained, indicating no bulk degradation or aggregation. Most pertinently, we performed post-cycling TEM/HRTEM analysis (Figure R25). The images clearly show that the layered crystalline structure of NGDY is retained. The measured lattice fringe spacing is 0.46 nm, and the preservation of clear lattice fringes confirms the robustness of the long-range ordered structure.

Collectively, the evidence from chemical state (XPS), morphology (SEM/EDS), and atomic-scale crystallinity (TEM) consistently demonstrates that the sp-C-based architecture of NGDY possesses exceptional structural integrity under harsh photo-electrochemical cycling conditions. This inherent stability is fundamental to its sustained high Coulombic efficiency. We have incorporated this discussion and the new

TEM data into the revised manuscript.

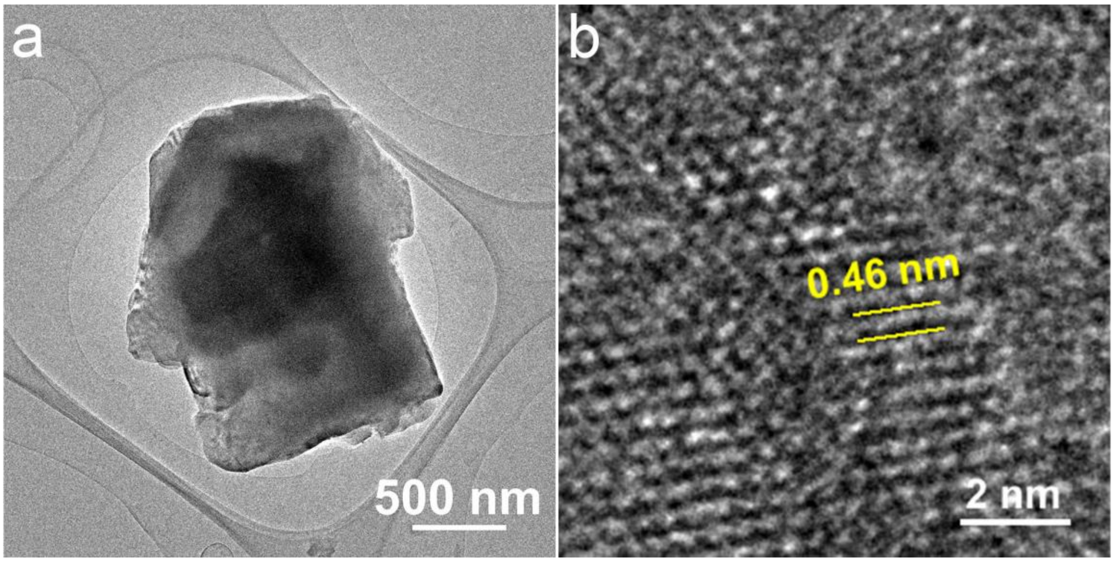

Figure R25. TEM images of NGDY after battery cycling tests.

6. The data in Supplementary Fig. 13 appear to be unprocessed. Please reprocess this figure appropriately.

Response. Supplementary Fig. 13 has been reprocessed accordingly. The original fluorescence lifetime decay curve has been fitted, and the updated figure now presents the fitted curve alongside the raw data, clearly displaying the fitting parameters.

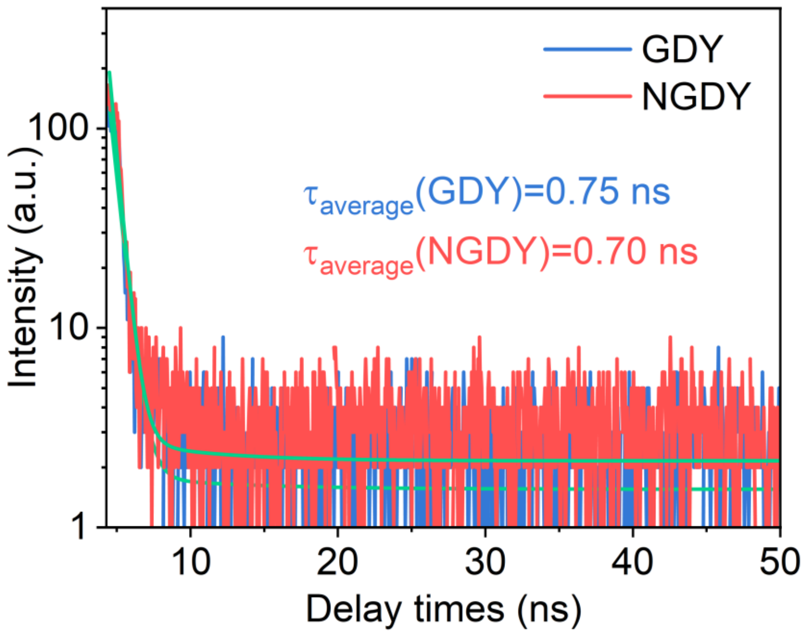

Figure R26. PL decay spectra measured at λ = 405 nm.

7. In large-scale applications, batteries are typically constructed as multilayer pouch or cylindrical battery. The authors should provide a reasonable model demonstrating how the proposed photocells can be effectively integrated into such configurations.

Response. We fully agree that bridging the gap between laboratory-scale proof-of-concept and industrially relevant cell formats is essential for the technology's future. While our current study focuses on the fundamental discovery of the novel sp-C photoexcitation mechanism and its validation in model cells, we have considered the integration strategy, which we outline below.

To illustrate the core integration concept, we have designed a simplified schematic model of a solid-state Zn-air battery (Figure R27). This model clearly depicts the essential layered structure: zinc foil anode, polymer gel electrolyte, the NGDY/CC-based photo-coupled air cathode and a functional air-diffusion layer that is perforated to serve a dual purpose: allowing oxygen diffusion while simultaneously permitting sunlight to reach the NGDY photocathode. This model, though simplified, validates the

key design principle: the seamless fusion of light-harvesting and air-breathing functions into a single, integrated electrode structure is feasible and forms the foundation for more complex device architectures.

Building on this principle, we can envision the pouch cell integration routes. This represents the most straightforward scaling path. The layered structure described above can be stacked into a multi-layer pouch. The primary engineering focus would be on the packaging: employing a transparent, high-barrier flexible window material (e.g., a tailored transparent composite film) on at least one side and carefully designing internal manifolds for both gas diffusion and light management.

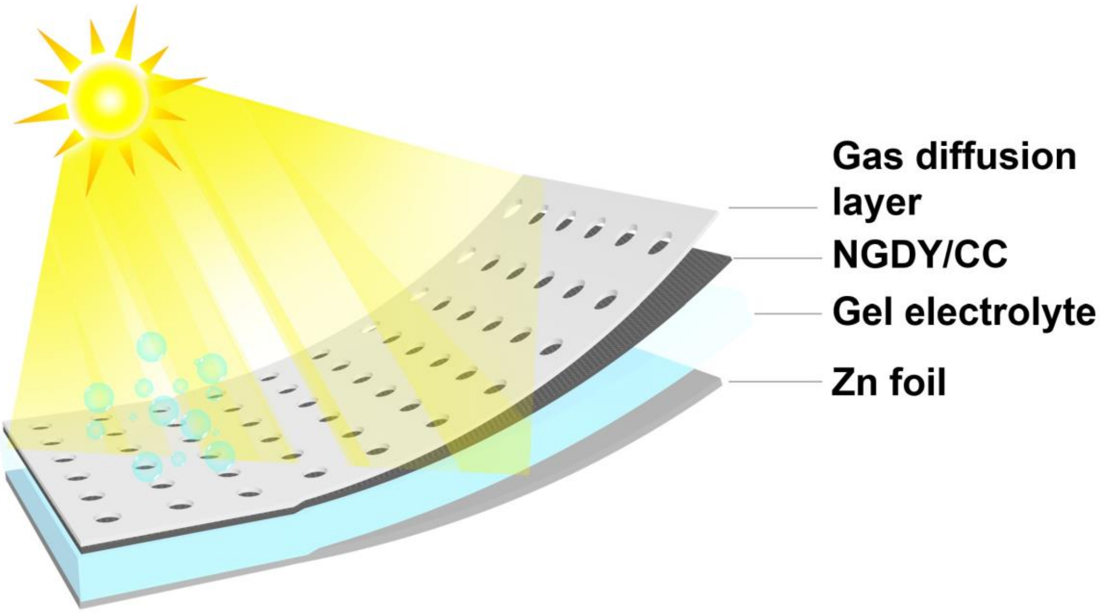

Figure R27. Schematic illustration of the structure of NGDY based solid state PZAB.

Point by Point Response to the Reviewers' Comments:

Reviewer #1 (Remarks to the Author):

The author has provided a good response and revision to the proposed opinions. However, there are still some details that need to be corrected. I suggest that the manuscript should undergo minor revisions again before it is officially accepted.

1. In Figure R9, the Gibbs free energy of the rate-limiting step at the C1 site is marked incorrectly. Please correct it.

Response. We thank the reviewer for the careful observation. The Gibbs free energy of the rate-limiting step at the C1 site is 1.78 eV and we have corrected it in the revised manuscript.

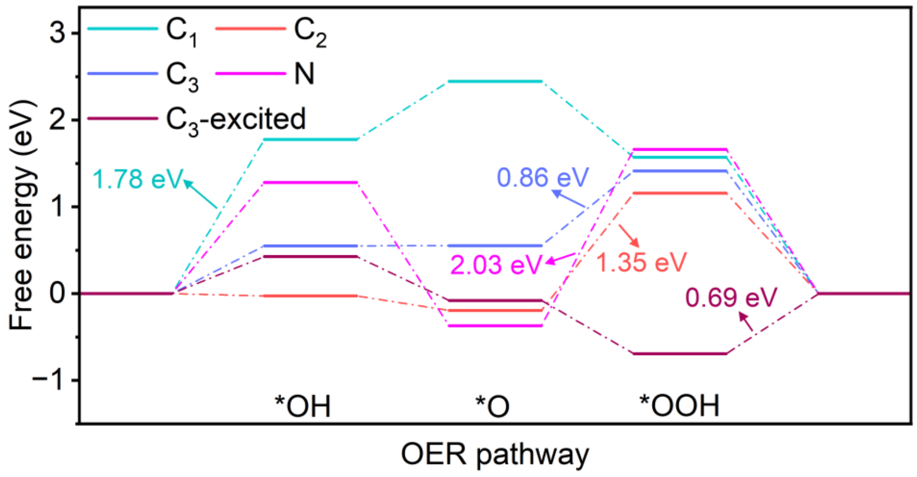

Figure R1. Free energy diagrams of different sites for OER.

2. In the calculation of the carrier mobility rate in the transient fluorescence photoluminescence spectrum, the author used a three-exponential decay fitting to obtain the average carrier mobility rate. Please provide the three carrier mobility rates obtained by the fitting and their corresponding proportions.

Response. In the initial manuscript, we wrote the average fluorescence lifetime formula in a general three-exponential form. However, during the fitting process, we found that

the decay curve could only be reliably fitted with a bi-exponential function; the three-exponential fitting did not converge or yielded statistically insignificant components. Therefore, in the revised manuscript, we have corrected this inconsistency by:

1. Using the correct general formula for intensity-weighted average lifetime:

$$\tau_{ave} = \frac{\sum B_i \tau_i^2}{\sum B_i \tau_i}$$

2. Presenting only the two physically meaningful components obtained from the bi-exponential fitting.

The detailed fitting results are as follows and have been added to the revised supplementary information:

Table R1. Solid fluorescence lifetime fitting data.

	τ_1	B_1	Rel%	τ_2	B_2	Rel%
GDY	0.5983	163.399	97.87	7.6175	0.279	2.13
NGDY	0.5308	206.815	96.71	5.6243	0.664	3.29

3. In Supplementary Fig. 11 and Figure 2c, the Eg between the theoretical calculation results and the actual obtained GDY band gap has increased. Can you explain the reason for this?

Response. The difference between the theoretical calculation results and the experimental band gap can be attributed to several factors:

i. Standard DFT calculations with generalized gradient approximation (GGA) functionals (such as PBE) are known to systematically underestimate bandgaps due to the incomplete treatment of electronic self-interaction. This is a common and expected source of discrepancy between DFT-GGA and experiment.

ii. The experimental value (1.87 eV) is an optical bandgap derived from UV-vis

absorption, which corresponds to the onset of direct or indirect electronic transitions that are dipole-allowed. The DFT-calculated value is the fundamental electronic bandgap. Differences between these two are common, especially in materials with complex excitonic effects or indirect band structures.

iii. The model may not fully capture all interlayer interactions or defects variations present in the actual synthesized GDY sample.

4. In the models shown in Figure 1k and 1i, the elements represented by each color of the balls are not indicated. Please mark them.

Response. We have marked the elements represented by each color of the balls in the revised manuscript.

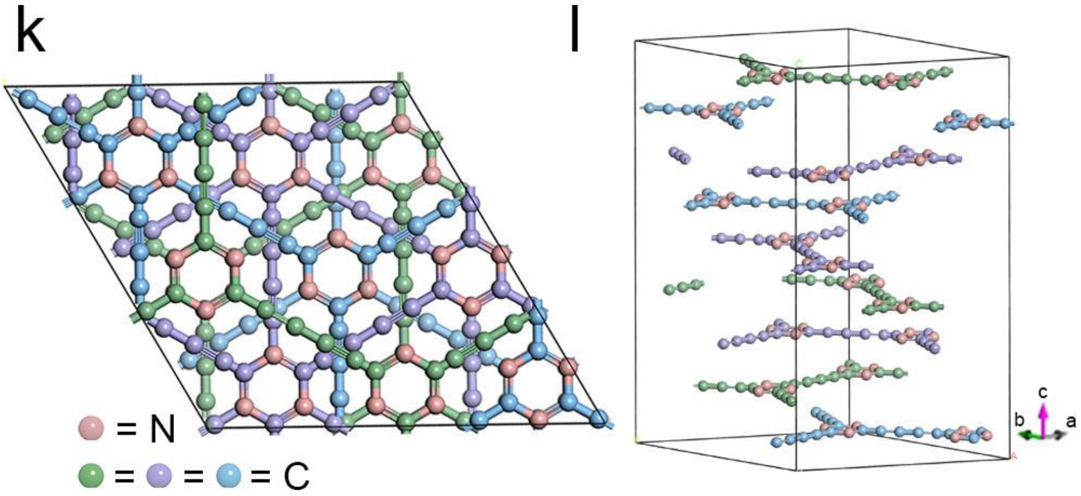

Figure R2. Top view and side view of the crystal structure of NGDY with 9-layers stacking structure.

Reviewer #2 (Remarks to the Author):

The revised manuscript "Direct solar energy charging applied to photo-coupled electrodes" tried to address the queries in detail, I would say, partially the authors could able to do so. But many things are still unanswered and a few concerns becoming more serious.

Response. We sincerely thank the reviewer for the critical evaluation of our work. These insightful comments have helped us identify key weaknesses and strengthen our mechanistic understanding. Below we address each point in detail.

Before going to the already raised concerns, one of the claims by the authors is high Coulombic efficiency, exceptionally high above 90%. This has to be really questioned as the air batteries (particularly Zn, Al etc. in alkaline medium), are known for their parasitic processes such as HER, ORR etc. in high alkaline medium, have low coulombic efficiency (unlike the conventional ion batteries). The details of Coulombic efficiency are missing from the article (or SI). Considering this as a known issue with conventional air batteries (though some reports say high Coulombic efficiency with MABs, but these are by addressing such parasitic issues by other means), this issue has not been well studied and mentioned in the manuscript, particularly the authors bring this as a novelty claim.

Response. We fully acknowledge that parasitic reactions such as the hydrogen evolution reaction (HER) are inherent challenges in aqueous metal–air batteries and have a significant impact on their Coulombic efficiency, which remains a critical and unresolved issue in metal–air battery research.

First, we wish to emphasize that the 96% CE reported in our work is not an exceptionally high or unprecedented value for Zn–air batteries under the specific conditions used. The commercial Pt/C+IrO_2 cathode also exhibits an CE above 96% in the first few cycles under identical test conditions (100 mA cm^{-2}, using a Cu current collector). Our study does not present high Coulombic efficiency as a novelty. Instead, the novelty lies in the stable retention of this high CE over 230 cycles under light irradiation, whereas the commercial catalyst and dark conditions show rapid decay. By comparing with dark conditions and commercial Pt/C+IrO_2, we emphasize the role of light in enhancing the cycling stability of the battery.

We also note that the severity of parasitic HER varies significantly depending on the anode metal: Zinc has a high hydrogen evolution overpotential in alkaline media, which

kinetically suppresses HER to a certain extent. As a result, moderate to high Coulombic efficiencies (typically 90–99%) have been reported in the literature under optimized conditions (e.g., high current density, proper electrolyte formulation)[1,2,3,4,5]. Our CE of 96% at 100 mA cm^{-2} is therefore consistent with these prior reports.

For Al–air and Mg–air batteries, these systems are indeed plagued by severe parasitic corrosion and HER, leading to much lower Coulombic efficiencies. In our work, we do not claim high CE for Al or Mg systems. We only demonstrate that the charging voltage is reduced under light in these systems, which is a separate and meaningful finding. The focus of our mechanistic study and high-CE demonstration is on the Zn–air system.

To our knowledge, while many studies on Zn–air batteries focus on charge–discharge cycling behavior, detailed and quantitative reporting of Coulombic efficiency remains rare, especially under high current densities. Where CE is mentioned, it is often discussed only qualitatively or without a clear description of the measurement protocol. In contrast, our work addresses this gap by providing a transparent, reproducible methodology and explicit CE data, contributing to a more rigorous understanding of reversibility in photo-coupled Zn–air batteries. As already described in the initial manuscript (Methods section):

To accurately assess the long-term operational stability, zinc deposition morphology evolution, and, crucially, the Coulombic efficiency (CE) of the Zn-air batteries at a high current density of 100 mA cm^{-2}, a specific testing protocol employing a copper foil as the anode current collector was adopted. This method replaces the conventional zinc foil and allows for the direct evaluation of zinc plating/stripping reversibility per cycle. The procedure is as follows: a polished copper foil was assembled as the working electrode (anode). In each cycle, the battery was first charged at a constant current density of 100 mA cm^{-2} for 60 s to deposit zinc onto the copper substrate. Subsequently, it was immediately discharged at the same current density until the deposited zinc was completely stripped, indicated by the cell voltage dropping to approximately 0 V, and the discharge time was recorded. The CE for each individual cycle was then calculated using the formula:

$$CE = \frac{Q_{discharge}}{Q_{charge}} \times 100\% = \frac{t_{discharge}}{t_{charge}} \times 100\%$$

Using this method, we obtained the CE values. Taking two of these cycles as an example, when the charging time is controlled at 60 s, the discharge times are 58.3 s and 57.7 s respectively, and the CE values are 97.2% and 96.2% respectively (Figure R3).

We attribute these relatively high CE values to our specific testing protocol. By depositing zinc onto the copper current collector at a high current density within a very short time (60 s) and immediately discharging to completely consume the deposited zinc, the entire process is completed rapidly. Under such a short timescale (60 s per half-cycle), the deposited zinc has insufficient time to undergo severe parasitic reactions such as HER, which are kinetically slower processes that typically become more pronounced over longer periods. Therefore, the CE values obtained under our fast-charging/fast-discharging conditions are higher than those commonly reported in the literature, where lower current densities and much longer cycle times often allow parasitic reactions to accumulate and significantly degrade the measured efficiency. This highlights that our high CE values are a reasonable result of the rapid testing protocol.

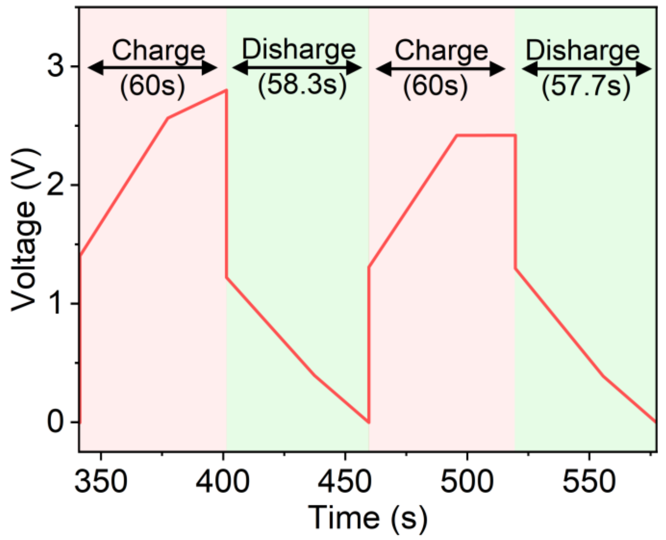

Figure R3. The cycling curves of PZAB using a copper foil as the current collector.

We hope this clarifies our position and addresses the reviewer's concern.

Different metal batteries have different set of issues in terms of capacity or energy density enhancement. Li air has cathode discharge product (LiO_2/Li_2O_2) storing/stabilising related issues while Zn air battery has issues related to sluggish kinetics of ORR/OER. Hence a study showing enhancement with all the air batteries, if it is true, may not help to prove the mechanism and role of NGDY.

Response. While the challenges (e.g., Li_2O_2 morphology, Zn dendrite formation) differ across MABs, they all share a fundamental bottleneck: the kinetics of the oxygen electrode reactions (ORR/OER) at the cathode. In all these systems, the charging process ultimately requires the oxidation of discharge products (OH^- in Zn–air, Li_2O_2 in Li–O_2) to evolve oxygen. This is precisely where the photoexcitation of NGDY exerts its primary effect: by generating transient, highly active sites and photogenerated holes, NGDY lowers the kinetic barrier for the oxygen evolution reaction itself.

The inclusion of Li–O_2, Mg–air, Fe–air, and Al–air batteries serve a distinct and important purpose: to demonstrate that the photo-enhancement effect is a general phenomenon rooted in the fundamental oxygen electrochemistry, rather than a system-specific artifact. If the effect were observed only in Zn–air batteries, one might argue that it arises from some peculiarity of the Zn/ZnO redox couple or the specific electrolyte. The fact that similar voltage reductions are observed across chemically diverse systems (non-aqueous Li–O_2, alkaline Mg/Fe/Al) provides powerful evidence for the underlying mechanism that photoexcitation of NGDY accelerating the oxygen evolution reaction.

Therefore, our multi-system demonstration is not intended to claim that NGDY solves each system's unique problems (e.g., Li_2O_2 passivation or Zn corrosion). Rather, it serves as proof of generality, while the Zn-air system serves as the platform for mechanistic understanding. This complementary approach strengthens, rather than dilutes, the overall conclusion: that photoexcited NGDY accelerates oxygen

electrochemistry across diverse metal–air batteries. We believe this balance of breadth and depth is a strength of the work, demonstrating both the robustness and the underlying mechanism of the phenomenon.

Again, being an established material, the novelty of it is again needs to be questioned.

Response. While NGDY has been previously synthesized,[6,7] the key novelty of our work lies not in the material itself, but in the discovery of a fundamentally new operating principle: the dynamic, photo-induced reconfiguration of sp-hybridized carbon networks to create transient, high-activity catalytic sites. This concept is distinct from conventional semiconductors photocatalysis in several ways.

For conventional semiconductors, the catalytic sites remain static, and the catalytic activity is an intrinsic property of the ground-state material. Overpotential reduction is often modest and attributed to photovoltage. But in our work, light directly excites the sp-C framework, causing intramolecular charge redistribution and the catalytic sites are dynamically created under light. The excited state has fundamentally different adsorption energetics, implying direct participation of excited states in the reaction coordinate.

These findings transform NGDY from a known composition into a platform for studying light-matter interactions at sp-carbon networks, opening a new paradigm for photo-coupled energy storage.

As I mentioned before, the thermal effect can happen in two ways: active and passive heating. By filtering the IR components in a spectrum, one may able to isolate the active heating. But the passive heating due to the absorption of the light by carbon or other components can still occur. This effect can be severe and the temperature can go to as higher as 55 degree celsius. Such an effect can be very different from the overall heating of the cell where, particularly in an aqueous battery, convection based mass transfer and

overall kinetics can be improved and hence having much pronounced effect.

Response. The carbon-based materials absorb light and undergo some degree of local heating. However, we have demonstrated that while photothermal effects cannot be completely excluded, we have minimized them as much as possible through experimental design. More importantly, even though such passive heating may contribute to battery performance to some extent, it cannot fully account for the remarkable magnitude and distinct nature of the performance enhancement observed in our system.

Firstly, the magnitude of passive heating is inherently limited under our conditions. The reviewer suggests that passive heating could raise the electrode temperature to as high as 55 °C. However, this estimate likely applies to systems without thermal management. In our experimental setup, several factors substantially limit passive heating: **Spectral filtering**: An 800 nm IR filter removes the most heating-efficient wavelengths (Supplementary Fig. 64.), significantly reducing the total absorbed power that converts to heat. **Active electrolyte circulation:** Continuous flow of the electrolyte rapidly dissipates any local heat generated at the electrode surface. **Thin electrode geometry:** The NGDY coating on carbon cloth is only a few tens of nanometers thick (Fig. 1), minimizing thermal mass and facilitating heat transfer to the flowing electrolyte.

Under these conditions, the actual temperature rise at the electrode surface is modest, which should be lower than 55 °C suggested by the reviewer. More importantly, as we demonstrate below, even this level of heating cannot explain our observations.

Secondly, we argue that photothermal contribution alone cannot explain the observed significant voltage reduction. To quantitatively assess the impact of thermal effects on the overall system performance, we have re-performed the controlled heating experiments by heating the entire battery to 60 °C in the dark, which is higher than any

plausible local heating under our illumination conditions:

1. EIS and distribution of relaxation time (DRT) analysis

We conducted electrochemical impedance spectroscopy (EIS) in the dark, under light irradiation and at 60 °C, and analyzed the data using distribution of relaxation time (DRT) method (Figure R4). In the DRT plot, each peak maximum represents a distinct electrochemical process contributing to the total polarization resistance of the battery, the time constant (τ) is characteristic of each polarization process, and the area under each peak corresponds to the polarization resistance contribution of that specific reaction to the total cell polarization. Therefore, changes in the DRT curves directly reflect variations in the nature and magnitude of the electrode reactions. The results reveal four main peaks in the DRT plot, corresponding to four major electrode processes or reactions. In the time constant region of 10^{-6} to 10^{-5} seconds (τ_1), which corresponds to electronic resistance, the peaks for dark at 25 °C and dark at 60 °C nearly overlap, while the peak under light is significantly reduced. This indicates that light excitation alters the electronic structure of NGDY, accelerating electron transport within the material. Heating alone at 60 °C in the dark does not produce this effect, confirming that the enhanced electronic transport is a genuine photoelectronic phenomenon, not a thermal artifact. In the time constant region of 10^{-2} to 10^{-1} seconds (τ_2), which relates to diffusion resistance, both light at 25 °C and dark at 60 °C show reduced peak areas compared to dark at 25 °C.

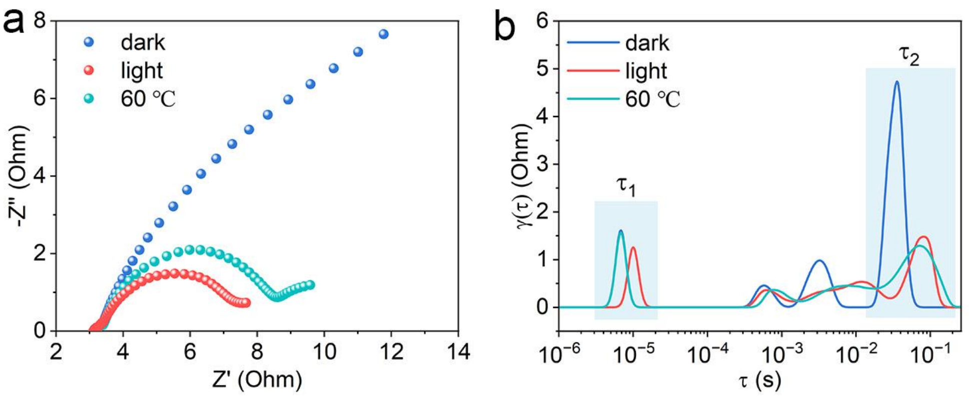

Figure R4. (a) EIS of PZAB in the dark, under light irradiation and at 60 °C. (b) DRT plots obtained by deconvolving the EIS.

This suggests that both light and heating enhance mass transport in the electrolyte, and the enhancement under light can be partially attributed to photothermal effects accelerating ion diffusion. So, just how significant is the contribution of heat to the improvement of battery performance? We have further provided the following data.

2. Temperature effect on charge

To further quantify the thermal contribution to charging voltage reduction, we measured galvanostatic charging curves at various current densities (1–20 mA cm^{-2}) under dark at 60 °C (Figure R5). The result shows that the charging voltage decreases from 1.96 V in the dark to 1.85 V at 60 °C. In striking contrast, under light at 25 °C, the charging voltage at the same current density dropped dramatically to 1.33 V—a reduction of 0.63 V. Critically, this 1.33 V under light is below the theoretical equilibrium potential of zinc-air batteries (1.65 V), which cannot be explained by enhanced mass transport or kinetics from heating alone. Such sub-thermodynamic charging voltage directly implicates the involvement of photo-generated charge carriers and photo-enhanced catalytic activity of NGDY. Crucially, this does not affect our core conclusion: the dramatic reduction in charging voltage cannot be explained by thermal effects alone and requires the intrinsic photoelectrochemical response of NGDY.

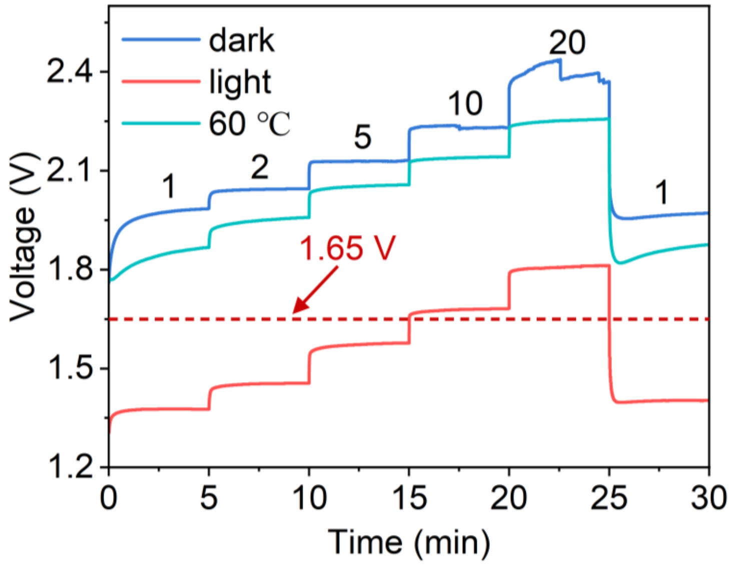

Figure R5. Charge curves at current densities from 1 mA cm^{-2} to 20 mA cm^{-2} based on NGDY in the dark, under light irradiation and at 60 °C.

In summary, we have taken extensive measures to minimize photothermal effects (IR filtering, active cooling, thin electrode geometry), and our controlled experiments quantitatively demonstrate that thermal contribution to charging voltage reduction is $\leqslant$0.11 V (at 1 mA cm^{-2}). The remaining >0.5 V reduction under light requires a photoelectronic mechanism, as sub-equilibrium charging (1.33 V < 1.65 V) is impossible via thermal effects alone. Moreover, DRT analysis confirms that light uniquely enhances electronic transport (τ_1), while heating only affects diffusion (τ_2).

Accordingly, we have added the following statement to the revised manuscript:

Moreover, the photothermal effects at the electrode/electrolyte interface would lower oxygen reaction barriers and accelerate charge/mass transfer,[58,59] *but they have been minimized through infrared filtering and active cooling. The predominant performance enhancement arises from the intrinsic photoelectrochemical response of NGDY.*

The references mentioned before from Prof Volder's groups and Prof Narayanan's group have also been added:

58. *Kumar, A. et al. Light vs Heat: Dissecting the De-intercalation in Photo-rechargeable Batteries. Nano Letters* ***25****, 628-634, (2025).*
59. *Pujari, A. et al. Does Heat Play a Role in the Observed Behavior of Aqueous Photobatteries? ACS Energy Letters* ***8****, 4625-4633, (2023).*

The wavelength dependent study shown in the revised manuscript is also not matched with the observed performance and the voltage enhancement is not coorelating. The absorption (quasi Fermi level shifting) with the observed battery performance needs to be discussed. I strongly believe that role of thermal effects is more pronounced than light effect, particularly in aqueous system, since the cathodic kinetics is known to be changed with temperature. The references need to be expanded with those addressed such thermal effects (as mentioned before from Prof Volder's groups and Prof Narayanan's group).

Response. In our wavelength-dependent study, we observed that the maximum performance enhancement occurs at 400 nm. As an organic semiconductor with sp-hybridized carbon networks, NGDY has a non-negligible exciton binding energy (ΔE_{EX}), which is the energy required to separate photogenerated electron-hole pairs into free charge carriers that can participate in electrochemical reactions. When photons with energy exactly matching the optical band gap (~1.87 eV) are absorbed, they generate bound excitons with relatively low dissociation efficiency (Figure R6). These excitons may recombine before contributing to quasi-Fermi level splitting or photovoltage generation.

As the photon energy increases above the band gap (toward 400 nm), the excess energy helps overcome the exciton binding energy, promoting efficient exciton dissociation into free electrons and holes. This leads to higher carrier densities and larger quasi-Fermi level splitting, which directly translates to a higher photovoltage and greater reduction in charging voltage. At even shorter wavelengths, while absorption remains strong, excess photon energy may be dissipated as heat through carrier thermalization, which can reduce the effective photovoltage contribution.

Therefore, the optimal wavelength for photoelectrochemical performance (400 nm) represents the point where the balance between absorption strength, exciton dissociation efficiency, and thermalization losses is most favorable.

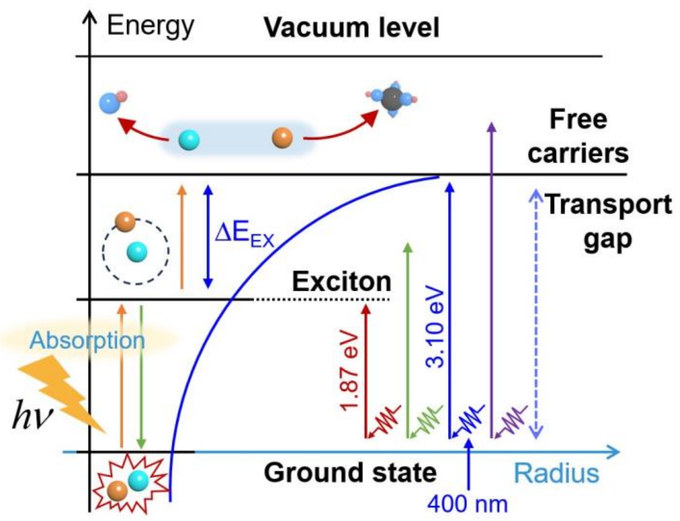

Figure R6. Schematics of the energy levels of NGDY.

Importantly, the fact that our performance peak occurs at the region where NGDY exhibits strong absorption, rather than showing a monotonic increase toward longer wavelengths, is itself evidence against a purely thermal mechanism. A thermal effect would produce a broadband response that increases with total absorbed energy, typically shifting toward the infrared where more photons are absorbed per unit energy. The presence of a distinct peak at a specific wavelength is a hallmark of an electronic, photoelectrochemical process governed by the material's band structure.

The reviewer also reiterates the concern that thermal effects may dominate, particularly in aqueous systems. We have carefully considered these important studies from Prof Volder and Prof Narayanan and have now cited them in our revised manuscript. In Prof Volder's work on aqueous zinc-ion batteries, the effect of light and heat on battery performance was systematically investigated. Interestingly, the discharge capacity increased only marginally from 112.6 mAh g^{-1} in the dark to 114.5 mAh g^{-1} under light irradiation, and heating alone enhanced the capacity to just 114.8 mAh g^{-1}. These improvements are very limited, which is consistent with our own controlled heating experiments shown in Figure R5, where the charging voltage decreased from 1.96 V at room temperature to only 1.85 V at 60 °C—a modest reduction of 0.11 V. In striking contrast, under light irradiation at room temperature, our NGDY-based battery exhibits a dramatic voltage reduction to 1.33 V. Such a substantial performance leap cannot be explained by thermal effects alone, as the magnitude of improvement far exceeds what heating can provide. This comparison further supports our conclusion that while thermal effects may contribute to a small extent, the dominant mechanism in our system is genuinely photoelectrochemical in nature.

It is worth noting that those works focus on lithium-ion batteries and aqueous zinc-ion batteries, where the electrochemistry involves ion intercalation/deintercalation processes. In contrast, metal–air batteries rely on oxygen electrocatalysis (OER/ORR) at the cathode, which is intrinsically more sensitive to the electronic structure of the catalyst. While thermal effects can enhance mass transport and general kinetics, they

cannot account for the specific changes in reaction pathway (Tafel slope), the sub-thermodynamic charging voltage (below 1.65 V), or the wavelength-specific response we observe. Our controlled heating experiments (discussed in detail in response to the previous concern) quantitatively demonstrate that the thermal contribution accounts for only a small fraction of the voltage reduction, and the remainder must arise from a genuine photoelectronic effect.

Furthermore, we note that photothermal materials have indeed been introduced into the air cathodes of metal–air batteries in some reports,[8,9,10,11] but this typically requires the material to possess broad and strong light absorption across the full solar spectrum to generate sufficient heat. In contrast, while NGDY exhibits good absorption in the visible region, it shows negligible absorption at wavelengths beyond 800 nm (Fig. 2b). This optical characteristic inherently limits its photothermal conversion efficiency under our experimental conditions, especially considering that we have already filtered out infrared components. Therefore, while we acknowledge the potential role of thermal effects in some battery systems, in our NGDY-based photo-coupled metal–air batteries, the predominant mechanism is photoelectrochemical in nature.

Since each battery system (air battery) has its own issues, my suggestion is to focus on one particular system in detail and establish the role of NGDY rather than showing everything but bot going to the details, which leave several doubts and concerns.

Response. We agree that focusing on one specific system to establish the role of NGDY in depth is an important principle for mechanistic studies. In the current manuscript, our intention in including multiple metal-air battery systems was to demonstrate the universality of the photo-enhancement phenomenon. The fact that similar voltage reductions are observed across chemically diverse systems (aqueous Zn, Mg, Fe, Al and non-aqueous Li-O_2) provides powerful evidence that the underlying mechanism is robust and not limited to a specific electrolyte or chemistry. However, we have been careful to maintain mechanistic depth by establishing Zn-air as the primary model

system for all in-depth investigations, including operando Raman spectroscopy, Tafel analysis, temperature-controlled kinetics, and DFT calculations. The other systems are presented more concisely as supporting evidence for the generality of the concept.

In subsequent work, we plan to focus on a single metal-air battery system to conduct an even more comprehensive investigation of NGDY's photo-coupled mechanism, including long-term stability and practical device optimization.

References

1. Bi, X. *et al.* Rechargeable Zinc–Air versus Lithium–Air Battery: from Fundamental Promises Toward Technological Potentials. *Adv. Energy Mater.* **14**, 2302388, (2024).
2. Liu, J.-N. *et al.* A brief history of zinc–air batteries: 140 years of epic adventures. *Energy Environ. Sci.* **15**, 4542-4553, (2022).
3. Huang, S. *et al.* A Dendrite-Resistant Zinc-Air Battery. *iScience* **23**, 101169, (2020).
4. Krishna, B. N. V., Ankinapalli, O. R., Ayyaluri, R. R. & Yu, J. S. Copper molybdenum sulfide-based rounded hollow cubes material as multifunctional electrocatalysts for zinc-air batteries and stable supercapacitors. *Journal of Energy Storage* **118**, 116148, (2025).
5. He, M. *et al.* Manipulating the ion-transference and deposition kinetics by regulating the surface chemistry of zinc metal anodes for rechargeable zinc-air batteries. *Green Energy & Environment* **8**, 318-330, (2023).
6. Kan, X. *et al.* Interfacial Synthesis of Conjugated Two-Dimensional N-Graphdiyne. *ACS Appl. Mater. Inter.* **10**, 53-58, (2018).
7. Yang, Z. *et al.* Graphdiyne Containing Atomically Precise N Atoms for Efficient Anchoring of Lithium Ion. *ACS Appl. Mater. Inter.* **11**, 2608-2617, (2019).
8. Li, J., Zhang, K., Wang, B. & Peng, H. Light-Assisted Metal-Air Batteries: Progress, Challenges, and Perspectives. *Angew. Chem. Int. Ed.*, e202213026, (2022).
9. Fang, Z. *et al.* Tactile UV- and Solar-Light Multi-Sensing Rechargeable Batteries with Smart Self-Conditioned Charge and Discharge. *Angew. Chem. Int. Ed.* **58**, 9248-9253, (2019).
10. Wang, S. *et al.* An extra-wide temperature all-solid-state lithium-metal battery operating from −73 ℃ to 120 ℃. *Energy Storage Mater.* **39**, 139-145, (2021).
11. Lin, Y., Yang, F., Wang, X., Zhong, L. & Yu, D. Organic Gradient Homojunction via D-A Engineering Enables Photoelectric/Photothermal Dual-Assisted Catalysis Toward Full Spectrum Light-Coupled Low-Temperature Seawater Batteries. *Adv. Mater.* **37**, 2415608, (2025).

Reviewer #3 (Remarks to the Author):

All of the issues raised in my previous review have been adequately addressed and resolved. I have no further concerns regarding this manuscript. In its current form, the manuscript is suitable for publication and can be accepted, provided that no additional major issues are raised by the other reviewers.

Response. We sincerely thank the reviewer for the valuable time and insightful comments.

Reviewer #4 (Remarks to the Author):

Authors answered all of my questions. I recommend its publication at the present state.

Response. We sincerely thank the reviewer for the valuable time and insightful comments.
